# Untargeted Metabolomics and GC–IMS Reveal Quality-Related Differences in Qilan Fresh Tea Leaves Grown in Wuyi and Baisha

**DOI:** 10.3390/foods15193478

**Published:** 2026-09-29

**Authors:** Longbin Zhong, Chanyi Xie, Mingchang Yang, Yibo Hu, Tianhang Huan, Zhigang Guang, Xia Xie, Yiyi Yin, Haiming Chen, Rongrong He

**Affiliations:** 1 HNU-HSF Collaborative Innovation Laboratory, College of Food Sciences & Engineering, Hainan University, Haikou 570228, Chinayangmingchanggd@163.com (M.Y.); qq3185125303@163.com (T.H.); hmchen168@126.com (H.C.); 2 Hainan University Research Development Office, Hainan University, Haikou 570228, China; 3 Hainan State Farms Tropical Products Industry Group Co., Ltd., Haikou 570226, China

**Keywords:** *Camellia sinensis* (L.) O. Kuntze cv. Qilan, regional cultivation, enzyme activity, antioxidant assays, volatile fingerprint, electronic nose, electronic tongue

## Abstract

In this study, fresh leaves of the same *Camellia sinensis* (L.) O. Kuntze cv. Qilan cultivar grown at similar elevations but contrasting latitudes in Wuyishan, Fujian (the 2L-FQ cultivation site), and Baisha, Hainan (the 2L-HQ cultivation site), were systematically compared using environmental characterization, enzyme activity assays, gas chromatography–ion mobility spectrometry (GC–IMS), and untargeted metabolomics. Black teas produced from the two groups of fresh leaves under identical standardized processing conditions were further evaluated using electronic nose and electronic tongue analyses. Correspondingly, 2L-HQ leaves were longer and wider than 2L-FQ leaves, with higher moisture content and water activity. The lower L*, b*, and chroma values of 2L-HQ, together with a ΔE*ab of 6.54, indicated a darker leaf appearance, which was accompanied by higher chlorophyll and carotenoid concentrations. Compared with 2L-FQ, 2L-HQ contained 11.8% more tea polyphenols, 26.4% more soluble polysaccharides, 11.4% more caffeine, and 10.6% more free amino acids. The non-gallated catechins catechin, epicatechin, epigallocatechin, and gallocatechin were significantly enriched in 2L-HQ. DPPH, ABTS, and FRAP antioxidant capacities were also 6.1%, 4.9%, and 2.2% higher in 2L-HQ, respectively. Lipoxygenase, polyphenol oxidase, and peroxidase activities increased by 137.0%, 98.0%, and 93.8%, respectively, whereas β-glucosidase activity remained unchanged. GC–IMS resolved 61 volatile compounds, of which 46 differed significantly between the two origins. 2L-FQ was enriched in several aromatic aldehydes, β-phenyl ethanol, and fruity esters, whereas 2L-HQ contained higher levels of selected alcohols, unsaturated aldehydes, furanic compounds, and lipid-derived esters. Untargeted metabolomics retained 4359 metabolic features, of which 4356 were shared by both groups. After multiple-testing correction, 793 differential metabolic features were identified using VIP > 1, FDR-adjusted *p* < 0.05, and |log_2_FC| > 1, including 420 features with higher abundance and 373 features with lower abundance in 2L-HQ relative to 2L-FQ. These differential features were mainly associated with carbohydrate, amino acid, flavonoid-related, lipid, and cofactor metabolism. Overall, regional cultivation was associated with coordinated differences in leaf morphology, hydration, pigmentation, taste-related composition, antioxidant capacity, oxidative-enzyme potential, volatile fingerprints, and metabolic allocation, providing a data foundation for region-adapted processing of Qilan tea leaves.

## 1. Introduction

Tea is one of the most widely consumed non-alcoholic beverages worldwide [1]. Fresh tea leaves are the primary raw materials for tea processing, and their physicochemical properties and metabolic profiles provide the chemical basis for final tea quality. Non-volatile compounds, such as catechins, caffeine, amino acids, organic acids, sugars, and flavonoids, contribute to bitterness, astringency, umami, sweetness, and aftertaste, whereas volatile compounds derived from lipid metabolism, carotenoid degradation, glycosidically bound precursors, and Maillard reaction-related pathways shape tea aroma [2,3,4]. Therefore, profiling the physicochemical properties and metabolites of fresh leaves is essential for evaluating their processing suitability and flavor-forming capacity.

In addition to cultivar and processing conditions, growing environments further shape the physicochemical and metabolic characteristics of tea leaves. Geographical factors, such as latitude and altitude, can shape local climatic conditions, including temperature, relative humidity, water availability, and light exposure. Together with soil properties, these environmental factors can influence tea secondary metabolism and modify the levels of polyphenols, amino acids, sugars, and aroma-related precursors [5,6]. For example, Tian et al. [7] used a metabolomics-based approach to show that cultivation altitude affected the metabolic profiles of tea leaves, particularly metabolites related to flavonoid and amino acid metabolism. Therefore, comparing the same cultivar grown under contrasting regional environments is a suitable strategy for evaluating environment-associated changes in fresh-leaf quality while reducing genetic variation.

Qilan (*Camellia sinensis* (L.) O. Kuntze cv. Qilan) is an oolong tea cultivar known for its potential to develop floral and fruity aromas. The cultivar is an important factor shaping the aroma quality of oolong tea, which is characterized by floral and fruity notes [8]. Previous studies have shown that oolong teas produced from different cultivars exhibit distinct volatile profiles and sensory characteristics [9,10,11]. However, most studies have focused on processed teas or processing-induced aroma changes. Systematic information remains limited on how contrasting cultivation environments across different regions reshape the physicochemical attributes, volatile fingerprints, and non-volatile metabolite profiles of Qilan fresh leaves.

GC–IMS is suitable for rapid volatile fingerprinting and sample discrimination, whereas untargeted metabolomics enables comprehensive profiling of global metabolite changes [9,12,13]. When combined with enzyme activity assays and targeted quality analysis, these approaches can link volatile fingerprints with upstream metabolic changes and fresh-leaf quality traits. Therefore, this study compared air temperature, relative humidity, precipitation, solar radiation, and wind speed in Fujian Wuyi and Hainan Baisha, together with Qilan fresh tea leaves quality traits, enzyme activities, volatile fingerprints, and metabolite profiles. By integrating environmental information, GC–IMS, and untargeted metabolomics, this study aimed to identify key metabolic changes associated with taste-related compounds and aroma precursors, thereby providing a scientific basis for region-specific processing and utilization of Qilan tea leaves.

## 2. Materials and Methods

### 2.1. Chemicals

Standard Rutin (≥99%), (−)-Epicatechin (EC), (−)-Epicatechin gallate (ECG), (−)-Epicatechin (EGC), (−)-Epicatechin gallate (EGCG), and (−)-Catechin (C) (≥98% purity) were purchased from McLean Chemical Company (Shanghai, China). Methanol, acetonitrile, and formic acid of chromatographic grade were obtained from McLean Chemical Company (Shanghai, China).

### 2.2. Plant Materials and Sampling

All samples were sourced from Hainan State Farms Baisha Tea Co., Ltd. (Baisha Li Autonomous County, China) and Mount Wuyi Yiqingerming Tea Industry Co., Ltd. (Wuyishan City, China).

In October 2025, fresh tea leaves consisting of one bud and two leaves were collected from the same tea cultivar, Qilan, at two sites in China. The first site was located in Baisha Li Autonomous County, Hainan Province, China (19.92° N, 109.58° E). This region has a tropical monsoon climate, with an annual precipitation of approximately 1725 mm. The second site was located in Wuyishan City, Fujian Province, China (27.93° N, 118.03° E). This region has a subtropical monsoon climate, with an annual precipitation of approximately 1894 mm; the mean temperatures in spring, summer, autumn, and winter were approximately 25.1, 27.5, 23.7, and 18.6 °C in Baisha and 18.1, 27.0, 20.2, and 9.3 °C in Wuyishan, respectively. The corresponding mean relative humidities were 78.7%, 80.7%, 85.3%, and 83.3% in Baisha and 78.7%, 79.0%, 73.0%, and 75.0% in Wuyishan, respectively. Qilan tea samples collected from Wuyishan, Fujian, and Baisha, Hainan, are designated 2L-FQ and 2L-HQ, respectively. Their corresponding cultivation sites are referred to as the 2L-FQ cultivation site and the 2L-HQ cultivation site.

All sampled tea plants were 13 years old. Within each plot, five sampling positions were arranged in a quincunx pattern: one central point and four peripheral points. At each sampling position, five healthy and uniformly growing tea bushes without visible symptoms of pests or diseases were randomly selected, and four shoots conforming to the one-bud-and-two-leaf standard were hand-picked from each bush. Thus, 20 shoots were collected at each sampling point. The shoots collected from the five positions within the same plot were combined in equal proportions and thoroughly mixed to form one composite biological replicate, whereas samples from different plots were kept separate and processed independently throughout the experiment. The samples were immediately frozen in liquid nitrogen and then stored at −80 °C until further analysis.

Fresh Qilan shoots from the two origins were processed separately into black tea using the same standardized orthodox manufacturing procedure, consisting of natural withering, rolling, enzymatic oxidation, and two-stage drying. Shoots conforming to the one-bud-and-two-leaf plucking standard were processed immediately after harvest. The shoots were spread in a thin layer in a shaded, well-ventilated room and allowed to wither for at least 24 h until the moisture content reached approximately 65% (w.b.). The withered leaves were then rolled for 60 min, producing tightly twisted strips with cell sap distributed over the leaf surfaces. After rolling, the leaves were loosened and spread to a uniform thickness of 3 cm for enzymatic oxidation. Enzymatic oxidation was conducted in a tea fermentation chamber (YY-FJ08, Youyuan, China) at 32 °C for 6–8 h, with the chamber relative humidity set to 95%. The endpoint was determined organoleptically and was reached when the leaves developed a uniform copper-red color and a pronounced sweet, honey-like aroma. The oxidized leaves were initially dried at 103 °C for 40 min and subsequently dried at 105 °C until the moisture content reached 3–5% (w.b.). After cooling to room temperature, the finished teas were sealed in aluminum-laminated bags and stored at 8 °C until electronic nose and electronic tongue analyses. Three independent processing batches were prepared for each origin.

### 2.3. Environmental Data Collection

Daily meteorological data, including air temperature, precipitation, relative humidity, solar radiation, and wind speed, were obtained from the NASA POWER database based on the geographic coordinates of each sampling site.

### 2.4. Phenotypic and Physiological Measurements

Fresh tea shoots, consisting of one bud and two leaves, were collected for physicochemical characterization and metabolomic analysis. The fifth fully expanded leaf from each Qilan plant was collected separately for morphological measurements. For each biological replicate, intact fifth fully expanded leaves were randomly selected. Leaf dimensions were measured using a digital caliper (DL90150, Deli Group Co., Ltd., Ningbo, Zhejiang, China) with a resolution of 0.1 mm and a manufacturer-specified accuracy of ±0.2 mm over a measurement range of 0–150 mm. Leaf length and width were measured using a digital caliper, and leaf thickness was determined at the central region of the leaf blade while avoiding the midrib. Leaf area was estimated using the empirical formula commonly applied to tea leaves [14]:

*LA* = *L* × *W* × 0.70 (1)

The leaf length-to-width ratio was calculated as:

*LWR* = *L*/*W* (2)

where *LA* is the estimated leaf area (cm^2^), *L* is leaf length (cm), *W* is leaf width (cm), and *LWR* is the leaf length-to-width ratio.

Leaf color was measured using a colorimeter (WSF-3J, Shanghai Yidian Scientific Instrument Co., Ltd., Shanghai, China) based on the CIE L* a* b* color system [15]. For each sample, at least ten intact fresh leaves were selected, and three points on the adaxial surface of each leaf were measured while avoiding the midrib. The mean values of L*, a* and b* were recorded. The total color difference, ΔE*ab, between fresh leaves from the two origins was calculated using the freshly harvested 2L-FQ as the reference:

ΔE*ab = [(L* − *L*_0_*)^2^ + (a* − *a*_0_*)^2^ + (b* − *b*_0_*)^2^]^1/2^ (3)

where *L*_0_*, *a*_0_* and *b*_0_* are the color parameters of the reference sample, and L*, a* and b* are those of the tested sample.

Moisture content was determined using an electronic halogen moisture analyzer (LC-DHS-16A, LICHEN, Xiamen, China). Approximately 3.0 g of fresh leaf samples, without prior cutting, were evenly distributed on the sample pan and dried at 101–105 °C until a stable mass was reached [16]. Moisture content was calculated from the mass loss and expressed on a wet basis. Measurements were performed in triplicate, and the mean value was used.

Water activity (aw) was measured using a water activity meter (AquaLab 4TE, Decagon Devices, Pullman, WA, USA) at 25.0 °C. Homogenized leaf samples were placed in the sample cup without compression, and readings were recorded after equilibrium was reached. All measurements were performed in triplicate.

Chlorophyll a, chlorophyll b, total chlorophyll, and carotenoid contents were determined using a chloroplast pigment content assay kit (G0613W, microplate method; Suzhou Geruisi Biotechnology Co., Ltd., Suzhou, China). Approximately 0.1 g of fresh leaf tissue was extracted according to the manufacturer’s protocol. The extraction solution was prepared from ethanol and distilled water at a volume ratio of 95:5, and the final extraction volume was adjusted to 10 mL. Aliquots (200 μL) of the extract and extraction buffer were transferred separately to a 96-well microplate as the sample and blank, respectively. Absorbance was measured at 665, 649, and 470 nm. Blank-corrected absorbance values were calculated as Δ*A_λ_* = *A_sampleλ_* − *A_blankλ_*. The pigment concentrations were calculated according to the manufacturer’s equations:

Chl a (mg g^−1^ FW) = *C_a_* × *V* × *D*/1000 × *W* (4)

Chl b (mg g^−1^ FW) = *C_b_* × *V* × *D*/1000 × *W* (5)

Total chlorophyll (mg g^−1^ FW) = *C_T_* × *V* × *D*/1000 × *W* (6)

Carotenoid (mg g^−1^ FW) = *C_c_* × *V* × *D*/1000 × *W* (7)

where *V* is the extraction volume (10 mL), *D* is the dilution factor, and *W* is the fresh sample mass (g). When the absorbance exceeded 1.0, the extract was diluted with extraction buffer, and the corresponding dilution factor was included in the calculation.

### 2.5. Determination of Quality-Related Components

Total polyphenol content was determined using a commercial total-phenol assay kit (Suzhou Geruisi Biotechnology Co., Ltd., Suzhou, China) based on the Folin–Ciocalteu colorimetric method. Briefly, 0.030 g of freeze-dried tea powder was extracted with 1.5 mL of 60% ethanol at 60 °C for 2 h. After extraction, the volume was restored to 1.5 mL with 60% ethanol, and the mixture was centrifuged at 12,000 rpm for 10 min at 25 °C. The supernatant was appropriately diluted before analysis. An aliquot of 40 μL was mixed with 200 μL of reagent I and incubated in the dark at 25 °C for 3 min, followed by the addition of 200 μL of reagent II and 360 μL of distilled water. After incubation at 25 °C for 30 min, absorbance was measured at 760 nm. Total polyphenol content was calculated using the standard curve supplied with the kit and expressed as mg g^−1^ DW. The official protocol uses 60% ethanol extraction and Folin-based detection at 760 nm.

Soluble polysaccharides were determined using a commercial polysaccharide assay kit (Suzhou Geruisi Biotechnology Co., Ltd.) based on the phenol–sulfuric acid method. Freeze-dried tea powder (3 mg) was mixed with 2 mL of distilled water and extracted in a boiling water bath for 2 h. After centrifugation at 8000 rpm for 5 min, 0.2 mL of the supernatant was mixed with 1.0 mL of ethanol and maintained at 4 °C for 1 h to precipitate the polysaccharide fraction. The precipitate was collected by centrifugation, washed with 1.0 mL of 80% ethanol, and redissolved in 2 mL of distilled water in a boiling water bath. Subsequently, 200 μL of the polysaccharide solution was mixed with 100 μL of reagent I and 500 μL of concentrated sulfuric acid. The mixture was heated at 95–100 °C for 20 min, cooled to room temperature, and measured at 488 nm. Polysaccharide content was calculated using a glucose standard curve and expressed as mg glucose equivalents g^−1^ DW. The kit protocol includes ethanol precipitation and washing before color development, thereby separating the polysaccharide fraction from most low-molecular-weight soluble sugars.

Free amino acid content was measured using a commercial amino acid assay kit (Suzhou Geruisi Biotechnology Co., Ltd.) based on the ninhydrin colorimetric method. Approximately 0.100 g of freeze-dried tea powder was homogenized with 1.0 mL of extraction buffer and centrifuged at 12,000 rpm for 10 min at 4 °C. An aliquot of 40 μL of the supernatant was mixed with 560 μL of reaction mixture and 40 μL of reagent III. The reaction mixture was heated in a boiling water bath for 15 min, cooled to room temperature, and mixed with 320 μL of 95% ethanol. After thorough mixing, 200 μL of the clarified solution was transferred to a 96-well plate, and absorbance was recorded at 570 nm using a microplate reader. Free amino acid content was calculated from the standard curve supplied with the kit and expressed on a dry-weight basis. The manufacturer’s protocol uses ninhydrin derivatization and detection at 570 nm.

Gallic acid, caffeine, and catechins were determined by HPLC in accordance with ISO 14502-2:2005, with minor modifications [17]. Freeze-dried leaf samples were ground into powder, and 0.100 g of powder was extracted with 70% methanol under ultrasonic conditions. The extract was centrifuged, filtered through a 0.22 μm membrane, and transferred into HPLC vials for analysis.

Chromatographic separation was performed on a C18 column (250 mm × 4.6 mm, 5 μm). The mobile phase consisted of solvent A and solvent B. Solvent A was prepared by mixing 92 mL of acetonitrile, 20 mL of acetic acid, and 2 mL of EDTA-2Na solution in a 1000 mL volumetric flask, and then diluting the mixture to volume with ultrapure water. Solvent B was prepared by mixing 800 mL of acetonitrile, 20 mL of acetic acid, and 2 mL of EDTA-2Na solution in a 1000 mL volumetric flask, and then diluting the mixture to volume with ultrapure water. Both solvents were filtered through a 0.45 μm membrane before use. The flow rate was 1.0 mL/min, the column temperature was maintained at 35 °C, the detection wavelength was 278 nm, and the injection volume was 10 μL. The gradient program was as follows: 0–10 min, 100% A; 10–15 min, a linear gradient from 100% A to 68% A; 15–25 min, 68% A and 32% B; and 25–30 min, 100% A for column re-equilibration. Quantification was performed using external calibration curves based on peak areas.

### 2.6. Antioxidant Assays

The DPPH radical-scavenging capacity, ABTS radical-scavenging capacity, and ferric-reducing antioxidant power were determined using commercial microplate kits (Suzhou Geruisi Biotechnology Co., Ltd., Suzhou, China) following the manufacturer’s procedures, which are based on the DPPH, ABTS, and FRAP principles, respectively [18,19,20]. For DPPH and ABTS assays, 0.050 g of freeze-dried leaf powder was extracted with 1.0 mL of 80% (*v*/*v*) methanol. For the FRAP assay, 0.050 g of freeze-dried powder was extracted with 1.0 mL of 80% (*v*/*v*) ethanol. Each suspension was sonicated at 60 °C and 200–300 W for 30 min and mixed at 5 min intervals. Any solvent loss was replenished to 1.0 mL with the corresponding extraction solvent. The extracts were centrifuged at 12,000 rpm for 10 min at room temperature, and the supernatants were used for the antioxidant assays.

For the DPPH assay, 150 μL of sample extract was mixed with 150 μL of DPPH working solution. A sample control containing 150 μL of extract and 150 μL of 80% methanol and a reagent blank containing 150 μL of 80% methanol and 150 μL of DPPH working solution were prepared in parallel. After incubation at 25 °C in the dark for 30 min, the mixtures were centrifuged at 12,000 rpm for 5 min, and 200 μL of each supernatant was transferred to a 96-well plate. Absorbance was recorded at 517 nm using a microplate reader (Synergy LX, Agilent Technologies, Inc., Santa Clara, CA, USA). For the ABTS assay, 10 μL of sample extract was mixed with 190 μL of ABTS working solution in a 96-well plate. The sample control contained 10 μL of extract and 190 μL of absolute ethanol, whereas the reagent blank contained 10 μL of absolute ethanol and 190 μL of ABTS working solution. The plate was incubated at 25 °C in the dark for 6 min, and absorbance was measured at 734 nm.

For the FRAP assay, the chromogenic working solution was freshly prepared by mixing the three kit reagents at a volume ratio of 10:1:1 and pre-equilibrated at 37 °C. Sample extract (5 μL), distilled water (25 μL), and chromogenic working solution (170 μL) were sequentially added to each well. The blank contained 30 μL of distilled water and 170 μL of chromogenic working solution. After reaction at 25 °C for exactly 10 min, absorbance was recorded at 590 nm. Trolox calibration ranges of 0–25 μg mL^−1^, 0–100 μg mL^−1^, and 0–3.6 μmol mL^−1^ were used for the DPPH, ABTS, and FRAP assays, respectively. The dilution factor was included in all calculations, and antioxidant capacities were converted using the molecular mass of Trolox (250.29 g mol^−1^) and expressed as μmol Trolox equivalents per gram of dry weight (μmol TE g^−1^ DW). Each biological replicate was extracted independently and measured in duplicate.

### 2.7. Enzyme Activity Assays

The activities of polyphenol oxidase (PPO), peroxidase (POD), lipoxygenase (LOX), and β-glucosidase were assayed using commercial kits (Suzhou Geruisi Biotechnology Co., Ltd., Suzhou, China). Fresh leaves were homogenized on ice with the extraction buffer provided in each kit. After centrifugation at 4 °C, the supernatants were collected as crude enzyme extracts. Absorbance was measured at the wavelength specified for each kit using a microplate reader. Enzyme activities were calculated according to the kit protocols and expressed as U/g fresh weight (FW).

### 2.8. Volatile Fingerprint Analysis by HS–GC–IMS

Volatile compounds in fresh Qilan tea leaves were analyzed using a headspace gas chromatography–ion mobility spectrometry system (HS–GC–IMS; FlavourSpec^®^ 25, G.A.S. Gesellschaft für analytische Sensorsysteme mbH, Dortmund, Germany). The analytical procedure was adapted from a previously reported HS–GC–IMS method for dry green tea, with modifications to the sample matrix, sample amount, incubation temperature, and internal-standard conditions [13]. An accurately weighed portion of 2.00 g was transferred into a 20 mL headspace vial, and 5 μL of 2-octanol internal-standard solution (1000 mg L^−1^) was added. The vial was immediately sealed with a magnetic screw cap fitted with a PTFE/silicone septum and incubated at 60 °C for 15 min with agitation at 500 rpm.

After equilibration, 500 μL of headspace gas was automatically withdrawn using a gas-tight syringe maintained at 85 °C and introduced into the GC–IMS system in splitless mode. Volatile compounds were separated on an MXT-5 capillary column (15 m × 0.53 mm i.d. × 1.0 μm film thickness; Restek Corporation, Bellefonte, PA, USA) maintained at 60 °C. High-purity nitrogen (≥99.999%) was used as both the carrier and drift gases. The carrier-gas flow was maintained at 2 mL min^−1^ for 2 min and then increased linearly to 100 mL min^−1^ over the subsequent 18 min. The drift tube was maintained at 45 °C, and the drift-gas flow was 150 mL min^−1^. The total analytical time was 20 min, and ion-mobility detection was performed in positive-ion mode.

Retention indices were calculated using a homologous series of n-ketones (C_4_–C_9_) analyzed under identical chromatographic conditions. Volatile compounds were tentatively identified by comparing the calculated retention indices and relative drift times with entries in the NIST 2020 retention-index database and the G.A.S. ion-mobility database. Monomeric and dimeric signals were retained as separate entries when they were clearly resolved. Raw data were processed using VOCal software (version 2.0.0, G.A.S., Dortmund, Germany). Two-dimensional topographic plots, difference plots, and gallery plots were generated to visualize the volatile fingerprints. Peak volumes were extracted to construct the data matrix used for the heatmap, hierarchical clustering, and principal component analyses. Three independent biological replicates were analyzed for each origin. The GC–IMS peak-volume data are provided in Table S1.

Internal-standard-based semi-quantification was performed according to:

*Ci* = (*A_i_*/*A_IS_*) × (*C_IS_* × *V_IS_*/*ms*) (8)

where *C_i_* is the relative concentration of compound *i*, *A_i_* and *A_IS_* are the peak volumes of compound i and 2-octanol, respectively, *C_IS_* is the concentration of the internal-standard solution, *V_IS_* is the volume of internal standard added, and *ms* is the fresh-leaf mass. Because 5 μL of a 1000 mg L^−1^ 2-octanol solution was added to 2.00 g of fresh leaves, the internal-standard level was equivalent to 2.50 μg g^−1^ FW. Results were expressed as μg 2-octanol equivalents g^−1^ FW. These values were regarded as semi-quantitative because compound-specific calibration curves and response factors were not established.

### 2.9. Untargeted Metabolomic Profiling

Untargeted metabolomic profiling was performed using freeze-dried tea leaf samples with minor modifications to previously reported tea metabolomics methods [21]. Briefly, tea samples were vacuum freeze-dried and then ground using a grinder (MM 400, Retsch, Haan, Germany) at 30 Hz for 1.5 min. Subsequently, powdered samples (50 mg) were mixed with pre-cooled 70% methanol solution (1200 µL). Intermittent extraction was performed using a vortex oscillator (VORTEX-5, Kylin-Bell, Nantong, China), with vortexing for 30 s every 30 min for a total of six cycles. After centrifugation at 13,400× *g* for 3 min using a centrifuge (5424R, Eppendorf, Hamburg, Germany), the supernatant was filtered through a 0.22 µm microporous membrane for UPLC-MS analysis using a UPLC system (LC-30A, Shimadzu, Tokyo, Japan) coupled to a TripleTOF 6600+ mass spectrometer (SCIEX, Foster City, CA, USA).

The UPLC-MS/MS was performed on a Waters ACQUITY Premier HSS T3 column (Waters Corporation, Milford, MA, USA) (1.8 µm, 2.1 mm × 100 mm), with0.1% formic acid aqueous solution used as mobile phase A, and 0.1% formic acid in acetonitrile used as mobile phase B. The separation was carried out at a flow rate of 0.4 mL/min, and the injection volume was 4 µL with the column temperature of 40 °C. The steps of gradient elution were as follows: 0–2 min, 5–20% B; 2–5 min, 20–60% B; 5–6 min, 60–99% B; 6–7.5 min, 99% B; 7.5–7.6 min, 99–5% B; 7.6–10 min, 5% B. Mass spectral full-scan data were in ESI (−) and ESI (+) modes with an acquisition time of 10 min, respectively. Spray ionization source temperatures were positive and negative at 550 °C and 450 °C. Ion spray voltages were 5000 V in positive ion mode and 4000 V in negative ion mode; Ion Source Gas1 was set to 50 psi; Ion Source Gas2 was set to 60 psi, and Curtain Gas was set to 35 psi. Quality control samples (QCs) were prepared by pooling aliquots of all sample extracts and were inserted regularly during instrumental analysis to assess analytical repeatability. Peaks with a missing rate greater than 50% in each sample group were filtered out, and missing values were imputed. After peak correction, metabolic features were putatively annotated by searching an in-house database, integrated public databases, predicted databases, and metDNA using MS and MS/MS information. Annotation confidence was evaluated using a composite identification score incorporating fragment-ion matching and spectral-library matching. Unless confirmed with authentic standards and retention-time matching, compound assignments were considered putative annotations rather than definitive metabolite identifications. Features with an identification composite score ≥ 0.5, MS1 mass error ≤ 25 ppm, MS/MS mass error ≤ 50 ppm, RT deviation ≤ 6 s, detection frequency ≥ 80% in biological replicates, and QC CV ≤ 30% were retained. The positive and negative ion modes were then merged, retaining the annotation with the highest confidence level and the lowest CV. Pooled QCs, prepared by combining equal aliquots from all sample extracts, were injected at regular intervals to monitor analytical stability [22].

### 2.10. Electronic Nose Analysis

Odor fingerprints of the finished Qilan black teas produced from 2L-FQ and 2L-HQ fresh leaves were obtained using a PEN3 portable electronic nose (AIRSENSE Analytics GmbH, Schwerin, Germany) with modifications to the previously reported tea-leaf procedures [8,23]. Fresh leaves were cut into approximately 5 mm pieces and mixed thoroughly. A 5.00 g portion was placed in a 50 mL airtight glass headspace vessel and equilibrated at 30 °C for 30 min. The electronic nose was preheated for 30 min before analysis, and the sensor chamber was purged with filtered clean air until all sensor responses returned to stable baseline values.

The PEN3 sensor array comprised ten metal-oxide-semiconductor sensors: W1C, W5S, W3C, W6S, W5C, W1S, W1W, W2S, W2W, and W3S. The operating parameters were as follows: automatic zeroing, 10 s; pre-sampling time, 5 s; measurement time, 70 s; cleaning time, 100 s; sampling interval, 1 s; chamber flow rate, 300 mL min^−1^; and sampling flow rate, 300 mL min^−1^. The headspace gas was drawn directly into the sensor chamber, and the conductance ratios generated by the instrument software were recorded. The mean response over the stable 65–70 s interval was used as the characteristic value for each sensor. Three independent biological replicates were analyzed for each origin, with three instrumental measurements per replicate. The sensor-response matrix was standardized before principal-component and hierarchical-clustering analyses.

### 2.11. Electronic Tongue Analysis

Taste fingerprints were measured using a cTongue voltammetric electronic tongue (BosinTech, Shanghai, China). Tea-infusion preparation was adapted from Zou et al. [23]. A 1.50 g portion of finely ground finished tea was infused with 75 mL of freshly boiled distilled water for 5 min. The infusion was filtered through four layers of gauze and cooled to 25 ± 1 °C, after which a 60 mL aliquot was transferred to the instrument sample beaker for analysis.

The electronic tongue was preheated for 20 min. Before the first measurement and between consecutive samples, the metal-electrode array was rinsed with ultrapure water for 30 s and electrochemically cleaned using the instrument’s potential-scanning program until a stable baseline was obtained. The manufacturer’s default pulse-voltammetry program was used. Signals were acquired for 120 s at 1 s intervals without a collection delay. Each sample was measured five consecutive times; the first and fifth measurements were discarded, and the three intermediate stable measurements were averaged. Three independently prepared biological replicates were analyzed for each origin.

### 2.12. Data Processing and Statistical Analysis

Data are expressed as mean ± standard deviation (SD). Statistical analysis was performed using GraphPad Prism 7.0 (GraphPad Software, La Jolla, CA, USA). The experiment followed a two-group comparative design, with growing origin as the fixed factor at two levels: the 2L-FQ and 2L-HQ cultivation sites. Three independent biological replicates were established for each origin using the five-point sampling method, and each replicate was pooled, processed, and analyzed independently. For analyses of the finished black teas, three independent processing batches were prepared for each origin under identical standardized manufacturing conditions. Technical or instrumental replicate measurements within each biological replicate were averaged before statistical analysis and were not considered independent biological replicates. Data are expressed as mean ± standard deviation (SD). Differences between 2L-FQ and 2L-HQ were evaluated using Student’s t-test, and *p* < 0.05 was considered statistically significant. For metabolomic data, raw files were converted to mzML format using ProteoWizard 3.x and processed with XCMS for peak extraction, alignment, and retention-time correction. Missing values were filtered and imputed, and peak areas were corrected. Differential metabolic features were defined using VIP > 1, FDR-adjusted *p* < 0.05, and |log_2_FC| > 1. PCA, OPLS-DA, a 200-permutation test, hierarchical clustering, volcano plots, and KEGG pathway enrichment were used to evaluate metabolic differences between the two groups.

## 3. Results and Discussion

### 3.1. Environmental Background and Phenotypic Traits of Fresh Tea Leaves

As shown in Table 1, the two sampling sites differed mainly in hydrothermal and radiation conditions rather than altitude. Although the two sites had similar elevations, the 2L-HQ cultivation site had warmer and wetter hydrothermal conditions, with higher mean air temperature, relative humidity, and total precipitation. In contrast, the 2L-FQ cultivation site received 13.28 MJ m^−2^ day^−1^ solar radiation compared with 11.44 MJ m^−2^ day^−1^. Surface and root-zone soil wetness were also slightly higher in the 2L-HQ cultivation site (0.84 and 0.82, respectively) than in the 2L-FQ cultivation site (0.78 and 0.79, respectively). These data indicate that the main environmental contrast between the two origins was a shift from a relatively cooler, brighter environment in the 2L-FQ cultivation site to a warmer, more humid, and lower-radiation environment in the 2L-HQ cultivation site. Such environmental factors are important regulators of fresh tea leaf quality, because temperature, rainfall, humidity, and light jointly regulate leaf growth, water status, pigment accumulation, and metabolite formation [21,24].

As shown in Figure 1B, consistent with these environmental differences, 2L-HQ exhibited markedly larger leaves than 2L-FQ. Leaf length increased from 7.89 cm in 2L-FQ to 10.05 cm in 2L-HQ, while leaf width increased from 3.40 to 4.38 cm. As a result, the leaf area of 2L-HQ was substantially higher than that of 2L-FQ. The water-related traits showed the same direction of change. Moisture content increased from 74.41% in 2L-FQ to 76.75% in 2L-HQ, and water activity increased from 0.979 to 0.988. Because the 2L-HQ cultivation site had higher rainfall, relative humidity, and soil wetness, water limitation during shoot expansion was likely reduced. In tea plants, water deficit is generally associated with reduced leaf area, lower leaf water content, and impaired physiological performance, whereas more favorable water supply supports tissue expansion and biomass accumulation [25,26]. Therefore, the larger leaf size and higher tissue hydration observed in 2L-HQ were likely associated with the warmer, wetter 2L-HQ cultivation site environment. Although wind speed was higher in the 2L-HQ cultivation site, the higher relative humidity and greater soil wetness may have mitigated excessive transpirational water loss, allowing fresh shoots to maintain a higher hydration status.

The color and pigment data further supported this interpretation. Compared with 2L-FQ, 2L-HQ showed a lower L* value (39.11), lower b* value (67.41), and lower c* value (67.66), together with an overall color difference of ΔE*ab = 6.54. Thus, fresh leaves from the 2L-HQ cultivation site were visually darker than those from Wuyishan. This visual difference corresponded to higher pigment contents in 2L-HQ, including chlorophyll a (3.96 mg g^−1^ FW), chlorophyll b (1.87 mg g^−1^ FW), total chlorophyll (9.20 mg g^−1^ FW), and carotenoids (0.89 mg g^−1^ FW). The stronger increase in chlorophyll b than chlorophyll a is noteworthy because chlorophyll b is closely associated with light-harvesting antenna complexes and often increases under lower-light conditions. Tea studies have shown that shading or reduced light intensity can produce larger, greener leaves and promote the accumulation of chlorophyll a, chlorophyll b, and total chlorophyll, thereby improving green coloration [27]. Previous studies have also shown that shading can enhance carotenoid accumulation in tea leaves [28]. In addition, temperature may have contributed to the pigment differences observed here. The lower minimum temperature in Wuyishan, especially the much lower monthly minimum temperature, may have limited pigment accumulation, because low-temperature conditions can reduce chlorophyll content and alter chloroplast development in tea leaves [29]. Therefore, the visually darker appearance and higher pigment concentrations of 2L-HQ may reflect the combined effects of lower radiation, higher humidity, and warmer minimum temperatures in the 2L-HQ cultivation site.

Taken together, the environmental and phenotypic data indicate that the 2L-HQ cultivation site grown Qilan leaves were associated with a warmer and wetter growing environment and exhibited greater leaf expansion, higher tissue hydration, and greater pigment accumulation than the 2L-FQ cultivation site grown leaves. These region-associated differences are important because fresh-leaf morphology, water status, and pigment composition provide the physiological basis for later processing performance and final tea quality. In particular, larger and more hydrated leaves may respond differently during withering and rolling, and higher chlorophyll and carotenoid levels may influence both fresh-leaf appearance and the availability of aroma-related precursors during subsequent tea manufacture. Because one site was examined per region, these associations do not establish independent effects of rainfall, humidity, radiation, or temperature.

### 3.2. Major Quality-Related Components

According to Table 2, the two origins differed not only in the overall abundance of quality-related compounds, but also in the distribution of individual catechins. Tea polyphenol content was 219.67 ± 2.46 mg g^−1^ DW in 2L-HQ, 11.8% higher than the 196.42 ± 1.75 mg g^−1^ DW measured in 2L-FQ. This increase was mainly associated with non-gallated catechins. Compared with 2L-FQ, 2L-HQ contained 34.2% more catechin (C), 144.5% more epicatechin (EC), 33.4% more epigallocatechin (EGC; 17.63 ± 0.32 vs. 13.22 ± 0.24 mg g^−1^ DW), and 35.7% more gallocatechin (GC). By contrast, catechin gallate (CG) was lower in 2L-HQ, while epicatechin gallate (ECG), gallocatechin gallate (GCG), and epigallocatechin gallate (EGCG) did not differ significantly between the two groups. 2L-HQ accumulated a larger pool of non-gallated catechins without a corresponding increase in the major gallated catechins.

Gallated catechins generally elicit stronger astringency than their non-gallated counterparts; ECG and EGCG, for example, exhibit greater perceived astringency than EC and EGC. Accordingly, the higher total polyphenol content of 2L-HQ should not be interpreted as a proportional increase in overall astringency. Instead, it reflects a higher phenolic concentration combined with a different catechin profile. The marked enrichment of EC, EGC, C, and GC may increase the contribution of non-gallated catechins to bitterness and antioxidant capacity. However, because EGCG and ECG did not increase significantly, the higher total polyphenol content may not result in a comparable increase in the pronounced astringency typically associated with gallated catechins [30].

Other taste-related constituents also tended to be more abundant in 2L-HQ. Caffeine increased from 9.26 ± 0.22 mg g^−1^ DW in 2L-FQ to 10.32 ± 0.17 mg g^−1^ DW in 2L-HQ, corresponding to an increase of 11.4%. Soluble polysaccharides showed a larger increase, rising from 41.61 ± 1.79 to 52.58 ± 0.98 mg g^−1^ DW, equivalent to a 26.4% increase. Free amino acid levels were also numerically higher in 2L-HQ than in 2L-FQ. Caffeine contributes directly to bitterness, whereas free amino acids, particularly theanine and glutamate, are associated with umami, freshness, and overall taste quality. Studies of Chinese teas have shown that higher free amino acid levels are generally associated with improved sensory quality. However, the overall taste profile is determined by the balance among free amino acids, polyphenols, and caffeine rather than by the concentration of any single component [31].

The concurrent increases in caffeine, total polyphenols, free amino acids, and soluble polysaccharides indicate that 2L-HQ did not exhibit a unidirectional shift toward a milder taste profile. Instead, its composition suggests enhanced contributions from several potentially opposing taste-related components. Higher caffeine and phenolic levels may increase bitterness and astringency, whereas the greater abundance of free amino acids may enhance umami and freshness. Certain tea polysaccharides and polysaccharide conjugates may also contribute to infusion body and mellowness. However, these sensory effects depend on molecular composition, conjugation state, extractability, and processing conditions and therefore cannot be inferred directly from total soluble polysaccharide content alone. Accordingly, the present results suggest a potentially fuller and more compositionally complex taste basis for 2L-HQ, rather than an unequivocally milder taste profile. Direct sensory evaluation and analysis of the processed tea infusion would be required to confirm this interpretation [31].

The antioxidant assays consistently indicated greater antioxidant capacity in 2L-HQ. DPPH radical-scavenging capacity increased from 577.65 ± 6.35 μmol TE g^−1^ DW in 2L-FQ to 612.80 ± 7.83 μmol TE g^−1^ DW in 2L-HQ, while ABTS radical-scavenging capacity increased from 684.35 ± 7.21 to 717.62 ± 8.46 μmol TE g^−1^ DW. Although the between-group difference in FRAP was smaller than those observed for DPPH and ABTS, FRAP remained significantly higher in 2L-HQ. The concordant results across the three assays indicate that the greater antioxidant potential of 2L-HQ was evident in both radical-scavenging and ferric-reducing systems, rather than being specific to a single analytical mechanism.

The greater antioxidant capacity observed in 2L-HQ was consistent with its higher total polyphenol content. Catechin composition can substantially influence DPPH, ABTS, and FRAP responses. However, the antioxidant capacity of a tea extract reflects the combined contributions and potential interactions of catechins, gallic acid, flavonoids, caffeine, and other reducing compounds. Lee et al. [32] similarly reported that variation in the concentrations of major catechins was associated with differences in antioxidant capacity measured using these three assays. Accordingly, the present results indicate greater in vitro antioxidant capacity in 2L-HQ, but the observed difference cannot be attributed to any single catechin alone [32].

Compared with the 2L-FQ cultivation site, the 2L-HQ cultivation site had a higher mean air temperature, greater relative humidity and precipitation, and higher surface and root-zone soil wetness, but received less solar radiation. Because the two sites were located at similar elevations, their environmental contrast was primarily hydrothermal and radiation-related rather than altitudinal. Reduced light exposure in tea plants is generally associated with greater amino acid retention and lower flavonoid and catechin biosynthesis. Controlled-shading studies have shown that reduced irradiance can suppress UVR8-mediated flavonoid biosynthesis and decrease the accumulation of several catechins, whereas temperature and light jointly regulate carbon and nitrogen allocation in developing tea shoots [33,34]. The numerically higher free amino acid content of 2L-HQ was consistent with this general response to reduced irradiance. However, its higher total polyphenol and non-gallated catechin contents did not conform to a simple shading-response model. This divergence indicates that solar radiation alone cannot explain the observed chemical differences. Warmer minimum temperatures, greater water availability, soil conditions, leaf developmental status, and interactions among these factors may have jointly modified carbon allocation and phenylpropanoid metabolism in the 2L-HQ cultivation site grown leaves. Multi-site studies using the same tea cultivar likewise indicate that fresh-leaf catechin and amino acid profiles are shaped by interacting climatic, edaphic, and geographical factors rather than by any single environmental variable [24]. Accordingly, the higher levels of total polyphenols, non-gallated catechins, soluble polysaccharides, and antioxidant capacity in 2L-HQ are more appropriately interpreted as an integrated origin-associated chemical profile than as a direct response to reduced solar radiation alone.

### 3.3. Enzyme Activity Profiles

According to Figure 2, the enzyme-activity data showed a more pronounced regional contrast than most individual quality components. LOX activity increased from 720.00 ± 52.92 U g^−1^ FW in 2L-FQ to 1706.67 ± 94.52 U g^−1^ FW in 2L-HQ, corresponding to a 137.0% increase. PPO activity increased from 66.67 ± 6.11 to 132.00 ± 4.00 U g^−1^ FW, while POD activity increased by 93.8%, from 5.33 ± 0.58 to 10.33 ± 1.53 U g^−1^ FW. By contrast, β-glucosidase activity changed only slightly, from 890.82 ± 6.40 to 922.23 ± 19.54 U g^−1^ FW, and the difference was not statistically significant. The regional enzyme response was therefore concentrated in lipid oxidation and phenolic oxidation rather than in the hydrolysis of glycosidically bound aroma precursors.

The largest difference occurred in LOX. This enzyme catalyses the initial oxygenation of linoleic and α-linolenic acids, producing hydroperoxides that can be cleaved through the LOX–HPL pathway and subsequently converted into aldehydes, alcohols, acids, and esters. The higher LOX activity of 2L-HQ therefore indicates a greater upstream capacity for lipid-derived aroma formation when leaf tissues are disrupted during withering, bruising, or rolling. It should not, however, be treated as a direct measure of the abundance of each final volatile compound, because downstream metabolic partitioning also depends on substrate availability, hydroperoxide lyase, alcohol dehydrogenase, aldehyde dehydrogenase, acyltransferases, and oxygen conditions [35].

PPO and POD showed almost parallel increases in 2L-HQ. Both enzymes participate in the oxidation of tea phenolics after cellular compartmentalisation is disrupted. PPO initiates catechin oxidation and contributes to the formation of quinones and theaflavin precursors, while POD can further participate in phenolic oxidation in the presence of hydrogen peroxide. Experimental work has shown that both enzymes influence the generation and subsequent transformation of black-tea pigments [36]. The higher activities observed in 2L-HQ therefore indicate greater potential for rapid phenolic conversion during processing. Higher PPO and POD activities may affect processing responsiveness, particularly the rate of catechin oxidation after rolling. However, the extent and composition of oxidation-derived pigment formation also depend on moisture loss, oxygen availability, tissue disruption, temperature, processing duration, and the relative availability of different catechin substrates. Therefore, the enzyme activity results indicate origin-related differences in oxidative potential but do not demonstrate that 2L-HQ would invariably produce a higher-quality or more extensively oxidised tea under all processing conditions.

The 2L-HQ cultivation site leaves developed under warmer, wetter, and more humid conditions and also showed greater leaf hydration, whereas the 2L-FQ cultivation site material experienced greater solar radiation and substantially lower minimum temperatures. Temperature, light, water status, and soil conditions can jointly alter tea-leaf primary metabolism, secondary metabolism, membrane-lipid turnover, and stress-related responses [24,34]. These environmental contrasts are compatible with the larger LOX, PPO, and POD activities observed in 2L-HQ, but the current dataset cannot establish which factor regulated each enzyme.

### 3.4. GC–IMS Volatile Fingerprints of Qilan Fresh Leaves

According to Table 3, GC–IMS analysis tentatively assigned signals to 61 volatile compounds in Qilan fresh leaves, including 13 aldehydes, 10 alcohols, 11 esters, 5 ketones, 6 furanic compounds, 6 ethers and acetals, 3 heterocyclic compounds, 2 acids, 2 sulfur-containing compounds, 2 hydrocarbons, and 1 phenolic compound. Figure 3C showed relatively consistent signal patterns among the three biological replicates within each origin, whereas numerous signal regions differed between 2L-FQ and 2L-HQ. PCA further confirmed the separation of the two groups, with PC1, PC2, and PC3 explaining 74.9%, 13.9%, and 6.8% of the total variance, respectively. The cumulative contribution of the first three components reached 95.6%, indicating that the model captured most of the variation in the volatile dataset. Studies have demonstrated that GC–IMS fingerprinting combined with multivariate analysis can effectively distinguish tea samples from different cultivars or geographical origins, as well as samples produced under different processing conditions, based on their volatile profiles [9,13].

Among the 61 detected compounds, 46 showed significant differences between 2L-FQ and 2L-HQ. Of these, 35 compounds were more abundant in 2L-FQ, whereas 11 were more abundant in 2L-HQ. These results suggest that the differences in volatile profiles between the two origins were mainly driven by quantitative changes in shared compounds, rather than by the presence or absence of a limited number of origin-specific volatiles. This pattern agrees with previous tea-aroma studies showing that differences among cultivars and growing regions are generally driven by changes in the relative proportions of aldehydes, alcohols, esters, ketones, and terpenoid-related compounds [9,24]. Compared with 2L-HQ, 2L-FQ contained significantly higher concentrations of several aldehydes. The compound 2-hexenal decreased from 19.01 ± 0.65 μg g^−1^ in 2L-FQ to 7.90 ± 0.75 μg g^−1^ in 2L-HQ. Benzeneacetaldehyde decreased from 19.51 ± 1.18 to 14.54 ± 0.88 μg g^−1^, while n-nonanal and (Z)-6-nonenal decreased from 9.60 ± 0.56 and 9.00 ± 0.54 μg g^−1^ to 5.72 ± 0.57 and 5.99 ± 0.42 μg g^−1^, respectively. Citronellal was also significantly higher in 2L-FQ than in 2L-HQ. These compounds are generally associated with green, citrus-like, melon-like, sweet, or oily aroma characteristics and may contribute to the fresher and more aromatic volatile background of 2L-FQ [9,35]. Tentative assignments and semi-quantitative abundances of GC-IMS volatile signals. Abundances are expressed as μg 2-octanol equivalents g^−1^ FW. Odor descriptors are drawn from library information and do not establish sensory contribution in the analyzed tea leaves.

Several aromatic alcohols and fruity esters were also enriched in 2L-FQ. β-Phenyl ethanol reached 19.28 ± 0.90 μg g^−1^ in 2L-FQ but only 7.91 ± 0.63 μg g^−1^ in 2L-HQ. Methyl octanoate decreased from 8.17 ± 0.19 to 3.59 ± 0.34 μg g^−1^, butyl butanoate decreased from 9.36 ± 1.08 to 1.76 ± 0.05 μg g^−1^, and 2-methylpropyl butanoate decreased from 12.07 ± 1.28 to 4.31 ± 0.70 μg g^−1^. β-Phenyl ethanol is commonly associated with rose-like aroma, whereas these esters are frequently described as fruity, apple-like, pineapple-like, or banana-like. Their enrichment suggests that 2L-FQ had a stronger potential for floral and ripe-fruit-related aroma expression [9].

Other compounds showing considerably higher concentrations in 2L-FQ included theaspirane, indole, 2-octanone, 2-pentylfuran, carvacrol, and the tentatively identified 3-methylcyclopentane-1,2-dione. Theaspirane decreased from 6.59 ± 0.30 μg g^−1^ in 2L-FQ to 2.65 ± 0.07 μg g^−1^ in 2L-HQ, while indole decreased from 6.92 ± 0.51 to 2.18 ± 0.18 μg g^−1^. The aspirane and indole have been associated with woody, fruity, and floral aroma characteristics in tea. However, sensory contribution cannot be determined from concentration alone because odor thresholds and matrix interactions strongly influence aroma activity. OAV or ROAV analysis would therefore be required before these compounds could be classified as key aroma-active compounds [9]. In contrast, 2L-HQ was distinguished from 2L-FQ by higher concentrations of selected alcohols, unsaturated aldehydes, esters, ketones, and furanic compounds. The largest increase was observed for 2,3-butanediol, which rose from 1.76 ± 0.21 μg g^−1^ in 2L-FQ to 8.43 ± 1.11 μg g^−1^ in 2L-HQ. The concentration of 1-penten-3-ol increased from 6.34 ± 0.38 to 12.55 ± 0.34 μg g^−1^, while dihydrolinalool increased from 3.19 ± 0.21 to 4.73 ± 0.04 μg g^−1^. These compounds may contribute sweet, fruity, green, floral, and citrus-like notes to the volatile profile of 2L-HQ [9,37].

The unsaturated aldehydes (E, E)-2,4-heptadienal and (E, E)-2,4-nonadienal increased from 3.58 ± 0.06 and 5.21 ± 0.24 μg g^−1^ in 2L-FQ to 4.90 ± 0.53 and 6.93 ± 0.45 μg g^−1^ in 2L-HQ, respectively. In addition, (Z)-3-hexenyl isopentanoate increased from 2.21 ± 0.13 to 4.22 ± 0.48 μg g^−1^, while bornyl acetate increased from 17.26 ± 1.55 to 20.71 ± 2.38 μg g^−1^. These compounds, including lipid-derived unsaturated aldehydes and selected esters, are generally associated with fresh, fatty, green, herbal, and fruity aroma notes. These lipid-derived aldehydes and ester volatiles are generally associated with fresh, fatty, green, herbal, and fruity aroma notes. Lipid oxidation can generate aldehydes, which may subsequently be converted into alcohols and esters through downstream enzymatic reactions [35].

The ketone 4-methylacetophenone increased from 0.72 ± 0.01 μg g^−1^ in 2L-FQ to 1.87 ± 0.06 μg g^−1^ in 2L-HQ, while 4-hydroxy-5-ethyl-2-methyl-3(2H)-furanone increased from 9.69 ± 0.34 to 11.26 ± 0.17 μg g^−1^. These compounds are associated with sweet, fruity, nutty, caramel-like, or candy-like odor descriptors. The 2-octanol-equivalent of these compounds suggests a stronger sweet and cooked-fruit-like volatile profile in 2L-HQ, although their actual sensory contribution should be verified by odor-threshold-based analysis [9]. The differences in lipid-derived volatile compounds were partly consistent with the LOX activity results. LOX activity increased from 720.00 ± 52.92 U g^−1^ FW in 2L-FQ to 1706.67 ± 94.52 U g^−1^ FW in 2L-HQ, representing an increase of approximately 137%. LOX catalyzes the oxygenation of linoleic and α-linolenic acids, after which hydroperoxide lyase generates C6 and related aldehydes. These aldehydes may subsequently be reduced to alcohols or converted into acids and esters. The higher LOX activity of 2L-HQ was therefore consistent with its higher concentrations of 1-penten-3-ol, (E, E)-2,4-heptadienal, (E, E)-2,4-nonadienal, and (Z)-3-hexenyl isopentanoate [35].

However, several other lipid-derived volatiles, including n-nonanal, (Z)-6-nonenal, 2-pentylfuran, methyl octanoate, and butyl butanoate, were more abundant in 2L-FQ despite its lower LOX activity. This apparent inconsistency indicates that LOX activity alone cannot determine the final volatile composition. The downstream distribution of lipid-derived intermediates is also influenced by hydroperoxide lyase, alcohol dehydrogenase, aldehyde dehydrogenase, alcohol acyltransferase, precursor availability, oxygen conditions, and tissue disruption. Previous research on oolong tea has shown that changes in oxygen availability can redirect the LOX–HPL pathway among aldehydes, alcohols, acids, and esters. Thus, 2L-HQ may have had greater upstream lipid-oxidation capacity, whereas the two origins differed in downstream metabolic partitioning [35].

By comparison, β-glucosidase activity did not differ significantly between 2L-FQ and 2L-HQ. β-Glucosidase can release free aroma compounds from glycosidically bound precursors, including linalool-related terpenoids, benzyl alcohol, phenylethanol, and methyl salicylate. However, because the β-glucosidase activities of the two groups were similar, the regional differences in dihydrolinalool and aromatic alcohols were unlikely to be explained by enzyme activity alone. Differences in glycoside precursor abundance, substrate accessibility, other glycosidases, and de novo terpenoid synthesis may also have contributed [37,38]. Although the two sampling sites had similar altitudes, the 2L-HQ cultivation site had higher mean temperature, relative humidity, precipitation, and soil wetness, together with lower solar radiation than the 2L-FQ cultivation site. These environmental differences may have influenced volatile formation by regulating lipid metabolism, precursor synthesis, leaf water status, redox balance, and enzyme activities. Tea studies have shown that temperature, light, rainfall, and soil-related factors can substantially alter the accumulation of alcohols, aldehydes, esters, terpenoids, phenylethanol, and other quality-related metabolites in fresh leaves [24,34]. These explanations are hypotheses consistent with previous studies rather than causal effects demonstrated by the present two-site design.

The warmer, more humid conditions at the 2L-HQ cultivation site may have contributed to greater tissue hydration in 2L-HQ, whereas the substantially higher LOX, PPO, and POD activities indicated enhanced enzymatic capacity. The increased concentrations of 1-penten-3-ol, dihydrolinalool, 2,3-butanediol, and unsaturated 2,4-dienals may therefore reflect enhanced lipid-related and stress-responsive metabolism under the 2L-HQ cultivation site conditions. In contrast, the relatively cooler and brighter 2L-FQ cultivation site environment may have promoted a different allocation of carbon and aroma precursors toward aromatic aldehydes, β-phenyl ethanol, fruity esters, indole, and theaspirane [24,34].

Overall, GC–IMS showed that Qilan fresh leaves from Fujian and Hainan shared a broadly similar volatile background but differed markedly in the relative abundance of several key compounds. This indicates that the growing region mainly reshaped the quantitative distribution of volatile compounds rather than producing completely distinct volatile profiles. These origin-related differences may influence not only the pre-existing aroma composition of fresh leaves but also their enzymatic potential for aroma formation and phenolic transformation during subsequent tea processing.

### 3.5. Untargeted Metabolomics Reveals Global Metabolic Differences

Untargeted metabolomic profiling was performed to characterize the overall metabolic differences between 2L-FQ and 2L-HQ. The differentially abundant features were putatively annotated based on MS/MS spectral information and database matching. After peak extraction, alignment, signal correction, annotation filtering, and integration of the positive- and negative-ion datasets, 4359 metabolic features were retained, including 2871 features detected in positive-ion mode and 1488 in negative-ion mode. As shown in Figure 4E, among these features, 4356 were shared by 2L-FQ and 2L-HQ, whereas only two were uniquely detected in 2L-HQ and one in 2L-FQ. The extensive overlap indicates that the two origins possessed essentially the same broad metabolite inventory. Their differentiation was therefore driven primarily by changes in the relative abundance of shared metabolites rather than by the appearance or disappearance of numerous origin-specific compounds. Annotation, relative abundance, and statistical information are provided in Table S2.

As shown in Figure 4D, the annotated metabolites covered a broad range of chemical classes. Organic acids and their derivatives represented the largest category, accounting for 26.21% of the class-assigned metabolites, followed by flavonoids (13.13%), benzenoids (12.55%), lipids and lipid-like molecules (7.32%), amino acids and their derivatives (3.72%), nucleotides and their derivatives (3.72%), and phenylpropanoids and polyketides (3.60%). These chemical classes contain metabolites involved in tea taste, antioxidant capacity, aroma-precursor formation, carbon and nitrogen metabolism, and environmental adaptation. However, the proportions in Figure 4C refer only to metabolites that could be assigned to a chemical class and should not be interpreted as the composition of all 4359 detected features. Untargeted metabolomics provides broad chemical coverage, but metabolite annotation remains constrained by spectral coverage, isomerism, and the availability of authentic standards [12].

As shown in Figure 4A, the PCA score plot showed clear separation between 2L-FQ and 2L-HQ. PC1 explained 53.70% of the total variance, whereas PC2 explained 13.64%; together, the two components accounted for 67.34% of the variation in the dataset. Samples from the same origin remained within the same region of the score space, while the two origins were separated mainly along PC1. This pattern indicates that origin-associated variation exceeded the variation among biological replicates and affected the overall metabolic state of the fresh leaves rather than a small subset of isolated compounds.

As shown in Figure 4B, OPLS-DA further enhanced the discrimination between the two groups. The predictive component explained 55.6% of the variation, while the first orthogonal component accounted for 11.9%. Separation along the predictive axis indicates that a substantial proportion of the metabolomic variation was associated with sample origin. Model validity was further evaluated using 200 random permutations (Figure 4C). The original model yielded R^2^X = 0.674 and Q^2^ = 0.965, and none of the permuted models exceeded the original model for either R^2^Y or Q^2^ (0/200 for both; empirical *p* < 0.005), indicating that the observed separation was unlikely to result from random class assignment. Nevertheless, OPLS-DA is a supervised method and can produce apparently strong separation when the number of variables is large relative to the number of biological replicates. Because only three biological replicates were available for each origin, the OPLS-DA results should therefore be interpreted as supportive evidence of origin-associated metabolic differentiation rather than as stand-alone proof of model robustness [39].

The volcano plot demonstrated extensive bidirectional changes in metabolite abundance between 2L-HQ and 2L-FQ (Figure 4F). Using the revised screening criteria of VIP > 1, FDR-adjusted *p* < 0.05, and |log_2_FC| > 1, 793 differential metabolic features were retained. Among these, 420 features showed higher abundance and 373 showed lower abundance in 2L-HQ relative to 2L-FQ. The higher number of upregulated features indicates that the leaves grown at the 2L-HQ cultivation site accumulated a broader set of metabolites at higher relative abundances. This observation is consistent with the higher contents of tea polyphenols, non-gallated catechins, free amino acids, and soluble polysaccharides reported for 2L-HQ in Section 3.2. It does not, however, indicate that overall metabolism was universally enhanced, because a substantial set of metabolites was preferentially accumulated in 2L-FQ.

As shown in Figure 4G, the VIP ranking identified the features contributing most strongly to group discrimination. Among the biologically plausible putative annotations were L-phenylalanine, indole-3-carboxaldehyde, 3-indoleacrylic acid, a linalool-related feature annotated as 3,7-dimethyl-1,6-octadien-3-ol, and methyl 7-epi-12-hydroxyjasmonate glucoside. These putatively annotated features provisionally connect the regional differentiation with aromatic amino acid metabolism, indole-related metabolism, terpenoid metabolism, and oxylipin or jasmonate-associated responses. Their biological interpretation should nevertheless remain tentative until their identities are supported by diagnostic MS/MS fragments, retention behaviour, or authentic standards. In accordance with accepted reporting frameworks for high-resolution mass spectrometry, database-derived names should not be presented as unequivocally identified metabolites when structural confirmation is unavailable [12,40].

Hierarchical clustering of the differential metabolic features provided an additional view of the abundance patterns (Figure 4H). The three biological replicates from each origin clustered together, whereas 2L-HQ and 2L-FQ formed separate sample branches. The heatmap further revealed reciprocal feature modules, with groups of features showing higher standardized relative abundance in 2L-HQ and lower abundance in 2L-FQ, and vice versa. These coordinated patterns are consistent with the PCA, OPLS-DA, and volcano-plot results and further indicate that regional differentiation mainly reflected quantitative shifts in shared metabolic features. Because the heatmap values are row-wise standardized Z-scores, the colors represent relative abundance patterns within each feature rather than absolute metabolite concentrations; the class annotation likewise reflects database-assigned chemical categories and does not imply confirmed structural identification.

The untargeted metabolomic separation was consistent with the environmental and biochemical differences described in Section 3.1 and Section 3.2. The 2L-HQ cultivation site was warmer and wetter, with higher relative humidity, precipitation, and soil wetness but lower solar radiation than the 2L-FQ cultivation site. These environmental conditions provide a plausible context for the phenotypic and biochemical profile of 2L-HQ, which was characterized by larger and more hydrated leaves, higher pigment concentrations, greater accumulation of selected quality-related constituents, and elevated LOX, PPO, and POD activities. Temperature and light regulate carbon allocation, amino acid metabolism, phenylpropanoid biosynthesis, and other quality-related metabolic processes in tea leaves. Field studies further indicate that moisture, soil properties, radiation, and temperature act jointly in determining fresh-leaf composition [24,34]. The separation observed in Figure 4, therefore, supports an integrated regional metabolic phenotype rather than the isolated effect of a single environmental variable.

Overall, untargeted metabolomics revealed pronounced quantitative reprogramming of the Qilan fresh-leaf metabolome. Although 99.93% of the detected features were shared between the two origins, their relative abundances produced clear separation in both PCA and OPLS-DA. The predominance of higher-abundance features in 2L-HQ paralleled the higher levels of several quality-related constituents measured in the targeted analyses. The substantial number of metabolites enriched in 2L-FQ indicates selective metabolic differentiation rather than a uniform reduction in metabolite abundance. These global results provide the basis for the subsequent identification of credible differential metabolites and the interpretation of their associated metabolic pathways.

### 3.6. Enrichment of Differential Metabolic Pathways

KEGG enrichment analysis was performed to identify the metabolic processes associated with the differences between 2L-FQ and 2L-HQ. As shown in Figure 5, the differential metabolic features were primarily associated with carbohydrate metabolism, amino acid metabolism, flavonoid-related biosynthesis, redox regulation, and cofactor metabolism. Starch and sucrose metabolism showed the highest enrichment factor, followed by lipoic acid metabolism, phosphonate and phosphinate metabolism, galactose metabolism, and pyruvate metabolism. However, pathway enrichment reflects the over-representation of differential features and does not independently indicate whether an entire pathway was activated or inhibited.

Carbohydrate-related pathways included starch and sucrose metabolism, galactose metabolism, glycolysis/gluconeogenesis, pyruvate metabolism, butanoate metabolism, and carbon fixation by the Calvin cycle. These pathways indicate that regional cultivation was associated with differences in carbon allocation among soluble carbohydrates, respiratory intermediates, and secondary metabolite precursors. This finding was consistent with the higher soluble-polysaccharide content of 2L-HQ. Previous studies have shown that temperature, light, nutrient supply, and other environmental factors can jointly regulate the allocation of carbohydrates, organic acids, amino acids, and flavonoids in tea plants [24,34,41,42].

Tyrosine metabolism, valine, leucine, and isoleucine biosynthesis, vitamin B6 metabolism, and cofactor biosynthesis were also represented among the enriched pathways. These pathways are closely associated with amino acid interconversion and carbon–nitrogen allocation. Their enrichment was broadly consistent with the higher free-amino acid content of 2L-HQ and suggests that the regional difference involved not only the total amino acid pool but also the distribution of specific amino acid-related metabolites. Carbon and nitrogen metabolism in mature tea leaves and roots can strongly influence amino acid and flavonoid accumulation in young shoots [41].

Isoflavonoid and anthocyanin biosynthesis further indicated changes in flavonoid-related secondary metabolism. This result agreed with the higher tea polyphenol content and the selective accumulation of C, EC, EGC, and GC in 2L-HQ. Temperature, light intensity, and nutrient availability have been reported to regulate catechin and flavonoid homeostasis in tea leaves [33,34,42]. Accordingly, these results support origin-associated alterations in flavonoid metabolism but do not independently verify the enhanced biosynthesis or accumulation of canonical isoflavonoids.

The enrichment of lipoic acid metabolism, ascorbate and aldarate metabolism, and vitamin B6 metabolism further indicated origin-associated differences in redox- and cofactor-related metabolic processes. These pathway-level differences were broadly associated with the higher DPPH, ABTS, and FRAP values observed in 2L-HQ [32]. Separately, the elevated PPO and POD activities indicated greater enzymatic phenolic-oxidation potential [36]. However, pathway enrichment alone does not indicate the direction of pathway regulation. KEGG enrichment analysis was used to identify metabolic pathways statistically associated with the differentially annotated features. Enrichment indicates over-representation of these features within specific pathways and should not be interpreted as direct evidence of pathway activation, inhibition, or altered metabolic flux.

Overall, the pathway-enrichment results indicate that the differentiation between 2L-FQ and 2L-HQ involved coordinated changes in carbon metabolism, amino acid metabolism, flavonoid-related biosynthesis, and redox/cofactor metabolism. Together with the targeted compositional, enzyme activity, and GC–IMS results, these findings suggest that regional cultivation was associated with different allocations of metabolic resources toward taste-related constituents, antioxidant compounds, and aroma-related precursors.

### 3.7. Electronic Nose and Electronic Tongue Profiles

To evaluate whether the origin-associated differences observed in fresh leaves remained detectable after processing, the finished black teas produced from 2L-FQ and 2L-HQ leaves were analyzed using an electronic nose and an electronic tongue. The electronic nose radar plots showed broadly similar response patterns for the two teas, indicating that they retained a comparable overall aroma framework. However, differences were observed in the response intensities of individual sensors, particularly W1W, with the 2L-HQ tea showing a higher response. Because metal-oxide-semiconductor sensors respond cross-reactively to complex volatile mixtures, these differences represent changes in the overall volatile fingerprint rather than variations in a single compound [8].

The electronic nose results were broadly consistent with the GC–IMS profiles described. The corresponding 2L-FQ fresh leaves contained higher levels of several aromatic aldehydes, β-phenyl ethanol, indole, theaspirane, and fruity esters, whereas 2L-HQ was enriched in selected alcohols, unsaturated aldehydes, dihydrolinalool, and lipid-derived esters. As shown in Figure 6C, the simultaneous increase and decrease in different volatile compounds may explain why the radar profiles largely overlapped while the electronic nose PCA still differentiated the finished teas.

As shown in Figure 6B, the electronic tongue radar plot also showed differences between the two finished teas. Overall, 2L-HQ exhibited slightly higher fitted responses than 2L-FQ, suggesting a stronger combined electrochemical response of the tea infusion. This trend was consistent with the higher levels of tea polyphenols, caffeine, free amino acids, soluble polysaccharides, and non-gallated catechins detected in the corresponding 2L-HQ fresh leaves. These compounds contribute differently to bitterness, astringency, umami, freshness, and mouthfeel, although the overall taste profile depends on their relative proportions and interactions rather than on any single constituent [4]. Therefore, the radar plot should be interpreted as a comparative taste fingerprint rather than as a direct sensory-intensity profile.

As shown in Figure 6D, Electronic tongue PCA provided further evidence of differentiation between the finished teas. PC1 and PC2 explained 57.3% and 28.3% of the total variance, respectively, accounting for 85.6% of the cumulative variation. The 2L-FQ samples were distributed on the negative side of PC1, whereas the 2L-HQ samples were located on the positive side, demonstrating distinct multivariate taste-response patterns. The separation may also be related to the higher PPO and POD activities of 2L-HQ, which indicated greater potential for phenolic oxidation during rolling and fermentation. Similarly, its higher LOX activity may have influenced the formation of lipid-derived volatiles during processing [35,36].

Overall, the electronic sensory results indicate that the regional differences detected in finished teas showed distinguishable instrumental sensor-response patterns; composition, enzyme activity, volatile fingerprints, and metabolite profiles were partially retained or transformed during black tea manufacture. Although the two teas exhibited broadly similar overall sensor-response patterns, PCA clearly separated them, indicating differences in the relative intensities and combinations of aroma- and taste-related signals.

## 4. Conclusions

This study systematically characterized the region-associated differences in Qilan fresh tea leaves grown in Wuyishan City, Fujian Province, and Baisha Li Autonomous County, Hainan Province, under comparable altitude, cultivar, plant-age, and plucking-standard conditions. Compared with 2L-FQ, 2L-HQ developed under a warmer, wetter, and lower-radiation environment and exhibited larger and more hydrated leaves, darker coloration, and higher pigment contents. The Baisha-grown leaves also contained higher levels of tea polyphenols, soluble polysaccharides, caffeine, free amino acids, and several non-gallated catechins, together with greater in vitro antioxidant capacity. The pronounced increases in LOX, PPO, and POD activities further indicated stronger lipid- and phenolic-oxidation potential, whereas β-glucosidase activity remained unchanged. GC–IMS detected 61 volatile compounds, of which 46 differed significantly between the two origins. 2L-FQ was characterized by higher levels of several aromatic aldehydes, β-phenyl ethanol, and fruity esters, whereas 2L-HQ accumulated higher levels of selected alcohols, unsaturated aldehydes, furanic compounds, and lipid-derived esters. Untargeted metabolomics retained 4359 metabolic features, 4356 of which were shared by both groups. Nevertheless, 793 differential features produced clear metabolic separation and were mainly associated with carbohydrate, amino acid, flavonoid-related, lipid, redox, and cofactor metabolism. These findings indicate that regional differentiation of Qilan fresh leaves arose primarily from the quantitative redistribution of a largely shared chemical inventory rather than from the formation of numerous origin-specific compounds. The electronic nose and electronic tongue results further suggested distinguishable aroma- and taste-response fingerprints in the finished products. Electronic nose analysis of finished black tea and electronic tongue analysis of the corresponding infusions further differentiated the instrumental response profiles of the processed teas. Overall, the contrasting environments were associated with coordinated changes in leaf physiology, taste-related constituents, oxidative-enzyme capacity, volatile composition, and metabolic allocation. This provides a basis for further investigating region-adapted processing strategies for Qilan tea. However, because the study involved two cultivation sites and one sampling period, multi-season and multi-site investigations do not isolate individual environmental drivers, verify pathway activation, or establish superior sensory quality. Replication across sites and seasons, targeted chemical confirmation, controlled processing comparisons, and standardized sensory evaluation are needed to assess the generality and practical implications of these findings. Together with targeted validation of key metabolites and standardized sensory evaluation of finished teas, these are required to establish the environmental drivers and practical quality consequences of these differences.

## Figures and Tables

**Figure 1:**
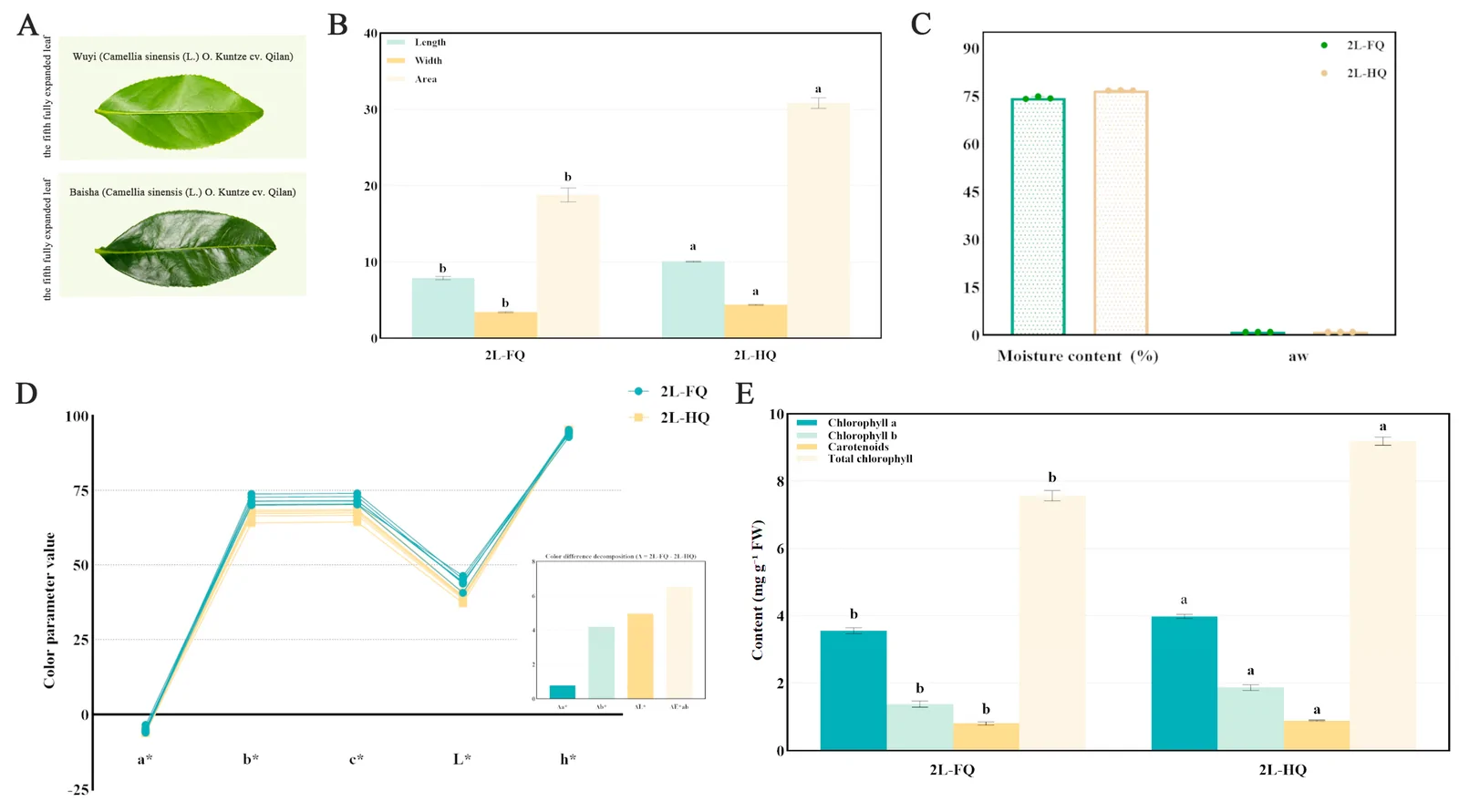
Phenotypic traits of fresh Qilan tea leaves from the 2L-FQ cultivation site, and 2L-HQ cultivation site (2L-HQ). (**A**) Representative fresh leaves. (**B**) Leaf length, leaf width, and estimated leaf area. (**C**) Moisture content and water activity. (**D**) Color parameters (L*, a*, b*, c*, and ΔE*ab). (**E**) Photosynthetic pigment contents, including chlorophyll a, chlorophyll b, total chlorophyll, and carotenoids. Data are presented as mean ± SD, n = 3 independent biological replicates; subsample measurements were averaged within each replicate. Different lowercase letters indicate significant differences between 2L-FQ and 2L-HQ at *p* < 0.05. FW, fresh weight.

**Figure 2:**
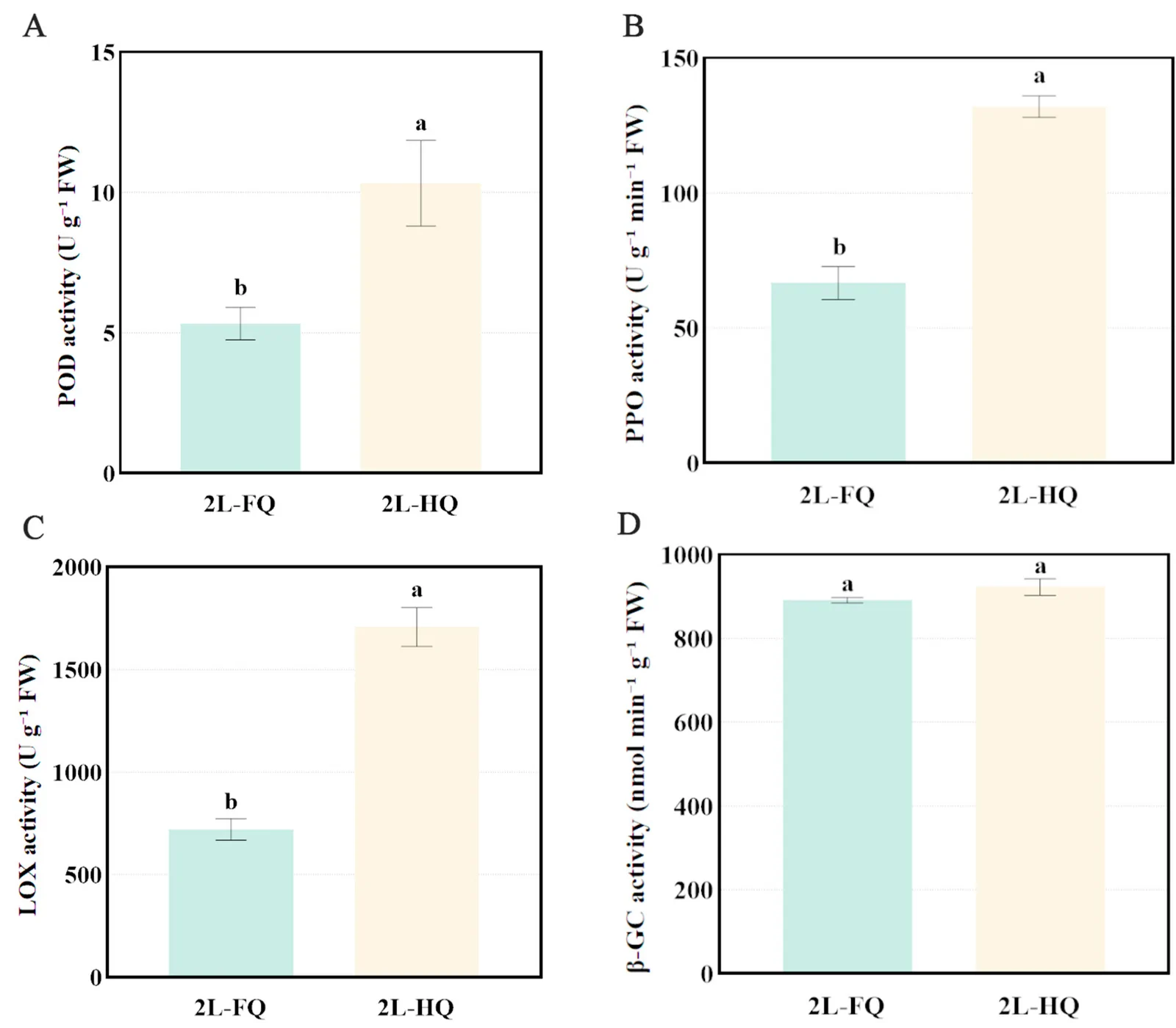
Enzyme activities of fresh Qilan tea leaves grown in the 2L-FQ and 2L-HQ cultivation sites, Hainan (2L-HQ). (**A**) Peroxidase (POD), (**B**) polyphenol oxidase (PPO), (**C**) lipoxygenase (LOX), and (**D**) β-glucosidase activities. Bars represent the mean ± SD (n = 3). Different lowercase letters indicate significant differences (*p* < 0.05). FW, fresh weight.

**Figure 3:**
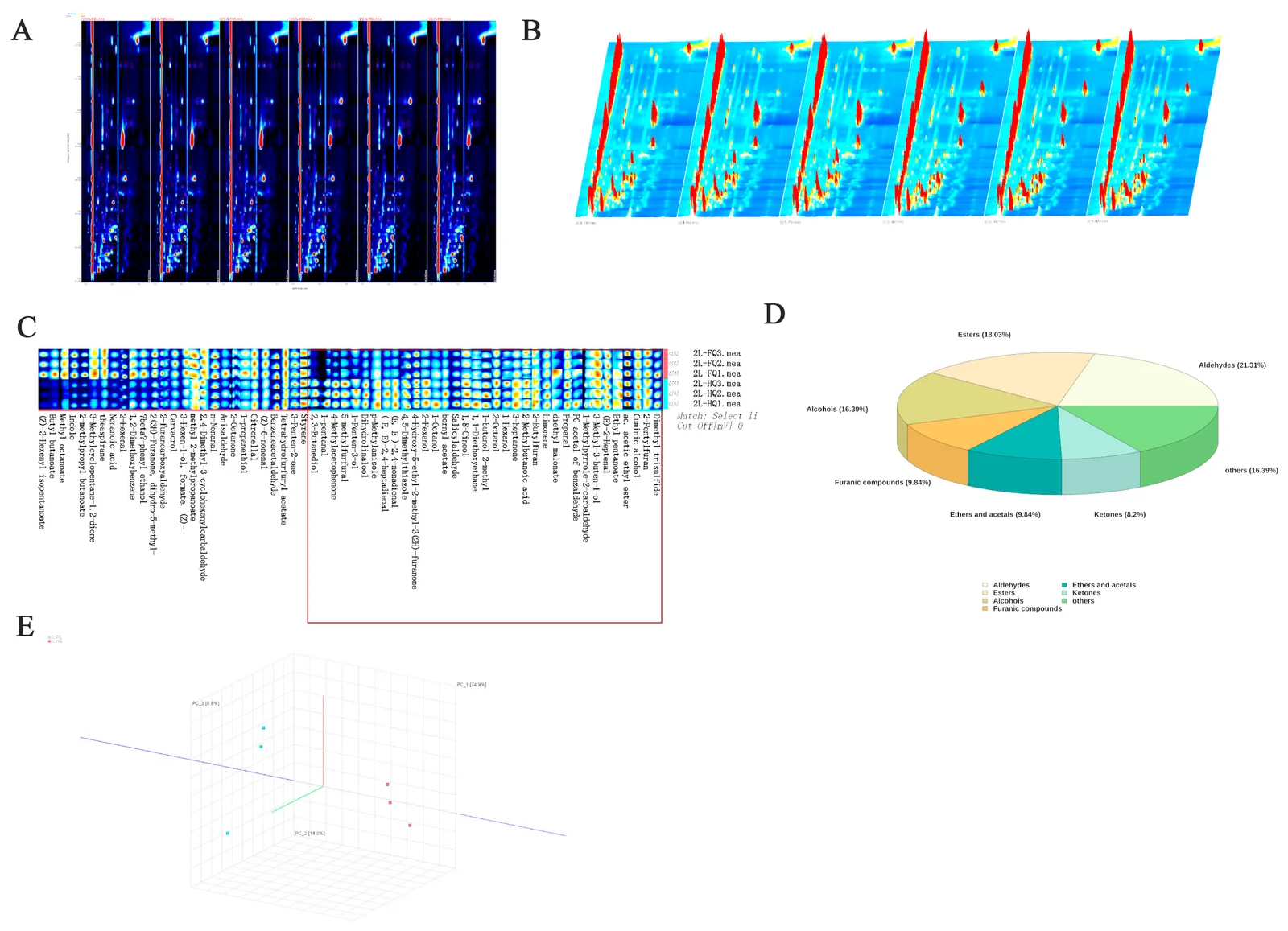
GC–IMS analysis of volatile compounds in fresh Qilan tea leaves grown in the 2L-FQ and the 2L-HQ cultivation sites. (**A**) GC–IMS topographic plots; (**B**) three-dimensional GC–IMS topographic plots; (**C**) gallery plot of volatile fingerprints; (**D**) distribution of the identified volatile compounds among chemical classes; and (**E**) three-dimensional PCA score plot. GC–IMS, gas chromatography–ion mobility spectrometry; PCA, principal component analysis.

**Figure 4:**
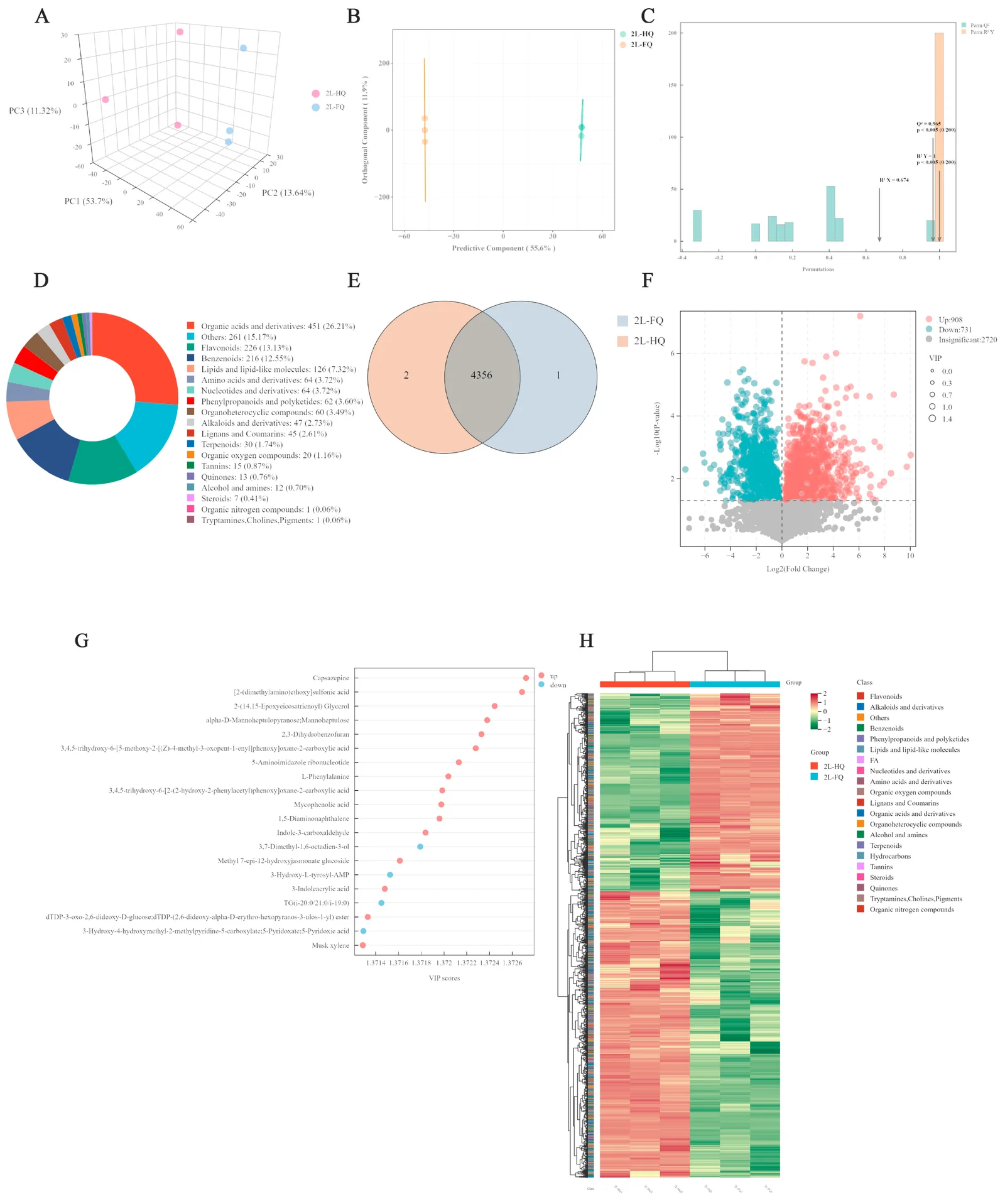
Untargeted metabolomic profiling of 2L-FQ and 2L-HQ fresh leaves. (**A**) PCA score plot. (**B**) OPLS-DA score plot. (**C**) OPLS-DA model validation by a 200-permutation test; the original model yielded R^2^X = 0.674 and Q^2^ = 0.965, with empirical *p* < 0.005 (0/200) for both R^2^Y and Q^2^. (**D**) Chemical classification of putatively annotated features. (**E**) Venn diagram showing shared and origin-specific detected metabolic features. (**F**) Volcano plot of differential metabolic features between 2L-FQ and 2L-HQ. (**G**) VIP-ranked plot of representative differentially abundant features with putative metabolite annotations. (**H**) Hierarchical clustering heatmap of differential metabolic features; colors indicate row-wise standardized relative abundances (Z-scores), and the side annotation indicates database-assigned chemical classes. Differential metabolic features were screened using VIP > 1, FDR-adjusted *p* < 0.05, and |log_2_FC| > 1.

**Figure 5:**
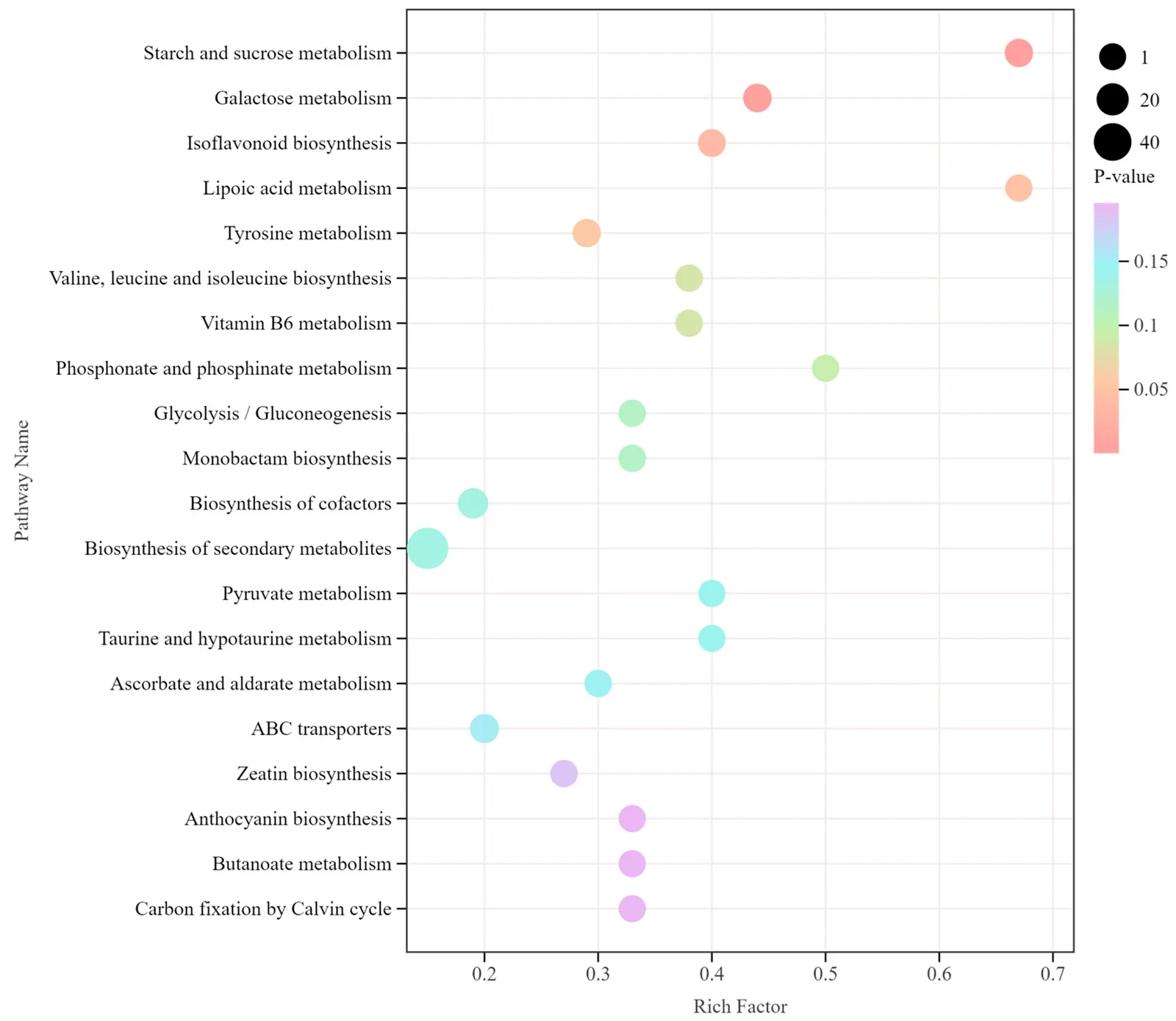
Pathway Enrichment of Differential Metabolic Features between 2L-FQ and 2L-HQ Fresh Leaves. Bubble size represents the number of differential metabolic features, and color represents the enrichment *p*-value.

**Figure 6:**
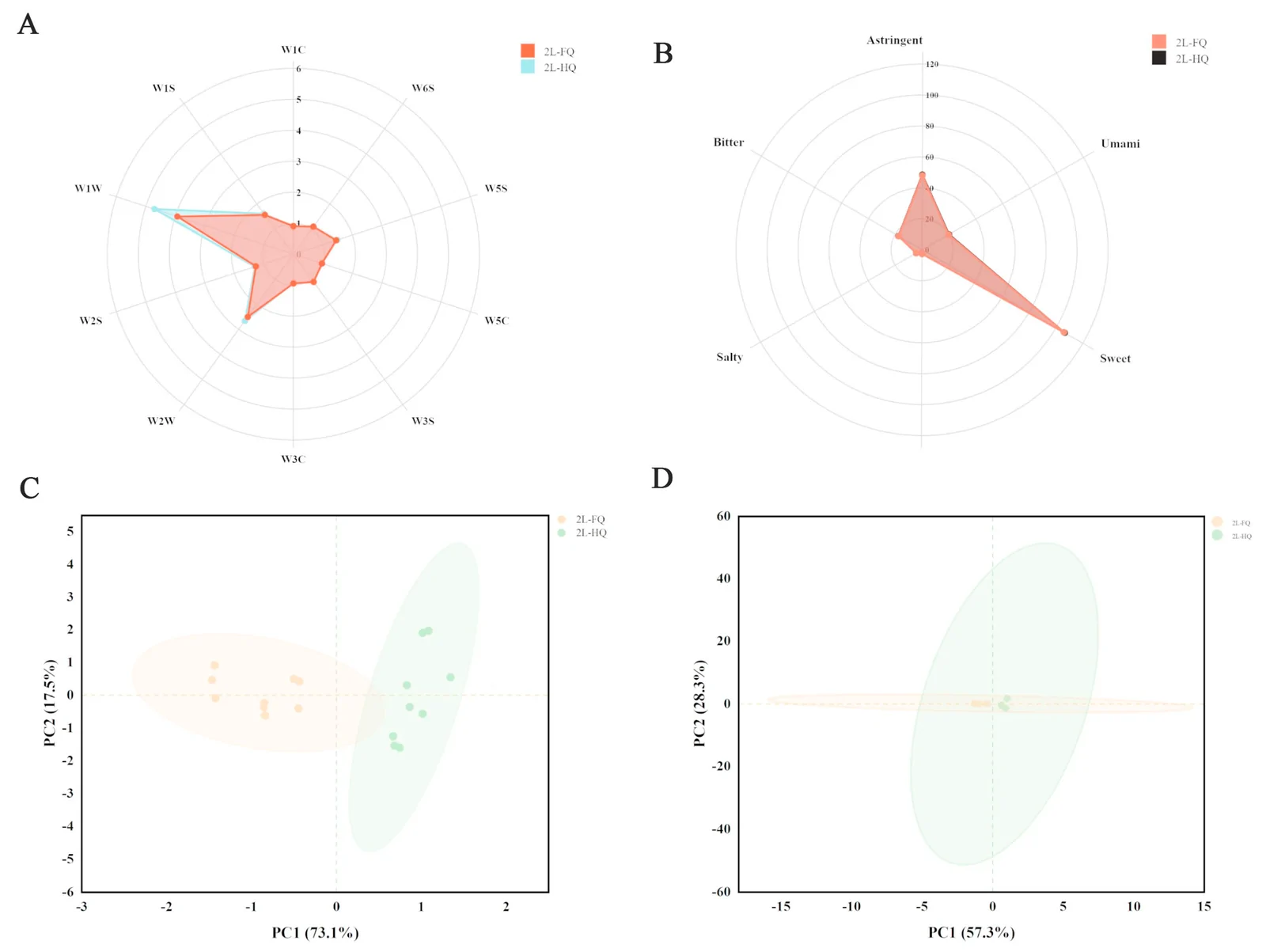
Electronic sensory profiles of finished black teas produced from 2L-FQ and 2L-HQ. (**A**) radar plot of electronic nose sensor responses; (**B**) radar plot of electronic tongue taste responses; (**C**) principal component analysis score plot of electronic nose sensor responses; (**D**) principal component analysis score plot based on electronic tongue response features. Each point in the PCA plots represents an independently prepared sample replicate (n = 3).

**Table 1:** Environmental Conditions of Qilan Fresh Tea Leaves from Wuyishan, Fujian, and Baisha, Hainan.

Parameter	Unit	Wuyishan, Fujian	Baisha, Hainan
Latitude	°N	27.93	19.92
Longitude	°E	118.03	109.58
Altitude	m	222	216
Monthly maximum temperature	°C	34.01	30.16
Monthly minimum temperature	°C	8.82	19.99
Relative humidity	%	80.34	85.86
Total precipitation	mm	133.62	308.20
Mean air temperature	°C	21.67 ± 5.71	26.10 ± 2.13
Mean daily maximum temperature	°C	26.30 ± 6.45	27.54± 2.52
Mean daily minimum temperature	°C	17.87± 5.33	24.92 ± 1.91
Solar radiation	MJ m^−2^ day^−1^	13.28 ± 4.64	11.44 ± 5.88
Wind speed at 2 m	m s^−1^	0.60 ± 0.19	4.22 ± 1.54
Surface soil wetness	/	0.78	0.84
Root-zone soil wetness	/	0.79	0.82
Profile soil wetness	/	0.86	0.83

Note: Environmental variables were summarized for October 2025. Values for mean air temperature, mean daily maximum temperature, mean daily minimum temperature, solar radiation, and wind speed are presented as mean ± SD. Surface soil wetness, root-zone soil wetness, and profile soil wetness are dimensionless indices.

**Table 2:** Major Quality-Related Components in Qilan Fresh Tea Leaves from Fujian and Hainan.

Compound	Unit	2L-FQ	2L-HQ
C	mg g^−1^ DW	6.29 ± 0.32 ^b^	8.44 ± 0.52 ^a^
EC	mg g^−1^ DW	4.38 ± 0.56 ^b^	10.71 ± 0.23 ^a^
CG	mg g^−1^ DW	0.56 ± 0.12 ^a^	0.40 ± 0.12 ^b^
ECG	mg g^−1^ DW	23.61 ± 1.40 ^a^	22.47 ± 1.42 ^a^
EGC	mg g^−1^ DW	13.22 ± 0.24 ^b^	17.63 ± 0.32 ^a^
GCG	mg g^−1^ DW	1.22 ± 0.02 ^a^	1.10 ± 0.15 ^a^
EGCG	mg g^−1^ DW	57.24 ± 3.35 ^a^	55.22 ± 4.12 ^a^
GC	mg g^−1^ DW	3.42 ± 0.41 ^b^	4.64 ± 0.32 ^a^
Caffeine	mg g^−1^ DW	9.26 ± 0.22 ^b^	10.32 ± 0.17 ^a^
Tea polyphenols	mg g^−1^ DW	196.42 ± 1.75 ^b^	219.67 ± 2.46 ^a^
soluble polysaccharides	mg g^−1^ DW	41.61 ± 1.79 ^b^	52.58 ± 0.98 ^a^
Free amino acids	μmol g^−1^ DW	29.23 ± 0.25 ^b^	32.34 ± 0.82 ^a^
DPPH	μmol TE g^−1^ DW	577.65 ± 6.35 ^b^	612.80 ± 7.83 ^a^
ABTS	μmol TE g^−1^ DW	684.35 ± 7.21 ^b^	717.62 ± 8.46 ^a^
FRAP	μmol TE g^−1^ DW	129.92 ± 1.15 ^b^	132.80 ± 0.95 ^a^

Note: Values are expressed as mean ± SD, n = 3. Different lowercase letters in the same row indicate significant differences between 2L-FQ and 2L-HQ at *p* < 0.05. DW, dry weight.

**Table 3:** Tentative assignments and semi-quantitative abundances of GC–IMS volatile signals.

No.	Compounds	CAS#	Formula	MW	RI	Rt (s)	Dt (RIP Relative)	Semi-Quantitative Content (μg 2-Octanol Equivalents g^−1^ FW)		Comment
								2L-FQ	2L-HQ	
Aldehydes										
1	Propanal	123-38-6	C_3_H_6_O	58.1	756.8	186,066	115,167	5.70 ± 0.28 ^a^	4.71 ± 0.08 ^b^	pungent; green, grassy
2	1-Pentanal	110-62-3	C_5_H_10_O	86.1	917.4	292,899	142,626	1.55 ± 0.03 ^a^	1.70 ± 0.11 ^a^	green grassy; faint banana; pungent
3	n-Nonanal	124-19-6	C_9_H_18_O	142.2	1113.2	522,063	151,499	9.60 ± 0.56 ^a^	5.72 ± 0.57 ^b^	rose; citrus; strong oily
4	(Z)-6-Nonenal	2277-19-2	C_9_H_16_O	140.2	1122.0	537,622	117,658	9.00 ± 0.54 ^a^	5.99 ± 0.42 ^b^	melon
5	2-Hexenal	505-57-7	C_6_H_10_O	102.2	777.8	197,481	127,731	19.01 ± 0.65 ^a^	7.90 ± 0.75 ^b^	sweet almonds; fruity; green leaves; apples; plums; vegetables
6	(E)-2-Heptenal	18829-55-5	C_7_H_12_O	112.2	931.8	305.04	124,418	3.30 ± 0.31 ^a^	3.53 ± 0.09 ^a^	spicy; green vegetables; fresh; fatty
7	(E, E)-2,4-Heptadienal	4313-03-5	C_7_H_10_O	110.2	1009.7	381,113	118,818	3.58 ± 0.06 ^b^	4.90 ± 0.53 ^a^	fatty; oily; aldehyde; vegetable; cinnamon
8	(E, E)-2,4-nonadienal	5910-87-2	C9H14O	138.2	1217.2	702,035	135,171	5.21 ± 0.24 ^b^	6.93 ± 0.45 ^a^	fruity
9	Citronellal	106-23-0	C_10_H_18_O	154.3	1134.4	560,518	132,932	4.15 ± 0.14 ^a^	2.66 ± 0.34 ^b^	lemon; lemongrass; rose
10	Salicylaldehyde	90-02-8	C_7_H_6_O_2_	122.1	1052.4	431,951	11,557	14.33 ± 0.75 ^a^	14.94 ± 0.44 ^a^	medical spicy cinnamon wintergreen cooling
11	Benzeneacetaldehyde	122-78-1	C_8_H_8_O	120.2	1024.9	398,441	15,524	19.51 ± 1.18 ^a^	14.54 ± 0.88 ^b^	hyacinth; sweet fruity; almond; cherry; clover honey; cocoa
12	Anisaldehyde	123-11-5	C_8_H_8_O_2_	136.1	1242.1	735,683	166,447	5.72 ± 0.48 ^a^	4.37 ± 0.18 ^b^	sweet; mimosa; floral fragrance; hawthorn
13	2,4-Dimethyl-3-cyclohexenylcarbaldehyde	68039-49-6	C_9_H_14_O	138.2	1160.0	610,911	117,487	2.64 ± 0.10 ^a^	1.60 ± 0.07 ^b^	fruity; grass
Alcohols										
1	Cuminic alcohol	536-60-7	C_10_H_14_O	150.2	1288.4	802,505	13,318	7.65 ± 0.33 ^a^	6.26 ± 0.13 ^b^	spicy; strong cumin; caraway; herbal
2	1-Hexanol	513-85-9	C_6_H_14_O	102.2	893.0	273,415	132,739	1.51 ± 0.14 ^a^	1.47 ± 0.06 ^a^	fresh; fruity; wine; sweet; green
3	2,3-Butanediol	137-32-6	C_4_H_10_O_2_	90.1	777.8	197,481	136,729	1.76 ± 0.21 ^b^	8.43 ± 1.11 ^a^	sweet; fruity; butter
4	1-Butanol, 2-methyl-	111-87-5	C_5_H_12_O	88.1	730.3	172,666	12,425	8.41 ± 0.82 ^a^	8.52 ± 0.77 ^a^	roast onion; fruity; floral; wine
5	1-Octanol	626-93-7	C_8_H_18_O	130.2	1052.9	432,568	148,183	3.73 ± 0.82 ^a^	4.03 ± 0.81 ^a^	citrus; sweet; herbs; waxy; rose; mushroom
6	2-Hexanol	616-25-1	C_6_H_14_O	102.2	777.8	197,481	127,731	11.55 ± 1.03 ^a^	12.37 ± 0.85 ^a^	fruity; fat; cauliflower
7	1-Penten-3-ol	78-69-3	C_5_H_10_O	86.1	654.6	139,413	132,994	6.34 ± 0.38 ^b^	12.55 ± 0.34 ^a^	ethereal; green; tropical fruity
8	Dihydrolinalool	763-32-6	C_10_H_22_O	158.3	1116.1	527,026	12,442	3.19 ± 0.21 ^b^	4.73 ± 0.04 ^a^	floral; Fruity; green; citrus
9	β-Phenyl ethanol	60-12-8	C_8_H_10_O	122.2	1134.0	559,781	164,826	19.28 ± 0.90 ^a^	7.91 ± 0.63 ^b^	rose
10	3-Methyl-3-buten-1-ol	736-32-6	C_5_H_10_O	86.1	736.4	175,68	128,244	1.94 ± 0.10 ^a^	1.56 ± 0.06 ^b^	sweet, fruity
Esters										
1	Methyl 2-methylpropanoate	547-63-7	C_5_H_10_O_2_	102.1	682.2	150,712	113,326	3.56 ± 0.24 ^a^	2.14 ± 0.16 ^b^	apple; pineapple
2	Ac. acetic ethyl ester	141-78-6	C_4_H_8_O_2_	88.1	617.5	125,546	11,146	3.27 ± 0.17 ^a^	2.65 ± 0.11 ^b^	fresh; fruity; sweet; grassy
3	Ethyl pentanoate	539-82-2	C_7_H_14_O_2_	130.2	1154.1	598,989	127,221	6.64 ± 1.34 ^a^	5.71 ± 0.81 ^a^	apple; pineapple; green
4	Diethyl malonate	105-53-3	C_7_H_12_O_4_	160.2	1074.2	460,471	126,925	16.84 ± 1.72 ^a^	18.00 ± 1.36 ^a^	sweet ester
5	Methyl octanoate	111-11-5	C_9_H_18_O_2_	158.2	1128.4	549,337	143,779	8.17 ± 0.19 ^a^	3.59 ± 0.34 ^b^	fruity; orange; wine; wax
6	Tetrahydrofurfuryl acetate	637-64-9	C_7_H_12_O_3_	144.2	1094.2	489,703	117,487	6.04 ± 0.26 ^a^	4.35 ± 0.33 ^b^	fruity; liquor
7	3-Hexen-1-ol, formate, (Z)-	33467-73-1	C_7_H_12_O_2_	128.2	923.4	297.91	11,734	10.35 ± 0.59 ^a^	6.05 ± 0.16 ^b^	green
8	Butyl butanoate	109-21-7	C_8_H_16_O_2_	144.2	1002.2	372,774	135,184	9.36 ± 1.08 ^a^	1.76 ± 0.05 ^b^	fruity; banana; pineapple; green cherry; tropical fruit mature and juicy fruity aroma
9	2-Methylpropyl butanoate	539-90-2	C_8_H_16_O_2_	144.2	958.8	329,281	134,741	12.07 ± 1.28 ^a^	4.31 ± 0.70 ^b^	apple; pineapple
10	(Z)-3-Hexenyl isopentanoate	35154-45-1	C_11_H_20_O_2_	184.3	1239.5	732,118	146,244	2.21 ± 0.13 ^b^	4.22 ± 0.48 ^a^	fruity
11	Bornyl acetate	5655-61-8	C_12_H_20_O_2_	196.3	1240.9	734,104	125,158	17.258 ± 1.55 ^b^	20.708 ± 2.38 ^a^	herbal, pine leaf
Ketones										
1	3-Penten-2-one	625-33-2	C_5_H_8_O	84.1	749.1	182,095	132,654	6.63 ± 0.49 ^a^	5.03 ± 0.05 ^b^	fruity; turns spicy during storage
2	2-Octanone	111-13-7	C_8_H_16_O	128.2	1292.8	809,121	175,885	15.87 ± 0.39 ^a^	8.11 ± 0.30 ^b^	mouldy; ketone; milk; cheese; mushroom
3	3-Heptanone	106-35-4	C_7_H_14_O	114.2	1167.6	626,782	160,497	18.57 ± 1.04 ^a^	18.36 ± 0.42 ^a^	fruity; Grass; Oil
4	4-Methylacetophenone	122-00-9	C_9_H_10_O	134.2	1168.2	628,022	168,659	0.72 ± 0.01 ^b^	1.87 ± 0.06 ^a^	sweet; fruity; nuts; vanilla
5	3-Methylcyclopentane-1,2-dione	765-70-8	C_6_H_8_O_2_	112.1	1093.8	488.99	150,373	27.57 ± 1.53 ^a^	0.90 ± 0.18 ^b^	sweet caramel; coffee; woody
Furanic compounds										
1	5-Methylfurfural	620-02-0	C_6_H_6_O_2_	110.1	979.8	349,349	140,718	1.63 ± 0.14 ^a^	1.99 ± 0.20 ^a^	spices; caramel wood
2	2-Butylfuran	4466-24-4	C_8_H_12_O	124.2	905.9	283,587	118,849	2.36 ± 0.08 ^a^	2.15 ± 0.10 ^a^	mild fruity; alcoholic; sweet and spicy
3	2-Pentylfuran	3777-69-3	C_9_H_14_O	138.2	979.7	349,245	125,451	25.83 ± 1.48 ^a^	21.17 ± 0.98 ^b^	bean; fruity; earthy; green; vegetable
4	2(3H)-Furanone, dihydro-5-methyl-	108-29-2	C_5_H_8_O_2_	100.1	957.3	327,896	110,262	6.24 ± 0.29 ^a^	3.26 ± 0.27 ^b^	herbal; sweet; tobacco; woody
5	2-Furancarboxaldehyde	98-01-1	C_5_H_4_O_2_	96.1	856.4	246,575	132,677	2.28 ± 0.24 ^a^	1.19 ± 0.06 ^b^	sweet; woody; almond; bready
6	4-Hydroxy-5-ethyl-2-methyl-3(2H)-furanone	27538-09-6	C_7_H_10_O_3_	142.2	1168.9	629,448	134,741	9.69 ± 0.34 ^b^	11.26 ± 0.17 ^a^	candy; Caramel; Cotton Candy
Esthers and acetals										
1	1,8-Cineol	470-82-6	C_10_H_18_O	154.3	1020.5	393.31	127,657	15.47 ± 0.21 ^a^	14.68 ± 0.40 ^b^	camphor; refreshing herbal
2	1,1-Diethoxyethane	105-57-7	C_6_H_14_O_2_	118.2	708.2	162,233	111,152	14.11 ± 1.44 ^a^	14.58 ± 1.42 ^a^	floral; fruity
3	p-Methylanisole	104-93-8	C_8_H_10_O	122.2	1039.8	416,266	110,999	2.93 ± 0.17 ^b^	4.16 ± 0.29 ^a^	nut
4	1,2-Dimethoxybenzene	91-16-7	C_8_H_10_O_2_	138.2	1135.8	563,141	157,009	4.08 ± 0.63 ^a^	1.40 ± 0.04 ^b^	sweet cream; vanilla; phenols; aldehydes; musty
5	Theaspirane	36431-72-8	C_13_H_22_O	194.3	1272.7	779,175	14,005	6.59 ± 0.30 a	2.65 ± 0.07 ^b^	black tea, fruity, woody
6	PG acetal of benzaldehyde	2568-25-4	C1_0_H_12_O_2_	164.2	1279.1	788,669	115,763	4.54 ± 1.35 ^a^	3.668 ± 1.07 ^b^	almond
Heterocyclic compounds										
1	4,5-Dimethylthiazole	3581-91-7	C_5_H_7_NS	113.2	935.9	308,652	148,443	2.34 ± 0.19 ^b^	2.95 ± 0.06 ^a^	green; Nut; Roast
2	1-Methylpyrrole-2-carbaldehyde	1192-58-1	C_6_H_7_NO	109.1	1023.8	397,222	149,451	3.35 ± 0.17 ^a^	3.77 ± 0.34 ^a^	nut
3	Indole	120-72-9	C_8_H_7_N	117.2	1242.1	735,683	114,538	6.92 ± 0.51 ^a^	2.18 ± 0.18 ^b^	floral; fecal odor
Acids										
1	2-Methylbutanoic acid	116-53-0	C_5_H_10_O_2_	102.1	876.6	261,007	121,194	20.50 ± 2.76 ^a^	21.46 ± 0.94 ^a^	pungent and spicy cheese; fruity
2	Nonanoic acid	112-05-0	C_9_H_18_O_2_	158.2	1240.9	733,984	156,253	14.34 ± 1.32 ^a^	6.89 ± 0.39 ^b^	fatty; coconut; cheese
Sulfur-containing compounds										
1	1-Propanethiol	107-03-9	C_3_H_8_S	76.2	870.5	256,558	138,148	3.24 ± 0.20 ^a^	2.11 ± 0.18 ^b^	lemon; sweet; orange; pine oil
2	Dimethyl trisulfide	3658-80-8	C_2_H_6_S_3_	126.3	935.9	308,605	133,267	4.12 ± 0.58 ^a^	4.05 ± 0.34 ^a^	special aromatic aroma; organic; sweet; pungent
Hydrocarbons										
1	Limonene	138-86-3	C_10_H_16_	136.2	1030.0	404,528	1277	5.13 ± 0.23 ^a^	4.49 ± 0.03 ^b^	lemon; sweet; orange; pine oil
2	Styrene	100-42-5	C_8_H_8_	104.2	869.4	255,775	142,155	4.28 ± 0.21 ^a^	3.05 ± 0.10 ^b^	special aromatic aroma; organic; sweet; pungent
Phenols										
1	Carvacrol	499-75-2	C_10_H_14_O	150.2	1297.5	817,671	12,639	8.15 ± 0.79 ^a^	4.54 ± 0.07 ^b^	spice, camphor, thymol

Note: Semi-quantitative contents are expressed as μg 2-octanol equivalents g^−1^ FW and are presented as mean ± SD (n = 3). Different lowercase superscript letters within the same row indicate significant differences between 2L-FQ and 2L-HQ (*p* < 0.05). 2-Octanol was used as the internal standard. Odor characteristics are putative descriptors obtained from the GC–IMS library and do not directly indicate aroma contribution. RI, retention index; Rt, retention time; Dt, drift time relative to the reaction ion peak; FW, fresh weight.

## Data Availability

The original contributions presented in the study are included in the article and its Supplementary Materials; further inquiries can be directed to the corresponding author.

## Supplementary Materials

The following supporting information can be downloaded at: https://www.mdpi.com/article/10.3390/foods15193478/s1, Table S1: GC–IMS compound assignments and peak-volume data of 2L-FQ and 2L-HQ samples; Table S2: Annotation, relative abundance, and statistical information of metabolites detected in 2L-HQ and 2L-FQ samples by untargeted metabolomics.
