# Supplementary material for: Untargeted Metabolomics and GC–IMS Reveal Quality-Related Differences in Qilan Fresh Tea Leaves Grown in Wuyi and Baisha
Source: Foods. 2026 Sep 29;15(19):3478. doi: 10.3390/foods15193478 (PMC13664276; doi:10.3390/foods15193478)
Supplement: Supplementary file 1 [file foods-15-03478-s001.zip › foods-4513642-supplementary.pdf]

Supplementary Materials: Untargeted Metabolomics and GC–IMS Reveal Quality-Related Differences in Qilan Fresh Tea Leaves Grown in Wuyi and Baisha

Longbin Zhong[1,2], Chanyi Xie[1], Mingchang Yang[1], Yibo Hu[1], Tianhang Huan[1], Liang Xu[3], Zhigang Guang[3], Xia Xie[2], Yiyi Yin[2], Rongrong He[1*], Haiming Chen[1*]

Table S1. AGC–IMS compound assignments and peak-volume data of 2L-FQ and 2L-HQ samples.

	2L-FQ1	2L-FQ2	2L-FQ3	2L-HQ1	2L-HQ2	2L-HQ3
2-Octanol	8911351	8337048	8644187	11153504	11110426	10933904
(Z)-3-Hexenyl isopentanoate	7508894	7856929	7443987	17718959	17425975	20890277
Nonanoic acid	47330356	52741504	48118405	29425087	29956668	32096002
Indole	22786892	23179204	25605618	9140553	9342188	10458469
Anisaldehyde	19618544	20907982	18577292	18958755	20369855	18727135
4-Hydroxy-5-ethyl-2-methyl-3(2H)-furanone	34727998	33326497	32248826	50306400	50750146	48493479
4-Methylaceto-phenone	2526804	2432558	2462835	8039163	8333748	8393778
3-heptanone	62264905	62392440	67559406	81805206	79762066	82191519
?beta?-phenyl ethanol	65017584	66262873	68213696	35111673	32475726	37468417
1,2-Dimethox-ybenzene	12417593	13385604	16389347	6452638	6087533	6072685
2-furancarboxy-aldehyde	7271547	7566621	8707899	5077542	5191149	5522205
Dihydrolinal-ool	11158172	11421191	10438881	20949568	20992876	20897886
Benzeneacetal-dehyde	64668154	67535199	69596997	61936836	63039515	67960763
Salicylaldehyde	48625392	50471167	49164411	64573443	66739777	67092737
2(3H)-Furanone, di-hydro-5-me-thyl-	21195613	20845364	22568867	13731329	13917555	15596267

2-methylpropyl butanoate	38033452	41211206	45559004	17027761	17745985	22358740
Butyl butanoate	28904346	33068045	34717782	8055704	7594985	7727061
2-Pentylfuran	92886510	90628735	83835910	89851935	94667175	96578960
(E)-2-Heptenal	11210838	12213612	10725709	15469108	16109995	15236511
3-Hexen-1-ol, formate, (Z)-	34543284	35096478	37466954	26698734	26373563	27288660
2-Hexenal	65340342	63578364	67867515	33959539	38905396	32097903
Tetrahydro-furfuryl acetate	21073523	21119314	20304717	18773110	18267051	20638442
1-Penten-3-ol	21183252	22377834	22006602	54374713	57168642	55048926
1-Methylpyr-role-2-carbalde-hyde	11301532	11676805	11686627	16402593	15463518	18114884
2-Octanone	55109182	53077573	56127289	37638748	35735454	34308242
Methyl octano-ate	29896373	26752818	28030884	16416362	17257404	14049478
diethyl malo-nate	52961754	59831264	61280885	73560715	85033834	80344704
2-Hexanol	37066766	41345795	40943221	58089842	50670242	55486316
2-Butylfuran	8328965	7629105	8472732	9093943	9768751	9733638
Ethyl pentano-ate	19852313	20720393	28164517	24151562	22649767	28974216
p-Methylani-sole	9748197	10152209	10452257	17448941	18144505	19610055
2,4-Dimethyl-3-cyclohexenyl-carbaldehyde	9201137	9196866	8945632	7252227	7377582	6682874
Dimethyl tri-sulfide	14862274	15583226	12165249	16994012	17364298	19405388
1-Octanol	10373894	12493729	15708354	16081384	15631787	21690404
(E, E)-2,4-hep-tadienal	12803706	12112089	12180401	21896297	24114113	19073484
3-Penten-2-one	22536304	23989823	22038582	22164465	22532737	22055378
Propanal	19762131	20100306	19151592	21098862	20561396	20913606
1-butanol 2-me-thyl	26830261	28468035	31673472	34032253	39744824	39291606
5-methylfurfu-ral	5236644	5629960	5998057	9869676	8583754	7957375
2,3-Butanediol	5758999	6691935	5745876	34572124	43146904	34198525
1-pentanal	5399816	5266529	5430287	7152965	7442102	7928232
4,5-Dimethyl-thiazole	7930887	8506015	7730652	13006537	13413839	12802235
1-Hexanol	5123456	5588782	4907824	6729836	6254102	6600521
2-Methylbuta-noic acid	68929324	78845044	64001663	99885706	95415735	89733262
ac, acetic ethyl ester	11030570	11458776	11377543	11228546	11960395	11952097
methyl 2-methylpro-panoate	12124283	12792352	11879529	10043711	8705652	9681165
Cuminic alco-hol	26615432	26768374	25788015	27408943	27712223	27971782

1,1-Diethoxyethane	44615934	48346786	52947754	59710673	63143634	70642074
Deca-2,4-dienal	33282196	34281036	30978122	27018762	26394858	27063623
Citronellal	14270231	14302676	14415924	11275665	10709594	13352885
1,8-Cineol	54292706	51843396	54080542	66378722	66412573	62159265
(Z)-6-nonenal	32859868	31344004	28981753	27404373	28036997	24092805
Styrene	14443704	14395118	15422196	13844314	13052012	13638611
1-propanethiol	10778433	10923528	11818956	8935796	8935772	10177344
3-Methyl-3-buten-1-ol	6637967	6859581	6553647	7194228	6665842	6890686
Limonene	18267623	17876042	16922589	20048779	19853332	19757513
n-Nonanal	31930525	32759764	34609473	24223105	23840295	27900629
3-Methylcyclopentane-1,2-dione	92133872	95878852	97197481	3745295	3350818	4797232

Table S2. Annotation, relative abundance, and statistical information of metabolites detected in 2L-HQ and 2L-FQ samples by untargeted metabolomics.

score	2L-HQ1	2L-HQ2	2L-HQ3	2L-FQ1	2L-FQ2	2L-FQ3	VIP	P-value	FDR	Fold_Change	Log2FC
0.9536	482.3	75.55	332.71	1774.4	26327.84	999.38	1.051117	0.375527	0.512821	0.030602	-5.03024
0.9508	373364	386700.2	401026.9	578209.3	558876.9	536071.1	1.354361	0.000669	0.0135	0.693952	-0.52709
0.9493	22035.44	21246.18	35114.16	46991.92	42580.22	24694.26	0.795539	0.227559	0.368064	0.686079	-0.54355
0.9456	69384.5	67618.97	78676.55	195611.4	205703.5	183815.3	1.363936	0.00037	0.010037	0.368602	-1.43987
0.9435	402918.2	361984.2	396075.5	150123.3	77216.8	122266.9	1.306881	0.001111	0.015928	3.320809	1.731535
0.9435	8370.02	8196.11	9800.34	11062.64	7948.54	7834.48	0.042328	0.90068	0.929906	0.98215	-0.02598
0.9384	33364.95	39877.17	61046.88	377.22	161582.8	30675.57	0.447918	0.733821	0.812478	0.697114	-0.52053
0.9334	3347403	2603281	2703127	3906264	3702563	3639506	1.174843	0.053126	0.13716	0.769342	-0.3783
0.9303	6551.68	8658.5	14656.95	26380.92	12325.11	20416.43	0.999642	0.124581	0.244047	0.505174	-0.98515
0.9293	2003753	2213170	2454415	2886629	2593354	2841606	1.17447	0.030771	0.095236	0.80169	-0.31888
0.9252	15280.93	3084.37	14005.62	19007.69	17633.77	18474.94	0.800237	0.188029	0.323449	0.587319	-0.76778
0.9202	270932.6	263250.7	378154.8	105509.9	86766.55	93445.53	1.339838	0.027916	0.089476	3.193097	1.674957
0.92	148856.2	122300.9	176067.9	86334.67	76073.14	80151.97	1.287928	0.043717	0.12084	1.843772	0.88266
0.9197	56665.07	50464.48	48804.16	760.71	2409.85	1015.54	1.349448	0.001515	0.017705	37.25035	5.219182
0.9195	1608.72	268.81	913.43	1681.24	2038.08	1166.16	0.785657	0.216561	0.356356	0.571277	-0.80774

0.9088	6201.32	4451.61	3792.78	574.37	1358.81	4326.22	0.944331	0.126381	0.246607	2.307843	1.206545
0.9035	12270.4	28471.05	22980.39	18087.32	7878.77	6697.23	0.914992	0.163302	0.293903	1.950868	0.964116
0.9021	206192.3	494289.5	412219.7	783288.1	222764.2	216613.4	0.05002	0.871174	0.909132	0.910062	-0.13596
0.8942	8928.25	6764.33	10062.93	528.71	1051.54	694	1.348973	0.013234	0.056224	11.32484	3.501418
0.8924	105022.4	109877.6	64219.22	114517.7	117678.4	97575.86	0.640368	0.369962	0.507798	0.846401	-0.24059
0.8924	97238.74	64899.17	96968.76	973287.3	924966.4	893501.6	1.364029	9.8E-05	0.006015	0.092811	-3.42955
0.89	68719.39	70820.63	87656.28	45979.97	33170.19	27681.41	1.260495	0.00788	0.041384	2.126678	1.088601
0.888	53694.35	49846.74	57324.96	126307	143180.6	155477.7	1.357856	0.006334	0.037461	0.378539	-1.40148
0.8879	106290	95213.19	99013.47	38444.08	35288.22	31520.72	1.362368	0.000236	0.009207	2.855183	1.513583
0.8854	15872.94	13942.66	15750.43	24364.01	28257.87	28381.52	1.338881	0.004726	0.032421	0.56252	-0.83002
0.8849	10163.16	8497.22	7130.28	7675.97	6414.66	5325.32	0.954725	0.132342	0.254438	1.328323	0.409606
0.8811	61649.3	49632.76	59673.92	514483.3	511894.4	463726.1	1.369592	0.000876	0.01464	0.114728	-3.12372
0.8802	1869.04	815.44	2582.61	381.13	324.55	2878.6	0.625567	0.605863	0.710061	1.469497	0.555323
0.8801	17456.08	16819.49	18569.67	59355.06	99540.36	49122.3	1.306982	0.078087	0.178825	0.254042	-1.97686
0.8786	22337.83	23662.12	18873.66	4288.09	14863.77	22671.75	0.737455	0.283573	0.426798	1.551124	0.633314
0.8733	74494.77	79703.88	69355.52	60.11	1874.29	1971.6	1.236891	0.001124	0.015971	57.23353	5.838789
0.8705	1882.55	2636.09	2309.11	3881.22	2368.26	2205.63	0.581175	0.424249	0.553083	0.807529	-0.30841
0.8623	21510.79	23856.06	21624.14	5271.01	4941.5	4644.11	1.37002	0.001184	0.015971	4.509168	2.172861
0.8583	579428.9	550478.1	577898.3	15065.54	14252.44	1375.57	1.294265	2.27E-05	0.003778	55.64053	5.798064
0.8559	413.45	736.06	367.27	458.81	551.89	384.07	0.132301	0.769944	0.838627	1.087477	0.120985
0.8528	10016797	13236578	10644188	5455808	5584051	6115042	1.32501	0.025832	0.084177	1.97597	0.982561
0.8454	79622.59	88039.8	69383.44	198247	322057.8	178607.9	1.288015	0.073533	0.171109	0.339164	-1.55995
0.8452	4357.45	2897.33	3564.08	6733.91	4194.89	5249.55	1.016222	0.120697	0.239185	0.668725	-0.58052
0.8424	16658.26	97757.54	119940.3	22878.16	15197.43	23812.29	0.871246	0.207739	0.346682	3.786786	1.920974
0.8396	17233.27	43506.68	16353.76	11753.42	10598.67	10046.79	1.04898	0.236124	0.378267	2.379518	1.250669
0.8381	17318.35	15564.36	21499.66	5678.2	22650.26	22672.72	0.323178	0.864219	0.904039	1.066296	0.092608
0.835	16687.92	39359.72	72416.9	3833.53	2648.04	23670.51	1.016904	0.16938	0.301482	4.260553	2.091041

0.8307	18528.02	10892.77	12665.26	5677.98	3579.58	4771.57	1.283092	0.047916	0.128216	2.999904	1.584917
0.8306	65830.74	66462	61063.55	82602.42	65723.61	87406.39	0.994398	0.15784	0.286677	0.820236	-0.28589
0.8303	1632.19	1313.97	2023.99	2304.96	1360.28	2833.91	0.588101	0.367358	0.505898	0.764738	-0.38696
0.8238	2139.52	3106.55	1446.93	133.14	748.52	328.29	1.189203	0.04889	0.129866	5.531634	2.467706
0.8184	108984	458687.2	94475.54	163826.5	161294.5	118731.1	0.179845	0.604211	0.708952	1.491818	0.577072
0.8177	26566.87	22077.64	63299.97	10773.19	19806.56	41516.44	0.584529	0.456384	0.583053	1.55271	0.634788
0.8136	645.23	1176.98	193.93	160.69	186.71	18.14	0.977204	0.188989	0.324077	5.515511	2.463495
0.8125	28258.21	32477.77	27946.54	32256.37	35398.5	37123.16	1.094277	0.058287	0.146104	0.846385	-0.24061
0.8115	127934.2	167935.6	127286.7	2672.87	585547.8	14841.63	0.61237	0.784906	0.848773	0.70168	-0.51112
0.8072	13521.51	12746.84	13054.89	67106.34	64457.31	61091.3	1.371791	0.000982	0.014975	0.204112	-2.29257
0.8072	1706.42	2584.9	2032.78	1850.4	535.11	798.59	0.995745	0.105092	0.218886	1.98615	0.989975
0.807	134846.6	39213.2	86694.15	52110.14	43247.31	33282.74	0.848293	0.249233	0.393816	2.027002	1.019348
0.8068	454.69	2059.49	2899.4	7593.84	6971.34	7376.75	1.130267	0.012419	0.054133	0.246723	-2.01904
0.8063	42036.65	10036.33	20937.99	5979.76	1703.07	3119.34	1.184481	0.155727	0.28395	6.758917	2.756792
0.8026	88.91	232.2	292.36	44182.86	47357.36	138.71	0.925094	0.184586	0.319797	0.006692	-7.22345
0.8017	159901.5	195288.6	157560.9	114622.4	125015.8	115755.4	1.269686	0.042171	0.117812	1.44277	0.528841
0.8007	2940.53	3695.2	2336	178.64	128.2	377.49	1.328588	0.016801	0.064416	13.11024	3.712622
0.7992	564669.3	718791.2	587566.4	344394.5	356600.2	373538.7	1.32022	0.028101	0.089803	1.741246	0.80012
0.7986	37133.36	38978.77	36141.03	16244.24	15841.92	23104.5	1.298886	0.008746	0.04428	2.033916	1.02426
0.7984	1030877	1209415	1226945	250983.5	304802.4	270026.6	1.364624	0.003282	0.026946	4.198576	2.0699
0.7973	47093.86	34325.52	21843.6	48491.96	53656.06	29469.93	0.55988	0.412988	0.545354	0.784566	-0.35003
0.7962	127665.4	26870.62	84294.72	31145.45	33651.98	5340.33	0.898338	0.186465	0.321647	3.405166	1.767725
0.795	931706.2	800077.9	861986.2	1556315	754035.4	1502527	0.787884	0.255657	0.401549	0.680266	-0.55583
0.795	10092.73	11910.97	10327.75	34403.01	34191.35	33339.99	1.367211	3.17E-05	0.003946	0.317179	-1.65663
0.7879	1654077	1403730	1528351	1989425	1226396	3547962	0.592867	0.399392	0.533872	0.678046	-0.56054
0.7876	2477.72	1549.2	2902.86	15714.99	13611.26	16004.06	1.344913	0.000597	0.012748	0.152873	-2.70959

0.7865	19143.33	13097.67	18365.23	20171.57	21414.76	21638.92	0.953937	0.150804	0.277365	0.800412	-0.32119
0.784	13077.11	64607.3	56313.85	34613	23186.95	13158.97	0.544156	0.31985	0.462265	1.888392	0.917158
0.7833	13872439	14705995	47612424	8648282	34001738	11165243	0.408268	0.618399	0.720171	1.415785	0.501603
0.783	4770.74	472.23	2464.15	5277.6	4158.69	728.63	0.255538	0.680665	0.769056	0.758208	-0.39934
0.7804	2892.22	4711.64	4524.35	190.79	396.79	689.52	1.298684	0.019518	0.07078	9.49668	3.247423
0.7801	2009.49	3002.77	3514.07	3467.98	5507.32	4051.88	0.972479	0.12274	0.242201	0.654503	-0.61153
0.7798	2143.19	1002.64	3104.91	219.21	372.21	680.48	1.185053	0.105607	0.219211	4.91449	2.297042
0.7792	11725.42	10330.6	9393.49	400.94	2909.84	380.26	1.220726	0.001231	0.016047	8.520501	3.090938
0.7791	793.68	965.34	82.25	1863.04	2146.15	676.68	0.80331	0.160867	0.290498	0.392941	-1.34762
0.7788	24347.09	35386.37	24555.34	52297.39	59139.99	53826.48	1.28595	0.006411	0.037587	0.510026	-0.97136
0.7788	5830.14	4116.11	6199.8	18474.06	19563.83	19389.35	1.346073	0.000301	0.009591	0.281157	-1.83055
0.7786	30945.68	406.93	28842.05	23983.21	1422.66	45433.9	0.184114	0.83675	0.885719	0.84973	-0.23492
0.7786	236.86	392.75	218.27	1058.72	738.55	89.66	0.3457	0.34793	0.488603	0.449344	-1.15411
0.7781	22890.71	42485.55	19547.8	30580.82	29709.07	31882.48	0.389306	0.768143	0.837084	0.921361	-0.11816
0.7776	77232.98	65055.65	76520.64	41894.37	37690.55	52845.47	1.252336	0.009038	0.044899	1.652259	0.72444
0.7776	1417.78	3806.98	3531.37	1920.26	2584.05	2013.99	0.425967	0.429642	0.557053	1.343315	0.425798
0.7773	20396.66	8210.78	13454.29	57718.91	58503.3	57479.57	1.29433	0.006095	0.036575	0.242149	-2.04603
0.7773	8775.87	2444.45	8364.53	6124.82	6647.14	2193.95	0.284832	0.572635	0.682558	1.308631	0.388058
0.7771	42034.15	29424.94	37920.43	11052.97	12051.47	9518.57	1.343644	0.017357	0.065872	3.352833	1.745381
0.7769	13721.15	20010.79	13462.23	11239.02	509.31	9893.72	0.775018	0.112542	0.228705	2.18067	1.124771
0.7767	28434.53	31400.85	32190.66	13755.9	2344.88	13684.07	1.047562	0.024008	0.08081	3.089693	1.627463
0.7766	2329.17	2333.3	1528.93	2999.68	232.23	2085.07	0.463301	0.76098	0.83188	1.164458	0.219659
0.7765	66201.03	32574.02	44810.35	47901.68	49608.63	27015.94	0.320116	0.632792	0.730623	1.153053	0.205459
0.7762	15264.76	11350.57	13769.29	6569.84	5839.65	4271.37	1.297182	0.007472	0.040111	2.421015	1.275612
0.7761	874.97	388.4	600.46	103.23	425.58	363.33	0.88172	0.141063	0.265554	2.089168	1.062928
0.7758	13639.15	18894.44	13313.17	22176.86	19873.87	986.46	0.437658	0.903771	0.931994	1.065282	0.091236

0.7758	1101.44	1239.91	587.98	746.31	640.72	614.58	0.787386	0.256811	0.402242	1.463487	0.54941
0.7755	7997.44	4235.89	5613.29	10278.37	8577.54	11855.75	1.111188	0.042821	0.119119	0.581102	-0.78314
0.7755	496005.1	620433.7	584817.3	638995.3	694542.8	662734	1.035702	0.10115	0.212668	0.852217	-0.23071
0.7753	22771.01	22045.55	19801.05	77626.27	20820.95	68982.21	0.947274	0.191729	0.327572	0.385939	-1.37355
0.775	45582.09	24048.35	34534.41	51848.39	47465.11	26802.22	0.4338	0.503195	0.621304	0.825947	-0.27588
0.7749	195726.2	215288	240923.1	68421.02	95752.61	95327.7	1.324665	0.001949	0.020396	2.51227	1.328991
0.7746	52053.91	37823.91	37730.18	34134.75	36332.62	30682.34	0.943462	0.197403	0.334817	1.261576	0.335227
0.7744	1939.39	4491.33	3723.34	10181.88	4101.37	7335.94	0.977986	0.14902	0.275128	0.469678	-1.09026
0.7743	2024.47	3640.7	2191.03	5885.57	2400.98	6049.44	0.845937	0.203413	0.341819	0.548005	-0.86774
0.7743	2655.37	5068.64	5655.85	643.17	541.9	518.81	1.334491	0.051164	0.133408	7.852584	2.973167
0.7728	131534.4	86977.9	108940.7	329929.2	340161.6	307888.6	1.340276	0.000281	0.009591	0.334826	-1.57852
0.7721	4365.11	8922.88	8024.6	4336.16	6156.69	4681.25	0.723124	0.278061	0.421735	1.404537	0.490095
0.772	17.35	80.91	83.56	92.08	79.33	120.11	0.73049	0.232667	0.37438	0.623696	-0.68108
0.7715	177435.3	236472.9	214491.2	223789.4	287653.3	227994.8	0.78569	0.242552	0.385879	0.849834	-0.23475
0.7712	596.31	756.1	28.42	518	286.2	9.01	0.306114	0.520612	0.636742	1.697999	0.763836
0.771	294.39	570.79	3934.26	324.77	2810.99	2555.96	0.269412	0.844768	0.890315	0.843232	-0.246
0.7708	35817.5	25929.35	29386.35	14404.37	15125.96	20399.77	1.239404	0.022088	0.076615	1.825216	0.868067
0.7702	30627.17	19871.44	19502.99	11909.19	14602.09	13153	1.187661	0.103335	0.215934	1.764852	0.819548
0.7702	37956.76	45223.38	46744.95	1733.51	599.85	2480.74	1.33201	0.003138	0.026301	26.98845	4.75427
0.7694	79552.1	76382.04	88485.23	60223.29	58489.89	51866.45	1.295587	0.007028	0.039121	1.432875	0.518913
0.769	23254.83	27340.52	30316.73	4086.66	1281.18	1027.3	1.308503	0.001985	0.020503	12.65212	3.661307
0.7677	224521.7	184452.8	218476.1	142976.2	142645.2	141205	1.306572	0.032852	0.099869	1.470037	0.555852
0.767	1042.97	977.6	66.16	1141.23	88.89	762.12	0.026672	0.946439	0.961661	1.047429	0.066852
0.7664	5671.75	4909.19	4948.6	5963.65	3275.03	5309.7	0.333366	0.731237	0.810646	1.067441	0.094157
0.7658	51963.43	34831.33	45621.1	120169.8	109335.6	129380	1.331124	0.000657	0.013454	0.368964	-1.43845
0.7656	5969.03	221263.6	5408.1	45300.1	13262.13	120024.1	0.370281	0.834494	0.883547	1.302679	0.381482

0.7654	50.36	7.07	20.47	105.17	161.04	72.64	1.116372	0.058624	0.14678	0.229895	-2.12095
0.7649	341.37	408.86	630.85	540.53	443.63	502.81	0.361132	0.732145	0.811447	0.928788	-0.10658
0.7644	42098.49	41684.52	50261.3	116918.9	126113.1	113864.9	1.359757	0.00014	0.006718	0.375583	-1.4128
0.7643	105515.7	92381.1	106486.1	54733.24	55257.26	61390.85	1.344879	0.003779	0.028899	1.776056	0.828677
0.7637	64862.43	104069	65888.01	4361.35	6937.06	2484.4	1.33766	0.028295	0.090224	17.03712	4.09061
0.7636	20856.95	12642.83	26746.79	131930.3	100072.1	136073.9	1.328214	0.006548	0.038003	0.16368	-2.61105
0.7631	6939.02	6716.88	4427.84	32667.49	14925.13	12879.47	1.193817	0.151402	0.278348	0.299043	-1.74158
0.7622	11166.78	8877.83	11547.14	8966.64	8780.02	9832.38	0.807857	0.245335	0.388596	1.145499	0.195976
0.762	120860.8	135503.8	148539.5	88391.04	101617.5	71373.2	1.211416	0.015918	0.062455	1.549091	0.631422
0.761	304.52	360.07	521.42	161.96	555.33	299.84	0.419941	0.697883	0.783635	1.166036	0.221612
0.7609	2476.55	3044.37	4413.39	298.82	2563.79	3026.51	0.688585	0.265387	0.411095	1.686892	0.754368
0.7609	482.56	902.24	2090.5	652.92	217.63	712.55	0.742139	0.319948	0.462265	2.19525	1.134385
0.7602	26602.23	25503.15	25919.68	31480.68	34761	36343.9	1.306214	0.024678	0.082241	0.760585	-0.39482
0.7597	2974.5	4256.58	6713.86	3294.84	2350.09	1045.63	0.945133	0.147151	0.27291	2.084271	1.059543
0.7596	13161.03	15963.43	7252.69	10791.16	4769.69	9710.6	0.644054	0.313193	0.455677	1.439456	0.525524
0.7594	24735.53	24903.13	28743.1	140216.9	125568.3	137131.4	1.369222	0.000797	0.014349	0.194536	-2.36189
0.7589	39017.01	23100.16	28302.12	9152.75	105805.8	6015.39	0.359239	0.786298	0.849856	0.747428	-0.41999
0.7583	71981.27	56772.45	170845.7	22444.57	17028.54	23322.58	1.238192	0.157598	0.286357	4.771019	2.254297
0.7579	215.31	68.09	172.96	442.73	651.79	19.09	0.149729	0.360275	0.5006	0.409802	-1.287
0.7575	16486.86	21148.16	38740.48	16607.73	9728.55	2535.05	0.946336	0.131072	0.253143	2.645375	1.403472
0.7564	30718.05	33620.45	39542.34	29539.44	28109.5	41343.74	0.274412	0.760805	0.83188	1.049379	0.069536
0.7561	47791.99	63920.34	74269.56	45303.97	39116.59	37971.73	1.129424	0.100527	0.211952	1.519556	0.603649
0.7549	45693.31	28798.74	29121.96	19523.41	20153.73	21377.45	1.170952	0.124718	0.244115	1.697072	0.763047
0.7545	260837.9	161266.4	223954.5	266898.2	302286.1	335144.6	1.026125	0.079005	0.179929	0.714407	-0.48518
0.7544	3004.22	2551.47	3908.13	4103.07	1264.92	4217.79	0.171967	0.971692	0.978652	0.987277	-0.01847
0.7542	29945.61	21474.19	12190.7	18721.2	37661.12	25810.68	0.537625	0.457591	0.584327	0.773916	-0.36975

0.7541	1302.66	808.97	886.65	246.49	837.73	828.07	0.751895	0.222671	0.362985	1.5679	0.648834
0.7536	983549.2	1074581	1008851	8779.2	1278536	63091.52	0.883553	0.301283	0.44398	2.271155	1.183426
0.7529	29764.39	35073.07	33246.26	8612.58	17022.85	18225.9	1.191536	0.013116	0.055996	2.236223	1.161064
0.7522	3863.41	2286.58	3713.35	6486.75	4049.99	4635.14	0.968857	0.126873	0.247335	0.650107	-0.62125
0.7519	16995.31	16094.14	11799.23	17789.54	19996.55	15035.1	0.706251	0.287214	0.430673	0.849823	-0.23477
0.7518	80632.45	88066.57	77141.33	82546.78	93106.16	81659.66	0.505861	0.47847	0.601488	0.955415	-0.0658
0.7518	30712.73	22144.63	15703.01	5410.29	6312.32	8597.92	1.276057	0.059974	0.148623	3.373946	1.754437
0.7515	21160.26	25889.14	15081.09	158104.1	159034.9	148788	1.356689	7.76E-06	0.003019	0.133348	-2.90673
0.7512	103725	106267	119569.7	60735.59	61444.49	64984.86	1.353168	0.007245	0.039577	1.760809	0.816238
0.7501	11055.67	11647.84	10694.36	15273.02	1470.13	13059.1	0.44362	0.805955	0.863988	1.120649	0.164335
0.7499	190.94	211.5	54.87	310.27	138.67	141.66	0.432927	0.586243	0.693281	0.774314	-0.36901
0.7499	15.93	1793.44	103.23	384.72	801.12	323.83	0.530108	0.84084	0.887893	1.266899	0.341302
0.7496	302690.3	336255.4	271032.5	272505.3	203290.1	192313.1	1.079406	0.066797	0.160323	1.362022	0.44575
0.7495	3396.29	1882.88	3569.76	3487.83	2176.83	3383.69	0.104779	0.927206	0.948261	0.977961	-0.03215
0.7477	66952.1	68535.61	71496.06	206119.5	199454.3	110519.9	1.244754	0.078722	0.179753	0.401059	-1.31812
0.7474	2150.47	3530.68	2980.38	27797.78	28848.93	23732.42	1.359502	0.002656	0.024066	0.107758	-3.21413
0.7474	1900.53	1304.52	679.84	3784.8	2645.46	1437.43	0.899523	0.180364	0.314482	0.493778	-1.01807
0.7468	284844.5	347623.4	180285.5	268053	257679.6	223299.2	0.163357	0.710828	0.793876	1.085072	0.117791
0.7468	135797.6	116601.2	117230.9	115094.5	123459	102313.2	0.660619	0.337229	0.477732	1.084382	0.116873
0.7465	89304.71	162789	142748.4	155933.8	293159.3	380719.6	1.01719	0.146755	0.272446	0.475821	-1.07151
0.7461	49381.22	114649.6	140234.6	74139.13	52857.51	55734.39	0.740972	0.270219	0.415793	1.6651	0.735609
0.7449	12232.13	6027.54	5004.53	18914.07	3450.49	19961.89	0.430357	0.361171	0.500654	0.549637	-0.86345
0.7443	4135.24	3126.38	3774.58	10800.11	7745.76	12921.76	1.303349	0.040941	0.11581	0.350716	-1.51163
0.7439	29242.11	38231.22	37191.93	16085.78	14962.3	15576.62	1.34156	0.019752	0.071333	2.244846	1.166616
0.7438	5384.77	5408.8	4037.67	4917.27	1797.4	2820.25	0.885324	0.18567	0.320782	1.555466	0.637346
0.7424	57296.06	61035.28	41805.96	121176.6	119469.6	114308.2	1.318037	0.004121	0.030042	0.451149	-1.14832

0.7423	13102.58	13875.61	16340.64	7648.79	8504.11	10944.88	1.215147	0.017641	0.066462	1.598612	0.67682
0.7423	9157.54	12651.66	14868.43	19690.15	15377.35	13592.45	0.862056	0.179939	0.314032	0.753754	-0.40783
0.7422	5778.05	5771.14	6133.2	11435.11	10774.14	11038.72	1.367973	8.47E-05	0.00568	0.531834	-0.91095
0.7422	11208.58	13512.3	11351.61	3744.07	7423.13	6182.7	1.199509	0.011933	0.052753	2.079118	1.055972
0.7421	31400.84	28610.55	30036.88	10385.37	9133.95	12316.66	1.352693	0.000106	0.006088	2.828506	1.50004
0.7419	3748.56	1256.59	2619.39	5073.42	6508.53	4866.81	1.08209	0.034076	0.102438	0.463533	-1.10926
0.7415	225262.7	215490.7	52219.88	140464	150705.8	194184.3	0.224512	0.96879	0.977084	1.015698	0.022472
0.7403	183.42	2076.83	187.89	221.97	75.23	66.68	0.843753	0.385297	0.521933	6.727877	2.750151
0.74	19619.73	2108.36	18132.23	5854.87	4875.15	4194.46	0.526527	0.275908	0.420225	2.670801	1.417273
0.7399	931.14	275.55	570.13	73.92	233.29	4213.08	0.125929	0.570151	0.680155	0.393077	-1.34712
0.7398	616.51	380.41	41.53	49.77	304.24	329.33	0.145079	0.575239	0.684147	1.519668	0.603756
0.7393	19617.91	17872.42	34530.93	11835.14	10247.84	10885.04	1.194383	0.131764	0.253676	2.184579	1.127356
0.739	1524.88	2803.9	1284.53	514.66	234.36	1627.09	0.92333	0.165239	0.296167	2.362395	1.24025
0.7373	127102.2	148977.2	119146.1	231993.1	256425.9	150743.8	1.09012	0.116924	0.234503	0.618349	-0.69351
0.7346	6285.97	186.71	6486.97	2406.23	2039.16	110.48	0.377144	0.30729	0.45066	2.844605	1.508228
0.7343	32255.4	29274.25	34690.72	19579	18961.94	22671.95	1.305431	0.005076	0.033561	1.571897	0.652507
0.7342	7965.19	8009.45	9124.63	5769.95	4891.78	3400.5	1.208584	0.017249	0.065552	1.784871	0.83582
0.7341	298945.4	212823.9	274747.9	264317.6	216871.5	244662.7	0.410506	0.536192	0.65148	1.083578	0.115803
0.7329	108472.7	103457.5	98781.03	85534.42	93195.41	116216.4	0.409989	0.632589	0.730623	1.053451	0.075123
0.7329	3863.44	3459.32	4977.93	13092.41	2836.19	4086.57	0.381142	0.510415	0.628325	0.614568	-0.70235
0.7327	1611.16	1587.38	1208.49	3213.79	4159.72	3660.4	1.329353	0.006185	0.036834	0.399408	-1.32407
0.7327	14770.67	11357.41	739.22	508.84	291.52	54	1.109404	0.176437	0.310366	31.44728	4.974863
0.7326	28092.09	16061.7	23160.39	54280.81	50292.54	49506.16	1.279126	0.00674	0.038427	0.43688	-1.19469
0.7325	65447.45	36610.1	63287.8	54400.75	415245.4	440537.7	0.960956	0.184115	0.31927	0.181661	-2.46068
0.7322	12157.55	5947.03	13632.54	2610.71	1753.86	5024.21	1.155461	0.070768	0.166385	3.380324	1.757162
0.731	11343.14	11909.71	10662.12	3345.75	5188.88	5251.19	1.303357	0.002021	0.020676	2.460134	1.298737

0.7305	98901.81	77787.63	100626.6	67643.06	63409.57	58556.32	1.230604	0.044947	0.122758	1.462568	0.548504
0.7303	71937.13	68826.39	63293.46	54714.31	53314.64	49572.41	1.287182	0.010648	0.049587	1.294767	0.372692
0.7299	43506.08	31700.01	42910.73	66325.89	63785.71	61345.18	1.261468	0.014726	0.059716	0.616937	-0.6968
0.7296	24275.11	19739.85	22945.21	32507.46	30996.22	31681.86	1.293399	0.013171	0.056121	0.70347	-0.50744
0.7296	149460.5	203828.3	193319.3	30723.29	123542.3	113357.3	0.943478	0.066483	0.159926	2.042456	1.030305
0.7292	30590.36	31182.67	37463.25	72000.61	61347.15	68920.16	1.33906	0.001462	0.017416	0.490618	-1.02733
0.7284	17007.75	13446.08	9660.92	3581.27	7969.92	6486.07	1.121987	0.052643	0.136266	2.223994	1.153153
0.7265	372397.8	332339.5	356151.6	102204.2	62535.53	114450.8	1.323534	0.000294	0.009591	3.799874	1.925951
0.7265	36053.54	30482.07	43558.7	12139.28	3961.66	7612.51	1.266528	0.005349	0.034388	4.642695	2.214962
0.7257	1462.63	1477.88	2051.37	5182.83	4378.94	1560.29	0.908721	0.200932	0.338695	0.448827	-1.15577
0.7237	5187.04	249.77	141.98	2648.75	2654.25	2200.01	0.757505	0.737502	0.815104	0.74354	-0.42752
0.7231	8466.61	104200.1	82009.45	57659.6	53181.18	31321.64	0.077296	0.612027	0.715044	1.369392	0.453536
0.7225	9340.47	13466.95	14721.44	62198.51	22623.48	66947.2	1.189694	0.111549	0.227749	0.247276	-2.01581
0.7224	84924.73	82434.9	93729.96	54780.42	50259.51	54875.46	1.347074	0.004	0.029455	1.632673	0.707236
0.7223	806926.2	448154.2	506415.6	630556	620790.3	357191.8	0.234964	0.739763	0.816568	1.095091	0.131051
0.7216	56639.41	48603.73	52308.2	38782.25	36789.81	45241.4	1.192576	0.024144	0.08108	1.304088	0.383041
0.7214	39075.41	61455.34	46308.55	10385.44	43.65	5858.86	0.980072	0.011313	0.051196	9.015211	3.172361
0.7213	10283.15	16095.72	8035.63	976.48	1996.62	1651.39	1.316791	0.0518	0.134347	7.441794	2.89565
0.7202	27076.34	41376.34	29786.37	28050.47	41604.58	38227.12	0.365438	0.619868	0.721277	0.910614	-0.13509
0.7202	72997.85	69893.41	67662.22	58139.96	55560.55	58081.62	1.327936	0.004671	0.032316	1.225701	0.293607
0.72	44407.61	57116.26	79446.94	68129.93	44064.79	67929	0.010785	0.983755	0.988062	1.004703	0.006769
0.7198	1344868	1229946	1074589	1098273	1006325	1199563	0.697731	0.303957	0.446411	1.104487	0.143376
0.7186	2735.59	302.52	836.83	2886.94	175.78	36.01	0.536154	0.838462	0.887316	1.250493	0.322497
0.7183	17421.58	16190.53	12756.5	6695.32	5772.74	2319.31	1.198251	0.005518	0.034788	3.13569	1.648783
0.7175	2685.89	461.43	114.7	2389.65	1984.13	2164.79	0.845882	0.307134	0.45066	0.498889	-1.00321
0.7153	22231.29	22740.64	23349.04	8107.85	10648.56	8185.01	1.348097	0.00129	0.016383	2.535908	1.342503
0.7147	26724.98	24079.12	28609.13	95744.95	77729.7	88424.42	1.360951	0.005012	0.033406	0.303221	-1.72156

0.7141	987.91	473.15	2929.38	86.64	1079.52	565.27	0.695083	0.361011	0.500654	2.535731	1.342401
0.7138	488243.3	473403.5	546893.5	446126.6	414381.2	450350.3	1.107038	0.080037	0.181144	1.150804	0.202642
0.7136	718.93	783.68	1620.24	614.69	1176.01	672.47	0.419523	0.560411	0.673229	1.267817	0.342347
0.7132	3326.21	3421.81	6938.77	6539.23	6220.91	6501.74	0.879215	0.257853	0.403439	0.710564	-0.49296
0.713	758.32	716.19	1206.51	415.41	723.19	139.4	0.917476	0.112498	0.228705	2.097825	1.068894
0.7116	575.39	123.01	341.69	65.47	271.91	248.89	0.549552	0.377804	0.514962	1.77408	0.827071
0.7108	9405.97	7193.06	7578.1	8329.89	9275.43	8234.02	0.508293	0.520018	0.636373	0.935671	-0.09593
0.7105	238.85	1097.45	1748.77	120.44	11289.22	10016.49	0.455368	0.224062	0.364435	0.143986	-2.796
0.7095	46176.24	32007.49	25333.49	298946.9	305051.4	324656.2	1.356253	1.56E-05	0.003622	0.11147	-3.16527
0.7084	36042.98	26720.65	39424.43	33877.62	34452.71	31059.88	0.087512	0.832334	0.882546	1.02815	0.040051
0.7083	105791.5	131939.7	96472	219250.9	203296.2	101223.6	0.834577	0.224441	0.364792	0.638072	-0.64821
0.7082	4209.05	1619.31	5862.51	16752	14705.72	20167.14	1.231099	0.00336	0.027275	0.226458	-2.14268
0.7078	96785.53	89243.86	39013.19	17313.68	22001.74	15784.99	1.248061	0.087461	0.193425	4.084227	2.030063
0.7077	176492.2	180559.9	170666	239385.2	221587.7	217922.7	1.327126	0.008205	0.042393	0.777319	-0.36342
0.7054	2919.35	1746.82	4543.47	1170.03	309.75	975.83	1.116668	0.097493	0.208014	3.750449	1.907063
0.7052	966.75	767.97	351.19	80600.85	78183.16	1244.13	1.058379	0.180757	0.315042	0.013035	-6.2615
0.705	421.08	60.67	63.75	842.11	750.7	520.12	1.10455	0.029093	0.091962	0.258172	-1.95359
0.7037	19138.96	22710.33	20600.92	13877.78	13780.62	13150.44	1.332959	0.016413	0.063371	1.530311	0.613825
0.7007	67822.02	67548.36	60891	19331.26	23905.18	10651.72	1.29053	0.001325	0.016575	3.642013	1.864736
0.9993	89902.08	75262.74	82640.12	21791.25	21102.21	22278.1	1.368466	0.004569	0.032241	3.802348	1.926891
0.9989	374943.2	306534.7	324685.3	6040.34	7282.97	6087.13	1.371611	0.003836	0.029026	51.83619	5.695888
0.9952	3544449	2946903	3242674	3694334	3231442	3488067	0.635427	0.361334	0.500654	0.93472	-0.09739
0.9904	204950	311088	206575	134160.1	107231.1	98757.03	1.254223	0.057753	0.145349	2.124406	1.08706
0.9897	90749.86	81325.94	86455.2	107318.5	92956.65	124918.2	1.064083	0.128735	0.250069	0.795007	-0.33096
0.9863	139.79	233.72	64.4	493.59	80.96	155.88	0.369058	0.532828	0.648652	0.599524	-0.73811
0.9858	1425010	1642270	1843777	929413.1	1051868	1269822	1.195164	0.025655	0.083892	1.510582	0.595104

0.9852	1563807	1669116	1483738	227882.1	222900.5	226126.1	1.372038	0.001574	0.018061	6.967942	2.800733
0.9848	59905.93	46379.63	52297.77	86048.47	81679.45	82800.54	1.305176	0.009861	0.047393	0.632995	-0.65973
0.9828	81383.33	62989.57	86342.9	879.52	8044.74	10336.11	1.205647	0.00453	0.032107	11.97878	3.582409
0.9826	13746.85	11388.59	11944.15	974.53	1995.92	1383.72	1.346886	0.001368	0.016837	8.51588	3.090156
0.9823	28457.07	27985.08	20712.98	316.09	1899.63	832.18	1.312925	0.008388	0.042963	25.31419	4.661875
0.982	123784.1	106997.6	123284.7	103206.8	88962.41	57122.45	0.985223	0.109792	0.225747	1.42029	0.506185
0.9808	34989.42	22258.87	33806.64	9151.66	8052.37	9016.97	1.333252	0.032742	0.099805	3.472596	1.796014
0.9793	169.35	1767.07	2191.09	495.27	3209.26	3982.38	0.463305	0.399314	0.533872	0.536953	-0.89713
0.9791	89073.93	83019.31	75546.38	72089.79	76218.07	66824.5	1.033551	0.093401	0.202699	1.151104	0.203018
0.9784	1377	1273.37	5785.06	3128.12	3770.18	6767.83	0.732582	0.405912	0.539442	0.617251	-0.69607
0.9779	58947.9	55978.65	154496	51478.61	101468.5	106769.2	0.050207	0.9354	0.953334	1.037372	0.052934
0.9761	3925.7	3860.34	4405.17	1105.81	1758.57	974.79	1.311365	0.001172	0.015971	3.175481	1.666975
0.976	66321.01	100979.1	118201.5	12266.93	12991.77	27268.08	1.293007	0.027361	0.08828	5.435354	2.442374
0.9746	90234.86	95139.4	91620.22	85157.83	78681.54	72933.57	1.179107	0.047212	0.127089	1.169874	0.226353
0.9742	148994.5	123040.1	159994	138341.6	123071	129570.2	0.667975	0.340952	0.481754	1.104981	0.144022
0.9727	2217.9	3107.23	3582.54	2669.63	1453.8	2486.78	0.753838	0.236873	0.379189	1.347562	0.430352
0.9727	408.79	101.35	520.67	829.95	459.59	800.23	0.864179	0.110673	0.22681	0.493265	-1.01957
0.9725	12573.29	11487.14	10492.58	12918.52	12475.24	13366.59	0.994743	0.130814	0.252869	0.891452	-0.16577
0.9718	6097951	3060721	5188587	12104069	8260983	11040967	1.184199	0.019406	0.070615	0.456832	-1.13027
0.9709	579948.7	490759.2	432774.6	21448.24	23771.97	25278.26	1.370027	0.007902	0.041433	21.32645	4.414572
0.9708	135170.2	109848.1	118204.8	41213.65	29250.19	25721.8	1.338853	0.00123	0.016047	3.776272	1.916963
0.9702	239099.3	142096.2	222134.2	69107.65	64090.26	107002.1	1.230636	0.039123	0.112565	2.51178	1.32871
0.9688	1342702	1477678	1648824	2012377	1799445	1953071	1.212259	0.020103	0.07222	0.775245	-0.36728
0.9687	1516485	2146591	1675153	1256531	1333413	1225323	1.149666	0.111497	0.227749	1.399176	0.484577
0.9687	280831.9	376913	206489	2246.85	4549.51	8866.13	1.34343	0.028933	0.091723	55.17857	5.786036
0.9678	3265.35	582.27	1600.87	26623.81	22321.57	19181	1.287166	0.005455	0.034679	0.079976	-3.64429
0.9677	59887.25	47205.47	65919.64	1396.12	1596.43	28114.29	1.103911	0.015899	0.062437	5.561875	

0.9672	49755.39	38631.24	45039.81	116855	112091.2	116928.7	1.356488	0.000332	0.010018	0.385765	-1.37421
0.9663	93085.89	87875.52	97204.9	54709.52	56969.37	58090.57	1.359982	0.002392	0.022717	1.638494	0.712371
0.9651	123806.9	110833.4	120119.2	19742.92	8264.07	6806.95	1.325701	4.67E-05	0.004503	10.19016	3.349104
0.9646	3041314	3191522	137508.5	358209.8	391511.9	407019.7	0.617144	0.222465	0.362785	5.507147	2.461305
0.9627	211075.6	180845.5	235795.5	90479.51	91322.27	93205.06	1.349209	0.017574	0.066381	2.282549	1.190646
0.9605	3709.65	3929.14	2705.95	2200.29	1255.73	4900.32	0.54455	0.614127	0.71692	1.237951	0.307954
0.9603	16400.76	14686.6	19112.49	17607.58	26680.61	20275.86	0.884263	0.211042	0.350112	0.77752	-0.36305
0.9603	2723193	2754217	2518501	4198105	4050542	4665374	1.343161	0.00611	0.036586	0.619165	-0.6916
0.9598	4686198	4730954	4321026	485054.2	537371.8	551159.9	1.371479	0.000804	0.014349	8.730492	3.126063
0.9596	2379408	2500914	2266383	246969.2	285026	245641	1.371131	0.000706	0.013717	9.190294	3.200111
0.9585	190221.6	192055.7	176843.9	76595.41	75615.86	80228.06	1.369264	0.000934	0.014963	2.40545	1.266307
0.9584	237180.8	215793.7	211946.2	304711.8	279050.5	276180.3	1.290366	0.005956	0.036092	0.773215	-0.37106
0.9579	16701.06	16774.5	17804.64	18998.24	16598.6	4117.38	0.573254	0.491434	0.610666	1.29123	0.368746
0.9572	18857.62	14522.76	27339.03	4711.35	3211.44	7733.25	1.250962	0.044925	0.122758	3.878338	1.955438
0.9572	256039.9	308958.2	266943.2	165505.4	169436	176832	1.332134	0.018685	0.068966	1.625605	0.700976
0.9551	283818	237030.7	105940.8	45521.98	58790	352.94	0.907311	0.069993	0.165097	5.988534	2.582203
0.9549	343014	284650.1	340107.5	408068	427435.5	409997	1.233175	0.029932	0.093528	0.777014	-0.36399
0.9538	2341.86	3484.5	5042.99	1397.77	172.79	987.64	1.074654	0.053434	0.137658	4.248827	2.087065
0.95	5181.6	3420.51	7116.01	22748.24	518.71	1676.07	0.361049	0.713132	0.795634	0.630161	-0.66621
0.95	114079.4	71539.88	106529.6	79108.77	89561.74	90399.03	0.43664	0.492636	0.611621	1.127685	0.173364
0.95	21895.16	36658.37	26851.83	18571.79	18492.14	16954.69	1.124908	0.135386	0.258158	1.581036	0.66087
0.9498	46818.37	24139.62	17991.48	6966.84	9223.28	9363.95	1.222623	0.136394	0.259852	3.480834	1.799433
0.9492	31921.4	30875.75	28757.25	63573.52	68966.84	76796.91	1.357599	0.006815	0.03873	0.437354	-1.19313
0.9491	98060.69	93198.83	73346.14	8289.84	4514.72	9112.49	1.349243	0.007035	0.039121	12.07305	3.593718
0.9475	1827.44	1663.67	1999.55	12725.64	15022.79	7465.81	1.335093	0.04713	0.12705	0.155922	-2.68111
0.9472	87874.33	86441.69	82748.09	97646.36	142527	96128.35	0.955578	0.22388	0.364411	0.764385	-0.38763

0.9471	92718.19	102564.5	86076.56	198176.1	308770.5	227631.3	1.316289	0.042216	0.117812	0.383022	-1.3845
0.9466	206247	131167.5	219963.9	63939	55595.88	46481.47	1.310536	0.038194	0.111439	3.357371	1.747332
0.9457	66599.88	47636.36	54642.85	46687.35	44768.36	42545.19	1.016756	0.165542	0.296229	1.260283	0.333748
0.9453	505727.6	474594.8	512845.3	41178.32	36898.92	41289.05	1.37213	0.000556	0.012569	12.50912	3.644909
0.9449	122211.4	44065	50149.78	21400.59	19840.69	22893.68	1.185234	0.180293	0.314482	3.374543	1.754692
0.9445	4217.17	3351.16	3054.78	2260.11	2020.27	1676.61	1.252255	0.029718	0.093196	1.783302	0.834551
0.9441	1705754	1545453	1715146	1116775	1132798	1120593	1.352087	0.010104	0.047978	1.473622	0.559367
0.9439	120.89	49.88	62.51	200.14	227.55	263.73	1.237999	0.006415	0.037587	0.337393	-1.5675
0.9438	135358.1	134045.4	127713.2	15549.79	16616.97	15306.03	1.372329	0.00029	0.009591	8.365143	3.06439
0.9416	2025.88	3230.38	2608.29	3256.99	1564.91	3641.35	0.063844	0.800873	0.861279	0.929259	-0.10585
0.9413	167689.4	357466.7	154938.8	104932.7	150887.5	93345.05	0.975571	0.22961	0.370693	1.947774	0.961826
0.9412	59299.42	60126.3	54991.3	13815.87	11134.57	15030.32	1.362052	4.6E-05	0.004503	4.362524	2.125163
0.9406	23705.08	20641.93	25767.14	881.83	1967.3	1791.95	1.348902	0.003242	0.026783	15.10729	3.917173
0.9404	40225.46	36333.3	44423.07	184490.6	154180.7	156315	1.36358	0.004223	0.030527	0.244414	-2.0326
0.939	5354	669.14	6606.01	14841.29	11383.03	15549.01	1.000471	0.014849	0.059876	0.302326	-1.72582
0.9389	483909.2	500318.8	451445.9	57281.9	61735.57	62218.79	1.371837	0.001047	0.015525	7.92156	2.985785
0.9385	263277.1	217988.8	205252.3	4646.43	7475.08	4044.04	1.365203	0.006007	0.036267	42.46798	5.408304
0.9362	27723.43	41773.64	36266.66	58892.42	85742.31	48802.02	1.12754	0.104147	0.217422	0.546761	-0.87102
0.9358	187142.9	135994.6	169037.7	70054.09	76857.76	72017.39	1.333533	0.024263	0.081247	2.248102	1.168708
0.9355	115530.5	71288.89	73888.95	16749.07	16108.18	16565.68	1.348557	0.038991	0.112432	5.275048	2.399184
0.9355	2147.07	601.96	1664.42	378.69	960.03	139.32	0.929593	0.153314	0.281032	2.986015	1.578222
0.9332	21688.14	45616.17	45817.11	3731.37	8978.53	2572.19	1.262777	0.04833	0.129104	7.402222	2.887958
0.9331	115.85	446.07	548.61	781.02	501.51	56.36	0.062014	0.777187	0.843452	0.829441	-0.26979
0.9324	12042.24	8170.22	17756.53	99246.7	117182.2	106790.3	1.343621	0.000464	0.011297	0.117471	-3.08962
0.9293	115012.3	113723.7	118536.2	21348.18	455.08	354.62	1.172103	0.003037	0.025806	15.67262	3.970175
0.9289	34.03	12684.67	424.32	2384.69	2121.87	46.82	0.026476	0.563666	0.675184	2.886432	1.529287
0.9284	58826.32	58091.98	59259.4	36.34	236.76	146.69	1.351354	2.08E-05	0.003773	419.6806	8.713148

0.926	360320.4	318267.8	374834.4	93100.34	123943.2	93717.52	1.35275	0.000707	0.013717	3.389815	1.761207
0.9243	10144.29	10730.78	14360.31	5584.7	7326.06	3755.23	1.191314	0.023048	0.078797	2.114209	1.080118
0.9218	11495.4	10863.13	41.11	58.15	43.35	110.54	0.897926	0.184918	0.320151	105.6387	6.722995
0.9213	9062.74	18091.66	9306.13	793.07	3078.2	3148.16	1.188495	0.072314	0.169221	5.194229	2.37691
0.9186	12357.51	5346.12	4506.18	6647.12	3222.01	3788.88	0.677966	0.376984	0.514481	1.626138	0.70145
0.9171	2454.79	1066.14	1912.08	7042.31	2898.71	5287.05	1.122934	0.100727	0.212117	0.356776	-1.48691
0.912	15503.84	13940.4	15686.83	32949.25	34359.43	35537.11	1.365415	6.01E-05	0.005041	0.438823	-1.18829
0.9118	1768762	1431107	2020174	2071969	2511370	1827873	0.828859	0.207197	0.346446	0.814205	-0.29654
0.907	48956.67	39089.16	48047.34	60314.67	63077.18	59599.98	1.231435	0.028205	0.090004	0.743712	-0.42718
0.9047	48107.21	46220.92	34399.63	13363.89	10316.81	10998.83	1.345926	0.014919	0.059993	3.711923	1.892167
0.9016	10921.98	19003.99	13623.14	25908.08	43560.75	22177.85	1.116608	0.123307	0.24288	0.475185	-1.07344
0.897	12059.33	17257.24	14757.24	13239.52	9899.32	9260.62	0.983604	0.118361	0.235802	1.360325	0.443952
0.896	3425.97	2870.76	2406.15	3130.32	1520.38	505.39	0.792428	0.2584	0.404005	1.687884	0.755215
0.8945	75450.71	93254.63	96071.78	94517.33	59840.51	50894.39	0.796437	0.275254	0.419815	1.290008	0.367381
0.8911	18322.37	13929.49	26151.05	256602.3	98699.77	107244.5	1.293115	0.118745	0.236243	0.126264	-2.98549
0.891	66336.59	60199.61	64075.93	50329.32	48305.75	51866.66	1.31582	0.006183	0.036834	1.266511	0.34086
0.888	1927.61	2241.01	677.65	2868.84	1785.9	258.18	0.217356	0.981598	0.98635	0.986434	-0.01971
0.8821	25061.87	24052.16	28270.06	24890.43	23679.26	23443.47	0.768363	0.292835	0.4353	1.074583	0.103776
0.8791	71594.06	74827.55	67751.96	16082.34	15909.5	12778.53	1.365877	0.000142	0.006719	4.783824	2.258164
0.8777	124.97	4094.71	1360.12	268.39	499.05	342.67	0.546664	0.331665	0.473078	5.026349	2.329511
0.8765	1072.91	336.17	30293.86	11787.29	8738.9	9058.54	0.658557	0.949589	0.96329	1.071598	0.099764
0.8739	581437.3	541920.1	583646.2	472809	430948.5	451395.6	1.310944	0.003095	0.0261	1.259639	0.33301
0.8684	343423.4	176540.6	155350.4	109198	452447.3	296895	0.207126	0.631277	0.72971	0.786584	-0.34633
0.8684	979.74	1476.97	966.35	3420.77	1777.15	2609.61	1.186441	0.077861	0.178534	0.438431	-1.18958
0.8661	286.58	270.08	140.4	10.04	25.31	146.13	1.037753	0.053224	0.137281	3.840974	1.941472
0.8655	73965.04	79458.09	88488.13	71135.95	69059.71	73093.9	1.029241	0.145037	0.27043	1.134192	0.181665
0.8638	108777	107080.2	107653	5063.24	3496.25	953.53	1.325499	1.33E-05	0.003614	34.0071	5.087764

0.8632	27180.82	30142.67	27709.66	1745.09	249.69	1885.42	1.279898	8.31E-05	0.00568	21.91463	4.453822
0.8623	5930.88	27933.21	50209.95	19372.31	8655.53	8945.09	0.527088	0.344165	0.485035	2.273935	1.185191
0.8611	267545.2	236471.8	385313	401283	272499.1	272259.7	0.228898	0.777276	0.843452	0.940054	-0.08919
0.8602	292076.8	458207.7	175851.9	122510.9	125346.8	130885.5	1.136639	0.155709	0.28395	2.445289	1.290005
0.8599	5005.3	4071.52	4018.5	2272.58	2285.33	2851.41	1.290452	0.011997	0.05293	1.767412	0.821638
0.8586	12723.8	10925.37	5340.47	9223.47	16324.57	14945.73	0.686712	0.285002	0.427945	0.715904	-0.48216
0.8581	2995.63	1434.58	2074.01	466.3	745.2	563.54	1.275842	0.069276	0.164027	3.664267	1.873525
0.8529	2281.42	513.08	1707.87	814.92	238.2	12243.68	0.032214	0.532432	0.648469	0.338606	-1.56232
0.8527	66262.4	79806.34	71537.17	79113.73	74326.49	62183.52	0.087365	0.922983	0.945098	1.009193	0.013202
0.8498	511.1	1290.49	1791.67	4439.81	4194.97	3987.91	1.204226	0.008779	0.044309	0.284667	-1.81265
0.8478	60829.47	59018.14	64052.27	58087.54	61508.56	65770.43	0.115037	0.864685	0.904309	0.992088	-0.01146
0.8454	969.19	40.88	637.08	808.22	439.88	799.33	0.507967	0.686501	0.775047	0.804496	-0.31384
0.8453	2246.71	221.63	53.05	170.34	298.92	986.96	0.123291	0.673607	0.763071	1.731462	0.791991
0.844	5248.38	4765.92	2669.15	2884.91	1309.18	1904.51	1.052497	0.090407	0.19823	2.079731	1.056397
0.8431	288168.1	325815.2	272477	486850.8	480498.4	493627.2	1.343137	0.004745	0.03246	0.606759	-0.7208
0.8425	797187.8	718321.8	690037.2	814351.5	755367.6	822001.2	0.865894	0.192059	0.327921	0.922159	-0.11691
0.8417	16600.55	25528.51	14232.85	35707.85	19578.79	17180.42	0.512111	0.480972	0.603673	0.777759	-0.3626
0.8407	35197.1	25207.88	37200.25	28472.05	33550.88	28103.4	0.341328	0.588473	0.695326	1.082982	0.11501
0.8386	11904.01	3979.25	11139.07	4048.18	6950.53	1110	0.806579	0.187115	0.322239	2.231644	1.158107
0.8376	60103.92	70440.9	65858.42	91889.1	87236.77	83811.69	1.290272	0.005093	0.033561	0.746958	-0.4209
0.8365	33261.39	26251.75	30390.91	13422.67	13851.02	11287.49	1.340402	0.007133	0.03937	2.331465	1.221237
0.8316	148647.4	96251.92	309.21	188267.1	179375.5	195246.8	0.717978	0.133464	0.25561	0.435625	-1.19884
0.8312	91962.34	80289.9	88833.53	68676.98	33974.88	31775.13	1.132042	0.061745	0.151804	1.942212	0.957701
0.8257	5669.25	7894.91	7028.25	1158	8977.32	5694.07	0.555105	0.561454	0.673575	1.300897	0.379507
0.821	12693.47	13868.63	4628.29	2794.51	2158.24	1626.29	1.216278	0.103879	0.216966	4.740873	2.245153
0.8184	17489.73	33341.13	31484.5	31378.9	16734.7	17299.83	0.516017	0.461713	0.587622	1.258386	0.331575

0.8166	8396.74	3775.52	6230.05	2405.88	4384.61	2944.79	0.995704	0.150308	0.276803	1.89027	0.918592
0.815	123580.4	37403.95	52508.68	89522.3	92272.32	86665.36	0.621005	0.561892	0.673622	0.795251	-0.33052
0.8132	234255.9	254086.6	262010.5	249867	229867.4	242289.4	0.574553	0.40843	0.540598	1.039236	0.055523
0.8121	64120.99	56065.18	54374.09	26800.92	43936.49	11494.01	1.027282	0.069931	0.165097	2.122793	1.085964
0.812	4001.79	6575.03	7809.47	18975.59	20494.48	18764.29	1.299359	0.00207	0.020844	0.315729	-1.66324
0.8119	126315.7	63885.03	83573.21	21502.88	158043	23894.78	0.633408	0.667387	0.757834	1.345718	0.428377
0.8117	49694.05	127595.3	73014.42	24388.16	24240.81	24178.93	1.241627	0.124437	0.243894	3.437866	1.781513
0.8105	18654.28	8089.67	13073.02	3770.06	90517.27	24126.33	0.333931	0.422749	0.551654	0.336253	-1.57238
0.8088	1146689	1143715	653379.3	1248517	1203095	1286969	0.804217	0.245873	0.389166	0.787407	-0.34482
0.8085	2919515	1951534	2866333	4438722	3911896	3436149	1.125455	0.034523	0.103171	0.656446	-0.60725
0.8078	6422.67	2947.56	399.75	195.49	106.54	2288.92	0.767124	0.304427	0.446951	3.77081	1.914874
0.8042	14220.39	9595.84	25832.81	8228.36	9661.19	9321.85	0.913655	0.261024	0.407231	1.824568	0.867555
0.8035	10157.1	9573.27	7314.43	40825.64	35014.35	43673.08	1.35563	0.003419	0.027425	0.226292	-2.14375
0.8015	11910.75	12835.92	16465.46	21167.16	24828.58	11905.01	0.724481	0.282828	0.426423	0.711772	-0.49051
0.8012	29314.14	69891.73	63211.43	4710.55	15973.13	11276.65	1.206313	0.066027	0.159276	5.081841	2.345351
0.8002	3030.18	1583.18	3845.3	768.29	1072.27	2343.12	0.911201	0.16337	0.293905	2.021823	1.015657
0.8	v	892.28	385.57	627.63	6440.69	495.57	0.323375	0.506535	0.624607	0.373925	-1.41918
0.799	2005.99	2249.5	7700.67	4086.88	977.63	3914.71	0.281965	0.670141	0.759923	1.331537	0.413092
0.7976	47928.9	40049.18	45468.29	59074.96	48606.12	58595.23	1.093321	0.064941	0.157511	0.802558	-0.31732
0.7975	6711.52	30339.33	5948.83	9625.75	9889.99	9251.98	0.137948	0.613587	0.716482	1.49472	0.579875
0.7975	3005.84	18874.43	844.95	573.41	1157.38	57.55	0.934953	0.344332	0.485079	12.70744	3.667601
0.7972	40999.1	55709.19	43863.31	110550.7	129387.2	107794.6	1.335179	0.001928	0.020396	0.404252	-1.30667
0.7962	22115.67	12840.06	23827.68	15079.53	22898.28	19963.28	0.018705	0.949203	0.96329	1.014538	0.020822
0.7962	80840.84	87129.03	89438.36	81259	77565.82	80071.07	1.021777	0.123957	0.243653	1.077491	0.107676
0.7952	23134.98	40172.99	31383.99	48791.59	49337.47	51097.16	1.141381	0.063367	0.154777	0.634553	-0.65619
0.794	25.82	34.35	33.05	49.53	2063.96	12.96	0.529482	0.422599	0.551654	0.043838	-4.51166

0.7937	58133.27	52683.67	46128.93	137390.2	220365.9	125360.1	1.299609	0.066009	0.159276	0.324862	-1.6221
0.79	78162.14	113928.6	114741	155737.1	66063.97	69300.97	0.265954	0.880571	0.915433	1.054035	0.075923
0.7876	32486.89	32415.77	31980.86	283.24	485.2	646.46	1.364101	6.41E-08	0.000279	68.47376	6.097479
0.7875	6938.72	9115.72	9077.45	12363.91	23743.14	20860.07	1.200776	0.084019	0.187622	0.441165	-1.18061
0.7875	358.22	817.89	399.84	624.52	385.18	1310.66	0.482267	0.486153	0.608061	0.679183	-0.55813
0.7874	41608.32	11360.34	32617.81	67585.57	37726.47	32344.97	0.715437	0.290008	0.432778	0.621737	-0.68562
0.7866	57533.19	136186.7	122020.1	114905.6	105836.1	119807.9	0.340658	0.766729	0.83617	0.927148	-0.10913
0.786	61.27	42.8	38.77	131.63	1470.4	1944.35	1.161222	0.171742	0.30469	0.040278	-4.63388
0.7859	93.28	569.26	441.88	68.26	314.88	350.49	0.326198	0.509273	0.627097	1.505418	0.590164
0.785	12301.46	31480.08	18717.96	48.77	12165.32	629.09	0.978491	0.081425	0.183616	4.866357	2.282842
0.7837	13106094	16368658	13456508	5761999	6078620	5947469	1.354809	0.014326	0.058969	2.413484	1.271117
0.7836	77806.09	67657.15	58477.8	25343.74	195.94	1797.79	1.068248	0.005768	0.035765	7.460129	2.8992
0.7836	146669.2	135473	158372.8	27141.57	21300.5	27893.52	1.364766	0.001389	0.016914	5.770768	2.528763
0.7825	32640.08	20353.38	53256.28	23767.3	24603.34	24014.66	0.666469	0.360627	0.500628	1.467836	0.553691
0.7823	11552.93	10278.03	16611.32	12008.01	13390.37	8179.52	0.425588	0.551002	0.665693	1.144868	0.195182
0.7809	6.98	111.41	3.15	1.23	5.11	1059.15	0.099498	0.466017	0.590858	0.11407	-3.13201
0.7807	18904.31	22317.48	21420.24	30783.91	11317.79	15922.48	0.362901	0.819173	0.873691	1.079585	0.110477
0.7791	22066.96	15589.01	20189.65	20078.61	14811.94	15841.86	0.579195	0.39986	0.534331	1.14021	0.1893
0.7784	1143.59	759.54	100.76	249.91	725.64	715.87	0.110439	0.781013	0.845901	1.184738	0.244568
0.7777	102803.8	104667.7	101161.5	75966.93	74478.61	69743.05	1.355072	0.000721	0.013717	1.401676	0.487152
0.7771	1950.88	22.55	1918.8	1149.76	55.05	92.17	0.338379	0.318474	0.460899	3.000995	1.585441
0.7724	49681.29	32291.25	33462.42	20015.99	49269.74	30618.56	0.423706	0.643316	0.738729	1.155455	0.208462
0.7719	19235.96	14586.32	17233.56	6907.3	11045.36	13443.97	1.057955	0.054872	0.139958	1.626157	0.701466
0.7714	108348.1	102140.6	69089.53	55645.91	69084.2	59579.19	1.086082	0.109632	0.225525	1.516897	0.601123
0.7706	2327.18	334.8	2098.13	1154.93	2724.66	1378.39	0.318571	0.845962	0.890712	0.905312	-0.14351
0.7697	8542.38	12150.84	8131.77	1497.19	37.89	1390.62	1.078097	0.012918	0.055476	9.85234	3.300466

0.7681	82361.18	66046.36	69297.2	39468.44	57618.89	52214.18	1.132382	0.036209	0.107151	1.458155	0.544144
0.7677	3706529	3340741	4521045	1911697	1306516	1865302	1.289686	0.011042	0.050451	2.275653	1.18628
0.7668	59399.02	54377.96	59778.59	30036.77	32645.07	31153.76	1.362027	0.001276	0.016332	1.849571	0.88719
0.7655	9845.76	38369.56	26161.07	55237.63	44128.24	51516.44	0.985456	0.075542	0.174412	0.492943	-1.02051
0.765	7586.31	6876.77	8487.83	6020.24	5652.43	6246.21	1.202786	0.055358	0.14054	1.280823	0.357071
0.765	85619.78	75290.09	81827.56	29647.54	28556.25	29649.31	1.368754	0.003036	0.025806	2.762992	1.466231
0.7641	75471.33	72793.31	78681.29	68462.7	54146.87	58358.41	1.162163	0.053602	0.137774	1.254067	0.326614
0.764	34761.09	36109.63	36576.92	26145.51	24790.94	24050.09	1.356008	0.000208	0.008701	1.432892	0.51893
0.7633	66559.6	51507.69	67591.16	17958.58	17445.55	17060.49	1.359338	0.013261	0.056224	3.538736	1.823234
0.763	74445.53	180648.4	73366.19	104543.1	94523.25	94629.06	0.040794	0.776043	0.842953	1.11837	0.161398
0.7624	18048.15	8751.2	11094.29	5302.98	1523.08	4952.06	1.110873	0.072324	0.169221	3.217291	1.685846
0.7619	26749.65	21471.57	23626.63	17919.24	18662.73	5018.08	0.857791	0.139625	0.263735	1.72711	0.78836
0.7593	482.43	276.51	351.06	1435.9	1605.42	705.51	1.221028	0.079925	0.181092	0.29625	-1.75511
0.7591	12299.78	5388.32	3287.93	12837.24	11653.05	14189.38	0.959737	0.155101	0.283237	0.542301	-0.88283
0.758	3246.73	2906.93	2412.1	2729.39	3020.44	3415.2	0.428872	0.559763	0.673086	0.934613	-0.09756
0.7578	2467.52	2480.01	4622.66	849.67	57.25	345.8	1.145305	0.049572	0.130967	7.639528	2.933484
0.7576	3058.07	942.62	3145.56	3523.29	1229.02	2881.53	0.141343	0.877921	0.913986	0.936128	-0.09522
0.7557	2031.5	1505.6	1546.35	1730.21	1814.28	393.02	0.551604	0.502317	0.620987	1.291032	0.368524
0.7554	8754.06	10552.02	10967.13	2275.45	9613.27	9355.94	0.664599	0.336908	0.477732	1.42498	0.510941
0.7554	9981.51	7506.49	6723.04	3931.7	101.69	4455.03	0.827807	0.04117	0.116306	2.852243	1.512097
0.7531	228638.2	310783.2	232666.2	129423.1	134603	100898.1	1.292054	0.024567	0.081963	2.115748	1.081168
0.7527	47514.11	47294.07	60239.22	19771.17	16768.1	32141.5	1.228917	0.010822	0.049761	2.257508	1.174731
0.7521	43875.55	38072.79	33929.68	59936.97	65201.61	56215.13	1.283778	0.0051	0.033561	0.638961	-0.6462
0.7518	5410.92	6297.08	10881.9	5380.3	712.34	4232.52	0.842495	0.13934	0.263735	2.18785	1.129514
0.7517	16121.72	19271.64	32524.11	268371.6	305788.8	252064.4	1.352874	0.001951	0.020396	0.082202	-3.60468
0.7516	1767.53	1072.05	1038.11	1550.42	1644.77	770.84	0.000483	0.93965	0.956994	0.977726	-0.0325
0.7514	19138.09	13945.94	26027.02	1817.62	3834.99	7225.71	1.211221	0.031909	0.0978	4.589966	2.198483

0.7513	48552.3	25646.13	30580.75	58684.7	74126.94	61105.21	1.163055	0.029828	0.09327	0.54033	-0.88809
0.7511	70525.35	34888.75	46062.84	21058.52	20584.43	20394.8	1.236521	0.105226	0.218886	2.44169	1.28788
0.7497	1542.96	2798.7	565.96	1517.65	4326.33	1579.09	0.52864	0.503611	0.62149	0.661131	-0.59699
0.7489	67590.85	54921.73	65891.04	58032.55	56136.93	60044.08	0.658258	0.356151	0.497078	1.081452	0.11297
0.7489	6069.32	7970.6	1219.27	13550.41	4758.03	1985.42	0.21707	0.703069	0.787526	0.751912	-0.41136
0.7484	27438.66	19146.7	22590.1	2698.9	5082.18	7377.06	1.277642	0.006295	0.037334	4.563585	2.190168
0.7484	32902.46	36643.14	88829.55	362836	355203.1	364800.8	1.310754	0.002792	0.024783	0.146259	-2.7734
0.748	29764.92	23246.19	26939.82	7396.93	12789.65	11505.76	1.280646	0.003191	0.02649	2.522721	1.334981
0.7475	166407.5	144159.6	150598.1	61046.92	74847.69	71901.27	1.348828	0.00097	0.014975	2.219318	1.150117
0.7471	6970.42	8565.81	9016.76	2381.7	5399.93	6132.38	0.990867	0.07068	0.166267	1.764624	0.81936
0.747	36945.72	13536.04	42382.79	34083.07	30837.09	32633.79	0.297355	0.876431	0.91287	0.95193	-0.07107
0.7467	34.41	150.28	38.89	32.41	252.76	27.65	0.032183	0.745034	0.820516	0.714724	-0.48454
0.7458	574.54	358.74	227.03	289.69	411.8	143.64	0.519006	0.459336	0.585794	1.372937	0.457265
0.7456	3557.93	5371.48	1772.21	21541.76	11654.32	14799.45	1.24172	0.038695	0.112091	0.222971	-2.16507
0.7447	461.86	977.93	120.21	789.46	1055.39	613.38	0.698987	0.364177	0.503153	0.634603	-0.65607
0.7443	16154.4	19491.71	19178.92	26676.6	23756.02	23982.72	1.246923	0.010382	0.048879	0.736744	-0.44077
0.7442	584.27	1772.21	2787.26	322.28	205.65	351.93	1.170792	0.154576	0.282628	5.846089	2.547472
0.7442	192.96	776.71	340.63	32.12	208.07	243.8	0.822536	0.252544	0.397702	2.707287	1.436848
0.7429	341.46	258.75	426.15	845.76	76.78	112.93	0.466229	0.991517	0.993446	0.991202	-0.01275
0.7425	7553.24	6080.43	9384.43	1996.54	1577.18	2517.23	1.323421	0.020892	0.074391	3.779066	1.91803
0.7425	1375.46	449.61	1067	1759.31	1609.12	188.25	0.061517	0.722672	0.803604	0.813138	-0.29843
0.7423	54353.12	54808	68988.1	13426.04	27311.46	36728.61	1.135045	0.019091	0.070048	2.299705	1.201449
0.7409	6.54	801.8	77.68	18.98	107.32	38.72	0.250419	0.44428	0.57178	5.369167	2.424698
0.7406	34524.43	22788.7	37104.82	1601.31	2428.26	2407.92	1.357115	0.021487	0.075726	14.66689	3.874491
0.7404	1839.22	2355.04	946.39	3729.57	2192.36	2943.25	0.942829	0.109911	0.225885	0.57987	-0.7862
0.7402	5364.4	7648.37	12349.98	19549.91	26152.74	14903.02	1.153255	0.047778	0.127926	0.418488	-1.25674

0.7394	49919.14	39324.99	62123.72	45053.01	75127.22	49381.7	0.339946	0.627926	0.727221	0.892699	-0.16375
0.7389	999.66	1571.02	1180.77	9855.7	8481.23	1141.16	0.93333	0.191779	0.327572	0.192598	-2.37633
0.7376	7550.62	1664241	3358.04	11930.52	5817.14	9036.15	0.462533	0.425076	0.553083	62.54338	5.966785
0.7368	8838.45	3447.98	4969.1	19241.12	20737.93	17191.04	1.257146	0.003943	0.0292	0.301828	-1.7282
0.7363	12776.89	15910.31	15250.37	4307.3	2433.47	4033.43	1.326075	0.001425	0.01714	4.078035	2.027874
0.7354	1830.93	922.48	23974.25	1048.24	1721.63	480.86	0.7119	0.408209	0.540517	8.222049	3.039498
0.7353	323.34	10572.04	8123.43	11392.24	13978.33	1072.75	0.290523	0.648543	0.74246	0.719229	-0.47548
0.734	2671	5528.93	6358.24	1517.18	2215.8	1510.94	1.171864	0.102982	0.21553	2.7762	1.473111
0.7337	1760.86	915.38	972.81	9772.48	1685.11	1229.95	0.730338	0.391028	0.527593	0.287609	-1.79782
0.7317	13246.97	12111.56	12727.04	156.88	347.05	10627.01	0.994524	0.120452	0.23897	3.421595	1.774669
0.7317	4771.93	369.43	6072.33	753.63	6269.09	7196.79	0.226262	0.724978	0.804936	0.788613	-0.34261
0.7316	109754.8	97747.73	111766.6	77229.34	71210.13	79907.74	1.306381	0.00757	0.040387	1.398174	0.483544
0.7305	10550.41	3902.68	5235.38	13092.38	16722.42	13749.55	1.129299	0.039208	0.112605	0.45194	-1.1458
0.7301	3676.44	2365.76	2824.31	22679.87	19849.84	9146.27	1.289887	0.073014	0.170287	0.171579	-2.54306
0.7299	2659.45	1730.24	2793.58	17796.68	18020.76	20959.43	1.356872	0.001765	0.019432	0.126518	-2.98259
0.7297	513.98	1854.32	1686.45	3060.69	2506.18	2051.38	0.909927	0.08961	0.196799	0.532242	-0.90985
0.7288	11872.44	5489.36	18369.46	395.86	1300.2	920.02	1.282268	0.096477	0.206858	13.65832	3.771708
0.728	10.37	136.14	17.25	1308.98	74.82	391.98	1.003905	0.282926	0.426423	0.092219	-3.4388
0.7278	207868	205846.4	200976.7	72034.5	87983.92	90147.36	1.355699	0.000788	0.014349	2.457135	1.296977
0.7267	7389.85	11217.06	8442.71	5932.05	4896.93	7513.62	1.020959	0.112033	0.228523	1.474688	0.56041
0.7262	1290185	1066778	1182913	1340739	1172480	1252784	0.585673	0.40722	0.540192	0.939955	-0.08934
0.7256	40562.92	18481.78	39703.84	11174.48	35974.69	22539.81	0.577497	0.394996	0.531088	1.416989	0.502829
0.7253	643.77	1042.04	1871.41	1175.08	2889.91	1510.27	0.682696	0.357641	0.498387	0.638037	-0.64829
0.7249	9128.03	5726.57	8124.01	8082.94	8946.64	7336.2	0.326816	0.706989	0.791007	0.943069	-0.08456
0.7238	911	1261.86	698.45	4327.71	6629.87	5490.96	1.335454	0.016374	0.063333	0.174563	-2.51818
0.7238	7162.25	6321.13	11339.83	37227.09	21440.08	23911.69	1.271113	0.048412	0.129148	0.3006	-1.73408

0.7237	20818	46923.75	24541.9	3691.21	29898.74	8468.42	0.872055	0.217951	0.35795	2.19418	1.133682
0.723	195291.6	151917.6	159507.5	174680.2	143501.9	151440.5	0.487444	0.495484	0.614282	1.078987	0.109678
0.7225	18375.38	20053.82	26753.33	1618.58	2251.97	26689.41	0.896566	0.295001	0.43802	2.132939	1.092843
0.7217	44613.32	41396.93	41377.57	108717.3	123243.1	119853.8	1.367612	0.002182	0.021417	0.362088	-1.46559
0.7215	7858.29	7312.11	8558.05	21031.21	19534.48	32631.26	1.31438	0.056588	0.143079	0.324173	-1.62517
0.7214	601.44	888.28	697.21	1919.27	760.91	669.94	0.571514	0.437982	0.565657	0.652792	-0.61531
0.7205	4787.42	1058.01	4204.38	3762.69	5785.74	8700.91	0.767883	0.215212	0.354941	0.550694	-0.86068
0.7204	1391.21	3116.02	4472.67	16073.51	339.76	16010.77	0.253775	0.272404	0.417289	0.276952	-1.85229
0.7203	54929.18	121890.1	125510.3	72382.27	69773.39	59039.67	0.726	0.277972	0.421735	1.502667	0.587525
0.7195	175.1	172.02	35.05	237.98	104.9	68.94	0.172375	0.893267	0.924668	0.928003	-0.1078
0.7185	3966.02	2033.93	4681.91	10157.81	6969.8	6977.46	1.143832	0.03135	0.096576	0.443137	-1.17417
0.7177	6803.84	2917.16	6142.36	1415.39	1324.95	1471.27	1.263388	0.083544	0.187137	3.766579	1.913255
0.7177	49.82	67.26	3469.57	72.77	2782.96	2284.99	0.441157	0.733439	0.812322	0.697694	-0.51933
0.7172	175.77	3904.72	363.22	956.85	1201.28	1265.43	0.409714	0.80583	0.863988	1.297979	0.376267
0.7171	3281.62	2959.69	3429.09	4995.22	3253.38	4961.73	0.967046	0.170846	0.303224	0.732033	-0.45002
0.7168	15117.85	24546.46	6851.43	202281.1	1487.54	13622.34	0.073597	0.473511	0.597047	0.213973	-2.2245
0.7168	218849	165922.7	236100.2	117452.7	91155.67	119048.5	1.259608	0.029075	0.091962	1.894884	0.92211
0.7164	4384.92	448.08	1310.94	708.92	418.38	683.58	0.725258	0.350092	0.491008	3.392792	1.762473
0.7164	8767.69	8764.94	3460.19	6741.69	8261.52	5923.94	0.152603	0.991623	0.993446	1.003138	0.00452
0.7158	38983.34	34137.93	43509.43	40541.93	35551.46	37951.91	0.163476	0.796615	0.857478	1.02267	0.032341
0.7155	130.1	76.18	135.98	315.17	251.26	322.37	1.268766	0.003856	0.029026	0.385081	-1.37677
0.7146	53240.49	387994	43652.02	85613.3	81956.29	363693.4	0.274573	0.921332	0.944828	0.912705	-0.13178
0.7145	127038.4	122546.5	149458.9	125016.8	124713.7	117361.9	0.725839	0.328656	0.470192	1.087039	0.120404
0.7142	176758.6	101737.8	38404.85	215874.6	264428.9	177263	0.957349	0.086153	0.191309	0.48193	-1.0531
0.7141	1448.06	2440.38	659.37	1466.23	1387.97	610.09	0.296112	0.579026	0.687544	1.312768	0.392612
0.713	696.87	279.92	1802.82	496.48	2582.14	1143.54	0.431679	0.566898	0.67813	0.658338	-0.6031

0.7127	234.93	528.98	178.08	125.04	421.86	24.13	0.646968	0.486832	0.608061	1.649633	0.722145
0.7127	10588.4	10636.92	15096.47	3726.11	5870.74	10597.51	0.981396	0.105966	0.219746	1.798611	0.846883
0.7126	11870.66	9529.86	8208.96	142.3	181.61	3383.76	1.143881	0.004702	0.032349	7.986007	2.997474
0.7122	11205.95	9318.21	10138.09	9109.6	14497.04	12649.32	0.629112	0.361475	0.500689	0.845716	-0.24175
0.7118	187859.1	157564.5	5048.94	228868.7	174674.9	158.68	0.267171	0.852425	0.896002	0.868146	-0.20399
0.7115	18191.78	14335.88	17332.17	13877.49	15037.71	15881.02	0.728277	0.288388	0.431386	1.113037	0.154501
0.7115	4838.51	5401.12	5488.65	14530.07	14939.57	12676	1.361154	0.003642	0.028249	0.373189	-1.42202
0.7113	223.4	10612.58	10194.36	6931.57	1306.87	31.4	0.468902	0.358412	0.498984	2.543017	1.346541
0.7107	883.35	488.98	296.64	993.82	569.81	1346.68	0.815329	0.222424	0.362785	0.573468	-0.80221
0.7106	266.74	289.05	2368.94	62.77	169.83	352.59	0.818378	0.379532	0.516757	4.997915	2.321326
0.7103	174002.4	191256.8	164002.7	195699.7	191065.2	162701.9	0.328912	0.634115	0.731052	0.963228	-0.05405
0.7098	29707.77	38130.3	31094.23	52364.35	46500.8	52420.16	1.273462	0.007244	0.039577	0.653945	-0.61276
0.7097	3112.35	958.1	3034.6	2156.41	861.29	1086.89	0.638949	0.301075	0.443974	1.731001	0.791607
0.7084	8605.55	5471.39	14377.14	15173.57	5686.1	4660.21	0.256724	0.829639	0.880492	1.114977	0.157014
0.7083	23.25	210.96	531.44	1074.72	125.17	30.92	0.074878	0.701768	0.786504	0.62207	-0.68485
0.7074	56169.51	75372.02	89954.23	51321.89	50750.43	81770.94	0.578242	0.4258	0.553485	1.204808	0.268803
0.7052	962882.1	1276241	994958.9	569894.1	585363.8	452037.9	1.29725	0.019641	0.07111	2.012126	1.008721
0.7048	64933.45	79625.98	61008.5	90170.66	102593.4	90413.88	1.198319	0.024564	0.081963	0.725932	-0.46209
0.7037	13174.29	11020.49	10511.15	1034.8	6697.06	4487.19	1.029136	0.027945	0.089502	2.840313	1.50605
0.7033	2794.59	301.22	5370.58	2022.22	2982.33	1210.57	0.119299	0.667797	0.757836	1.362225	0.445965
0.7028	2538.22	1059.16	497.47	9496.14	7986.26	6855.13	1.236787	0.002764	0.02464	0.168252	-2.5713
0.7027	21384.13	8928.24	11110.78	5853.72	4740.72	7502.66	1.092439	0.175675	0.309651	2.288939	1.194679
0.702	6529.11	4299.52	6953.31	32093.79	29703.17	26091.11	1.34581	0.001548	0.017853	0.202325	-2.30525
0.7004	102158.6	78570.72	91088.11	64196.34	53119.75	55512.28	1.264904	0.02381	0.080475	1.572759	0.653298
0.7003	1301.15	8300.44	12058.68	78173.73	1700.96	71135.27	0.610378	0.21775	0.357909	0.143436	-2.80152
0.6997	55613.35	44855.87	48814.78	36600.82	1571.76	32605.58	0.767408	0.133166	0.255268	2.109182	1.076683
0.6996	9875.7	7627.94	17042.35	107709.6	98734.01	9198.88	0.894024	0.194017	0.330343	0.1602	-2.64205

0.6992	116394	96914.62	111050	82329	67155.29	80735.14	1.230921	0.015197	0.060802	1.408911	0.49458
0.6992	1056099	1002070	992440.4	783286.3	814499.9	972729.4	1.075713	0.099956	0.211304	1.18677	0.24704
0.6992	24078.31	20.48	1093.82	280.11	18756.11	1906.2	0.276074	0.892781	0.924598	1.202946	0.266572
0.6988	202716.3	172410.5	187244.9	157284.7	129364	165005.6	1.077167	0.059494	0.148191	1.245138	0.316305
0.6985	495098.5	440110.2	482981.8	348013.1	325110.1	410035.6	1.193151	0.02735	0.08828	1.30931	0.388807
0.6984	7348.06	26205.14	30045.77	6095.76	26611.61	5626.92	0.569887	0.440829	0.568038	1.659062	0.730368
0.6982	25628.36	23250.68	26547.18	7766.58	6441.03	4047.52	1.325073	0.000215	0.008842	4.131782	2.046764
0.698	2455.17	3043.42	2333.25	6770.44	3255.29	4221.19	1.060281	0.173677	0.307208	0.549722	-0.86323
0.6979	194.1	7116.98	3818.25	25200.79	20071.25	21563.46	1.03241	0.002326	0.022458	0.166518	-2.58625
0.6977	10517.5	5383.09	8909.47	11726.67	2830.57	944.55	0.717852	0.462278	0.58817	1.600464	0.67849
0.6976	22484.62	17961.74	18906.7	21458.58	31349.61	31988.97	1.047866	0.116396	0.233919	0.699942	-0.51469
0.6971	9809.45	6960.35	7821.05	20387.19	14534.29	15504.01	1.273259	0.025881	0.084254	0.487667	-1.03603
0.697	48484.53	30413.86	44032.71	15626.28	16292.59	13335.8	1.309773	0.038264	0.111568	2.716429	1.441711
0.6967	2397.96	2255.62	3372.17	1061.21	53.06	418.56	1.069324	0.009827	0.04728	5.235904	2.388439
0.6965	927.88	1398.44	1184.4	348.11	983.4	837.39	0.865677	0.138045	0.262195	1.618664	0.694803
0.6964	4913.86	3047.67	5153.92	71924.84	42566.17	69432.2	1.35099	0.02569	0.083901	0.071309	-3.80976
0.6962	90657.64	73661.16	83151.18	1506.07	2375.3	2994.48	1.363562	0.003527	0.027803	35.99118	5.169572
0.6958	41114.88	41999.56	40240.22	29497.98	28531.19	28566.02	1.36703	0.000123	0.006457	1.424498	0.510453
0.6957	205.46	1.12	70.11	682.55	695.65	325.52	0.945557	0.040634	0.115316	0.162403	-2.62235
0.6953	77907.4	90446.94	92403.3	18046.25	25398.72	18564.46	1.353841	0.000975	0.014975	4.205129	2.07215
0.6943	85392.81	109553.6	82845.47	100027.4	135475.6	99750.07	0.778697	0.266391	0.412357	0.828604	-0.27125
0.694	765.1	1493.83	887.38	104.54	511.11	2152.46	0.513705	0.863967	0.903992	1.136628	0.18476
0.6929	5145.04	9625.68	4139.41	4271.53	8810.42	9276.97	0.333545	0.64659	0.740927	0.845753	-0.24169
0.6929	5275.97	5580.12	4900.56	1626.46	672.14	1457.18	1.286247	0.000714	0.013717	4.195307	2.068776
0.6928	27817.71	28620.46	24009.45	34673.34	43185.78	49825.69	1.217562	0.05802	0.145613	0.630048	-0.66647
0.6924	45033.19	53073.1	31941.39	88054.49	42299.92	86767.42	0.886623	0.184032	0.31927	0.598962	-0.73946

0.692	32957.27	31756.59	32362.72	23322.84	24158.72	8278.89	0.94113	0.115715	0.233303	1.740958	0.799881
0.6916	7224.79	10962.8	3818.2	5701.52	4553.89	4113.67	0.651284	0.342121	0.483249	1.531468	0.614915
0.6916	7538.1	13267.18	19858.96	4945.7	9463.65	13029.64	0.60599	0.367588	0.505941	1.481987	0.567533
0.6914	83135.67	71822.24	95394.31	1337.39	1619.81	5575.29	1.330607	0.0055	0.034788	29.34105	4.874849
0.6904	6944.01	3936.15	1869.41	9922.43	19567.72	15647.27	1.162449	0.041504	0.117021	0.282461	-1.82388
0.6903	13942.9	17159.43	17637.17	15794.96	15388.89	11790.87	0.654272	0.327427	0.469255	1.134144	0.181603
0.6897	17103.52	14955.22	18521.96	74.58	7798.09	246.62	1.06923	0.019218	0.070294	6.229695	2.639162
0.6884	76879.8	67678.05	59074.44	39839.26	38599.64	35584.92	1.318628	0.023072	0.078816	1.785875	0.836631
0.688	30082.86	27123.83	27705.58	14982.06	13800.95	15290.14	1.360395	0.000976	0.014975	1.926621	0.946073
0.6879	1533.78	6150.41	7327.73	2231.27	2225.85	1794.2	0.775541	0.239851	0.38241	2.4014	1.263876
0.6878	6697.39	6966.29	5816.09	8920.78	8534.75	8074.56	1.250318	0.011669	0.052168	0.763012	-0.39022
0.6878	6701708	5662866	6333839	1282731	1462505	1422116	1.367955	0.003096	0.0261	4.486881	2.165713
0.6877	612.32	648.84	719.23	2104.16	791.14	1213.24	1.029498	0.207583	0.346555	0.482018	-1.05284
0.6874	145267.1	123147.4	134935.8	120515.7	125003.6	109358.7	0.981741	0.116996	0.234503	1.136588	0.18471
0.6868	12750.71	13631.84	35939.76	13234.95	15030.29	23238.1	0.182664	0.693131	0.779706	1.210063	0.275083
0.6866	12862.17	11912.24	12338.14	262.76	2004.04	440.37	1.27162	0.000393	0.010355	13.70898	3.77705
0.6864	2167	716.67	5223.99	1650.96	1088.65	1931.75	0.316173	0.480853	0.603673	1.73561	0.795443
0.6847	10760.15	2898.31	7939.16	2183.97	149.93	802.64	1.089459	0.107664	0.222527	6.88581	2.783626
0.6847	2414.52	1965.55	1416.5	1654	1857.11	1847.33	0.237087	0.66653	0.757439	1.081764	0.113386
0.6845	77.55	353.26	146.13	219.52	226.46	620.76	0.716443	0.365744	0.504219	0.540844	-0.88672
0.6845	63330.49	180211.5	63202.04	98221.92	60615.94	70385.66	0.320721	0.581141	0.689321	1.338187	0.42028
0.6844	147029.1	152910.8	129103.1	157670.5	133518.9	126985.8	0.223937	0.774439	0.842107	1.025989	0.037015
0.6843	2134.3	1620.25	4419.11	2223.49	3532.5	1383.49	0.192682	0.763515	0.833499	1.144854	0.195163
0.6842	2254.65	5554.24	4777.82	1327.18	2252.54	1321.79	1.10973	0.113202	0.229605	2.567925	1.360603
0.6831	179.69	199.03	326.79	3103.24	738.54	545.15	1.108624	0.273975	0.418597	0.160821	-2.63647
0.6829	2448.5	114.28	1614.9	2663.79	5541.27	1842.21	0.767463	0.22482	0.364986	0.415803	-1.26603
0.6827	297271.3	505502.8	230888.6	713354.4	757603.6	593800.4	1.149635	0.032877	0.099869	0.500622	-0.99821

0.6819	2414.57	953.2	2744.83	18460.28	11457.44	11743.18	1.287651	0.029634	0.093065	0.146723	-2.76884
0.6818	58856.62	66146.54	58689.34	81218.39	84118.39	72017.63	1.237624	0.019846	0.071615	0.773917	-0.36975
0.6807	11104.84	13976.63	11844.17	2410	6378.97	4694.54	1.203766	0.006892	0.038918	2.738578	1.453427
0.6805	60927.62	59454.27	61313.88	844.02	488.29	258.48	1.35967	2.39E-05	0.003778	114.2173	6.835638
0.6794	166799.7	114255.1	153106.4	901850.1	983527.3	900370	1.36213	9.47E-05	0.005985	0.155851	-2.68176
0.6788	194933.9	198230.3	191576.1	173884.5	107005.3	110167	1.106489	0.09637	0.20673	1.495283	0.580418
0.6786	80755.01	103301.9	87154.35	40628.86	40835.64	41291.34	1.349997	0.017818	0.066896	2.209356	1.143626
0.6786	627.55	824.45	610.53	96.97	719	122.83	0.940164	0.199161	0.33662	2.196986	1.135525
0.6784	34232.72	29350.54	22826.11	173.49	16956.83	17930.7	0.752531	0.077205	0.177311	2.464542	1.30132
0.6782	14871.61	10578.21	10484.33	10348.8	7881.76	7663.17	1.007344	0.133708	0.255853	1.387755	0.472753
0.6774	4553.92	8168.95	3871.41	16880.46	15145.27	13940.08	1.258414	0.005824	0.03591	0.361014	-1.46988
0.6774	57593.88	66618.48	33363.25	23578.88	22042.36	20594.02	1.218756	0.090791	0.198774	2.379748	1.250809
0.6768	107690.2	118046.7	94200.83	17281.13	17331.26	8791.87	1.338003	0.002018	0.020676	7.371114	2.881883
0.6767	17383.92	12995.18	17743.88	152719.3	177651.4	145823.2	1.365862	0.00382	0.02898	0.101058	-3.30675
0.6764	240737.7	177449.7	227037.6	47674.37	99569.11	87283.43	1.243623	0.005904	0.036092	2.751176	1.460049
0.676	4708.16	1871.36	2957.7	7845.91	2457.08	7504.55	0.719915	0.25246	0.397702	0.535572	-0.90085
0.6753	15140.84	8279.26	8499.95	1779.65	1078.31	5834.94	1.119612	0.053922	0.138263	3.671968	1.876553
0.6749	838.83	1787.4	2590.41	806.74	1430.54	1269.94	0.546564	0.381103	0.518	1.4874	0.572793
0.6746	134.48	15.89	281.76	44.18	46.99	85.14	0.30851	0.383063	0.520016	2.450967	1.293351
0.6745	1534.24	601.36	981.33	66.35	30.87	239.26	1.216229	0.06871	0.16322	9.263344	3.211533
0.6744	11982.62	12056.92	8831.4	7984.26	23699.13	24386.74	0.673658	0.283903	0.426882	0.586247	-0.77042
0.6743	20630.21	14707.71	30020.48	5531.84	13182.71	26277.48	0.636716	0.421926	0.551478	1.452666	0.538703
0.6742	168435.9	167561.5	189766	349541.8	315236	356975.7	1.353694	0.001202	0.016008	0.51457	-0.95856
0.6738	5007.37	14888.97	16329.81	1760.31	2293.05	53577.28	0.330072	0.720932	0.802283	0.628592	-0.6698
0.6735	55447.29	58783.03	76429.71	93131.8	58786.3	59447.04	0.326491	0.632155	0.730532	0.902041	-0.14874
0.6734	23521.08	28288.41	21799.51	27720.92	41389.23	30075.92	0.970573	0.169874	0.301867	0.74213	-0.43026

0.673	329.88	2339.71	1670.04	4340.73	633.65	1850.8	0.366971	0.550902	0.665693	0.635826	-0.6533
0.673	68799.29	48189.58	56527.94	12544.11	15196.25	12285.92	1.353695	0.01582	0.062294	4.335072	2.116056
0.6723	184939	162906.7	170232.7	39478.1	34500.12	44413.72	1.364543	0.000546	0.012468	4.37596	2.1296
0.6717	3122.08	3715.4	2597.12	1658.96	2332.43	3563.26	0.644968	0.398291	0.533216	1.248847	0.320596
0.6712	40253.07	59277.26	42785.94	469796.8	417731.2	385009.8	1.362923	0.002824	0.024921	0.111837	-3.16054
0.6706	435.26	58.05	496.02	944.77	1225.23	199.34	0.653144	0.270709	0.415793	0.417555	-1.25996
0.6704	119.9	63.25	78.53	292.65	257.53	267.89	1.305426	0.001693	0.019065	0.319875	-1.64442
0.6704	397.58	220.2	70.13	554.82	1102.79	644.79	1.07678	0.066193	0.159588	0.29878	-1.74285
0.6698	3089.09	3409.02	3417.71	7039.11	7688.25	4737.48	1.243917	0.068787	0.163312	0.509422	-0.97307
0.6693	12990.92	9102.02	13822.82	24515.26	22048.92	23220.27	1.277287	0.006713	0.038427	0.514667	-0.95829
0.6678	14811.17	27948.36	17345.08	1407.23	1908.66	747.26	1.333272	0.042544	0.118649	14.79261	3.886805
0.6672	19.24	3243.68	1005.86	23.33	1306.34	76.13	0.371417	0.432897	0.560106	3.036549	1.602432
0.6671	37535.95	32761.12	35944.94	15066.99	15480.71	16431.07	1.363572	0.002893	0.025269	2.26149	1.177274
0.667	492.1	1112.89	91.99	62.46	79.18	565.7	0.588181	0.400553	0.534797	2.399101	1.262494
0.6668	27907.28	28853.4	29798.31	15119.69	15180.32	12577.06	1.350568	0.000375	0.010037	2.018771	1.013477
0.6668	3965.38	5468.05	4450.3	1264.72	451.03	1056.32	1.278532	0.004633	0.032268	5.008434	2.32436
0.6666	42569.49	35963.56	43048.71	23379.54	18084.34	21568.44	1.318883	0.003374	0.027336	1.92888	0.947763
0.6665	1662.15	1973.06	1097.1	1969.25	612.25	1681.4	0.315187	0.766562	0.83617	1.110115	0.150709
0.6656	6951.26	5407.19	8018.42	13291.5	14969.27	13387.94	1.301888	0.002358	0.022642	0.489256	-1.03134
0.6653	7993.34	8295.63	9878.87	8092.51	5560.27	6856.75	0.967295	0.117562	0.235178	1.275887	0.3515
0.6653	19116.77	15922.36	18335.35	15583.93	15581.28	16142	0.990121	0.165942	0.296601	1.128253	0.17409
0.6652	34913.19	39183.2	39823.69	8907.26	1742.26	275	1.158135	0.001142	0.015971	10.42793	3.38238
0.6646	95948.34	14765.87	47864.97	12799.61	10864.61	49267.28	0.63837	0.361969	0.501055	2.174358	1.120589
0.6644	1738.56	3406.34	4785.21	3905.84	16436.33	3431.4	0.693449	0.39224	0.528614	0.417695	-1.25948
0.6642	8111.2	6273.86	5132.27	5120.13	2943.17	6888.21	0.656249	0.352124	0.493064	1.305375	0.384465
0.6629	1231.44	847.1	1619.17	228.25	160.12	832.94	1.072112	0.055755	0.141218	3.027659	1.598203

0.6628	8547.69	9712.43	11675.52	6567.02	1733.1	3009.59	1.119057	0.029575	0.092946	2.646897	1.404302
0.6626	17892.55	16958.09	27438.92	2191.14	793.9	3857.51	1.270423	0.025008	0.082772	9.103267	3.186384
0.6623	10.81	145.6	100.67	1790.49	12.75	106.43	0.36425	0.440982	0.568038	0.13462	-2.89303
0.6619	3863.13	2096.87	2791.01	6713.78	14445.35	5309.11	1.139851	0.169507	0.301584	0.330623	-1.59674
0.6612	2847.56	3304.03	2558.89	368.32	113.54	1121.93	1.156269	0.004345	0.031089	5.431185	2.441267
0.6612	398300.9	351574.3	413926.6	187516.8	169624.1	180078.6	1.358219	0.005105	0.033561	2.166343	1.115262
0.6601	29398.77	24394.5	31082.45	36.61	1632.28	9301.32	1.006239	0.003181	0.02649	7.736928	2.951761
0.66	15543.7	20336.23	20408.32	7251.69	16712.26	16397.07	0.76951	0.225947	0.366284	1.394619	0.479871
0.6598	788.85	175.38	2482.44	66.13	333.55	202.22	0.85825	0.302485	0.4453	5.726317	2.517607
0.6591	53566.77	22490.25	58722.38	30417.13	23778.83	30492.91	0.718313	0.276179	0.420344	1.591465	0.670356
0.6585	23082.86	15653.05	24466.6	790.12	784.42	2391.32	1.324183	0.01585	0.06235	15.93665	3.994276
0.6581	9913.01	24441.69	36222.36	12359.4	12973.84	14824.96	0.673754	0.313828	0.45636	1.757476	0.813505
0.6575	47282.4	39680.06	46150.3	53246.44	53038.3	47959.86	1.037047	0.080123	0.181244	0.862998	-0.21257
0.6562	27235.82	18009.77	21198.35	3371.88	6893.24	5313.44	1.304778	0.015232	0.060802	4.265089	2.092576
0.6562	2649.61	1173.78	3816.97	2899.8	2262.68	3657.39	0.409209	0.679848	0.768532	0.866267	-0.20712
0.6561	4152.63	1377.63	6783.25	2607.62	3544.47	2175.5	0.298969	0.487523	0.608392	1.47864	0.564271
0.6556	2179.21	1180.81	1040.31	3912.52	4503.36	4097.14	1.265268	0.007312	0.039741	0.35166	-1.50775
0.655	206223.3	212267.3	206517.2	932843.9	996512.2	1243286	1.365118	0.012173	0.053491	0.196999	-2.34374
0.6549	29819.33	16158.09	27996.07	17206.58	9848.27	9939.51	1.08673	0.082998	0.186392	1.999588	0.999703
0.6544	182572.3	176466.1	170221.9	282441.4	188220.6	173481.5	0.678561	0.378421	0.515641	0.82165	-0.2834
0.6543	369.51	3763.86	1960.02	6397.17	748.31	739.04	0.058198	0.79689	0.857478	0.77283	-0.37178
0.6535	6723.03	6270.92	9429.82	3901.66	3734.47	6379.22	1.031259	0.099673	0.211012	1.599944	0.678021
0.6535	141674.3	106300.2	123344	316665.1	520046	324864.3	1.306382	0.055119	0.140178	0.319668	-1.64535
0.6533	1744.99	898.58	2221.27	2197.69	2151.86	2723.64	0.84143	0.188571	0.323655	0.687786	-0.53997
0.6533	31251.8	35844.01	36339.32	12879.9	18954.24	22241.61	1.225699	0.011567	0.051928	1.912782	0.935673
0.6532	107157.7	50809.79	111388.2	160065.4	150124.6	335707.5	1.050401	0.163732	0.294312	0.417025	-1.26179

0.6529	35926.63	27827.2	31871.48	3232.43	507.44	1725.57	1.294945	0.002987	0.025612	17.49636	4.128983
0.6525	341.62	156.77	423.4	106.33	274.67	350.65	0.369771	0.585433	0.692511	1.259878	0.333284
0.6521	2275.74	1683.7	1042.05	1142.82	1898.82	1650.66	0.088678	0.820418	0.874591	1.065893	0.092063
0.6509	252701.1	292537.9	149208	25161.1	140877.6	258132.5	0.679211	0.331788	0.4731	1.637186	0.711218
0.6509	3022.81	4225.19	2718.07	1450.92	3010.04	485.68	0.890904	0.139489	0.263735	2.014715	1.010576
0.6509	2793.23	3703.38	919.69	8205.04	7872.47	2097.75	0.800131	0.204524	0.343156	0.408044	-1.2932
0.6508	3853.69	5911.1	4042.25	15436.93	9441.42	17142.79	1.268049	0.047671	0.127797	0.328574	-1.60571
0.6507	9712.16	6665.25	13527.29	3037.42	1022.08	2672.88	1.221006	0.049575	0.130967	4.441921	2.151184
0.6506	2590.72	13120.15	4042.33	80998.83	100201.5	116318.6	1.301365	0.007198	0.039577	0.066393	-3.91282
0.65	3322.39	1092.76	4154.99	1728.33	172.24	637.53	0.935165	0.145679	0.271281	3.376597	1.75557
0.6499	122121.3	98077.7	112514.8	467641.7	407443.6	435257.4	1.364725	0.000869	0.01464	0.253914	-1.97759
0.6499	2741.49	1950.42	821.65	17900.13	15868.59	18701.87	1.313332	0.000253	0.009348	0.105079	-3.25045
0.6493	6454.43	3362.25	38061.65	46768.25	44381.36	37952.28	0.990389	0.127928	0.248835	0.370857	-1.43107
0.6493	46.68	35.9	1089.09	282.56	178.29	261.14	0.390319	0.710117	0.793489	1.622834	0.698516
0.6491	3635.17	7453.2	4109.11	7701.41	5498.32	5569.23	0.618538	0.453351	0.580539	0.809713	-0.30452
0.6481	48737.51	91451.77	65391.09	72962.47	121996.2	116502.3	0.897645	0.153836	0.281635	0.660052	-0.59935
0.6475	4956.92	7303.11	4236.43	4287.62	8736.72	9044.14	0.567048	0.370718	0.508078	0.747512	-0.41983
0.6474	12030.91	17015.91	8024.79	6727.78	3947.07	4832.86	1.162276	0.098655	0.209773	2.390528	1.257329
0.6472	1007.86	858.06	1162.36	141.11	45.67	730.82	1.017266	0.065506	0.158633	3.300218	1.722561
0.6471	86680.55	73396.64	82692.6	2333.58	2894.7	1673.19	1.367056	0.002334	0.022458	35.17653	5.136541
0.6469	826946.4	656284.5	937321	64003.16	592069.2	649352.8	0.755425	0.173725	0.307208	1.854225	0.890816
0.6463	30378.51	19492.23	26483.2	9166.88	22765.37	20519.32	0.77457	0.210768	0.349871	1.455704	0.541717
0.6453	90741.02	82556.12	61677.02	43493.55	41262.43	46806.66	1.261198	0.053275	0.137331	1.786025	0.836752
0.6451	118724	89786.94	103441.2	26075.63	24042.68	25273.21	1.363312	0.010752	0.049697	4.137762	2.048851
0.6448	703.47	228.83	1075.8	263.02	160.66	1428.84	0.291792	0.919376	0.943844	1.083983	0.116342
0.6443	3703.56	9734.9	11983.88	568.11	3257.82	873.84	1.132865	0.095175	0.205075	5.409273	2.435435

0.6443	431.08	1174.19	473.86	36726.86	7453.58	8786.63	1.278763	0.21741	0.357484	0.039253	-4.67104
0.6436	60517.7	73397.01	81822.52	64739.65	90458.05	51397.94	0.229204	0.829791	0.880492	1.044249	0.062465
0.6435	2197.96	615.63	2286.22	963.81	270.98	792.16	0.839339	0.191153	0.327006	2.516002	1.331133
0.643	6277.42	6694.48	3694.34	14859.72	8319.13	13255.99	1.146853	0.060119	0.148896	0.457426	-1.12839
0.643	198865.5	236316.2	173565.3	94876.59	90116.04	98415.1	1.333164	0.025429	0.083469	2.147955	1.102964
0.6422	22030.33	28661.95	21720.28	56032.07	49496.11	32113.78	1.16981	0.081549	0.183705	0.526094	-0.92661
0.6422	3948.48	17181.61	3976.51	6117.52	3393.91	4472.26	0.445894	0.490073	0.609818	1.79542	0.844322
0.642	168629	147774.3	149281.1	84570.57	81982.07	83813.24	1.359486	0.007999	0.041806	1.860015	0.895314
0.6414	2847.23	1185.02	4322.06	1262.96	1570.2	1114.8	0.856698	0.24486	0.388211	2.116108	1.081413
0.6411	21422.76	33687.23	36125.09	19970.65	13183.57	12907.26	1.158231	0.06079	0.150132	1.980724	0.986028
0.6407	773982.6	892012.7	824860.4	287283.7	311743.5	345209.2	1.359903	0.001014	0.01519	2.637958	1.399421
0.6403	12233.37	9578.64	12567.08	15320.38	18568.59	18074.61	1.225726	0.013435	0.056593	0.6616	-0.59597
0.6393	812.77	151.39	3129.98	1111.3	31637.64	1743.13	0.721701	0.420441	0.550583	0.118698	-3.07463
0.6391	23255.68	21757.72	22892.02	22245	20213.61	19719.51	1.001015	0.116374	0.233919	1.092111	0.12712
0.639	1661.19	115.52	639.61	112.62	150.08	3094.71	0.14646	0.792893	0.854653	0.719698	-0.47454
0.6389	59866.79	17957.58	15314.51	54344.08	50852.59	22905.5	0.551687	0.546775	0.661686	0.727067	-0.45984
0.6378	19068.37	15835.09	24119.52	13154.31	14289.9	16771.34	0.965738	0.166026	0.296601	1.334892	0.416723
0.6371	11679.7	6752.98	10976.42	2748.01	3433.53	3545.29	1.294923	0.047598	0.12768	3.023503	1.596221
0.6369	18506.3	12505.17	25813.77	20117.1	19388.36	23818.53	0.436937	0.639334	0.735125	0.897373	-0.15622
0.6368	6950.76	8933.77	1857.25	10537.11	1080.7	1292.81	0.499707	0.693596	0.780027	1.3742	0.458592
0.6366	3428.96	4627.67	2115.92	5674.73	655.29	436.35	0.715677	0.588805	0.695326	1.503398	0.588227
0.6357	20951.64	23116.08	27411.57	7894.24	3913.03	7837.96	1.28876	0.002667	0.024096	3.638506	1.863346
0.6356	26560.1	21276.1	10677.15	23769.33	23186.91	32563.47	0.720938	0.28733	0.430681	0.735835	-0.44255
0.6355	6938.34	2851.75	1854.37	16354.04	20281.9	14092.09	1.229443	0.005823	0.03591	0.229547	-2.12314
0.6349	96508.73	107277.5	109184.2	68175.78	60143.32	55973.08	1.328968	0.001345	0.016654	1.698229	0.764031
0.6346	17693.81	15204.45	21660.57	14273.67	14236.28	15453.19	0.957704	0.196924	0.334134	1.241013	0.311518

0.6343	907.2	61.35	419.37	799.29	686.41	13.78	0.165787	0.919752	0.944007	0.925601	-0.11154
0.6342	156314	406026.5	121713.6	152638.2	57471.93	118306.4	0.796878	0.31577	0.458116	2.082886	1.058584
0.6332	21230.4	28368.72	20407.99	33847.13	13252.82	23723.77	0.113064	0.96931	0.977156	0.98847	-0.01673
0.6327	555.2	53.56	882.02	517.15	151.02	735.71	0.179011	0.927044	0.948261	1.0619	0.086648
0.6326	9970.63	8975.17	10136.49	14579.5	14358.41	10857.62	1.138149	0.087036	0.19278	0.730793	-0.45247
0.6326	97773.24	74739.77	93785.27	83235.32	75808.64	85637.06	0.546844	0.425875	0.553485	1.088349	0.122141
0.6322	14390.37	12649.64	13893.61	3027.21	1823.03	1492.01	1.339972	8.42E-05	0.00568	6.454116	2.69022
0.632	27428.03	21076.09	21274.15	48125.08	39146.97	43407.36	1.303094	0.004222	0.030527	0.533965	-0.90518
0.6317	21772.1	23940.78	23540.21	6440.93	49951.35	6356.48	0.54791	0.89504	0.926058	1.103657	0.142291
0.6316	337.36	374.55	442.77	1791.9	668.1	751.64	1.127295	0.197519	0.334884	0.35953	-1.47582
0.6315	23789.67	71123.75	60851.71	31810.38	29011.88	30667.31	0.730645	0.274384	0.419076	1.702545	0.767693
0.6315	9169.31	32757	33338.04	411.13	8105.05	120311.4	0.413294	0.692893	0.779706	0.584226	-0.7754
0.6314	62285.5	54923.16	56912.74	38819.7	30318.49	24805.82	1.263369	0.009678	0.046822	1.853459	0.89022
0.6309	232566.5	152292.8	154206.6	59339.25	79281.11	106955.5	1.200677	0.046118	0.124725	2.19511	1.134293
0.6307	56557.57	47421.4	53958.53	58426.75	60694.84	60134.82	1.054514	0.112784	0.229053	0.88107	-0.18267
0.6301	18955.78	16319.14	18616.42	16457.12	15060.15	29537.33	0.250162	0.658198	0.74977	0.882675	-0.18005
0.6291	2.45	92.88	119.51	175.64	24.6	10.38	0.07385	0.98357	0.988062	1.020036	0.02862
0.6288	8992.38	16536.12	15396.99	7614.57	1617.55	6567.51	0.995651	0.051758	0.134347	2.590282	1.373109
0.6287	120.3	109.21	125.44	163.27	177.48	177.86	1.328174	0.001296	0.016383	0.684426	-0.54703
0.628	84904.33	99549.17	76031.87	5498.55	3336.67	4235.22	1.364888	0.006487	0.037805	19.92935	4.316823
0.6276	2250.55	2439.87	2709.87	1757.53	65.64	1273.18	0.813385	0.095541	0.205661	2.390004	1.257013
0.6275	1887.19	7235.06	2480.89	5969.65	7427.91	10937.42	0.967819	0.13296	0.255232	0.476809	-1.06852
0.626	3578.04	604.49	3197.74	15079.23	4698.38	4172.81	0.909993	0.256736	0.402242	0.308148	-1.69831
0.6256	774.11	548.75	130.25	1031.05	289.79	1622.19	0.596934	0.333348	0.475013	0.493746	-1.01816
0.6247	40836.82	31549.65	34015.57	27456.16	24563.94	37676.51	0.712815	0.321981	0.464214	1.186244	0.2464
0.6241	5960.54	7312.84	6625.94	76343.84	168980.6	164753.1	1.351402	0.04989	0.131232	0.048526	-4.36511

0.6241	959.96	985.2	4531.51	13047.63	1703.99	1167.64	0.428936	0.507205	0.625079	0.406845	-1.29745
0.624	291392.7	308584.5	317700.6	70467.93	71097.51	60107.02	1.368761	0.000155	0.007197	4.550337	2.185974
0.6236	146617.7	172866	158613.8	288057.7	297572.7	218988	1.264762	0.038486	0.111989	0.594192	-0.751
0.6235	5676.1	5891.1	6233.81	5673.38	5383.05	5441.8	1.054154	0.0979	0.208779	1.078965	0.109648
0.6234	14848.89	6414.23	16720.47	10496.71	10686.48	17888.27	0.1509	0.93233	0.95154	0.972157	-0.04074
0.6224	23556.34	14401.29	16832.24	34894.11	45208.02	36008.1	1.259419	0.00936	0.045793	0.471878	-1.08351
0.6223	20314.38	29686.54	23393.78	58255.17	42748.42	47890.92	1.273617	0.014571	0.059559	0.492931	-1.02054
0.622	2184.45	1920.89	1219.91	378.61	55.56	638.17	1.087589	0.020599	0.073599	4.966009	2.312087
0.6211	3649.81	5444.46	6411.17	4408.39	2853.51	4123.42	0.785648	0.233384	0.374842	1.36188	0.4456
0.6199	17596.74	9635.11	7099.03	9963.08	16087.73	20755.13	0.610468	0.402579	0.536649	0.733473	-0.44718
0.6197	12314.22	14654	16593.48	1509.84	3671.7	1952.85	1.316252	0.003032	0.025806	6.105876	2.610198
0.6193	9927.94	5754.69	8788.92	82286.56	57879.89	54611.34	1.34594	0.021072	0.074737	0.125638	-2.99265
0.6189	1852.96	3701.8	2442.73	8108.62	3349.63	4905.21	0.998547	0.17492	0.308695	0.488741	-1.03286
0.6181	2911.72	4165.12	1948.65	287.68	403.18	630.21	1.304078	0.053977	0.138322	6.831954	2.772298
0.618	3251.12	2096.67	1336.19	8649.63	3199.9	1718.13	0.627041	0.391186	0.527593	0.492641	-1.02139
0.6171	494.43	1805.85	458.11	7576.82	7596.34	5899.09	1.272929	0.001311	0.016474	0.130902	-2.93345
0.6161	77682.7	73264.77	95757.26	43019.77	40285.92	42341.62	1.332237	0.026456	0.085869	1.96347	0.973406
0.6153	4584.12	5391.43	7697.4	2948.25	7970.25	5650.75	0.245542	0.843241	0.889136	1.066611	0.093035
0.6149	2612.92	2059.76	4387.29	6831.23	2276.98	5523.61	0.644818	0.310245	0.453635	0.619196	-0.69153
0.6133	253.44	94.24	184.35	358.59	3872.36	1118.39	1.091853	0.271273	0.416513	0.099457	-3.32978
0.6126	183262	171091.7	170381.1	113264.1	108485.1	116060.7	1.359557	0.000886	0.01464	1.553343	0.635377
0.6126	49306.13	46668.66	40040.38	24229.19	21206.3	19040.03	1.331899	0.004247	0.0306	2.109563	1.076944
0.6117	102603.2	89344.03	90219.69	168396.3	199952.9	155870.9	1.322459	0.017616	0.066446	0.53826	-0.89362
0.6104	14733.72	11171.11	14819.42	14475.91	14577.04	30592.42	0.717053	0.360022	0.500426	0.682773	-0.55052
0.6097	15929	7623.93	16249.79	18962.62	21157.21	15188.93	0.788977	0.208201	0.347188	0.719646	-0.47464
0.6095	274.17	769.05	21062.47	170.76	1065.19	1136.27	0.45754	0.438202	0.565657	9.318567	3.220108
0.6081	8730.44	8398.15	12573.72	2370.08	2794.85	2941.22	1.339841	0.031206	0.096267	3.66417	1.873486

0.6067	24248.26	29177.91	21706.92	13417.54	13818.92	8929.86	1.254459	0.010915	0.050031	2.077433	1.054802
0.6066	331095.5	288283	329976.3	173277	104972.2	168359.5	1.25945	0.005173	0.033759	2.125697	1.087936
0.6064	12705.43	23360.04	23458.77	34078.84	30355.75	55127.3	1.081938	0.105429	0.219049	0.497853	-1.00621
0.6056	409.51	32.63	76.52	406.3	557.19	89.14	0.645264	0.384945	0.521933	0.492728	-1.02114
0.6054	23986.92	21215.16	25700.83	23144.55	20753.46	24926.17	0.257392	0.717053	0.798986	1.030203	0.042929
0.6054	645459	631724.9	614934.9	245365.7	367945	451557.5	1.179754	0.041481	0.117021	1.776857	0.829328
0.6049	5563.4	5058.02	4965.63	6286.85	5586.07	5647.96	1.028856	0.093421	0.202699	0.889627	-0.16873
0.6048	9301.42	18239.15	9797.85	9142.87	15393.09	3107.47	0.541924	0.520923	0.636943	1.350716	0.433724
0.6047	383892.4	301110.6	391170.9	181743.7	314903	220020	1.044014	0.076678	0.176216	1.501638	0.586537
0.6045	12343.59	9441.95	2666.01	4556.57	931.28	1286.97	0.968341	0.165422	0.296229	3.609181	1.851671
0.6044	14455.4	11247.02	10482.7	15607.82	14744.95	18567.36	1.082998	0.064991	0.157511	0.739678	-0.43503
0.6043	167890.5	145151	108357.3	140454.5	246295	179533.2	0.779182	0.262257	0.407987	0.744149	-0.42634
0.604	228545	222158.2	234027.9	56414.5	54050.7	55736.3	1.372445	0.000239	0.009219	4.119885	2.042604
0.6031	1301.65	18706.61	77.27	9780.31	1251.41	3662.09	0.409837	0.802894	0.862023	1.366938	0.450948
0.6026	198713.3	184901.8	207172.3	139488.7	137408.2	152595.6	1.317735	0.003536	0.027803	1.375548	0.460006
0.601	50940.14	60241.82	55233.89	47044.99	58119.83	48523.82	0.612964	0.391796	0.528253	1.082812	0.114782
0.6002	16271.81	15006.35	15974.16	3529.09	51503.83	59558.75	0.251258	0.327945	0.469771	0.412354	-1.27805
0.6001	523.81	562.89	1027.09	423.39	2903.29	321.03	0.083203	0.608635	0.711723	0.579484	-0.78716
0.5996	704.04	3596.86	557.05	280.09	1095.15	965.56	0.459593	0.489018	0.609137	2.075337	1.053346
0.5993	6854	48822.16	43569.93	2493.05	2019.27	47624.66	0.679241	0.478541	0.601488	1.903564	0.928703
0.5989	2255.09	390.43	3962.35	1189.93	1420.01	1580.43	0.087353	0.517003	0.633395	1.576918	0.657108
0.5966	50707.41	59092.98	58686.71	25259.13	23713.38	21497.49	1.356562	0.002807	0.024873	2.390905	1.257557
0.5965	13748.66	5422.32	5928.12	45511.98	43181.96	49585.63	1.303162	0.000605	0.012759	0.18151	-2.46188
0.5963	4146.25	5716.2	12771.94	11741.78	4601.33	5892.72	0.024382	0.971139	0.978548	1.017924	0.02563
0.5955	146225.5	95817.65	154045.6	121825.3	89556.88	257750.7	0.172949	0.691813	0.779085	0.8443	-0.24417
0.5952	824.32	800.21	5761.63	5041.83	2164.9	1199.37	0.351033	0.875012	0.912046	0.878667	-0.18661

0.5946	16980.53	10667.7	24662.79	11349.39	6471.79	14289.84	0.798869	0.238756	0.380979	1.629068	0.704046
0.5938	8286.66	10589.88	9644.17	24086.64	28856.29	21192.13	1.334697	0.014799	0.05985	0.384713	-1.37815
0.5937	9279.1	9023.3	4968.23	4805.45	1704.43	4368.38	0.995066	0.079756	0.181047	2.139187	1.097062
0.5937	92128.88	97906.76	110896.7	128436.4	117661.1	130189	1.197231	0.025151	0.083056	0.799743	-0.32239
0.5936	6544.03	9458.93	6457.56	12539.63	14440.66	11290.89	1.21357	0.017492	0.066132	0.586878	-0.76887
0.5936	27007.63	3348.19	5793.55	4425.99	5131.26	3632.63	0.60014	0.415527	0.54677	2.74069	1.454539
0.5934	427.54	202.76	285.75	120.48	115.38	90.13	1.242456	0.092758	0.201846	2.810056	1.490599
0.5918	285.53	2507.42	7964.43	3060.41	3694.8	1729.87	0.274126	0.775002	0.842452	1.267799	0.342327
0.5912	3016.55	1250.99	834.37	721.44	1719.9	739.14	0.544072	0.455199	0.582051	1.604132	0.681793
0.5905	20344.56	23592.69	18814.16	2910.47	2973.8	3254.48	1.368726	0.005934	0.036092	6.86652	2.779579
0.5904	189776.5	159681.7	164356.6	189787.2	197541.2	196471.4	1.051592	0.122181	0.241426	0.880122	-0.18423
0.5886	23572.23	15783.24	15626.62	382.26	300.25	278.17	1.36831	0.02055	0.073492	57.23247	5.838762
0.588	14009.26	18933.26	15835.16	4794.59	781.5	3325.92	1.188992	0.00233	0.022458	5.479401	2.454018
0.5862	4459.7	6080.64	7202.59	5406.66	2212.52	2892.1	0.953526	0.130133	0.252224	1.687989	0.755306
0.585	323.05	580.15	1049.34	607.74	832.11	728.61	0.389892	0.772505	0.840996	0.900427	-0.15132
0.5849	45529.86	42754.87	30977.6	62084.39	37678.49	40513.2	0.492159	0.48553	0.607645	0.850197	-0.23413
0.5849	1510.8	1318.96	1483.5	6281.19	3963.84	3483.35	1.304684	0.067422	0.161284	0.314186	-1.67031
0.5839	62765.48	32916.67	55087.62	2194.69	73221.44	58447.61	0.465397	0.827116	0.879638	1.126293	0.171582
0.5838	420333.4	391840.8	425041.4	1198.9	541611.5	4391.21	0.921294	0.328671	0.470192	2.260987	1.176953
0.5833	12860.19	8179.12	7765.64	46081.66	43568.19	48633.62	1.345296	8.3E-05	0.00568	0.208304	-2.26324
0.5825	807.92	4186.97	5646.22	13537.68	15718.55	16424.15	1.12432	0.004435	0.031587	0.232947	-2.10193
0.5821	59980.01	19664.86	54170.89	23475.6	21747.34	25159.01	0.821009	0.234348	0.375836	1.90128	0.926971
0.5817	9451.88	805.13	12865.77	23080.56	6291.17	10385.25	0.590489	0.426718	0.554415	0.581603	-0.78189
0.5802	744.04	240.07	1035.32	381.86	82.32	270.73	0.858046	0.198634	0.335989	2.74786	1.458309
0.5786	11604.29	8175.84	10785.89	14338.01	16825.13	16720.89	1.218576	0.013285	0.056224	0.638334	-0.64762
0.5778	15710.81	17452.92	15795.92	12561.69	12439.99	13700.62	1.278337	0.010325	0.048762	1.265032	0.339174
0.5774	247.88	18.33	121.85	17.58	203.51	292.19	0.124295	0.711121	0.794	0.75604	-0.40347

0.5773	1640.66	1079.62	750.03	3513.64	1483.2	1854.28	0.952473	0.205167	0.343837	0.506532	-0.98128
0.577	13319.03	4204.98	15005.69	28.69	1086.23	1962.13	1.034951	0.095807	0.20595	10.57172	3.402138
0.5767	13063.9	13115.35	13914.21	58.69	265.89	658.49	1.298401	1.16E-05	0.003599	40.78393	5.349929
0.5764	45796.52	61677.93	33512.37	54796.83	59286.3	57716.58	0.742226	0.334272	0.475983	0.820646	-0.28517
0.5762	884.08	885.01	5446.36	730.03	925.86	679.62	0.712501	0.396733	0.531969	3.089454	1.627352
0.5736	5469.74	9948.27	15216.11	10021.8	1026.87	10764.65	0.516794	0.523828	0.639241	1.404377	0.48993
0.5719	20686.23	20190.53	13644.4	459.63	427.34	421.1	1.369095	0.015973	0.062461	41.68061	5.381305
0.5702	15975.99	13058.27	11901.88	130258.8	135303.2	129287.1	1.368965	3.97E-06	0.002475	0.103675	-3.26985
0.5698	32787.48	29696.64	33144.35	23993.47	24738.9	24586.51	1.324325	0.017621	0.066446	1.304282	0.383255
0.5695	31265.41	29158.38	13975.93	57628.98	54446.48	31945.89	1.007149	0.085033	0.189112	0.516588	-0.95291
0.5691	353.03	322.04	229.08	124626	3385.41	8741.99	1.202525	0.370772	0.508078	0.006612	-7.2408
0.5683	10951.54	1927.52	358.22	335.65	2880.22	2770.38	0.191241	0.543972	0.659026	2.211281	1.144882
0.5681	3383.25	7316.13	2603.22	3341.41	2297.86	3595.53	0.526674	0.45369	0.58059	1.440486	0.526556
0.568	127771.3	184820.4	194264.6	114282.7	111483.5	149405.1	0.921682	0.159272	0.288808	1.351	0.434027
0.5679	8319.84	7353.81	6572.16	112898.9	113299.1	136771.4	1.37003	0.004656	0.032268	0.061288	-4.02824
0.5676	23665.73	29957.23	30610.54	7647.03	8599.32	10011.26	1.347641	0.008149	0.042258	3.207965	1.681659
0.5666	4292.9	3195.05	4156.14	7138.18	9151.54	13707.23	1.247002	0.083508	0.187137	0.388176	-1.36522
0.5661	1927.41	786.24	1667.96	1045.81	656.63	1359.07	0.588476	0.347043	0.488083	1.431192	0.517218
0.5657	165501.8	154032.3	171139.5	121837.1	123813	118535.6	1.339425	0.009064	0.044899	1.347317	0.430089
0.5642	33498.86	26810.67	36456.81	12408.46	10427.59	9025.98	1.337234	0.010392	0.048879	3.037043	1.602667
0.5618	16802.85	8875.76	16271.57	16111.57	18481.4	19783.52	0.775028	0.242558	0.385879	0.771476	-0.37431
0.5612	47.26	27.71	132.38	26.7	84.34	67.75	0.050722	0.810402	0.867094	1.15974	0.213802
0.5575	5634.48	22371.55	15375.15	721.5	122.35	5606.29	1.043378	0.113963	0.230518	6.725618	2.749667
0.5574	265.52	610.65	472.97	644.1	210.96	976.85	0.237462	0.559014	0.672761	0.736466	-0.44131
0.5554	5566.99	6925.36	5393.44	48288.2	13438.53	21192.27	1.203792	0.17635	0.310339	0.215702	-2.21289
0.5545	26630.2	21510.93	22922.65	61049.31	51929.59	58811.75	1.351074	0.001445	0.017259	0.413665	-1.27347

0.5542	56827.05	46703.2	51717.69	112283.8	127008.8	129670.6	1.354439	0.001224	0.016047	0.420768	-1.2489
0.5528	49785.7	55754.41	92097.22	3124.62	7890	3382.44	1.327984	0.042148	0.117812	13.72762	3.779009
0.5513	70906.88	64848.18	62682.94	88424.33	91835.27	94202.25	1.331588	0.001791	0.019432	0.723008	-0.46792
0.5505	345725.3	348320.5	413430.1	295499.7	306660.5	298585.3	1.185834	0.086491	0.191784	1.22951	0.298084
0.5502	1320.05	7075.14	5879.23	7183.86	3400.72	3702.59	0.192804	0.998516	0.998975	0.999108	-0.00129
0.5485	81832.97	95119.86	81425.97	12581	13060.76	13737.22	1.370369	0.003621	0.028133	6.561338	2.71399
0.5482	1208.63	3679.06	2349.78	12595.66	7153.98	2575.67	0.924714	0.220205	0.360448	0.324182	-1.62512
0.548	3670.57	30514.03	25766.61	25652.55	64085.01	29044.7	0.734488	0.265199	0.411095	0.504715	-0.98646
0.5472	76.7	159.66	234.29	234.74	102.06	357.84	0.475452	0.446994	0.574255	0.677545	-0.56161
0.5466	16387.95	12789	18049.98	21561.98	20556.86	24054.55	1.149664	0.034218	0.102724	0.713685	-0.48664
0.5466	62.7	38.13	29.94	46.8	483.37	36.87	0.596616	0.427409	0.554818	0.230619	-2.11642
0.5461	967.2	526.68	706.82	1850.91	1714.85	229.44	0.224502	0.415223	0.54677	0.579864	-0.78621
0.5459	793.24	1002.67	254.59	362.39	980.94	176.18	0.392918	0.620216	0.721277	1.349448	0.43237
0.5458	55753.12	44750.82	44519.26	50584.1	21446.64	73917.86	0.197291	0.98587	0.98973	0.993659	-0.00918
0.545	27738.73	27082.88	24150.4	2504.4	4615.42	502.17	1.231064	0.00013	0.006596	10.36107	3.373102
0.5434	108.13	715.79	30.13	372.44	727.33	155.42	0.586029	0.652	0.74595	0.680415	-0.55551
0.5433	60.58	240.86	552.94	335.88	309.37	152.88	0.219415	0.912251	0.937854	1.070477	0.098254
0.5431	979.6	567.89	466.25	976.91	1833.79	1424.77	1.096858	0.076116	0.175365	0.475447	-1.07264
0.5417	124.64	521.28	178.69	4309.19	4643.4	2424.89	1.29865	0.032999	0.09996	0.072477	-3.78633
0.5411	307621.9	257176	412613.4	326121.9	311394.8	358948.8	0.172947	0.904905	0.932501	0.980878	-0.02785
0.5407	22692.38	24019	26765.51	25871.03	25711.87	24078.08	0.381018	0.623627	0.724073	0.971133	-0.04226
0.5405	5325.27	4158.82	4563.94	6686.84	6944.07	6562.27	1.274013	0.01864	0.068858	0.695682	-0.5235
0.54	379.56	385.16	407.99	385.51	368.86	483.59	0.362459	0.609096	0.712001	0.947292	-0.07812
0.5397	2864.36	1621.09	2105.29	2816.72	2160.4	2808.99	0.613572	0.409461	0.541353	0.846474	-0.24046
0.5387	28297.87	27423.53	34949.54	38215.51	41243.32	38958.73	1.185053	0.045999	0.124688	0.765688	-0.38517
0.5378	21737.91	21101.28	20810.8	3460.39	4342.95	4372.21	1.366776	2.03E-06	0.001767	5.227689	2.386173
0.536	511.28	986.91	1053.24	5615.83	6269.54	5533.14	1.335344	0.000123	0.006457	0.146478	-2.77124

0.5343	312.03	376.79	751.8	574.36	321.12	1156.09	0.419438	0.521369	0.63731	0.702204	-0.51004
0.532	32759.01	27655.23	33452.86	20249.32	18645.84	22356.35	1.285556	0.011915	0.052727	1.532486	0.615874
0.5319	138765.4	307147.5	202731.3	1190.74	49622.84	1012.6	1.149757	0.04458	0.12237	12.51576	3.645674
0.5318	997.51	287.35	354.42	495.02	247.08	208.44	0.607499	0.424538	0.553083	1.724578	0.786243
0.53	674.45	2460.92	3021.4	1892.59	6001.18	2773.96	0.641196	0.368171	0.506328	0.57714	-0.79301
0.5297	15556.87	15019.79	17007.94	12969.32	12525.67	13109.65	1.284018	0.028984	0.091819	1.232613	0.30172
0.529	8769.9	7625.71	6883.68	7415.96	7982.25	6423.89	0.436384	0.534309	0.649485	1.066776	0.093257
0.5281	34667.8	31258.43	39885.54	36277.8	30370.12	34165.8	0.349739	0.616994	0.719489	1.049577	0.069808
0.5263	34714.95	70616.2	45947.96	10427.27	9636.66	11284.85	1.323571	0.063416	0.154777	4.825678	2.270732
0.5243	6244.93	5342.23	5029.59	9021.48	7675.92	53.1	0.485778	0.988764	0.991265	0.992015	-0.01157
0.5232	13344.86	12674.61	14075.13	14724.02	12808.78	11514.52	0.268386	0.755958	0.828868	1.026821	0.038184
0.5226	604.32	1492.55	1076.86	739.54	237.34	305.51	1.021731	0.118478	0.235927	2.474856	1.307344
0.5215	2508.07	2168.37	2424.32	98.43	184.3	214.7	1.351186	0.000836	0.0145	14.27489	3.835408
0.5211	305024	290796.5	300425.7	129477.7	259160.8	138644.1	1.092451	0.097192	0.207677	1.699746	0.765319
0.5205	1617.34	5755.86	892.48	2738.56	2298.49	2556.49	0.269383	0.896181	0.926871	1.088515	0.122361
0.5205	1551.81	7235.28	1647.73	1028.81	375.89	592.75	1.075065	0.272426	0.417289	5.224071	2.385174
0.5204	23781.93	26720.51	26963.26	19731.6	25106.27	21010.28	0.973046	0.126387	0.246607	1.176429	0.234415
0.5189	7771.6	3981.68	5416.91	1437.13	1659.93	1967.08	1.292122	0.064833	0.157511	3.390544	1.761517
0.5184	1515.26	1379.96	471.26	71.44	436.59	414.22	0.982546	0.118299	0.235802	3.65029	1.868011
0.5183	31651.4	42359.25	36589.57	13540.81	16949.97	13303.66	1.332674	0.010766	0.049697	2.52544	1.336535
0.5179	7220.14	7436.09	12095.39	6680.12	11428.89	142.23	0.605045	0.494712	0.613674	1.465743	0.551632
0.5176	16638.97	11759.79	17171.48	9851	3330.82	4758.64	1.142689	0.025423	0.083469	2.540082	1.344875
0.5176	24297.71	16788.12	22086.98	20259.16	24625.7	31277.15	0.659412	0.336295	0.477523	0.829453	-0.26977
0.5176	4764.52	2884.72	5305.93	7774.51	3062.76	4846.06	0.310648	0.599543	0.704232	0.826047	-0.2757
0.5168	3381.56	5907.31	4740.53	3762.38	7301.6	6253.28	0.49393	0.44504	0.572505	0.81014	-0.30376
0.5155	524.47	650.48	43.55	104.2	231.49	868.04	0.075551	0.987737	0.991148	1.01227	0.017594

0.513	7732.74	3147.86	5804.58	7908.1	9093.76	6667.26	0.807959	0.218104	0.35795	0.704935	-0.50444
0.5129	2530.82	1582.19	5988.73	3005.13	3735.66	2182.79	0.010438	0.802757	0.862023	1.132028	0.178909
0.5118	859.99	52.33	537.91	348.91	55.63	430.99	0.221839	0.491531	0.610666	1.735701	0.795518
0.5117	3672.99	155.13	735.27	2116.54	2530.38	3012.47	0.758452	0.445243	0.572511	0.59579	-0.74712
0.511	15463.73	13578.54	10077.47	893.06	4635.77	3603.03	1.156071	0.008807	0.044309	4.283874	2.098916
0.5087	4733.97	6593.94	4143.99	2237.42	3993.8	3205.07	1.035905	0.096684	0.207099	1.639617	0.713359
0.5057	681.09	1257.89	6223.82	2541.16	3611.82	1057.5	0.178502	0.879632	0.915111	1.132074	0.178969
0.5045	273.63	247.4	428.94	69.17	326.51	258.51	0.634965	0.365369	0.504002	1.452132	0.538172
0.5028	7474.45	2407.65	7144.57	2638.1	3247.65	6098.94	0.435711	0.445446	0.572604	1.420702	0.506604
0.5014	661.15	258.36	308.49	784.25	550.38	236.64	0.321502	0.604553	0.7091	0.781533	-0.35562
0.5013	790.77	666.03	307.27	3865.22	3286.75	4214.98	1.31186	0.001727	0.01925	0.155193	-2.68787
0.5011	21933.72	1749.01	19710.14	5992.64	939.71	750.08	0.870503	0.197723	0.3351	5.648326	2.497823
0.5007	46257.16	41603.93	39612.78	48184.51	44354.59	48126.1	0.938958	0.145721	0.271281	0.906222	-0.14206
0.5005	3323.71	1306.58	1864.19	11511.07	10499.77	9978.51	1.303935	0.000524	0.012159	0.20302	-2.30031
0.95	3130916	2684139	3392708	2261965	1442653	2214590	1.121254	0.03394	0.1021	1.555574	0.637447
0.9343	81999.37	81358.56	62705.24	217683.7	216812.6	227593.9	1.354803	0.000218	0.008882	0.341439	-1.5503
0.9328	19915.29	9506.46	9345.62	12388.88	11606.76	11935.14	0.026675	0.81239	0.868156	1.078946	0.109623
0.9251	29872.15	35898.28	36754.4	77618.5	59913.55	96124.95	1.285603	0.04759	0.12768	0.438783	-1.18842
0.8834	7840.3	15157.18	6100.6	228.78	5689.55	4710.91	0.814984	0.145737	0.271281	2.73755	1.452886
0.8828	38084.19	38193.75	33672.27	3772.76	2570.36	5115.55	1.351835	0.000334	0.010018	9.595373	3.262339
0.8772	16699.5	9263.34	15091.03	9394.14	6495.27	9495.93	0.995543	0.133022	0.255232	1.617228	0.693523
0.8715	19266.42	18153.22	32377.18	12379.03	14569.75	10173.24	1.127816	0.131383	0.253182	1.8802	0.910886
0.85	13200.31	1004.87	19318.79	8255.18	8988.68	1419.89	0.183099	0.467246	0.591727	1.796208	0.844954
0.8429	1491301	1369954	1316211	2135747	1734755	2202946	1.251466	0.03756	0.110105	0.687824	-0.53989
0.8321	8884.93	9995.62	4535.37	2405.18	1339.53	1135.47	1.262175	0.058785	0.147014	4.798167	2.262483
0.8285	254043.9	173511.4	248517	9172.01	5234.32	6232.5	1.363602	0.013696	0.057351	32.75729	5.033744

0.815	60514.48	148550.2	157466.7	108992.9	65899.35	66352.52	0.631355	0.312941	0.455614	1.519334	0.603439
0.8131	1493.06	2285.44	1039.82	4696.27	2068.1	2763.58	0.979984	0.173504	0.307191	0.505704	-0.98364
0.7981	87602.93	78008.55	66100.99	89749.62	84974.72	101847.4	0.933112	0.137881	0.261998	0.837802	-0.25532
0.7951	469977.2	974668	420093.8	269429.9	277889	274660	1.120064	0.188707	0.32372	2.268597	1.181801
0.7933	163295.8	198428.9	145888.2	193588.6	177350.6	185623.5	0.646702	0.404269	0.537915	0.91205	-0.13282
0.7848	365552.4	308162.5	391185.8	221752.7	202923.2	116340.3	1.176558	0.014849	0.059876	1.968334	0.976975
0.782	227662.4	244697.3	220267.3	225154.1	245700.9	269926.4	0.651288	0.354974	0.496258	0.934995	-0.09697
0.7814	121329.1	106130.8	112742	46865.57	36245.61	40902.86	1.354677	0.000345	0.010018	2.743253	1.455888
0.7801	8759.67	7439.96	7402.95	6963.34	7073.14	5665.43	0.975404	0.110071	0.226107	1.197984	0.260609
0.779	198.99	1634.91	804.8	10662.41	13334.62	3973.77	1.195195	0.090558	0.198464	0.094338	-3.40602
0.7773	823.85	36832.51	634.23	20816.68	19782.79	266.86	0.201638	0.954044	0.967359	0.936972	-0.09392
0.7766	2655.82	2722.89	2087.95	6422.8	3919.61	4540.76	1.227257	0.072764	0.169796	0.501685	-0.99515
0.7762	516.97	658.56	479.49	810.43	425.73	1585.39	0.628857	0.372273	0.509494	0.586564	-0.76964
0.7742	33442.6	27193.92	25247.79	16731.63	18962.81	17626.23	1.278848	0.040678	0.115363	1.610713	0.6877
0.7734	69152.91	40290.66	50270.88	29127.33	31376.67	36299.35	1.120669	0.124109	0.24369	1.649886	0.722366
0.7717	30787.49	31980.75	26134.09	7833.1	11848.73	11570.97	1.321896	0.001427	0.01714	2.84462	1.508236
0.7706	388126.9	411495	485900.2	174913	164507.2	154983	1.356185	0.010101	0.047978	2.600149	1.378594
0.7691	768972.7	677511	679880.5	1101890	1033837	1887677	1.151627	0.145754	0.271281	0.528499	-0.92003
0.7689	28780.22	19793.81	17744.2	107731	196880	102557.8	1.320439	0.06387	0.155709	0.162877	-2.61815
0.7689	7376.82	6467.69	7368.78	4402.55	5089.48	6238.46	1.115499	0.05534	0.14054	1.348546	0.431405
0.7684	38069.41	86103.89	81013.56	69204.47	60417.59	54757.9	0.126166	0.698555	0.784188	1.112848	0.154256
0.7682	5606.13	630.25	34193.89	11644.46	5882.42	4725.69	0.187062	0.623587	0.724073	1.816881	0.861464
0.7675	3732.05	4334.74	196.37	1311.69	100.74	1175.64	0.491192	0.277657	0.42144	3.192788	1.674817
0.7659	21624.65	16623.08	21314.12	20972.78	17102.38	22716.96	0.116665	0.868141	0.906749	0.979763	-0.0295
0.7647	2330.18	713.07	1751.45	2932.59	4274.97	2274.2	0.944686	0.110616	0.226798	0.505676	-0.98371
0.7641	5217.83	8685.06	7497.52	781.69	319.52	822.74	1.323237	0.021476	0.075726	11.12316	3.475495

0.7627	836460.8	702645.8	782806.5	873936.8	772831.2	816690.7	0.601443	0.391095	0.527593	0.942542	-0.08537
0.7624	754526.6	610730.2	865542.2	938683.3	990164.7	794810.9	0.887276	0.159549	0.288808	0.819045	-0.28799
0.762	150063.5	120674.5	155041.5	123445.4	114551.5	115054	1.01849	0.144495	0.269628	1.206	0.27023
0.7601	737.17	1223.09	905.12	250.03	155.96	327.44	1.284314	0.027438	0.088333	3.906821	1.965995
0.7588	180828.7	171959.5	185399.4	200741.1	196592.1	176491.5	0.779282	0.254194	0.400012	0.937895	-0.0925
0.7587	38490.11	15058.2	10801.63	38674.07	54770.68	32342.66	0.957389	0.137701	0.261769	0.511577	-0.96698
0.7587	3900.44	5156.88	12138.6	6543.76	14298.23	5578.8	0.371919	0.667909	0.757836	0.802244	-0.31789
0.7569	2779.79	3722.28	1436.55	613.49	2419.99	3348.97	0.403071	0.64548	0.740433	1.24382	0.314778
0.7568	389.02	355.7	6031.36	2740.52	52617.71	3649	0.865264	0.401535	0.535802	0.114835	-3.12237
0.7561	6650.65	4673.69	5963.64	5628.36	43998.29	57936.7	0.95975	0.194083	0.330343	0.160724	-2.63735
0.756	661.3	5656.89	452.78	5112.11	5619.45	4763.9	0.934958	0.227663	0.368094	0.436965	-1.19441
0.7543	2094.65	2225.61	3472.9	6467.05	6625.38	5111.75	1.267985	0.006092	0.036575	0.428097	-1.22399
0.7537	14491.16	10610.56	14621.16	9790.52	10288.88	9241.62	1.099759	0.112256	0.228705	1.354758	0.438035
0.7528	2555.69	4473	2953.48	4523.2	2459.64	3576.17	0.152169	0.829146	0.880434	0.94537	-0.08105
0.7489	1881745	2694583	1933415	1072029	1103265	854860.9	1.286932	0.03857	0.112011	2.14832	1.103209
0.7473	28625.05	62513.06	84457.68	93028.97	54568.37	56008.56	0.377591	0.674484	0.763656	0.86243	-0.21352
0.7473	4226.87	1071.96	72.57	1130.16	391.65	676.7	0.015957	0.488401	0.608964	2.4432	1.288772
0.7469	9311.41	9530.2	15771.22	10458.64	14384.37	13379.56	0.40251	0.652288	0.746028	0.90556	-0.14312
0.7462	193.93	33565.57	31269.18	67.6	28.36	318.64	1.035667	0.183307	0.318849	156.8468	7.293212
0.7445	27530.89	26898.23	28284.21	50205.04	35164.51	39122.04	1.209658	0.08937	0.196575	0.664409	-0.58986
0.7414	836.09	1588.53	1015.38	11284.47	33163.4	11822.08	1.316437	0.134311	0.256677	0.061134	-4.03188
0.7407	199.28	17250.15	17202.25	186.92	232.39	3356.95	0.681308	0.208397	0.347249	9.17619	3.197895
0.7379	13108.89	37200.14	25232.96	48459.72	37789.66	35340.3	0.89843	0.146208	0.271894	0.621286	-0.68667
0.7355	23468.28	30170.75	25614.1	92728.21	99936.54	85031.82	1.359866	0.001115	0.015932	0.285395	-1.80897
0.7342	13930.01	9117.22	12359.23	5193.97	4678.12	7351.74	1.221072	0.030611	0.094977	2.055667	1.039607
0.7329	17476.1	17687.83	17281.37	17128.13	14621.92	18458.44	0.452461	0.576176	0.68472	1.04455	0.062882

0.7317	11797.37	7736.5	12400	5196.14	6889.56	2984.12	1.108558	0.041708	0.117292	2.119061	1.083425
0.7313	23951.06	20342.73	18258.22	41745.07	37625.84	35641.7	1.315783	0.002081	0.020849	0.543871	-0.87866
0.7312	39883.25	51778.75	51941.31	63836.36	56185.21	64514.36	1.089098	0.054921	0.140001	0.778186	-0.36181
0.7272	315686.1	287272.5	234180.6	49487.88	117931.1	331596.9	0.798756	0.314668	0.457061	1.67758	0.746382
0.7264	369.46	2051.01	318.83	23117.88	22344.71	22897.09	1.303131	0.00013	0.006596	0.040072	-4.64127
0.7233	868.68	851.38	1099.2	1224.59	881.89	975.36	0.439823	0.539033	0.653951	0.914798	-0.12848
0.7221	71246.85	66333.53	73526.66	5959.72	4364.07	8174.79	1.357925	0.000111	0.006218	11.41207	3.512488
0.7204	1269.99	715.88	2726.74	23965.45	1716.49	1778.51	0.696808	0.413648	0.545932	0.171614	-2.54276
0.7198	29791.22	14725.73	970.37	104393.2	44354.34	108222.5	1.011509	0.060699	0.149992	0.177014	-2.49806
0.7177	639.62	1382.08	1403.41	161.44	99.96	196.79	1.304113	0.057149	0.144245	7.475305	2.902132
0.7161	10143.55	11047.94	8013.73	13027.45	8398.86	9509.29	0.205223	0.748546	0.822929	0.944065	-0.08304
0.7148	506.55	15.69	609.62	477.43	429.7	71.34	0.186166	0.831587	0.881999	1.156765	0.210096
0.7146	1739.17	924.2	445.1	4113.13	6039.14	4446.83	1.234084	0.008818	0.044309	0.212922	-2.2316
0.7132	1163.41	719.28	881.14	1648.68	9078.57	870.63	0.765029	0.377215	0.514481	0.238305	-2.06912
0.7076	82382.98	112926.2	90484.01	89742	78253.55	77685.87	0.781176	0.280045	0.42386	1.163268	0.218183
0.7057	4219.37	2845.24	3202.81	367.78	3575.94	3483.4	0.577358	0.472838	0.596728	1.382423	0.467199
0.7057	251584.7	1186.02	214710.8	311653.4	5363.66	329875.2	0.217998	0.673695	0.763071	0.722657	-0.46862
0.7025	19262.66	14067.22	19433.28	4024.15	3728.42	3559.43	1.358541	0.015417	0.061151	4.664353	2.221677
0.7014	6498.75	7693.13	2743.17	1119.07	3843.13	3349.74	0.818414	0.187178	0.322239	2.037437	1.026755
0.7011	30204.24	23123.32	32959.32	39607.42	37324.29	40737.66	1.132667	0.057732	0.145349	0.733299	-0.44753
0.9998	179448	185357.5	186907.1	222537.7	224684.4	218381.6	1.355566	0.00026	0.009459	0.82889	-0.27075
0.9997	250854.8	226464.1	241326.6	421805.8	401719.6	437720.2	1.360117	0.000296	0.009591	0.56979	-0.8115
0.999	36682.97	37743.19	39782.37	44898.33	42892	45995.98	1.27597	0.007101	0.039332	0.853664	-0.22826
0.9949	538023.3	1481919	249152.3	2410797	461112.9	2251919	0.675712	0.275481	0.420015	0.442851	-1.17511
0.9947	2909.22	1589.94	854.49	38913.52	28324.83	28222.65	1.333157	0.011856	0.052522	0.056082	-4.15632
0.9937	799.5	388.25	8681.93	5784.6	1525.97	797.08	0.194723	0.861774	0.902348	1.217329	0.283719

0.9929	29849.86	25728.06	29497.38	12379.83	12598.94	13529.8	1.359865	0.004655	0.032268	2.209256	1.143561
0.9928	14896.01	13548.57	20688.42	13186.59	35857.71	14263.54	0.309079	0.593745	0.699496	0.776097	-0.36569
0.9896	666740	726221.8	642516.4	824287.5	887215.7	1655181	0.976658	0.237805	0.380541	0.604594	-0.72596
0.9874	317801.6	318283.6	282967	118328.9	134777.6	119463.9	1.361881	0.001103	0.015864	2.466788	1.302634
0.987	667902	1242764	672773.5	500511.5	577241	544509.8	0.973331	0.234009	0.375568	1.592492	0.671286
0.9847	41646.17	90747.64	74579.45	25315.85	21729.99	267.92	0.880356	0.046124	0.124725	4.374483	2.129113
0.9841	3468.15	2707.86	4538.11	20063.56	16880.2	32911.65	1.325794	0.05504	0.140139	0.153376	-2.70486
0.9839	243108.5	74693.21	210392.6	391099.6	272228.7	97311.58	0.390985	0.489099	0.609137	0.694408	-0.52615
0.9839	59704.25	76053.47	67082.98	56172.22	57576.68	71966.72	0.537386	0.455663	0.582474	1.092211	0.127252
0.981	385.61	12110.53	535.47	10530.61	246.2	798.52	0.028432	0.929153	0.948964	1.125809	0.170962
0.9797	44285.9	49255.75	58336.3	31315.22	32863.97	34471.41	1.275717	0.044107	0.121377	1.539554	0.622513
0.9796	23428.3	32213.47	12996.35	2603.78	4255.98	1694.15	1.291277	0.066553	0.159926	8.024181	3.004354
0.9789	36103.99	9329.12	35896.9	89791.93	82423.54	105811.4	1.147841	0.005194	0.033818	0.292526	-1.77336
0.9768	20777.93	21955.8	31562.51	203919.2	194377.3	204916.3	1.362098	3.26E-06	0.002371	0.123168	-3.02131
0.9749	176038	149314.1	183453.3	684434.7	623542.5	650913.2	1.365859	9.73E-05	0.006015	0.259742	-1.94485
0.9748	648354.9	828435.6	676091.2	352599.3	358062.2	384132.8	1.335286	0.021627	0.075904	1.966472	0.975609
0.9712	2919.51	1601.92	1534.28	1544.21	848.01	1852.46	0.683634	0.335108	0.476586	1.426659	0.51264
0.9709	3303.75	2013.49	3723.61	1989.88	3216.61	2935.02	0.259685	0.663456	0.754699	1.110464	0.151162
0.9657	43022.84	19007.25	53996.93	1538.04	6726.02	8624.29	1.174783	0.079866	0.181092	6.87024	2.780361
0.9655	185.74	33.66	888.34	141.52	81.31	36.58	0.533092	0.395174	0.531164	4.270229	2.094313
0.9644	5292.41	23317.89	12122.43	6155.94	6740.89	14121.31	0.407054	0.493037	0.611944	1.507607	0.59226
0.9643	10391.02	15895.18	10896	2762.59	1500.67	1629.75	1.33185	0.022643	0.077961	6.309543	2.657536
0.963	14590.04	15802.21	17424.82	6292.34	8465.59	8130.04	1.319963	0.001679	0.018965	2.089179	1.062936
0.9602	3120.57	1216.83	4773.96	918.5	179.57	299.88	1.157682	0.123345	0.24288	6.517658	2.704354
0.9585	90212.33	188714	238530.5	13589.86	16875.52	5699.2	1.304186	0.065697	0.158832	14.30839	3.838789
0.958	12491.51	20569.25	15368.82	2970.41	171.08	3181.55	1.099103	0.016226	0.063093	7.659224	2.937198

0.9548	4367.49	5595.22	1895.7	12357.13	4876.11	3669.37	0.642775	0.388413	0.524827	0.567317	-0.81777
0.9535	30327.76	40909.29	25933.05	17537.76	4185.92	1580.16	1.094301	0.02117	0.074964	4.169703	2.059945
0.9535	21214.77	23697.07	22475.19	1654.37	1322.75	1776.93	1.369507	0.00083	0.0145	14.17466	3.825242
0.9529	21199.39	20897.58	24432.79	147124.5	158838.9	130590.9	1.368403	0.003823	0.02898	0.152397	-2.71409
0.9527	85962.94	77471.14	80534.5	27482.34	31902.11	27958.31	1.364766	0.00026	0.009459	2.793232	1.481935
0.9523	26213.19	14169.96	11658.26	25094.93	24619.37	32311.58	0.963134	0.143202	0.267818	0.634451	-0.65642
0.9501	27870.12	31235.23	26767.22	22482.11	20879.18	23821.24	1.23272	0.023746	0.08043	1.278198	0.354111
0.95	1335635	1160147	1196859	510185.3	627612.4	589122.9	1.346974	0.001076	0.015667	2.13828	1.096451
0.9481	36633.29	22812.64	13045.9	11793.38	7916.44	9284.41	1.093181	0.165053	0.296077	2.500216	1.322053
0.9478	161767.4	132963.9	151437.5	29240.76	29096.73	31850.77	1.368407	0.004625	0.032268	4.947083	2.306578
0.9472	167848.6	207672.3	113592.6	72758.15	36543.39	40372.68	1.24615	0.038099	0.111235	3.267854	1.708343
0.9469	12213.82	11381.74	7022.36	11864.65	7471.78	4984.44	0.545878	0.462991	0.588905	1.258915	0.332181
0.9467	19121.99	11981.9	9557.64	1461.75	1688.59	1123.21	1.342676	0.051277	0.133523	9.514696	3.250158
0.9456	6013.51	5152.45	4039.2	2189.44	3201.48	2663.44	1.225606	0.034359	0.102935	1.887817	0.916719
0.9447	8148.07	11347.72	9533.25	45522.04	50345.18	37372.82	1.3534	0.008598	0.043784	0.21787	-2.19846
0.9439	2111673	2164575	2066117	2367962	2331723	2678158	1.170845	0.079932	0.181092	0.85965	-0.21818
0.9423	18917.05	10895.24	16276.33	16998.89	14915.14	19434.26	0.457241	0.560418	0.673229	0.897569	-0.15591
0.9397	5465.09	4235.64	2267.67	1228.95	1716.57	3661.99	0.835157	0.211433	0.350566	1.811333	0.857052
0.9375	32838.69	68742.71	28386.52	4589.45	8674.79	4213.15	1.29182	0.097327	0.20776	7.436346	2.894594
0.9374	56070.31	49531.67	52450.24	282319	238368.1	255094.8	1.368898	0.003271	0.026901	0.203733	-2.29525
0.9364	55167.59	43561.58	54405.86	96888.79	86067.79	88128.44	1.316933	0.001508	0.017674	0.564897	-0.82394
0.9328	317573.3	495063.7	282147.3	24980.13	55148.16	50723.86	1.320804	0.037223	0.109335	8.366575	3.064637
0.9291	21377.08	133164.5	114432.8	67335.53	30805.55	22813.29	0.596253	0.288546	0.431386	2.223767	1.153006
0.9281	28193.36	45406.79	26801.97	15746.28	20005.63	16957.95	1.183217	0.111499	0.227749	1.904807	0.929645
0.9238	17184.24	12798.22	18849.27	10927.35	12902.48	11959.94	1.050374	0.127319	0.248082	1.364405	0.448272
0.9175	1085.47	501.59	590.96	255.18	159.25	74.84	1.203099	0.080384	0.18174	4.451571	2.154314

0.9154	7842.94	3942.58	3320.47	10078.81	8017.16	7005.2	0.973567	0.130685	0.252843	0.601804	-0.73263
0.9104	16960.33	5988.25	5383.02	9073.01	16752.66	10574.34	0.552798	0.582914	0.690843	0.77834	-0.36153
0.9086	81692.92	36961.01	62308.96	95456.73	13897.21	104000.4	0.10178	0.75604	0.828868	0.84818	-0.23756
0.9078	44126.83	36776.14	43465	20171.05	10639.02	31812.06	1.056419	0.063469	0.154818	1.986007	0.98987
0.9059	7987.83	1986.12	11319.88	10531.48	4418.77	534.86	0.418588	0.652993	0.746107	1.375116	0.459554
0.9051	1269.97	1276.18	2871.07	2416.51	1799.48	2549.69	0.60838	0.500376	0.619114	0.800691	-0.32068
0.9032	32088.66	26323.18	28130.74	13560.68	1316.57	8939.58	1.039258	0.014815	0.05985	3.633673	1.861429
0.896	10390.1	8220.71	8478.86	12980.43	905.88	9750.43	0.468528	0.781912	0.846586	1.146083	0.196712
0.895	4008.3	5133.06	3264.64	27739.6	5953.13	3753.47	0.704211	0.389411	0.526006	0.331302	-1.59378
0.8922	12464.38	16370.5	19615.11	35026.64	28164.08	36162.16	1.264886	0.007004	0.039121	0.487656	-1.03607
0.8894	18928.04	7363.18	2723.16	13529.49	4927.53	8557.1	0.147869	0.909957	0.936	1.074045	0.103054
0.8826	3387.59	5268.37	4559.23	36159.71	34912.59	29016.31	1.358706	0.003842	0.029026	0.132035	-2.92101
0.8826	489	49.27	85.66	374.54	54.59	72.62	0.082242	0.828345	0.880434	1.243508	0.314415
0.8809	62789.31	50991.89	62426.26	46304.51	49334.46	36214.66	1.076528	0.056015	0.141795	1.336387	0.418338
0.8798	3175.48	3370.49	1733.18	6446.47	1307.96	6149.91	0.39827	0.379018	0.516294	0.595436	-0.74798
0.8754	8791.81	10502.01	8535	14613.06	14684.82	33409.34	1.089896	0.203091	0.34152	0.44379	-1.17205
0.8752	38358.02	38384.1	44660.13	3751.86	4554.88	22675.03	1.140683	0.029234	0.092273	3.918506	1.970304
0.8734	27463.06	17848.57	23232.64	5308.49	21106.64	7955.63	0.96583	0.130808	0.252869	1.994261	0.995854
0.8731	629.51	981.15	485.78	666.56	260.55	414.81	0.786951	0.256691	0.402242	1.562269	0.643643
0.8726	4933.75	2469.31	1217.18	3075.14	7152.56	3836.32	0.714875	0.337044	0.477732	0.612929	-0.70621
0.8716	1898.59	2735.68	1362.72	2010.62	2450.07	1303.59	0.080678	0.889	0.921557	1.040371	0.057098
0.8714	9523.99	9201.93	11635.76	9147.39	11617.54	12797.28	0.496635	0.468476	0.592108	0.904639	-0.14459
0.8697	6029.98	6711.17	5453.48	5488.15	9919.29	6328.32	0.504739	0.48002	0.602999	0.837083	-0.25656
0.8688	26363.29	41604.47	36169.76	19236.88	16269.82	15519.78	1.262711	0.050454	0.132169	2.040853	1.029172
0.8681	9201.04	15649.68	3116.59	9399.41	23445.88	1345.63	0.097641	0.796719	0.857478	0.817975	-0.28987
0.8632	61761.98	60224.21	46615.75	75660.7	88297.15	87580.09	1.225078	0.012618	0.054611	0.670284	-0.57715
0.858	48697.73	44297.72	48394.71	30318.48	34188.87	38467.56	1.242583	0.015219	0.060802	1.373054	0.457389

0.857	2444.09	2055.62	6933.96	869.58	439.7	826.4	1.198909	0.185395	0.320435	5.353644	2.420521
0.8539	4159.86	3038.36	4601.88	6388.62	9260.72	7652.28	1.2371	0.025258	0.083162	0.506407	-0.98163
0.8524	205560.6	179043.8	199775.5	233141.6	227439.2	218903.9	1.175332	0.039664	0.113224	0.860034	-0.21753
0.8482	5086048	4623481	4415157	2972567	2333022	2895778	1.307931	0.002215	0.021644	1.722235	0.784282
0.8459	92666.65	92499.39	91589.05	25728.77	31163.36	25671.26	1.365236	0.000554	0.012569	3.352032	1.745036
0.8423	15630.88	17541.57	21013.88	11629.95	10912.2	14508.75	1.156063	0.047264	0.127098	1.462484	0.54842
0.8418	39344.48	51750.22	35873.09	15154.04	32696.89	24198.58	1.056708	0.059045	0.14741	1.76223	0.817402
0.8417	19661.74	9630.13	14352.27	7490.3	7072.73	5871.82	1.173713	0.11253	0.228705	2.13577	1.094756
0.8407	11072.57	9750.88	15892.09	25718.06	26336.65	26328.23	1.284853	0.01665	0.064001	0.468412	-1.09415
0.8377	64325.55	52302.12	63703.16	11955.75	23318.2	13089.25	1.306025	0.001201	0.016008	3.728679	1.898664
0.8365	63800.94	82263.32	66768.34	49848.65	35803.95	64072.5	0.972868	0.110449	0.226671	1.421489	0.507403
0.8358	4441.3	5707.65	11297.69	3697.1	3559.16	4478.82	0.927977	0.262951	0.408452	1.827567	0.869924
0.8344	14049.18	15033.67	18204.45	255.41	3119.11	2036.64	1.176798	0.001418	0.01714	8.738847	3.127443
0.8312	147178.4	135931	162142.5	140895.2	106869	142590.4	0.742571	0.268617	0.414773	1.140634	0.189836
0.8298	62074.72	67345.1	53565.27	20269.98	28907.63	26671.09	1.319761	0.003117	0.026176	2.412501	1.27053
0.8277	4740.18	2124.06	4922.53	2898.44	4395.31	3446.6	0.092275	0.752089	0.82599	1.097429	0.134127
0.8272	61636.63	74970.55	57443.34	56084.74	77971.8	66358.55	0.164443	0.809892	0.866762	0.968243	-0.04656
0.8264	19018.94	22222.92	15430.55	43187.95	1024.67	20757.16	0.375827	0.842514	0.888712	0.872289	-0.19712
0.8228	6711.22	11353.45	12128.86	9672	12420.47	12710.29	0.514694	0.485016	0.607444	0.867561	-0.20496
0.8225	334.29	117.93	921.72	254.01	739.22	579.5	0.358135	0.826658	0.879619	0.873602	-0.19495
0.8219	35793.01	31758.13	33267.82	23647.14	17577.53	20540.17	1.286678	0.005389	0.034505	1.632303	0.706909
0.8182	60975.08	38811.74	38920.1	22894.58	20636.73	23532.98	1.255792	0.081472	0.183628	2.068268	1.048423
0.8175	3347.88	1413.75	5439.61	2690.18	4967.16	2998.46	0.227886	0.91792	0.942571	0.957342	-0.06289
0.8168	1627.48	1483.95	1805.51	5397.19	151.07	226.6	0.580638	0.884403	0.918978	0.851439	-0.23203
0.8161	89.93	1212.71	353.54	13205.39	13581.89	83.02	0.64721	0.198138	0.335509	0.061636	-4.02008
0.8153	3857.68	1136.98	869.38	817.33	1542.66	619.89	0.609384	0.422801	0.551654	1.967878	0.976641

0.8048	2183.18	10487.13	7030.47	36737.32	50789.68	59551.33	1.25113	0.014973	0.060154	0.133948	-2.90026
0.8037	2658.2	1407.34	2081.33	8583.69	6973.43	6760.06	1.313735	0.00273	0.024485	0.275432	-1.86023
0.8028	47495.58	43842.22	34460.67	34898.1	30180.47	41054.49	0.741997	0.262666	0.408452	1.18529	0.24524
0.7993	133279.1	81839.89	113577.8	288198	260034.5	279148	1.310869	0.001964	0.020396	0.397274	-1.33179
0.7988	6940.88	4734.94	3796.06	3422.43	6235.39	5940.39	0.016084	0.975535	0.981305	0.991901	-0.01173
0.796	23.99	158.55	38.38	1014.09	254.08	277.61	1.124219	0.216201	0.356167	0.142918	-2.80674
0.7945	86503.92	107142.9	69374.09	28234.99	12931.82	18436.8	1.300046	0.014527	0.059459	4.412835	2.141706
0.7923	1310.01	124.99	2498.75	3350.61	707.17	1444.92	0.451211	0.643174	0.738729	0.714876	-0.48423
0.7917	62535.32	46977.94	41075.74	93389.08	84280.31	74484.5	1.212487	0.016594	0.063879	0.597211	-0.74369
0.7899	15011.23	20709.47	17166.82	61258.35	50449.77	46302.68	1.338434	0.008847	0.044327	0.334708	-1.57902
0.7896	9519.79	12481.02	5951.05	12362.92	7708.35	8440.29	0.113227	0.941461	0.958391	0.980369	-0.0286
0.7862	572633.3	569762.1	639498.5	221609.3	195096.6	223586.7	1.364372	0.001164	0.015971	2.782937	1.476608
0.786	9340.85	7005.01	8954.87	21045.53	20582.02	21096.58	1.347108	0.002265	0.022042	0.403365	-1.30984
0.7811	77417.68	64604.66	80581.36	369764	316870.5	314552.9	1.363915	0.002915	0.025269	0.22234	-2.16916
0.7806	74.51	233.37	36108.54	3247.29	3911.69	2856.25	0.458345	0.539225	0.654002	3.636104	1.862394
0.7795	8178.87	4119.29	5508.95	8654.06	13701.38	7966.46	0.9903	0.13729	0.261216	0.587269	-0.76791
0.7786	110216.5	108105.6	85285.06	43166.19	56636	45404.83	1.307993	0.009755	0.047036	2.090857	1.064094
0.777	42208.59	23113.69	37549.84	4266.23	5262.16	4684.28	1.346487	0.035521	0.105402	7.238057	2.855603
0.7768	6545.76	4021.06	5601.76	135.85	169.19	166.06	1.367237	0.019225	0.070294	34.32091	5.101016
0.7759	2728.55	11236.49	10220.31	2843.06	5672.34	1077.92	0.807584	0.205087	0.343836	2.521062	1.334031
0.7742	13132.31	9876.3	12486.04	35120.8	29106.23	35819.15	1.343197	0.003459	0.027553	0.354783	-1.49499
0.7734	52705.57	52623.81	67479.76	62807.93	75782.12	28911.68	0.227722	0.913927	0.938913	1.031686	0.045004
0.7732	469071.7	389838.7	470931.2	1940483	1795528	1732659	1.366052	0.00045	0.011141	0.243175	-2.03994
0.7722	2974.59	3643.95	3238.23	2802.74	3269.2	4134.13	0.137135	0.807039	0.86415	0.965775	-0.05024
0.7709	257.78	2682.06	10739.04	18361.21	15864.08	28610.05	0.998358	0.032963	0.09996	0.217694	-2.19963
0.7698	155.36	4381.27	30054.06	48620.07	42347.46	30326.67	0.897447	0.070095	0.165249	0.28518	-1.81005

0.7684	109157.4	91823.07	101226.5	61768.45	60575.36	60196.41	1.345732	0.014768	0.059825	1.655563	0.727322
0.7681	6259.94	4265.65	6435.71	5669.36	3089.06	3165.52	0.852006	0.203157	0.34152	1.422458	0.508386
0.7652	43350.04	35689.11	47050.03	24756.16	17310.29	26701.06	1.224099	0.012904	0.055476	1.833557	0.874645
0.7652	339599.1	459312.7	397715	326112.4	362509.8	495207.2	0.086064	0.948832	0.96329	1.01081	0.015512
0.7641	8683.72	34867.4	10510.95	32213.58	24323.1	19624.43	0.68234	0.487252	0.60831	0.709838	-0.49444
0.764	5536.22	5268.22	8005.51	4697.25	4320.09	4798.25	1.005503	0.193379	0.329585	1.361502	0.445199
0.7632	11948.3	12620.84	10801.95	29058.06	31263.33	29547.03	1.365088	4.26E-05	0.004423	0.393588	-1.34524
0.7625	19056.43	8379.84	11436.19	34652.3	36017.96	36197.94	1.253018	0.017213	0.065472	0.363742	-1.45901
0.7621	17670.97	25192.39	21327.65	15854.69	8604.86	17905.93	0.92405	0.11509	0.232365	1.515172	0.599482
0.7612	43506.06	50864.14	43706.16	30803.94	31736.51	33389.23	1.310435	0.020644	0.073699	1.43935	0.525417
0.7608	528.19	69.97	540.42	3389.25	2666.16	2987.59	1.194157	0.000803	0.014349	0.125907	-2.98957
0.7601	336.38	420.46	487.91	116.79	14330.82	49.51	0.023811	0.450496	0.577733	0.085862	-3.54184
0.7594	383.87	282.14	283.88	451.55	472.03	306.21	0.812361	0.21907	0.359265	0.7724	-0.37258
0.7592	110147.4	93568.91	102400.1	84113.83	76852.61	80360.57	1.249119	0.030613	0.094977	1.268471	0.343091
0.7591	21753.15	20771.09	25600.68	21657.37	61081.74	28477.15	0.725073	0.358921	0.499214	0.612545	-0.70711
0.7589	20753.3	22270.17	24998.7	12496.89	10442.22	12815.23	1.328573	0.00373	0.028674	1.902487	0.927887
0.7588	184617.2	160258.6	178011.5	5999.62	5026.76	6510.6	1.371129	0.0018	0.019473	29.81627	4.898028
0.7577	18918.99	21571.23	54388.14	6473.88	19101.6	30125.23	0.640148	0.392963	0.528919	1.70336	0.768383
0.7551	3025.73	71.75	2589.67	73.38	335.67	481.67	0.589609	0.22309	0.363396	6.384891	2.674662
0.7537	6806.22	5566.89	2980.96	3232.43	12175.36	10880.11	0.566748	0.323179	0.465391	0.584074	-0.77578
0.7511	99112.05	78474.78	86257.83	48057.55	63275.84	51457.67	1.255904	0.013095	0.055961	1.620756	0.696667
0.7508	82948.99	85867.86	248927.2	84734.46	90479.63	84958.46	0.577128	0.439318	0.566564	1.605642	0.68315
0.75	1461.1	138.31	1081.28	1417.96	1138.99	1282.64	0.617985	0.430587	0.55778	0.698171	-0.51835
0.7498	468.71	1505.45	222.5	808.09	1405.31	309.16	0.25496	0.840618	0.887873	0.870806	-0.19958
0.7496	5919.79	4670.87	7281.42	6500.35	6421.55	9140.24	0.720617	0.299878	0.442807	0.810079	-0.30387
0.7495	961.17	673.67	671.39	15322.2	15770.94	3555.84	1.274504	0.114365	0.231222	0.06656	-3.9092
0.7491	22489.43	15418.31	27581.42	17327.02	19350.52	17811.19	0.566335	0.408088	0.540517	1.201884	0.265298

0.7488	8994.91	8226.79	17976.64	5461.85	4002.86	516.61	0.980995	0.098056	0.208907	3.526421	1.818205
0.7482	382.75	304.39	227.95	832.58	1053.22	621.54	1.264016	0.038658	0.112091	0.364964	-1.45417
0.7482	21570.08	18566.63	22493.63	573.51	4807.51	672.35	1.235103	0.000571	0.012605	10.34636	3.371051
0.7481	45358.04	37738.99	47953.6	9658.52	10271.46	10533.04	1.365361	0.007908	0.041433	4.301958	2.104993
0.7473	773879.6	669621.2	710580.3	491.83	913.33	644.13	1.370984	0.001782	0.019432	1051.135	10.03773
0.7469	2578.99	1780.71	1609.24	39161.09	37505.13	37528.76	1.366665	8.54E-06	0.003019	0.05227	-4.25788
0.7469	995737	931957.6	904757	1094848	1065970	1094187	1.264351	0.024472	0.081843	0.870184	-0.20061
0.7465	193319	225620.4	186723.1	112021.4	84783.69	96456.07	1.324712	0.003328	0.027115	2.065266	1.046328
0.7463	9276.55	8983.46	11561.57	379.88	5246.46	610.65	1.10881	0.0218	0.076035	4.781406	2.257435
0.7459	9973.08	13583.92	786.82	6547.78	8213.07	8038.39	0.344209	0.905352	0.932741	1.067747	0.09457
0.7458	15559.06	16912.67	18659.05	13357.77	14673.86	11809.47	1.149974	0.037157	0.109215	1.283368	0.359935
0.7458	2809.59	3381.22	665.35	458.63	956.5	1247.95	0.774734	0.228159	0.368623	2.574523	1.364305
0.7458	82690.82	84797.57	52990.87	43656.31	48434.52	48725.81	1.106012	0.119208	0.236948	1.565719	0.646825
0.7458	454.15	1148.47	702.64	1124.24	914.48	1271.77	0.819542	0.238772	0.380979	0.69635	-0.52212
0.7454	645737.5	2121779	1177448	698185.9	903287.1	746637.3	0.691928	0.342367	0.483283	1.680059	0.748512
0.7454	811.38	171.24	66.13	516.97	113.7	280.29	0.156547	0.871516	0.909272	1.151258	0.203211
0.7449	12442.36	7641.74	14992.97	3913.63	8128.97	7515.43	0.955714	0.124344	0.243894	1.793487	0.842767
0.7448	330508	303173.4	348436	114465.7	107813	90807.61	1.359843	0.000585	0.012688	3.136891	1.649335
0.7444	1264	328.53	836.29	643.58	507.03	407.42	0.489192	0.396545	0.531969	1.558905	0.640533
0.7442	11133.88	11518.21	14877.28	2809.93	4977.19	5537.17	1.261053	0.007065	0.039217	2.816613	1.493961
0.7441	550.34	1050.4	1789.29	2085.94	1977.71	1301.86	0.813638	0.214295	0.353697	0.631819	-0.66242
0.7434	534.35	255.98	141.74	165.74	223.24	339.49	0.222423	0.634112	0.731052	1.27949	0.355569
0.7424	20634.38	18469.9	25272.01	29760.95	25786.04	29754.09	1.113812	0.052431	0.135798	0.754695	-0.40603
0.7406	285.9	2433.54	167.14	571.32	258.74	230.62	0.315317	0.496404	0.615247	2.721443	1.444372
0.7406	25998.39	25320.11	47229.92	57580.16	24414.26	36138.88	0.337622	0.619997	0.721277	0.834214	-0.26151
0.7406	27266.78	11793.08	69032.34	185005.8	186938.6	188511.2	1.208114	0.012382	0.054133	0.192865	-2.37434
0.7404	2084.89	4595.61	729.05	2167.39	1417.55	2042.63	0.043587	0.655037	0.7474	1.316652	0.396874

0.7399	75162.24	40153.3	51267.52	33952.11	76019.28	60469.74	0.008021	0.940079	0.957208	0.977364	-0.03303
0.7396	11825.96	18838.6	9754.29	2893.57	2958.23	3820.09	1.312148	0.063375	0.154777	4.179002	2.063159
0.739	253939.2	149801	279924.1	190613.9	57223.53	126472.6	0.886939	0.136021	0.259255	1.826466	0.869055
0.7389	4203.48	6104.36	8696.67	23293.15	24854.82	72272.85	1.235455	0.169338	0.301482	0.157817	-2.66367
0.7386	70193.04	70743.84	46400.23	60989.59	48570.53	63275.99	0.283966	0.635013	0.731894	1.0839	0.116232
0.7382	7353.74	5900.54	12259.14	8167.39	5033.2	3741.25	0.756569	0.295438	0.438246	1.505942	0.590666
0.7366	15760.72	13459.23	15956.22	14890.39	14940.4	13808.24	0.354939	0.604825	0.7091	1.035224	0.049943
0.7354	30338.06	23838.56	23941.17	40881.14	41361.03	48260.92	1.287635	0.005715	0.035639	0.59859	-0.74036
0.7353	333.87	1310.53	1781.72	265.3	427.09	1033.73	0.599819	0.325599	0.467643	1.984868	0.989043
0.7352	3233.09	2939.63	3609.41	3663.53	3832.78	3826.47	1.062563	0.108382	0.223548	0.863934	-0.21101
0.7351	1230.98	494.44	1845.56	1713	2665.32	1528.17	0.799129	0.213682	0.352953	0.604586	-0.72598
0.7349	5681.79	4424.71	1793.63	13753	8172.16	15114.41	1.146787	0.03879	0.112127	0.321282	-1.63809
0.7344	3877.71	19483.72	19620.87	1516.14	12405.87	10979.74	0.485316	0.397116	0.531969	1.726075	0.787496
0.7343	426568.6	440686.9	507946.9	1454826	1398302	1610033	1.363762	0.001288	0.016383	0.308123	-1.69842
0.7338	441.94	362.62	576.22	197.69	188.22	208.82	1.302854	0.051025	0.133265	2.321692	1.215177
0.7337	14726.53	3.45	0.69	19422.21	19110.69	0.69	0.421169	0.385433	0.521933	0.382281	-1.38729
0.7337	16.02	72.61	0.85	383.32	359.23	198.11	1.085335	0.027658	0.088845	0.095125	-3.39404
0.7333	10503.83	9609.03	9245.33	621.19	497.64	211.07	1.344431	0.000634	0.013098	22.07549	4.464373
0.7327	2430.72	1314.87	1452.46	1235.26	974.92	372.53	0.942215	0.121847	0.240876	2.012634	1.009085
0.7324	685.56	878.46	2896.5	1603.84	553.06	1982.74	0.001798	0.904519	0.932324	1.077514	0.107707
0.7317	32902.44	51324.23	44648.15	23334.32	23410.02	15006.46	1.211525	0.034504	0.103171	2.087015	1.061441
0.7317	1154.27	1580.68	2195.05	17223.32	15756.25	18836.16	1.355126	0.001383	0.016884	0.095145	-3.39373
0.7312	932.72	372.01	598.58	4261.81	3245.8	1557.84	1.218731	0.088339	0.194874	0.209952	-2.25187
0.7311	70987.63	32018.16	21760.76	12725.44	106656.8	90551.87	0.268378	0.448572	0.576112	0.594313	-0.75071
0.731	4646.57	4730.67	603	9590.63	1199.08	2879.6	0.227167	0.699982	0.785087	0.73012	-0.45379
0.73	326022.2	345494.1	357506.8	143515.2	158568.2	140543.4	1.36497	0.0002	0.008588	2.32481	1.217113
0.7286	2715.46	2283.83	2082.2	620.67	1002.6	1173.12	1.259775	0.004766	0.03246	2.532369	1.340487

0.7282	8848.16	15543.86	7720.24	134234.3	138279.4	148143.7	1.354738	6.24E-05	0.005041	0.076338	-3.71145
0.7282	27671.49	23048.7	26900.36	2013.7	9147.53	6062.82	1.206106	0.00212	0.021052	4.506521	2.172014
0.728	6749.82	5055.63	15495.65	373.13	853.36	748.16	1.304242	0.120499	0.23897	13.82579	3.78929
0.7279	1824.17	987.13	807.16	70.46	166.55	756.49	1.036742	0.091205	0.199378	3.642134	1.864784
0.7277	11261.52	6022.88	5647.91	16799.71	10855.01	23678.86	1.082798	0.108322	0.223548	0.446731	-1.16252
0.7276	750.06	659.49	969.24	136.64	956.65	186	0.863112	0.300218	0.443116	1.859461	0.894885
0.7272	1693.33	3279.43	2039.74	1387.64	958.18	2633.63	0.662576	0.385371	0.521933	1.408288	0.493942
0.7259	163006.7	166095.9	231810.6	114389.8	105634.3	100838.7	1.257537	0.066284	0.159718	1.74814	0.805821
0.7259	58868.61	44541.24	55476.27	67778.24	55360.04	59178.85	0.771695	0.242301	0.385753	0.871482	-0.19846
0.7254	2236.53	2276.94	965.48	729.95	342.44	1205.69	0.97297	0.115559	0.233096	2.405074	1.266081
0.7244	2869.96	51.99	519.16	314.2	282.09	209.56	0.273743	0.419821	0.55021	4.270162	2.094291
0.7242	21993.43	19577.23	54733.65	84723.12	46297.29	6537.78	0.021402	0.624618	0.724896	0.700099	-0.51437
0.724	3533.33	3939.51	5391.88	761.21	5327.08	4791.52	0.452182	0.701881	0.786504	1.18244	0.241767
0.7236	14437.51	77876.92	27988.47	31213.15	24181.42	25741.47	0.220081	0.568975	0.679311	1.482731	0.568257
0.7233	119713.7	266375.8	155651.6	347409.4	343524.3	94863.76	0.369174	0.451866	0.579149	0.689416	-0.53655
0.7231	502.59	168.18	619.79	750.5	943.86	446	0.747047	0.226583	0.366943	0.602964	-0.72986
0.723	548.84	425.79	688.74	783.82	1171.86	1100.77	1.172153	0.038709	0.112091	0.544216	-0.87775
0.7226	126201.5	118765.6	113737.4	175543.2	130261.4	165976.9	1.11276	0.103329	0.215934	0.760319	-0.39532
0.7223	26164.72	27242.86	30608.78	10263.39	5153.17	10584.99	1.278258	0.001278	0.016332	3.231206	1.692073
0.7223	5279.42	379.43	5292.02	1530.56	1918.76	1333.2	0.253567	0.335303	0.476707	2.28977	1.195203
0.7222	1652.32	650.67	233.76	1112.17	966.67	1317.72	0.619484	0.569867	0.680002	0.746859	-0.42109
0.7212	37699.94	21836.34	35919.95	3884.85	3726.75	2334.39	1.343342	0.028626	0.091014	9.597459	3.262652
0.721	44097.42	32494.35	26965.77	30728.9	26569.8	28249.41	0.683791	0.356359	0.497078	1.210518	0.275625
0.7207	3406.41	24829.32	2112.07	25368.75	3984.55	2511.02	0.077101	0.963672	0.973652	0.952407	-0.07035
0.7205	1085.25	349.05	2358.89	57.91	1785.88	1330.74	0.336731	0.80531	0.863766	1.194882	0.256869
0.7197	4066.38	3352.61	4270.48	4790.49	4935.57	4699.18	1.125222	0.074171	0.172249	0.810348	-0.30339

0.7196	42853.29	29306.74	45456.39	19716.36	22852.69	24536.54	1.200335	0.068706	0.16322	1.752707	0.809585
0.7195	711.43	654.9	241.62	398	450.25	239.66	0.513895	0.368218	0.506328	1.478017	0.563663
0.7192	17715.98	13481.21	20333.04	5237.46	3670.63	5811.41	1.31772	0.017859	0.066937	3.500814	1.80769
0.7189	553.48	460.52	1175.75	166.84	403.79	727.56	0.69367	0.349429	0.490393	1.686772	0.754265
0.7187	2907.67	5502	4359.95	1406.56	4236.28	1887.72	0.859575	0.205792	0.344488	1.695707	0.761887
0.7186	7847.78	22253.32	13832.61	4499.69	5183.67	3132.86	1.189536	0.128325	0.249384	3.427977	1.777358
0.7186	276177.7	281488.1	257676.7	478495.9	492895.5	468413.9	1.36517	3.31E-05	0.004014	0.566287	-0.8204
0.7186	12627.29	13539.08	12336.95	7084.52	6167.23	9465.73	1.239328	0.022525	0.077679	1.694876	0.76118
0.7183	3283.1	2117.2	2071.35	5720.65	2277.56	1184.42	0.032117	0.722369	0.803604	0.813672	-0.29748
0.7176	57092.43	47472.77	54165.03	24305.52	26267.18	26968.93	1.35184	0.00727	0.039627	2.047032	1.033534
0.7175	181369.1	162667.1	157223.4	186888.8	167910.8	187688.3	0.792205	0.232864	0.374463	0.924001	-0.11403
0.7163	38695.95	11425.96	41085.49	51673.16	68922.07	71835.05	0.983218	0.049742	0.131093	0.473976	-1.07711
0.7162	118449.1	115560.4	123949.6	76041.61	78306.31	77371.59	1.365893	0.001999	0.020603	1.544795	0.627415
0.7153	21565.41	17789.9	19165.49	7901.34	7027.1	14945.92	1.143758	0.045714	0.124076	1.958897	0.970042
0.7152	3118.96	2600.51	4174.64	44474.75	25323.67	3980.07	1.013927	0.210079	0.348984	0.134106	-2.89856
0.7152	66146.1	64277.86	64488.89	65957.6	83008.45	91442.82	0.986671	0.179654	0.313746	0.810756	-0.30266
0.7147	936.59	307.65	434.33	336.6	1266.96	876.73	0.460511	0.469372	0.592698	0.676764	-0.56328
0.7147	73344.61	91952.48	108802.6	40791.72	12133.52	42453.11	1.114477	0.013801	0.057649	2.873815	1.522967
0.7142	103.57	71.83	268.37	1663.33	335.33	294.12	1.0053	0.303305	0.446055	0.193551	-2.36921
0.7135	1190684	1134813	1136371	394228.7	406129.2	421412.2	1.371286	9.35E-05	0.005985	2.833486	1.502578
0.7135	23426.09	24407.54	29366.26	600.9	72.44	1167.15	1.276126	0.004351	0.031089	41.94529	5.390437
0.7129	164620.4	53579.61	166190.8	217245.4	176145.6	180765.9	0.769912	0.225955	0.366284	0.669488	-0.57887
0.7127	110.64	227.79	141.85	223.03	384.03	237.68	1.000383	0.131947	0.253821	0.568554	-0.81463
0.7126	824.65	867.24	1098.32	510.86	131.81	958.53	0.790177	0.233616	0.375077	1.742574	0.80122
0.7126	21397.07	6381.84	18626.63	24298.13	40058.81	28559.02	0.957547	0.078342	0.179167	0.499436	-1.00163
0.7116	37626.06	27675.27	25814.43	104128.9	95709.3	93395	1.345515	0.000177	0.007967	0.310728	-1.68628

0.7115	3369.26	8215.12	6065.27	4166.93	7118.87	4675.57	0.141439	0.7559<br>63	0.828868	1.105773	0.145055
0.7111	400.82	262.52	361.95	214.73	347.96	398.62	0.242797	0.7724<br>92	0.840996	1.066555	0.092958
0.7108	27700.16	10882.09	12793.6	22013.4	23504.81	15944.16	0.517034	0.6062<br>97	0.710061	0.835891	-0.25861
0.7108	4925.68	426.81	6070.19	5975.23	5359.63	302.96	0.049362	0.9783<br>98	0.983588	0.981514	-0.02692
0.7102	19124.83	18670.82	19224.63	50500.73	48433.71	47238.63	1.371449	0.0007<br>45	0.014059	0.390087	-1.35813
0.7083	89.17	3926.23	4040.48	5150.19	2056.05	3915.69	0.545959	0.5569<br>27	0.671177	0.724324	-0.46529
0.7078	292.98	180.11	2298.2	1244.13	4843.27	1086.83	0.806316	0.3702<br>15	0.507798	0.386284	-1.37227
0.7076	15975.64	16035.98	17635.9	17978.38	18193.13	11159.43	0.291768	0.7729<br>83	0.841096	1.048944	0.068938
0.7075	2782.62	1861.58	3079.5	137.68	201.89	177.14	1.359406	0.0222<br>73	0.076931	14.94784	3.901865
0.7074	4477.31	2227.5	2674.49	700.84	2022.77	1348.62	1.045727	0.1064<br>41	0.220522	2.303234	1.203661
0.7073	40910.35	27727.22	30127.3	17520.08	19912.52	21189.11	1.220984	0.0722<br>18	0.169155	1.684783	0.752563
0.7068	11473.53	9209.88	7731.94	756.89	1431.39	542.77	1.330499	0.0119<br>93	0.05293	10.40455	3.379143
0.7067	9371.29	4009.31	8303.83	21350.42	22581.15	19510.87	1.241016	0.0045<br>02	0.032016	0.341797	-1.54879
0.7063	599.14	1146.09	437.88	268.43	362.13	107.51	1.058755	0.1427<br>67	0.267436	2.957863	1.564555
0.7062	34710.68	42993.96	49866.21	28015.07	34452.72	29819.93	1.089131	0.0990<br>47	0.210095	1.382317	0.467088
0.7061	11496.98	9528.58	6748.98	9733.16	11228.29	7603.11	0.138677	0.8870<br>6	0.920861	0.972343	-0.04046
0.706	232.34	362.65	335.06	218.55	415.83	184.47	0.403231	0.6819<br>85	0.770148	1.1358	0.183709
0.7053	19502.56	11158.85	26220.39	16696.05	2015.71	1286.4	0.962748	0.1394<br>28	0.263735	2.844352	1.5081
0.704	42355.18	36506.38	33717.11	53374.82	63565.63	55061.88	1.273843	0.0090<br>62	0.044899	0.654518	-0.61149
0.7039	70160.54	80381.46	74544.44	20970.74	19776.19	17016.2	1.36513	0.0008<br>84	0.01464	3.896715	1.962258
0.7038	4722.2	14198.68	2587.7	3005.92	1385.73	1562.53	0.965117	0.2822<br>97	0.426085	3.61235	1.852938
0.7035	2577724	4832853	2606564	2273968	2404096	1910188	0.897376	0.2634<br>22	0.408662	1.520455	0.604503
0.7033	18433.86	17803.11	10507.43	4140.25	6642.67	3835.02	1.260574	0.0400<br>9	0.114143	3.197742	1.677054
0.7031	2478300	2578306	2519130	2422399	2081638	1985260	1.087702	0.1058<br>41	0.219591	1.16742	0.223324
0.7029	12734.05	16733.27	12565.87	16827.87	18188.98	17620.18	1.06057	0.1123<br>64	0.228705	0.798548	-0.32455
0.7028	1076.31	1806.76	866.59	1668.92	593.97	2094.23	0.107161	0.7248<br>97	0.804936	0.860582	-0.21662

0.7024	10235.66	6709.04	11001.23	4154.8	51.04	3039.76	0.87484	0.018837	0.069351	3.856952	1.947461
0.702	460488.5	445547.3	458732.4	720767.8	621959	682102.5	1.339164	0.014727	0.059716	0.674016	-0.56914
0.7019	1507.2	2137.95	1683.64	874.11	50.26	826.32	0.907284	0.026149	0.084998	3.043823	1.605884
0.7016	16867.6	14982.34	17631.32	13109.74	10101.2	13388.05	1.141258	0.034524	0.103171	1.351984	0.435078
0.7012	12160.76	12441.44	8828.62	1598.42	1677.35	3766.2	1.28814	0.005506	0.034788	4.747368	2.247128
0.7009	8775.7	6738.33	6300.57	10304.74	9412	9417.08	1.122137	0.07029	0.16553	0.748772	-0.4174
0.7007	16638.97	26658.32	15234.04	1771.59	663.33	2322.41	1.307104	0.035915	0.106419	12.3034	3.620985
0.7006	68233.38	29359.09	84135.34	36912.13	34820.42	33832.87	0.787552	0.2588	0.404341	1.721471	0.783642
0.7005	79013.41	112950.8	105146.9	5516.12	1886.86	2309.32	1.347405	0.010552	0.04935	30.59121	4.935045
0.6996	2809.33	4794.14	5952.57	7117.11	8646.43	5397.45	0.920263	0.125671	0.24565	0.640615	-0.64247
0.6994	862.64	984.89	584.75	383.91	54.02	381.44	0.984257	0.029283	0.092362	2.968476	1.569722
0.6992	490.5	319.78	157.76	298.12	64.16	341.68	0.455525	0.533028	0.648652	1.375135	0.459573
0.6987	136439	146288.8	105068.3	103799.8	107613.7	104396.7	0.952318	0.19227	0.328025	1.22794	0.29624
0.6984	104875.7	60833.17	257933.5	27427.66	167251.4	88314.64	0.448019	0.555803	0.670564	1.497003	0.582077
0.6981	14889.28	12691.71	11529.14	2062.19	760.56	1723.1	1.321049	0.003027	0.025806	8.60348	3.10492
0.6978	365.05	40.85	51.25	967.88	370.97	342.5	1.015335	0.17366	0.307208	0.271895	-1.87888
0.6973	492.52	436.12	1664.9	1317.69	567.88	558.05	0.067031	0.921845	0.944828	1.061352	0.085903
0.6973	4106.61	7644.68	4632.68	3811.72	2725.06	4550.92	0.839219	0.248253	0.392505	1.477671	0.563325
0.6972	31.47	38.13	40.85	73.75	12.03	15.39	0.479698	0.892037	0.924047	1.091727	0.126612
0.6959	9127.82	5570.85	6889.63	18186.25	18316.95	23259.57	1.308586	0.005325	0.034337	0.361233	-1.469
0.6956	3448.32	4806.86	489.85	163.28	1178.31	494.97	0.863432	0.20804	0.347052	4.761636	2.251457
0.6955	96959.63	104207.3	108677	85.17	105503.6	14849.41	0.791872	0.194307	0.330594	2.572639	1.363249
0.6954	7358.06	558.46	7019.29	11777.26	687.78	9913.4	0.184707	0.581198	0.689321	0.66742	-0.58333
0.6953	4295.46	3523.32	944.8	2955.57	1570.03	827.09	0.470131	0.403395	0.537244	1.637229	0.711256
0.6952	164740.6	130105	207861.6	87086.11	76566.46	85051.23	1.270681	0.061139	0.150738	2.021309	1.01529
0.6946	1300.58	1989.85	3647.25	4893.82	5331.86	7167.92	1.153458	0.024047	0.080881	0.398864	-1.32603
0.6939	113.04	143.4	1241.27	758.98	571.82	577.15	0.657668	0.749439	0.823702	0.784984	-0.34927

0.6938	1818.44	3062.05	5477.34	8932.91	8988.08	9263.75	1.177187	0.033763	0.10185	0.381016	-1.39208
0.6934	54.26	1952.77	1347.63	1073.34	991.65	453.52	0.233273	0.676443	0.765278	1.332002	0.413596
0.6934	18452.68	22305.69	20504.23	16964.39	21630.83	16441.2	0.657404	0.36345	0.502353	1.113128	0.15462
0.6932	50872.78	92753.21	65682.09	48478.16	77125.22	70126.7	0.168821	0.77937	0.84538	1.069371	0.096763
0.6931	100131.4	92727.98	95471.41	156377.9	166670.9	146192.8	1.351026	0.004752	0.03246	0.614461	-0.70261
0.693	905.14	683.74	1104.15	888.05	1470.1	1150.65	0.750194	0.26667	0.412497	0.767507	-0.38175
0.6921	35725.43	33473.06	45689.1	50950.37	55625.58	51741.29	1.177314	0.046867	0.126496	0.72568	-0.4626
0.6919	62559.19	15289.69	81299.59	167307.8	169554.6	154356.9	1.085604	0.024782	0.082461	0.323987	-1.62599
0.6912	5542.06	6365.84	4159.99	8053.23	5200.86	4849.73	0.355024	0.607422	0.710806	0.887541	-0.17211
0.6905	26635.3	18086.54	30382.16	1540.04	1379.3	1140.57	1.362798	0.022709	0.078128	18.49893	4.20937
0.6895	48359.34	46854.22	49301.57	30370.22	31647.38	38725.54	1.266429	0.023723	0.08043	1.434491	0.520539
0.6894	8614.06	2733.94	1979.42	4177.93	6917.68	3108.49	0.292534	0.910018	0.936	0.93828	-0.09191
0.6894	6756.47	5413.69	6355.6	6937.92	3105.65	2814.16	0.834291	0.288726	0.431386	1.440827	0.526897
0.6891	8824.77	4246.52	4952.48	813.8	558.93	3869.38	1.063539	0.07967	0.181044	3.438266	1.781681
0.689	9492.39	9064.1	11767.55	9494.57	3660.91	62.06	0.760191	0.165387	0.296229	2.294227	1.198008
0.6886	9540.72	8743.49	10681.2	3131.93	7262.05	4176.12	1.135332	0.04351	0.120404	1.988004	0.99132
0.6872	1256.57	1708.24	9973.84	21513.03	67979.49	23412.42	1.173992	0.155319	0.283515	0.114598	-3.12535
0.6869	1146.67	677.46	1048.4	520.24	2061.95	227.83	0.397879	0.974567	0.981094	1.022245	0.031742
0.6868	17530.94	23667.1	24387.16	2995.48	3087.69	2544.64	1.363345	0.012488	0.054164	7.601605	2.926304
0.6868	3567.96	4066.53	3545.02	5668.69	9347.17	6169.45	1.217435	0.098399	0.209434	0.527701	-0.92221
0.6868	9936.46	5053.83	6140.09	1512.12	677.73	262.61	1.245914	0.045134	0.123038	8.615994	3.107017
0.6866	1654.49	18520.07	485.43	157.38	941.14	92.73	0.923781	0.38126	0.518053	17.34312	4.116291
0.6864	2665.77	1290.13	1794.67	2226.69	2875.99	1922.09	0.592794	0.440653	0.568038	0.818613	-0.28875
0.6861	35227.56	12043.82	32428.37	24341.68	24516.18	12554.41	0.329933	0.515048	0.631823	1.297782	0.376048
0.6859	169.12	227.94	459.31	1633.24	327.92	107.73	0.310495	0.486777	0.608061	0.413927	-1.27255
0.6858	12140.63	9304.97	9484.26	23308.5	24640.45	26248.06	1.344125	0.000333	0.010018	0.416861	-1.26236

0.6856	7518.57	6232.7	8217.9	3922.27	1099.6	1313.58	1.189808	0.012514	0.054224	3.467657	1.793961
0.6853	52018.14	44114.17	51242.1	50880.51	64667.45	37581.96	0.069022	0.833707	0.883268	0.962414	-0.05527
0.6852	431.1	418.87	336.72	146.17	177.79	210.39	1.304585	0.00596	0.036092	2.22081	1.151086
0.6849	5614.17	1578.54	1400	89.78	569.45	1261.5	0.95655	0.243802	0.387435	4.473669	2.161458
0.6846	12988.66	10397.51	10004.77	6979.29	5084.45	5115.66	1.276549	0.012072	0.053207	1.943662	0.958777
0.6845	358785.8	399597.6	189861.5	328294	186985.8	425043.9	0.027825	0.979018	0.983985	1.008424	0.012102
0.6843	711.69	1800.44	1486.53	1212.8	296.79	1175.8	0.575843	0.377184	0.514481	1.489043	0.574385
0.684	340.29	778.12	1022.99	228.22	555.26	233.48	0.880921	0.195033	0.331571	2.105688	1.074291
0.6839	10102.49	11521.94	2738.96	2947.38	411.99	3686.55	0.890453	0.157497	0.286292	3.457801	1.789855
0.6838	505.92	282.18	417.75	4566.91	1190.58	659.87	1.032282	0.291346	0.434169	0.187904	-2.41193
0.6833	14501.77	11569.98	16041.02	1998.31	1760.53	2050.38	1.364958	0.011258	0.051174	7.249299	2.857841
0.6828	14607.2	15693.95	28658.06	3106.17	3136.74	3850.88	1.328679	0.068523	0.162953	5.841137	2.546249
0.6826	6204.15	5299.83	10216.58	6217.32	7658.19	3504.77	0.489965	0.499103	0.618066	1.249724	0.32161
0.6824	13317.1	14530.78	10598.62	16254.14	13946.92	12454.36	0.550834	0.431262	0.558488	0.901327	-0.14988
0.6823	24765.4	15047.61	14512.1	5539.62	11580.64	10790.03	1.04464	0.100524	0.211952	1.946419	0.960822
0.682	20589.94	10659.03	23436.73	236.7	1124.83	607.52	1.315597	0.044748	0.122599	27.77263	4.795592
0.6817	1285.54	93.73	1066.43	26696.43	20228.12	23063.03	1.255296	0.005391	0.034505	0.034945	-4.83878
0.6803	19708.83	20768.24	22246.13	18118.01	9675.19	7696.44	1.066995	0.098023	0.208907	1.767366	0.821601
0.6799	984.97	1854.18	3245.64	3665.55	1342.77	1554.97	0.123018	0.880106	0.915267	0.927094	-0.10921
0.6795	56.8	142.49	77.17	52.79	434.97	423.77	0.716726	0.230754	0.372127	0.303292	-1.72122
0.6791	958.66	320.13	822.85	857.39	2584.91	167.09	0.100388	0.561544	0.673575	0.58227	-0.78024
0.679	94138.3	98042.87	106038.3	25086.01	17620.55	13409.53	1.342082	7.94E-05	0.00568	5.314331	2.409888
0.6788	759.97	1501.52	1402.86	649.47	1451.57	558.79	0.619077	0.414794	0.546579	1.377663	0.462223
0.6787	169832.6	163388.6	185230.7	312578.4	301860.8	347465.5	1.350645	0.002883	0.025269	0.538985	-0.89168
0.6784	3626.57	15453.07	4357.58	3144.25	4078.69	2178.31	0.814241	0.34558	0.486401	2.49299	1.317877
0.6783	51337.68	41548.36	50136.94	23905.16	20292.97	23172.12	1.340166	0.008356	0.042902	2.12294	1.086063

0.678	237.31	1842.61	78.3	462.62	174.46	44.53	0.427274	0.476532	0.59964	3.166356	1.662824
0.6779	4998.39	4590.34	4757.4	118.65	47.17	4277.51	0.980435	0.141196	0.265554	3.228689	1.690948
0.6777	182219.3	152631.6	187895.5	206126.4	231871.7	190973.6	1.007864	0.094481	0.204286	0.831113	-0.26688
0.6776	59840.61	58938.48	68848.73	50117.5	39153.31	46110.27	1.212053	0.018025	0.067314	1.385923	0.470848
0.6772	190058.4	122527.5	188015	214353.7	140739.1	173912.3	0.215662	0.77347	0.841417	0.946306	-0.07962
0.677	6558.28	433.79	23053	6398.25	8218.63	12381.41	0.420264	0.896251	0.926871	1.112851	0.15426
0.6768	4869.13	2425.33	4776.68	808.5	295.19	164.88	1.262928	0.039361	0.112856	9.515549	3.250287
0.6768	246.51	501.33	541.84	916.91	567.94	390.05	0.636744	0.352494	0.493266	0.687866	-0.5398
0.6766	6902.18	6950.46	6488.94	6620.63	5178.59	5012.95	1.0141	0.139062	0.26352	1.209932	0.274926
0.6763	124019.6	97137.3	125646.5	127054.8	126111.6	116178.3	0.49793	0.510801	0.628622	0.93897	-0.09085
0.6761	686.97	159.5	1175.63	5145.64	4125.88	5816.53	1.213329	0.003485	0.02757	0.13402	-2.89948
0.6754	25648.49	21792.03	25215.4	2912.03	2037.86	2781.26	1.364809	0.002154	0.021199	9.397815	3.232325
0.6749	9051.88	8742.66	10840.57	7644.77	3553.87	2190.51	1.056547	0.074619	0.172646	2.13868	1.096721
0.6739	13186.49	10828.46	13650.77	12398.1	13321.42	12887.03	0.263019	0.759498	0.831355	0.97563	-0.03559
0.6736	7550.07	8975.66	10419.03	9792.84	7868.26	4842.31	0.581855	0.435054	0.56273	1.197363	0.259861
0.6734	23986.07	13641.09	14834.36	12230.12	15063.01	16615.45	0.495883	0.484359	0.606876	1.19479	0.256757
0.6728	23380.32	12562.98	32547.24	12006.23	22694.23	22235.43	0.292407	0.604825	0.7091	1.202941	0.266566
0.6728	4804.98	5470.98	5419.53	12276.01	9981.66	11030.3	1.347416	0.007641	0.04042	0.471506	-1.08465
0.6724	6464.49	3604.71	8387.18	1371.72	667.58	795.19	1.294379	0.061174	0.150738	6.511358	2.702959
0.6722	10141.6	5016.89	9646.78	4690.52	7208.75	7665.06	0.495926	0.417146	0.548303	1.267882	0.342421
0.672	32686.99	34048.45	36978.21	109739.8	91670.59	92684.28	1.360887	0.00643	0.037622	0.352654	-1.50367
0.672	189.42	445.34	1580.34	1055.02	1400.81	1749.96	0.843548	0.25934	0.405016	0.526679	-0.925
0.6718	18957.67	31490.29	14169.03	2368.84	2281.46	6045.1	1.262369	0.066359	0.15974	6.041568	2.594923
0.6717	21486.9	17343.05	16201.91	11909.18	9361.44	7787.51	1.246162	0.014721	0.059716	1.893854	0.921325
0.6717	814549.3	319541.5	610771.2	374189.7	354869.8	389060.3	0.767051	0.282768	0.426423	1.560532	0.642038
0.6715	973145.2	963378.4	947689.4	1055751	1015011	1095429	1.227125	0.045218	0.123038	0.910941	-0.13457

0.6714	161892.1	187377.9	163626.5	74015.34	78950.07	55252.98	1.32818	0.00081	0.014356	2.463262	1.30057
0.6713	389.1	535.54	135.72	60.17	25.42	248.1	0.92742	0.16523	0.296167	3.17768	1.667974
0.6707	278979.4	306873	274734.6	344154.2	330473.7	327364.6	1.230654	0.025551	0.083741	0.858876	-0.21948
0.6706	10686.1	8978.11	4537.78	39.5	205.06	148.04	1.326354	0.049298	0.130493	61.64542	5.945922
0.6702	2053.25	1738.96	1540.95	3155.93	1837.34	4524.61	0.944708	0.210773	0.349871	0.560331	-0.83565
0.6701	549.09	2320.3	310.52	203.82	124.37	219.15	1.031188	0.300389	0.443116	5.809753	2.538477
0.67	3658.08	7847.27	9348.2	4072.95	5295	5576.23	0.55381	0.36744	0.505898	1.39543	0.480709
0.6696	2081728	1635354	2114863	1641272	1495051	1585400	1.044795	0.130645	0.252843	1.235131	0.304664
0.6693	18443.9	15486.74	20538.92	25189.09	20221.7	23749.13	1.033706	0.078124	0.178825	0.787589	-0.34449
0.6693	235806.3	205465.6	196387.8	6646.11	7214.19	7202.44	1.371984	0.003339	0.027156	30.2743	4.920021
0.6692	28695.8	36360.54	33650.22	20181.87	23168.32	23846.38	1.250458	0.025777	0.084104	1.468923	0.554758
0.6691	23372.22	17811.01	22937.59	14700.56	16062.73	20125.42	0.922158	0.142717	0.267436	1.260021	0.333447
0.6689	578.45	210.01	653.07	635.04	210.22	1754.91	0.338015	0.49486	0.613683	0.554398	-0.85101
0.6686	79309.3	83852.03	91560.54	56706.87	71996.2	73228.22	1.082008	0.05965	0.148242	1.261428	0.335058
0.6684	761.45	115.1	757.34	752.17	986.7	1241.6	0.771551	0.166877	0.297633	0.548199	-0.86723
0.6683	53	160.55	1129.59	1085.99	441.4	448.59	0.659531	0.633771	0.731042	0.679734	-0.55696
0.6679	103383.1	80517.74	99008.78	67950.18	61931.53	26764.41	1.00952	0.061401	0.151042	1.806043	0.852832
0.6678	3799.7	814.14	1704.72	1040.79	4726.46	3180.49	0.378132	0.563019	0.674601	0.706163	-0.50193
0.6675	8968.47	1924.14	5196.87	4708.48	3953.92	3355.47	0.17792	0.57573	0.684377	1.338796	0.420936
0.6673	362418.1	446213	371138.1	391691.6	404871.5	422310.6	0.350747	0.680267	0.768806	0.967918	-0.04704
0.6672	56335.19	46756.67	58768.15	533.48	75180.41	8918.33	0.790348	0.389527	0.526006	1.912511	0.935468
0.6668	8057.46	5968.68	8635.4	4821.56	3157.73	3590.17	1.228611	0.025028	0.082774	1.958738	0.969924
0.6667	2006.63	1904.58	1524.69	16092.53	14234.27	15506.12	1.368017	0.000878	0.01464	0.118603	-3.07579
0.6667	16599.2	21229.65	14230.3	11579.68	13767.05	8865.53	1.061488	0.083946	0.187569	1.521652	0.605638
0.666	148874.6	116259.5	105068.8	76007.96	114159	109236.5	0.752242	0.255699	0.401549	1.236469	0.306225
0.6659	1926.2	3487.38	1575.07	614.01	2593.61	1445.57	0.639691	0.396971	0.531969	1.501905	0.586794

0.6658	73615.93	53922.42	55123.79	37687.91	42384.89	46848.59	1.143856	0.084255	0.187957	1.439175	0.525242
0.6654	123453.6	102930.2	95129.79	79368.24	78993.89	95397.02	1.04767	0.099197	0.210311	1.267003	0.34142
0.6646	5877.89	10052.11	4407.96	2277.87	129.11	1199.23	1.061884	0.066904	0.160326	5.639705	2.49562
0.6645	5751.98	3649.71	6325.79	1226.54	7392.59	6893.88	0.275087	0.975677	0.981305	1.013825	0.019809
0.6625	376.33	1046.61	688.8	156.3	4525.12	360.01	0.013285	0.564596	0.675747	0.418877	-1.2554
0.6624	5808.83	5332.66	6330.6	5856.94	4275.81	6161.38	0.420011	0.590383	0.696288	1.072294	0.1007
0.6622	47526.93	29171.07	35625.45	58059.23	83609.7	75739.93	1.211665	0.023414	0.079698	0.516646	-0.95275
0.662	24547.99	20532.9	23054.29	8626.89	19461.36	17849.33	0.893028	0.149159	0.275268	1.483212	0.568725
0.662	851.39	34.33	27.71	58.31	213.62	158.92	0.17428	0.61769	0.71973	2.120065	1.084108
0.6619	2377.17	8751.75	5481.17	7749.53	8660.38	5842.26	0.628844	0.425164	0.553083	0.746448	-0.42189
0.6613	13282.65	14436.93	19077.68	43074.2	40051.63	41758.69	1.33922	0.001078	0.015667	0.374724	-1.4161
0.6608	15395.88	4065.12	16951.38	14083.26	22115.01	12593.63	0.555877	0.461582	0.587622	0.746279	-0.42221
0.6608	891391	855250.2	912717.2	638834.2	642688.4	621659.1	1.361778	0.001661	0.018805	1.397322	0.482665
0.6603	99669.32	123491.8	157671.9	50951.64	100405.8	93927.28	0.928582	0.120149	0.238602	1.552616	0.634701
0.6601	371.07	437.96	550.1	9850.52	11852.47	16421.65	1.364242	0.024196	0.081131	0.03565	-4.80997
0.6601	1007839	205799.3	233467	1085083	853071.5	692727.4	0.870225	0.270591	0.415793	0.550046	-0.86238
0.6598	119.95	236.82	378.85	509.3	942.89	533.17	1.105641	0.078151	0.178825	0.370522	-1.43237
0.6597	5271.26	307.72	3718.86	7079.75	6474.03	5976.44	0.794623	0.139875	0.26406	0.476075	-1.07074
0.6594	8560.12	1721.48	3068.13	6535.69	9879.44	2277.98	0.402819	0.58893	0.695326	0.714152	-0.4857
0.6591	2653.75	1500.11	4090.31	3434.78	472.88	2620.04	0.426797	0.647886	0.742217	1.262952	0.336799
0.6589	2935.94	8967.95	6348.44	12555.91	18094.48	14462.93	1.125179	0.020064	0.07222	0.404588	-1.30547
0.6589	78.92	3336.57	297.95	801.98	279.48	190.69	0.118435	0.521539	0.637338	2.919027	1.545487
0.6582	5496.32	3047.13	1169.98	5705.99	7154.7	5336.72	0.904621	0.138977	0.26352	0.533781	-0.90568
0.658	11723.17	10288.11	9784.19	31.18	902.24	108.69	1.247481	0.000686	0.013584	30.51067	4.931242
0.6576	6857.41	395.47	1407.08	912.3	4341.88	1713.27	0.140241	0.818881	0.873593	1.242917	0.31373
0.6575	203807.1	321589.4	309574.8	191072.9	302791.5	290248.8	0.206486	0.758435	0.830588	1.064861	0.090665

0.6574	953.91	7525.55	7387.03	6358.44	7064.7	2738.12	0.25143	0.971441	0.978625	0.981761	-0.02656
0.6573	86619.83	44090.06	16209.56	9458.82	36581.6	46004.73	0.424105	0.487318	0.60831	1.596167	0.674612
0.6571	49553.37	33891.57	37885.1	169.52	19671.1	131.5	1.059233	0.016554	0.063858	6.074971	2.602877
0.6571	41905.56	32572.54	39178.67	23638.59	28821.68	26143.36	1.219628	0.032625	0.099575	1.445948	0.532016
0.6567	472.89	2323.66	2988.66	212.05	451.59	691.43	0.954826	0.185308	0.320411	4.269307	2.094002
0.6563	124022.7	151509.5	131879.6	37830.85	44215.95	34386.64	1.357747	0.003429	0.027425	3.499095	1.806982
0.6561	4626.52	628.17	245.21	621.01	1607.32	637.9	0.026378	0.598391	0.703069	1.918862	0.940251
0.6561	9582.17	43672.61	27333.77	14387.04	11786.32	10634.12	0.767994	0.275667	0.420152	2.189461	1.130576
0.6554	24663.4	22919.16	42033.2	3833.48	2987.49	4170.14	1.344321	0.049723	0.131093	8.153477	3.027415
0.6553	9407.73	9497.43	13660.52	9331.96	12080.99	9987.47	0.127614	0.825867	0.87911	1.03711	0.052568
0.6547	314.83	158.62	240.66	737.45	865.7	162.91	0.733731	0.243243	0.386828	0.404352	-1.30632
0.6544	5076.01	10521.42	7159.65	17068.85	5583.87	21565.15	0.706145	0.268379	0.414698	0.514658	-0.95831
0.6537	68799.01	46507.82	42846.4	56335.39	30139.33	22737.67	0.781482	0.281797	0.425625	1.448125	0.534186
0.6535	880810.6	1169089	890311.2	515172.4	523624	398733.6	1.29266	0.020938	0.074446	2.045322	1.032328
0.6533	938.32	1710.72	470.28	2160.58	1971.24	1117.68	0.802623	0.216783	0.356588	0.594213	-0.75095
0.6532	59904.31	20255.88	68597.84	56963.47	52735.61	74328.78	0.522112	0.526524	0.641991	0.808345	-0.30696
0.653	29766.23	18103.96	29936.17	1088.93	14516.15	1017.48	1.085921	0.027452	0.088333	4.680769	2.226746
0.6528	20029.52	39373.45	25689.36	454359.9	79140.12	50261.7	1.002079	0.329715	0.471377	0.145766	-2.77828
0.6523	399878.8	235787.3	274386.7	216273.4	387085	315612.9	0.022291	0.968182	0.976696	0.990295	-0.01407
0.6523	16416.17	16206.17	14259.37	16852.4	15690.85	2312.16	0.581253	0.481566	0.604245	1.345034	0.427643
0.6519	4740.79	16703.72	10493.18	10540.72	3072.06	11398.52	0.322163	0.625499	0.725276	1.27693	0.35268
0.6514	15665.42	263912	5413.27	5728.11	1940.39	3280.21	0.912903	0.392743	0.528919	26.02961	4.702082
0.651	647.24	1446.34	1623.22	4624.12	3882.14	5050.06	1.256481	0.002079	0.020849	0.274175	-1.86683
0.6505	29926.61	22261.86	24848.56	5524.85	7125.62	4896.74	1.348966	0.008313	0.042805	4.390272	2.13431
0.6504	9146.83	8354.52	8250.3	10971.37	10186.34	10495.76	1.284906	0.006408	0.037587	0.813549	-0.2977
0.6504	7789.48	9550.39	5475.28	5366.57	9133.05	7671.5	0.079283	0.90044	0.929878	1.029048	0.04131

0.6503	7908.62	2983.7	4897.66	4675.31	3117.18	3604.01	0.542715	0.417988	0.548303	1.385511	0.470419
0.6502	16587.37	4739.07	18234.81	8931.17	10282.03	7212.98	0.380777	0.41212	0.544373	1.497048	0.58212
0.6501	21923.81	24717.24	39092.3	9798.05	17964.81	24313.33	0.878414	0.177023	0.31077	1.646306	0.719233
0.65	16002.56	3084.84	25681.55	111486.6	121159.2	112742.2	1.205804	0.001103	0.015864	0.129619	-2.94765
0.6492	4558.24	3822.33	1563.06	493.8	286.01	1526.06	1.10465	0.089518	0.196778	4.312312	2.108461
0.649	89275.32	115739.4	92161.42	4224.05	91059.36	120597.3	0.580523	0.522155	0.637914	1.376576	0.461084
0.6485	7745.09	4512.92	5251	1411.99	839.08	1163.73	1.324994	0.037452	0.109861	5.12739	2.358225
0.6482	61152.85	53584.16	225248.2	78100.7	90774.23	83109.57	0.113042	0.652947	0.746107	1.349231	0.432137
0.648	3384.73	1361.94	3261.26	1386.83	2349.58	2522.02	0.376729	0.489003	0.609137	1.279543	0.355629
0.6476	61763.2	54507.11	53989.57	9507.75	14232.06	3616.03	1.268477	0.000343	0.010018	6.223895	2.637818
0.6475	1161.47	1395.52	2538.22	239.47	1136.01	1805.18	0.652569	0.362947	0.50193	1.601935	0.679815
0.6475	7609.89	8783.39	3671.1	1729.7	2556.18	1029.09	1.193559	0.076565	0.176216	3.775069	1.916503
0.647	974.71	715.16	883.14	333.07	423.1	354.87	1.322844	0.01503	0.060326	2.315857	1.211546
0.6468	279223.7	383781.2	322953.6	71739.48	113176.1	94842.12	1.330356	0.008586	0.043773	3.52433	1.817349
0.6464	7819.38	28713.63	41341.64	8446.02	9756.86	17963.29	0.662873	0.288642	0.431386	2.153246	1.106513
0.6461	771825.1	740922.9	872662.4	558126.1	211820.4	614966.8	0.937408	0.107038	0.221526	1.722426	0.784442
0.6461	2974.65	1312.14	1087.28	1953.64	1857.99	1844.95	0.328875	0.888914	0.921557	0.950056	-0.07391
0.646	16596.71	19810.43	13388.17	26514.24	37496.84	26013.59	1.200291	0.050839	0.132936	0.55313	-0.85431
0.6459	1119.99	681.34	1078.76	310.72	89.56	102.93	1.252461	0.015269	0.060838	5.723436	2.516881
0.6455	11954.9	19066.3	8139.6	6552.87	4226.75	6326.02	1.113455	0.142404	0.267099	2.28935	1.194938
0.6452	1040.8	708.89	1296.57	63.91	419.91	1950.72	0.595305	0.763301	0.833474	1.251267	0.32339
0.6448	2060.98	1773.88	1105.26	2342.97	4022.8	502.13	0.055353	0.597171	0.702014	0.719306	-0.47532
0.6448	5035.37	4920.9	2123.61	45334.43	24279.79	42928.25	1.313017	0.035008	0.104233	0.107336	-3.21979
0.6447	9223.67	3250.73	11843.08	12354.6	10302	7187.35	0.475034	0.573889	0.683289	0.814821	-0.29544
0.6447	560230.9	601737.5	437750.4	293710.3	280235	258230.5	1.30723	0.030599	0.094977	1.922333	0.942858
0.6446	4720.04	4016.08	4223.32	4144.07	4169.78	5133.26	0.271899	0.699391	0.784925	0.963734	-0.05329

0.6445	65.26	847.38	1224.41	1067.55	975.8	519.56	0.469077	0.734849	0.813284	0.833837	-0.26216
0.6444	3514.66	2029.46	3481.44	196	281.08	873.21	1.24395	0.021359	0.075509	6.684164	2.740747
0.6441	19134.89	27615.21	21128.14	1644.37	7050.66	6928.39	1.175858	0.007022	0.039121	4.344647	2.119239
0.6437	29885.7	23960.57	25797.41	15444.7	20470.65	16334.06	1.223719	0.017948	0.067156	1.524298	0.608145
0.6427	1764.92	914.27	4126	1990.88	2071	355.38	0.451716	0.522437	0.638079	1.540591	0.623484
0.6427	6144.31	3769.35	3987.21	9138.32	8252.93	8611.45	1.236482	0.023992	0.08081	0.534593	-0.90349
0.6426	7447.02	8342.22	8602.86	102895.4	96444	114581.3	1.371042	0.002908	0.025269	0.077701	-3.68591
0.6422	726272.1	625703.2	818836.7	481450	527142.4	515487.2	1.241208	0.053675	0.137774	1.424343	0.510297
0.6415	43403.31	35202.29	40897.71	172976.3	150733.2	165703	1.365352	0.001005	0.015104	0.244177	-2.034
0.6411	15344.98	15559.89	15693.52	126.09	693.07	2459.37	1.213081	0.001979	0.020492	14.2132	3.829159
0.6408	6317.99	1854.39	14423.62	2445.91	12307.28	3050.02	0.177426	0.759644	0.831355	1.269209	0.34393
0.6407	12495.12	1906.68	12768.1	6048.92	7572.6	8039.89	0.068571	0.660508	0.75213	1.2543	0.326882
0.6406	22901.94	18185.13	26943.75	31071.14	35825.92	28397.58	1.088217	0.054043	0.138329	0.7139	-0.48621
0.6403	26073	21414.89	24667.73	55132.98	58297.67	45348.97	1.333593	0.010714	0.049629	0.454439	-1.13784
0.6401	3150.78	5040.29	3479.96	19387.99	13585.51	13201.52	1.323968	0.021915	0.076179	0.252756	-1.98418
0.6397	219921.3	186087.4	214782.8	397320.6	360797	390350	1.347603	0.000339	0.010018	0.540539	-0.88753
0.6384	4011.22	5137.51	1623.67	7584.71	5757.78	6232.38	0.957552	0.086374	0.191702	0.550318	-0.86166
0.6383	38353.33	25613.45	44886.21	38895.18	34869.34	44862.92	0.387228	0.643955	0.739079	0.917604	-0.12406
0.6382	346.07	2497.98	1982.13	11157.98	301.89	5822.17	0.41604	0.315233	0.457729	0.27926	-1.84032
0.638	264941.3	315400.4	331959.4	559974.6	568170.4	520197.5	1.332499	0.000914	0.014857	0.553466	-0.85343
0.638	14505.36	10038.12	11098.25	4459.83	13333.25	3396.99	0.858742	0.263493	0.408662	1.682002	0.750179
0.6374	523228.8	417128.9	614628.4	608878.6	738183.9	541960.6	0.779149	0.241597	0.384912	0.823169	-0.28074
0.6371	28561.55	26420.94	16678.88	27127.1	24983.47	22381.54	0.238953	0.82717	0.879638	0.961999	-0.05589
0.6371	5517.32	1827.13	4266.09	423.39	455.65	250.9	1.300544	0.083715	0.187424	10.27536	3.361117
0.6371	7727.99	17140.73	12899.39	2750.47	5660.25	6041.89	1.118928	0.089381	0.196575	2.613238	1.385839
0.637	5901.58	5524.92	4566	306.28	695.86	910.97	1.313026	0.002444	0.022956	8.359425	3.063404

0.6369	640559.6	461853.5	549585.3	186166.3	134021	140856.4	1.341599	0.010724	0.049629	3.583171	1.841237
0.6366	4030.78	6912.62	9940.31	81.54	518.17	311.05	1.288337	0.059092	0.147441	22.92998	4.519163
0.6365	522.33	17722.77	4240.76	13954.7	23224.37	7857.13	0.74737	0.337023	0.477732	0.499284	-1.00207
0.6365	19558.27	42821.53	33563.57	7603.67	15625.25	10566.76	1.176788	0.07912	0.180096	2.838924	1.505344
0.6361	20060.86	18482.37	23675.82	29520.05	5619.37	13539.87	0.583781	0.589612	0.695608	1.278142	0.354048
0.6358	351.74	369.5	3463.1	676.96	403.29	670	0.263126	0.515226	0.631823	2.39071	1.257439
0.6357	206962.5	213192.6	225235.9	96371.4	96882.93	95598.91	1.370379	0.001946	0.020396	2.234322	1.159837
0.6355	83809.53	191301.6	78443.19	61654.82	62446.22	57641.31	0.972729	0.259418	0.405016	1.94536	0.960037
0.6351	15772.98	20735.94	15198.89	29589.38	27789.66	34348.38	1.274157	0.007339	0.039748	0.563712	-0.82697
0.635	41691.97	44923.31	47691.38	36160.6	45732.8	34108.23	0.847636	0.226179	0.366511	1.1578	0.211386
0.6346	4261.36	3922.29	2895.25	62370.13	9989.42	5431.91	0.946396	0.347604	0.488603	0.142418	-2.8118
0.6345	1607.73	447.39	949.72	807.53	674.47	1372.7	0.048915	0.907103	0.934103	1.052594	0.073949
0.6343	18382.33	23299.44	15268.58	7621.87	8489.1	8342.51	1.312863	0.041967	0.117782	2.328926	1.219665
0.6341	66.46	1386.65	205.83	144.28	1111.51	1531.9	0.476646	0.556195	0.670736	0.595095	-0.74881
0.6339	8863.42	4971.35	8081.33	3915.38	4325.12	3168.3	1.157246	0.088791	0.195673	1.920982	0.941844
0.6336	4009.84	3819.86	2400.83	1020.54	770.41	3623.73	0.886204	0.218034	0.35795	1.889406	0.917933
0.6332	604.89	499.26	60.36	333.4	1008.14	161.35	0.269906	0.735326	0.813525	0.774847	-0.36802
0.6327	33906.38	49183.36	27779.39	11868.84	8701.31	9867.32	1.315385	0.049335	0.130493	3.642521	1.864937
0.6324	18907.31	16158.3	21973.72	8973.66	9361.65	10213.71	1.32164	0.025452	0.083479	1.997944	0.998516
0.6323	23180.76	25968.58	20766.98	21811.66	15108.58	22833.72	0.689033	0.312326	0.455023	1.17007	0.226595
0.6321	10635.75	33130.3	19730.53	18111.98	30743.22	10645.93	0.081799	0.886852	0.920861	1.067149	0.093762
0.6319	502.45	319.22	823.42	3024.86	2658.34	2077.31	1.28892	0.006935	0.039073	0.211982	-2.23798
0.6316	19487.83	24595.56	16422.37	33319.44	44277.33	43809.32	1.263662	0.012753	0.055095	0.498375	-1.0047
0.6316	10778.52	9478.84	12115.84	756.18	762.42	2687.39	1.281606	0.000807	0.014349	7.696927	2.944283
0.6314	32074.78	42132.44	33556.78	63185.12	59650.74	64775.88	1.310207	0.00532	0.034337	0.574399	-0.79987
0.6309	24815.27	27160.83	24227.37	8961.95	17565.88	11005.91	1.207322	0.027818	0.089227	2.030266	1.021669

0.6308	368195	239212.8	469609.9	229727.8	260887.9	228192.2	0.926371	0.212856	0.352121	1.498339	0.583364
0.6302	13856.94	19548.29	12041.6	30591.43	4098.13	33971.21	0.086443	0.50162	0.620478	0.661904	-0.59531
0.6299	4652.95	66279.89	45729.65	18728.24	18923.9	8290.98	0.39459	0.321998	0.464214	2.539281	1.34442
0.6297	3397.88	9684.85	2813	6195.42	2017.14	4255.82	0.246424	0.678845	0.767596	1.274883	0.350365
0.6295	88063.93	71789.51	20335.46	137999.7	414951	196599.8	1.097657	0.146607	0.272404	0.240396	-2.05652
0.6295	42919.49	24049.73	30459.55	3903.61	5713.15	3429.7	1.338825	0.034908	0.104007	7.467832	2.900689
0.6293	153933	267768.4	307414.7	264969.8	218561.1	210480.7	0.041577	0.829333	0.880434	1.050582	0.071189
0.6292	2639.98	1526.22	2361.52	2159.9	2669.54	1211.75	0.241036	0.78072	0.845901	1.080535	0.111746
0.6292	227.24	492.87	1097.31	4685.48	4992.15	4089.35	1.268271	0.000422	0.010815	0.132013	-2.92125
0.6285	60649.14	58866.9	59933.34	137423.3	67573.75	105875.3	1.063008	0.162218	0.292189	0.577245	-0.79275
0.6285	4442.07	4223.42	5763.87	1233.14	1880.43	1704.4	1.318681	0.012431	0.054133	2.994904	1.58251
0.6283	576979.6	839251.1	67263.94	11165.84	8613.66	16548.55	1.23273	0.167077	0.297869	40.83607	5.351772
0.628	23344.53	714.7	2115.45	7805.18	6327.01	2483.18	0.257657	0.708796	0.792622	1.575329	0.655653
0.6279	3236.27	3962	1378.63	26116.64	20570.01	21622.99	1.312953	0.002431	0.022937	0.125559	-2.99356
0.6274	27064.25	22354.89	24849.96	48169.9	12456.19	13780	0.305509	0.997248	0.997935	0.998159	-0.00266
0.6273	349.83	810.82	2377.25	102.5	278.69	42.78	1.10625	0.231645	0.372873	8.344694	3.060859
0.6272	55735.56	12492.21	12801.11	23846.08	65547.97	42274.3	0.69117	0.420484	0.550583	0.615401	-0.7004
0.6269	184340	167048.9	176426	134104.6	155257.8	142680.6	1.223524	0.017032	0.065069	1.221672	0.288857
0.6268	1594.38	1135.06	701.06	10205.89	8642.17	10326.37	1.340159	0.000952	0.014975	0.117586	-3.08821
0.6266	123435.3	170865.3	109786.3	44037.64	24009.95	68748.16	1.189411	0.020906	0.074391	2.953943	1.562642
0.6263	5031.07	11423.38	7815.03	31766.73	24868.08	11304.62	1.078746	0.126461	0.246642	0.357222	-1.48511
0.6258	10953.08	18461.97	9887.9	1451.41	165.07	9385.65	0.94865	0.075528	0.174412	3.572304	1.836855
0.6254	42441.5	44735.76	52717.88	1419.04	13109.85	3204.05	1.214092	0.001161	0.015971	7.888999	2.979842
0.6252	1808.17	1029.66	1255.35	619.81	1414.38	520.66	0.843129	0.235775	0.377874	1.602121	0.679984
0.6243	3744.61	2003.11	5272.62	3642.2	2966.62	8041.9	0.413242	0.556408	0.670736	0.752205	-0.4108
0.6241	21547.2	19431.62	18657.36	30223.02	24387.37	25380.38	1.207942	0.045525	0.123794	0.745538	-0.42365
0.6227	32969.3	20661.68	21936.84	41502.28	41205.1	41353.11	1.195024	0.0537	0.137774	0.609121	-0.7152

0.6226	9731.92	7465.81	9708.04	11852.85	10897.09	11260.58	1.094379	0.073446	0.171071	0.791101	-0.33807
0.6226	122185.2	149760.2	167747.3	16532.47	113063.3	92348.78	0.84189	0.116365	0.233919	1.981093	0.986296
0.6219	3238.83	2411.5	1122	1251.19	109.66	490.05	0.992396	0.098747	0.209868	3.658939	1.871425
0.6216	11731.69	17619.7	6467.48	4093.49	5488.33	5219.74	1.096681	0.159482	0.288808	2.419939	1.274971
0.6216	22468.95	30200.27	29056.72	9524.55	11461.72	3286.74	1.178237	0.00515	0.033706	3.366947	1.751441
0.6215	1729.41	1072.97	852.71	479.14	1013.85	571.85	0.940825	0.176666	0.310519	1.770157	0.823877
0.6211	2412.67	1961.86	2555.97	8931.92	7495.8	8994.34	1.356922	0.002815	0.024891	0.272618	-1.87505
0.6209	8686.08	9097.79	8543.25	3233.48	2227.4	4365.56	1.282206	0.008656	0.044029	2.679212	1.421809
0.6209	379707.3	350840.6	273349.6	64942.19	137751.5	466794.3	0.721247	0.465175	0.590613	1.4995	0.584482
0.6198	9399.53	6909.18	8659.31	1537.66	1884.03	1741.34	1.359721	0.011156	0.050922	4.835924	2.273792
0.6195	823.97	1114.18	2879.36	342.68	2111.18	2109.84	0.174376	0.927377	0.948261	1.055615	0.078084
0.6192	1201.97	1611.42	575.73	1009.84	284.04	207.56	0.891458	0.188267	0.323507	2.257246	1.174564
0.6191	75224.31	35942.61	61606.3	42995.02	34535.26	47990.94	0.695401	0.30366	0.446125	1.376446	0.460948
0.6191	15678.21	5544.23	7978.95	11486.09	10148.42	6104.84	0.016406	0.896687	0.927102	1.052706	0.074103
0.618	235923.3	260450.3	333939	213392.7	208024.6	201816.7	1.095599	0.142064	0.266749	1.332265	0.413881
0.6179	1809.83	2099.46	2597.3	9744.5	1538.35	8884.9	0.795323	0.22171	0.362096	0.322623	-1.63208
0.6178	83.9	72.53	53.97	45.52	25.96	9.38	1.046508	0.035264	0.104742	2.602028	1.379637
0.6175	182231.9	206056.5	219859.1	216533.6	203952.7	275784	0.709128	0.322149	0.464214	0.873436	-0.19523
0.6175	31608.12	41250.85	43910.94	14924.32	16917.6	16541.76	1.332989	0.02339	0.079698	2.413415	1.271076
0.6174	757.63	553.73	2390.35	21563.47	22443.75	17195.8	1.314348	0.003394	0.027425	0.060482	-4.04734
0.6171	6744.06	5094.99	4426.68	329.51	2150.15	523.25	1.189201	0.008564	0.043718	5.416656	2.437402
0.617	2626.18	3709.43	2311.16	6932.16	6546.24	4137.38	1.171404	0.057007	0.144054	0.490854	-1.02664
0.617	317.59	271.26	247.79	258.29	608.54	1194.02	0.863511	0.273172	0.417956	0.405968	-1.30056
0.6169	258180.8	234564.2	209027.3	199746.2	169967	198313.1	1.089251	0.068459	0.16289	1.235457	0.305045
0.6169	6591.75	8976.59	6426.89	10001.85	6756.09	4830.4	0.148204	0.941945	0.95866	1.018848	0.026938
0.6167	109489.6	117445.6	70438.93	33121.53	43777.18	41757.09	1.272679	0.048629	0.129488	2.506191	1.325496

0.6166	39349.23	42967.22	46492.41	108925	112360.7	115367.4	1.365197	1.68E-05	0.003653	0.382616	-1.38603
0.6162	1588.85	582.47	145.74	228.13	268.1	620.76	0.366331	0.451586	0.57896	2.074378	1.052679
0.6162	25580.26	22886.49	27714.22	35319.42	43364.45	38579.67	1.284496	0.012286	0.053878	0.649656	-0.62225
0.6157	2841.69	2800.79	2906.44	8981.14	3094.62	63005.7	0.869227	0.364748	0.50367	0.113862	-3.13464
0.6154	2502.57	1850.68	1819.75	1539.76	321.33	236.5	1.058864	0.06403	0.156012	2.942901	1.557239
0.6152	13723.79	15569.62	29736.92	6041.75	5614.74	7426.17	1.242332	0.117429	0.235019	3.093402	1.629194
0.6152	331.36	77.11	285.4	114.05	385.84	216.49	0.105363	0.94951	0.96329	0.968578	-0.04606
0.6151	589.81	699.73	38.97	470.21	264.21	415.17	0.275879	0.80259	0.862023	1.155638	0.20869
0.6151	20056.69	15293.15	19688.88	12276.54	1505.12	10297.34	0.889634	0.071223	0.167184	2.285756	1.192671
0.6145	36100.13	35645.87	36691.82	4929.25	3388.48	3431.57	1.364855	5.96E-06	0.003019	9.2293	3.206221
0.6138	1302.66	2725.12	1154.45	20455.21	21021.82	17928.8	1.341741	0.000432	0.011008	0.087234	-3.51896
0.6138	90857.32	89939.29	87966.45	136240.5	122819.6	132898.7	1.352664	0.007418	0.039933	0.685692	-0.54437
0.6137	51832.01	26334.97	49774.83	16443.93	9156.66	14342.05	1.233118	0.061292	0.150945	3.203139	1.679486
0.6135	25053.52	29744.3	31804.07	3284.79	1142.15	3940.8	1.30809	0.002037	0.020785	10.3495	3.371489
0.6133	59849.81	72533.36	69569.59	26718.27	26237.67	25836.56	1.362298	0.008372	0.042934	2.563096	1.357888
0.6133	57237.04	10752.41	68316.06	44924.42	17825.31	32951.31	0.163887	0.537574	0.652544	1.424285	0.510237
0.613	65.89	87.21	995.11	24.37	236.06	51.5	0.566568	0.460546	0.586871	3.680986	1.880092
0.613	72617.8	69002.44	73315.61	103473.2	98809.05	86641.35	1.28098	0.031932	0.0978	0.743919	-0.42678
0.6129	2739.66	3074.27	423.79	2096.92	1990.63	2665.96	0.3867	0.858056	0.900185	0.923626	-0.11462
0.6128	290731.8	249368	252732.3	15362.46	14067.41	10431.85	1.368101	0.002515	0.023404	19.88956	4.31394
0.6124	7559.33	27614.31	16690.37	41351.51	2805.28	6346.97	0.388593	0.9756	0.981305	1.026934	0.038343
0.6123	15349.27	23313.17	14229.15	25958.25	32933.32	28033.66	1.149615	0.037076	0.109052	0.608472	-0.71674
0.6118	172.95	2179.38	396.99	535.81	1049.75	615.3	0.244045	0.803607	0.862445	1.249203	0.321007
0.6115	22789.46	17439.91	21142.4	6370.9	8366.26	6659.55	1.340729	0.007028	0.039121	2.868281	1.520186
0.6113	9575.47	3250.54	10352.14	4099.15	5381.05	6050.82	0.482592	0.375241	0.51259	1.492378	0.577613
0.6113	0.03	0.03	0.23	0.03	0.03	0.03	0.615396	0.42265	0.551654	3.222222	1.688056
0.6111	1530844	706408.9	1068936	417865.3	317724.5	502425.7	1.204207	0.095333	0.205315	2.670555	1.41714

0.611	15787.17	15693.61	15706.73	14988.66	11759.08	14837.37	0.880702	0.217848	0.357935	1.134721	0.182338
0.611	737.12	318.58	522.8	165.33	18.84	422.82	0.853108	0.12778	0.248657	2.600537	1.37881
0.6108	97768.25	22685.18	131928	90224.73	88695.33	71907.45	0.275621	0.988753	0.991265	1.006195	0.00891
0.6103	16202.46	13040.45	15320.5	18252.11	18458.15	14031.36	0.679264	0.307734	0.451047	0.878242	-0.18731
0.6103	40619.51	40112.86	41512.08	9513.21	11578.88	564.31	1.053113	0.009225	0.045392	5.644726	2.496904
0.6102	711.11	63.1	543.78	653.41	1030.07	1664.17	0.847845	0.138908	0.26352	0.393706	-1.34481
0.6101	3437.79	3086.42	3165.45	10185.98	12135.11	9754.79	1.362505	0.008422	0.043088	0.302086	-1.72697
0.61	292.56	561.37	71.74	383.43	75.56	7327.81	0.428637	0.436066	0.563704	0.118877	-3.07246
0.6089	7056.77	9729.04	9921.33	17116.28	12570.78	14454.89	1.196554	0.027195	0.087875	0.605029	-0.72492
0.6086	938.4	3953.31	4636.51	14722.82	2803.63	1895.5	0.40762	0.512788	0.630178	0.49059	-1.02741
0.6085	5211.98	3379.49	683	18612.92	25143.21	27821.7	1.208586	0.007281	0.039627	0.129572	-2.94818
0.6082	30768.78	26063.24	23387.3	12273.27	15476.99	11681.81	1.302927	0.010712	0.049629	2.034367	1.02458
0.6076	94974.47	208290.5	95529.1	67836.99	88619.61	46959.04	0.967221	0.220187	0.360448	1.960489	0.971213
0.6069	55006.92	70349.15	91641.15	74841.54	75976.36	77711.57	0.3291	0.752425	0.826151	0.949537	-0.0747
0.6063	26178.92	122050.3	121429.3	56513.83	30370.83	59231.61	0.512961	0.324565	0.46677	1.845506	0.884016
0.606	1371.91	634.76	2577.52	1053.86	158.92	8943.39	0.074961	0.576734	0.685195	0.45137	-1.14762
0.6059	78711.22	23507.83	59360.89	69035	71461.74	88880	0.721847	0.296374	0.43927	0.70443	-0.50547
0.6053	21263.71	17236.53	10061.44	34994.16	36522.61	30609.01	1.187313	0.016281	0.063099	0.475509	-1.07246
0.6046	1497.3	3037.46	9441.96	7997.61	9601.74	5797.02	0.786434	0.330286	0.4717	0.597388	-0.74326
0.6045	17590.51	9600.86	26712.65	1826.82	6998.42	1251.73	1.157113	0.083916	0.187569	5.349229	2.419331
0.6044	14431.53	15886.75	15429.48	16858.66	24020.62	27657.41	1.082905	0.136531	0.259999	0.667493	-0.58318
0.6043	14600.09	8906.18	9612.35	23570.54	18311.24	19831.5	1.212072	0.0166	0.063879	0.536653	-0.89794
0.6028	13908.1	21907.34	20189.15	21577.87	28660.25	18572.15	0.661355	0.332554	0.474036	0.813899	-0.29708
0.602	2278.02	3232.33	1021.27	171.46	645.47	157.26	1.195463	0.093746	0.203141	6.704667	2.745166
0.602	140.81	608.03	232.39	341.45	211.2	519.67	0.28239	0.867414	0.906542	0.915053	-0.12807
0.6018	111116.8	72792.95	111155.4	107235.1	79582.82	80361.64	0.350065	0.588514	0.695326	1.104371	0.143224
0.6014	3241.62	8187.76	3338.89	2618.75	1975.45	1930.56	1.031832	0.23289	0.374463	2.26342	1.178504

0.6013	1815.94	3012.13	11287.86	10688.52	12322.31	6313.93	0.819106	0.287745	0.430877	0.549567	-0.86363
0.601	291457	303047.3	207665.6	108746.1	127772.2	107372	1.308428	0.031665	0.097271	2.332633	1.221959
0.6008	14018.89	18791.18	19407.79	39196.74	28484.24	24319.82	1.162376	0.081089	0.183048	0.56758	-0.8171
0.6007	159748	146076.3	146702.5	3149.98	5338.04	4306.14	1.367673	0.000751	0.014105	35.3698	5.144446
0.5999	599.78	1613	1490.05	4320.99	6272.89	7189.23	1.260908	0.020361	0.073048	0.208222	-2.26381
0.599	7229.53	17744.32	8977.06	4532.53	3157.96	5266.8	1.133949	0.160281	0.289781	2.620217	1.389686
0.5985	112623.2	148753.3	96709.21	63484.36	44234.85	74973.66	1.18265	0.041928	0.117759	1.960042	0.970885
0.5982	2172.64	5203.26	2779.49	9501.06	4764.33	5153.81	0.962513	0.172296	0.305424	0.522956	-0.93524
0.5966	63146.85	73196.64	71521.82	25279.14	3010.67	39701.03	0.962312	0.03972	0.113237	3.057255	1.612237
0.596	1663.96	1906.19	2201.95	5117.19	3895.1	4525.03	1.326657	0.008833	0.044309	0.426384	-1.22977
0.5958	733326.1	636110	792078	530253.1	338851.2	400917.8	1.211699	0.016202	0.063093	1.70195	0.767189
0.5952	21053.31	13471.83	30456.45	25098.81	21947.45	23617.17	0.375905	0.738999	0.816345	0.919593	-0.12093
0.5947	66978.34	51817.82	38291.08	60936.03	2652.55	92881.26	0.419311	0.994644	0.995786	1.003946	0.005681
0.5945	1022789	2634167	1994976	4168439	1754962	2413565	0.636805	0.365252	0.503999	0.677936	-0.56078
0.594	9541.86	13701.3	9546.74	16465.52	8906.58	7966.17	0.060161	0.955641	0.967853	0.983551	-0.02393
0.5934	52494.45	50885.73	32352.72	44930.14	46929.53	88072.45	0.591602	0.415565	0.54677	0.754356	-0.40668
0.5929	646.71	1856.99	25529.77	18522.56	19916.27	120.2	0.040221	0.751651	0.825717	0.727027	-0.45992
0.5927	1986.55	1280.31	3097.87	251.09	406.47	102.49	1.260541	0.067976	0.162094	8.374094	3.065933
0.5926	6457.23	1670.58	3288.39	3732.87	3092.38	2767.05	0.059678	0.710092	0.793489	1.190142	0.251134
0.5919	19072.96	16749.98	16667.81	18529.67	39455.99	42725.23	1.013924	0.166703	0.297567	0.521202	-0.94008
0.5915	3092.65	7731.08	777.76	427.76	1119.47	441.04	0.975102	0.256637	0.402242	5.834967	2.544725
0.5915	33335.38	36575.13	30666.02	43226.36	40334.5	29983.69	0.566457	0.402564	0.536649	0.885789	-0.17496
0.5914	1865.59	1721.4	2639.14	1067.4	798.04	854.87	1.281655	0.045573	0.123847	2.288758	1.194565
0.5904	13323.27	15563.51	19751.77	48653.7	59998.81	64297.84	1.340031	0.005966	0.036092	0.281228	-1.83019
0.5889	289462	218037.2	180051.3	86491.51	86653.09	89363.74	1.317037	0.047504	0.127585	2.619157	1.389103
0.5885	47034.57	66324.66	72784.64	13595.23	13857.18	57565.12	0.980934	0.133032	0.255232	2.189476	1.130586

0.5884	10879.29	12630.34	15666.42	3135.13	1787.65	1361.22	1.321104	0.008678	0.044089	6.234254	2.640217
0.588	18850.2	18251.83	6764.51	23760.24	26891.43	25138.46	0.947763	0.107048	0.221526	0.57879	-0.78889
0.5876	2803.7	1832	2954.52	751.06	773.62	1007.28	1.304633	0.035135	0.104471	2.997765	1.583887
0.5873	29704.55	32248.51	34723.56	46802.55	31230.76	34761.63	0.657162	0.374025	0.51157	0.857101	-0.22246
0.587	19287.88	13577.51	12689.17	2260.44	4060.94	5274.02	1.27393	0.019382	0.070615	3.928675	1.974043
0.5868	679.14	171.51	1000.29	1320.72	1040.81	980.97	0.824474	0.164203	0.294794	0.553759	-0.85267
0.5867	11079.69	5571.79	13393.78	59094.06	33923.49	21128.92	1.172175	0.123011	0.242516	0.263217	-1.92568
0.586	3833.21	4553.6	3029.9	12835.99	17821.85	16431.04	1.342203	0.010303	0.048711	0.24245	-2.04424
0.586	6349.61	2426.45	4234.28	4010.17	1727.49	1725.27	0.798776	0.256531	0.402242	1.743329	0.801845
0.5859	648.66	950.41	2879.31	1164.95	1592.49	490.17	0.265565	0.632958	0.730623	1.378977	0.463599
0.5857	283.31	10.13	142.08	1990.89	2856.54	1414.85	1.162684	0.039483	0.112856	0.069547	-3.84588
0.5853	21830.7	41809.06	14710.12	44449.82	7585.05	7494.47	0.53076	0.695419	0.781409	1.316156	0.39633
0.5841	274547.8	252083	295176.3	11396.62	32023.8	36482.06	1.31347	0.000237	0.009207	10.28513	3.362488
0.5839	8770.47	13765.45	9554.75	12437.89	479.42	13989.99	0.502482	0.733369	0.812322	1.192638	0.254156
0.5834	128720.9	187151.3	128682.5	441871.9	194889.9	419680.7	1.116695	0.115279	0.23264	0.420803	-1.24878
0.5834	15589.97	19176.9	19615.81	17883.15	13005.46	15092.74	0.809381	0.215867	0.355752	1.182712	0.242098
0.5831	2276.83	1275.27	5501.41	6183.58	2671.52	2976.51	0.480131	0.615173	0.717756	0.765197	-0.3861
0.583	74474.66	28426.1	46470.85	85349.68	49970.61	29673.4	0.13831	0.817418	0.87246	0.905317	-0.1435
0.5824	22119.1	20167.79	19565.86	6136.21	8445.54	8522.97	1.33774	0.0003	0.009591	2.677061	1.42065
0.5822	2158968	1812926	3095272	2622297	2834958	2400952	0.500112	0.569608	0.679879	0.899336	-0.15307
0.5816	112.18	1680.75	746.2	1259.08	2163.44	735.73	0.627372	0.431876	0.559117	0.610625	-0.71164
0.5815	36633.16	45904.36	40077.61	25177.76	17338.58	18067.48	1.284836	0.005044	0.033561	2.023892	1.017133
0.5808	4293.41	2852.27	2819.61	12904.35	10467.22	11070.98	1.334689	0.001427	0.01714	0.289331	-1.78921
0.5801	422.19	176.19	642.43	571.38	146.19	309.29	0.253553	0.716673	0.798971	1.208354	0.273043
0.5798	12345.44	9607.5	7701.87	16177.55	15245.35	16748.4	1.202334	0.034344	0.102935	0.615612	-0.69991
0.5797	159.32	46.06	47.02	171.72	183.66	250.87	1.064155	0.068108	0.16232	0.41633	-1.2642
0.5797	583131.3	713913.4	945446.4	54153.72	796351.2	699549.8	0.596113	0.437999	0.565657	1.446717	0.532783

0.5794	2847.99	289.24	1285.59	36291.57	7825.84	1898.39	0.949252	0.321363	0.464001	0.096115	-3.37909
0.5789	88545.63	152033.1	129060.9	47269.18	19677.87	21751.07	1.261459	0.021861	0.076054	4.16739	2.059144
0.5788	906.73	823.22	291.49	349.5	531.07	1496.83	0.100732	0.788109	0.85093	0.850273	-0.234
0.5787	2295.47	5220.76	2508.64	267.22	432.92	1710.8	1.106386	0.096956	0.207365	4.158075	2.055916
0.5781	1979.59	3683.17	3063.09	1057.47	8005.14	7747.3	0.344791	0.357625	0.498387	0.51909	-0.94594
0.5776	902.61	1443.67	1038.68	4521.36	1838.46	749.66	0.586679	0.38338	0.520284	0.476119	-1.07061
0.5771	25272.43	9820.65	11379.15	27344.63	16330.08	14618.59	0.507238	0.568476	0.679087	0.797214	-0.32696
0.5766	21321.14	18999.22	19467.27	14196.77	12914.07	14114.09	1.331578	0.003759	0.028827	1.450279	0.53633
0.5759	6291.1	2269.11	1245.55	37909.3	33820.07	17425.03	1.253954	0.044816	0.122632	0.109986	-3.1846
0.5755	13492.4	12587.17	10324.51	20250.83	12300.78	13752.64	0.750167	0.309612	0.453191	0.786193	-0.34704
0.5753	118449.2	138876.9	124345.8	320535.1	327013.7	345341.8	1.364928	3.94E-05	0.004343	0.384405	-1.3793
0.5752	4876.81	6170.93	3739.66	858.53	2861.34	1001.61	1.158241	0.024773	0.082461	3.131942	1.647057
0.575	426653.6	359377.2	400251.9	64760.68	66868.49	63073.55	1.370579	0.003404	0.027425	6.09279	2.607103
0.5746	41526.6	30703.67	42295.7	9491.11	9457.11	9544.75	1.357711	0.016603	0.063879	4.019447	2.006997
0.5745	1345.81	782.43	1110.15	6764.46	2269.52	1482.71	0.98555	0.277673	0.42144	0.307929	-1.69933
0.574	2492.45	1552.3	2778.06	2660.43	2380.09	1709.68	0.006226	0.96119	0.972095	1.010757	0.015436
0.5739	1003.3	284.83	910.07	1221.8	474.99	1481.47	0.478357	0.438355	0.565657	0.691636	-0.53191
0.5736	9818.42	7466.95	7328.81	3377.39	4768.33	4733.78	1.249124	0.021967	0.076299	1.911113	0.934413
0.5731	45006.39	34479.74	26488.01	17853.94	29335.31	21515.2	0.980862	0.134715	0.256991	1.542464	0.625237
0.5728	35209.86	49643.5	35321.44	9204.29	11192.65	8634.49	1.347382	0.021685	0.075985	4.139472	2.049447
0.5722	189783.8	367901.5	160904.9	93939.37	95864.93	80534.07	1.194511	0.146546	0.272404	2.658114	1.410403
0.5717	36731.77	33573.48	33228.65	34464.71	31962.54	84151.87	0.548968	0.453665	0.58059	0.687571	-0.54042
0.5713	139815.8	87043.42	150772.5	117681	536087	132680.6	0.604463	0.425195	0.553083	0.480173	-1.05837
0.5707	183272	162490.5	110696.6	86162.6	198880.9	146957.3	0.20963	0.846369	0.890926	1.056617	0.079452
0.5705	30198.55	25389.95	36932.82	34489.44	11962.99	25464.72	0.611395	0.419552	0.550024	1.286499	0.36345
0.5705	4710.81	1893.42	4476.52	1318.56	935.12	3866.19	0.772775	0.268755	0.414838	1.810619	0.856483

0.5701	7590.58	4861.01	5200.96	8919.71	12380.82	9095	1.160606	0.043532	0.120404	0.580761	-0.78398
0.5701	6720.92	12811.06	6836.44	476.97	515.04	298.38	1.350876	0.053146	0.13716	20.43446	4.352932
0.5696	61955.58	2180.57	5080.67	40853.86	39899.2	43776.68	0.838448	0.443479	0.571086	0.555826	-0.8473
0.5695	17304.84	14955.61	15991	1628.2	1921	1728.47	1.370579	0.001958	0.020396	9.142567	3.192599
0.5668	64972.5	76095.81	89275.4	888290.7	841654.3	880176.6	1.368805	2.43E-05	0.003778	0.08825	-3.50226
0.5665	3552.91	1065.57	2346.81	3166.83	1344.93	2213.93	0.031238	0.933199	0.951758	1.035625	0.050501
0.5649	7783.22	919.29	10326.1	890.24	13161.55	7383.39	0.032856	0.868266	0.906749	0.887728	-0.17181
0.5648	527.49	585.44	211.99	1870.17	58.75	2070.16	0.231345	0.296876	0.439503	0.331306	-1.59376
0.5631	4162.54	208.08	721.05	2926.74	620.26	890.63	0.217705	0.888569	0.921557	1.147385	0.19835
0.5624	273.94	2101.66	964.83	2513.15	1063.67	1752.45	0.662496	0.386157	0.522751	0.626808	-0.6739
0.5618	202196.2	229261.7	181155.4	288939.3	282241.2	248380.8	1.19622	0.021606	0.075892	0.747489	-0.41988
0.5618	21819.47	22265.44	20508.14	29965.57	27907.34	25655.47	1.274192	0.02342	0.079698	0.773307	-0.37089
0.5616	57219.29	56338.92	43051.91	71385.6	66745.13	67263.03	1.136356	0.059683	0.148242	0.762487	-0.39121
0.5613	9158.1	4413.91	8270.8	13202.13	40450.02	20552.12	1.133974	0.161501	0.291262	0.294361	-1.76434
0.5612	103298.6	19159.01	99656.43	776.11	1626.06	1973.76	1.302848	0.118253	0.235802	50.75812	5.665567
0.5604	13159.35	21427.85	20948.16	16828.23	17151.15	17385.22	0.232321	0.655824	0.747686	1.081199	0.112632
0.5602	3996.1	3526.5	4495.66	3392.22	5323.68	3416.79	0.036704	0.960175	0.971767	0.990568	-0.01367
0.5586	54321.64	33082.37	54320.82	61103.03	40992.98	129032.6	0.645877	0.380492	0.517654	0.613186	-0.7056
0.5583	45.21	41.22	3.09	28.77	148.31	54.08	0.720501	0.324425	0.466723	0.387264	-1.36861
0.5583	16759.11	32444.44	21069.25	25112.71	16497.74	17629.89	0.420897	0.542103	0.656944	1.186232	0.246386
0.5568	4830.65	5534.48	5579.81	42463.63	37859.39	44552.28	1.369961	0.002617	0.023767	0.127687	-2.96932
0.5568	42831.6	51466.99	42324.06	28501.82	23240.48	26284.56	1.311448	0.010042	0.047891	1.750969	0.808154
0.5567	369486.8	489992.6	357319.6	397501.1	430080.5	424126.6	0.249824	0.811931	0.867878	0.972111	-0.04081
0.5564	19488.29	2272.12	5707.92	64171.85	74809.25	74440.64	1.220185	0.001181	0.015971	0.128704	-2.95787
0.5557	29036.25	19401.01	25885.11	15286.03	4066	18090.39	0.9054	0.084816	0.188822	1.984978	0.989123
0.5556	77.19	179.05	63.87	76.53	53.24	30.02	0.850349	0.277176	0.42144	2.003317	1.002391

0.5552	52316.58	277976	26120.4	29390.74	28203.85	17624.11	0.825827	0.361728	0.500881	4.738356	2.244386
0.5549	3158.84	1127.23	1151.82	2295.82	68.09	318.76	0.776284	0.399393	0.533872	2.027044	1.019377
0.5535	2848.58	3647.18	1572.66	10258.33	5189.29	14353.39	1.173522	0.10525	0.218886	0.270743	-1.885
0.5535	416.13	79639.79	37447.72	895.86	514.05	94200.25	0.320712	0.860264	0.901416	1.228987	0.29747
0.5528	10337.58	2315.97	2010.43	39519.01	43003.25	47331.54	1.263871	0.000491	0.01162	0.112927	-3.14654
0.5526	6987.05	2696.41	5365.32	54.72	85.12	165.81	1.341024	0.059002	0.147387	49.23533	5.621622
0.5523	1653.86	1674.86	1700.18	2319.84	206.68	1913.25	0.455619	0.790288	0.852268	1.132694	0.179758
0.5498	12286.34	11449.61	13650.51	1492.53	2097.51	3421.19	1.320241	0.000319	0.009924	5.332368	2.414776
0.5498	36229.12	34029.4	29390.95	34454.91	30301.22	37766.78	0.215839	0.762075	0.832344	0.971973	-0.04101
0.5491	4903.36	3202.02	2054.84	918.96	2881.77	1037.83	0.934541	0.169042	0.301249	2.099844	1.070282
0.5489	2232.66	1975.3	2164.44	818.7	815.92	650.13	1.354882	0.000245	0.009295	2.789102	1.479801
0.5487	8962.31	9105.91	13365.35	11248.14	11452.58	6463.22	0.266711	0.746064	0.821007	1.077823	0.10812
0.5484	2142.61	4981.99	7100.39	4987.75	6561.4	3068.94	0.151184	0.944478	0.960599	0.973109	-0.03933
0.5476	128.66	70.23	39.09	32.5	33.7	359.32	0.030094	0.627289	0.726835	0.559269	-0.83839
0.5454	1957.38	3385.88	3121.05	33394	37880.28	33090.75	1.359752	0.001216	0.016008	0.081103	-3.6241
0.5454	38324.51	62721.48	33603.64	54082.54	46738.06	57996.76	0.610524	0.473627	0.597047	0.847827	-0.23816
0.5447	1556.92	5825.16	1749.35	10757.08	4800.18	295.51	0.008652	0.552716	0.667208	0.576015	-0.79582
0.5435	17950.38	25485.73	22081.22	54305.29	50540.51	50324.34	1.334736	0.000884	0.01464	0.422229	-1.2439
0.5434	135686	113357.6	151513.6	83875.13	95391.78	92624.57	1.234251	0.049946	0.131232	1.473225	0.558977
0.5432	4007.76	31.55	2311.53	144.37	1469.28	87.62	0.346523	0.311167	0.45421	3.732999	1.900335
0.5414	1172.31	208.37	456.06	2453.28	2494.97	103.67	0.329851	0.307367	0.45066	0.363573	-1.45968
0.5396	495.02	613.99	217.88	241.9	773.78	222.81	0.203582	0.898798	0.928563	1.071377	0.099467
0.5394	1768.17	622.35	2126.67	528.86	170.13	425.48	1.09675	0.123533	0.243107	4.017173	2.00618
0.5376	57867.16	47316.49	43942.65	58267.01	63694.5	52878.41	0.877649	0.18231	0.317494	0.85293	-0.2295
0.5375	5019.76	5363.87	5131.8	1549	2172.26	205.85	1.055043	0.019302	0.070468	3.950852	1.982164
0.5364	726.29	2083.21	2370.92	3041.97	6881.8	1666.51	0.772488	0.303054	0.445836	0.446962	-1.16177

0.5363	27970.86	21189.32	24341.64	28129.12	32354.03	28051.83	0.982338	0.113903	0.230506	0.830201	-0.26847
0.5359	674.78	488.65	133.15	203.27	203.87	194.49	0.677865	0.282177	0.426051	2.155112	1.107763
0.5353	19436.66	4852.42	12211.28	33702.36	33815.05	33793.19	1.126424	0.035937	0.106419	0.360282	-1.4728
0.5352	10923.99	10209.31	8289.57	11402.26	12416.56	12459.46	1.064556	0.08392	0.187569	0.811033	-0.30217
0.5352	2621.63	1879.64	1510.75	826.33	498.37	100.43	1.095132	0.023007	0.078782	4.218577	2.076756
0.535	3487.15	1842.4	2639.37	26510.23	22969.88	20963.62	1.351604	0.003509	0.02771	0.113125	-3.14402
0.5347	70.39	51.95	13.4	35.49	18.5	27.59	0.399184	0.396758	0.531969	1.663888	0.734559
0.5345	2539.34	1063.99	1762.56	879.14	468.33	766.97	1.124843	0.116662	0.234237	2.537736	1.343542
0.5341	1341236	2925130	1545957	274716.3	1830648	596388.8	0.879027	0.206007	0.344715	2.151315	1.105219
0.534	14769.86	9037.79	5036.84	8369.77	9544.42	9792.41	0.111573	0.90608	0.933271	1.041069	0.058066
0.534	14936.13	14835.86	14875.45	19991.1	20128.52	19917.6	1.372532	8.77E-06	0.003019	0.743663	-0.42728
0.5336	1102.78	213.58	625.9	1826.65	1198.52	1759.08	1.01088	0.046797	0.126387	0.40597	-1.30056
0.5334	460376.8	448635.7	491324.1	237515.6	254614.7	279494.8	1.350686	0.00029	0.009591	1.814789	0.859802
0.5322	3281.36	3532.13	1579.87	9129.1	9637.72	9269.31	1.272391	0.006121	0.036597	0.299377	-1.73997
0.5319	1125.72	1170.03	587.08	1019.61	468.69	375.48	0.745646	0.284314	0.427353	1.546765	0.629254
0.5315	1981.28	1342.44	1986.98	10875.63	10570.35	11213.93	1.359011	6.68E-06	0.003019	0.162606	-2.62055
0.5308	6542.45	5113.4	6252.63	1403.19	4225.7	112	0.913205	0.065617	0.158727	3.119461	1.641297
0.5307	186.83	301.1	279.45	13222.29	20963.47	438.94	1.059492	0.19995	0.337351	0.022163	-5.49572
0.5302	21912.07	52835.1	53162.86	28728.01	95440.82	48392.07	0.371364	0.552637	0.667208	0.741246	-0.43198
0.5294	362274.8	111621.9	109175	170641.4	139253.6	273391.3	0.19857	0.999438	0.999438	0.999632	-0.00053
0.5293	2337.84	242.35	4375.66	297.83	3369.28	1094.68	0.164319	0.654357	0.746978	1.460764	0.546723
0.5291	14024.13	4749.19	3063.74	39.35	2470.85	1493.23	0.902903	0.219761	0.359992	5.454588	2.44747
0.5283	23153.97	67514.39	36870.98	22639.08	19582.02	18327.98	0.987066	0.229855	0.370951	2.106379	1.074765
0.5279	10598.59	5837.62	8330.67	18436.73	18555.68	21152.86	1.272184	0.004237	0.030581	0.425948	-1.23125
0.5278	70685.48	53978.84	84233.2	77895.36	98740.49	74151.15	0.714351	0.296934	0.439503	0.832968	-0.26367
0.5255	11412.39	24324.89	12786.71	5312.34	5056.03	7284.68	1.206748	0.124627	0.244047	2.74876	1.458781

0.5254	210.43	1806.43	1525.79	584.46	492.9	440.83	0.471102	0.303423	0.446079	2.333469	1.222477
0.525	8490.63	9094.57	7861.45	48324.06	40066.93	48667.85	1.367348	0.005086	0.033561	0.185662	-2.42925
0.5246	15714.86	13546.31	13710.01	12614.55	8561.19	7068.04	1.077195	0.081205	0.183216	1.521439	0.605436
0.5242	1267.04	1087.88	787.96	1084.16	201.6	718.54	0.71818	0.281599	0.425473	1.568069	0.648989
0.5238	1983.21	663.33	95.35	412.32	2222.29	1273.37	0.473494	0.63843	0.73486	0.701613	-0.51125
0.5227	28682.4	6655	4375.2	66208.73	63121.86	81284.48	1.191326	0.005339	0.034376	0.188555	-2.40694
0.5218	74337.73	48507.72	38896.7	69589.45	67283.15	63032.23	0.745086	0.352004	0.493054	0.809096	-0.30562
0.5216	18421.46	25837.41	31528.05	21988.91	25066.02	5445.96	0.637211	0.351148	0.49233	1.443536	0.529607
0.5207	630.02	4520.31	822.61	1914.64	300.7	1401.87	0.283818	0.608695	0.711723	1.651256	0.723564
0.5198	155754	170581.3	152470.3	26516.14	48172.57	67545.71	1.260953	0.004092	0.029926	3.366313	1.751169
0.5188	11343.36	9986.18	13121.86	2130.13	1829.29	1114.86	1.342108	0.004643	0.032268	6.789416	2.763288
0.5182	906.46	1602.02	1145.06	426.04	971.26	928.71	0.841226	0.176798	0.310625	1.570733	0.651438
0.5174	67612.63	40969.75	66913.76	18976.08	18589	25841.24	1.278283	0.042964	0.119438	2.767802	1.468741
0.5167	3368.07	4239.29	3228.96	3097.85	334.5	3219.55	0.690073	0.273967	0.418597	1.629056	0.704037
0.5167	3173.92	993.62	1435.32	1180.52	2440.56	1297.41	0.10504	0.786758	0.850143	1.139142	0.187948
0.5165	3741.99	3419.8	3009.29	13007.19	8816.41	7188.98	1.290541	0.066366	0.15974	0.350575	-1.51221
0.515	1533.27	769.87	85.71	643.55	333.61	741.56	0.115929	0.652537	0.746028	1.389901	0.474982
0.5141	11936.86	16901.46	8212.48	1411.64	2044.62	1709.07	1.3342	0.051067	0.133295	7.172978	2.842572
0.5138	6127.34	3336.04	4289.27	1617.8	3208.39	853.52	1.047994	0.067952	0.162094	2.421365	1.27582
0.513	3662.89	2059.53	1424.61	2227.15	5386.88	4971.09	0.807843	0.213502	0.352857	0.567895	-0.8163
0.5126	581.5	795.6	468.9	706.79	863.62	1026.66	0.942728	0.133168	0.255268	0.710801	-0.49248
0.5126	4036.87	16559.16	8008.38	19188.93	20005.73	14978	0.94401	0.133906	0.25612	0.528023	-0.92133
0.5123	4182.34	4850.54	5888.77	16206.28	18901.71	13752.29	1.338418	0.010572	0.049393	0.305394	-1.71126
0.5122	1500.03	2860.3	1199.13	331.54	271.16	94.26	1.257262	0.083952	0.187569	7.976728	2.995797
0.5122	1396.26	364.47	207.97	480.91	445.6	258.17	0.243199	0.557817	0.672063	1.661799	0.732746
0.5112	5056.36	3048.53	4306.49	7367.98	7460.38	6609.1	1.205959	0.021434	0.075713	0.578958	-0.78847

0.5108	2973.9	1841.41	1213.58	10059.16	1932.76	6613.75	0.909346	0.213107	0.352403	0.324035	-1.62578
0.5098	455.44	4944.78	370	3859.18	5313.25	648.42	0.548773	0.545457	0.660457	0.587548	-0.76722
0.5087	3196.83	733.59	760.62	2486.44	2324	1799.75	0.702084	0.519006	0.635491	0.709668	-0.49478
0.5074	12673.44	9064.87	11430.24	10011.46	10253.99	7503.62	0.732566	0.263118	0.408452	1.194442	0.256337
0.5074	27195.55	54930.92	27227.93	7516.8	14887.94	12872.77	1.196192	0.109082	0.224712	3.099833	1.632191
0.5064	3236.39	2129.91	1563.59	8878.86	4929.98	4378.14	1.160086	0.105511	0.219116	0.381036	-1.392
0.5038	2087.21	28331.9	1685.34	4713.74	790.37	244.7	0.728959	0.424504	0.553083	5.584538	2.481438
0.5035	8949.19	4576.76	4219.65	6894.09	11710.1	13944.63	0.962262	0.134545	0.256991	0.545199	-0.87514
0.7787	118795.5	131102.3	118794.6	232252.2	315230	237989.6	1.323414	0.032618	0.099575	0.46939	-1.09114
0.7414	21459.39	17241.26	20795.47	23534.29	23281	32122.93	1.01507	0.142115	0.266749	0.753705	-0.40793
0.7359	37980.21	35510.95	39608.85	13010.46	12387.98	12706.38	1.370212	0.001866	0.019981	2.968129	1.569554
0.9936	2973878	2693933	3289231	4239846	4650739	3980032	1.273172	0.00773	0.040843	0.69593	-0.52299
0.9816	260326.3	258762	267430.4	39057.26	72619.37	60185.29	1.335728	0.001238	0.016047	4.576457	2.194231
0.9816	8401892	7614111	7807391	5922202	6034969	6306716	1.327282	0.006714	0.038427	1.304399	0.383385
0.9756	192721.1	298501.6	205588.9	22495.68	21112.21	19414.18	1.363238	0.02392	0.080703	11.05663	3.46684
0.9697	60636.72	316870.6	179813.8	9365.75	10907.32	100293.9	1.002673	0.17829	0.31199	4.622502	2.208674
0.9548	18959.76	17152.95	24949.36	34584.73	18578.78	28081.33	0.723792	0.288126	0.43115	0.751581	-0.412
0.9462	37400.62	28249.25	30976.79	10141.11	13944.35	12113.83	1.331088	0.009377	0.045823	2.669297	1.41646
0.9393	440589.9	450260.6	374709.9	459901.2	383499.4	463353.5	0.252237	0.7169	0.798986	0.968476	-0.04621
0.9358	1348.67	1948.13	277.67	1610.78	936.01	3563.43	0.582229	0.422821	0.551654	0.584999	-0.77349
0.9344	58155.56	54372.31	33649.84	1485.48	1279.66	1252.97	1.365866	0.024867	0.082674	36.37972	5.185062
0.9319	32564.53	39013.71	40958.44	35978.09	35523.41	33635.11	0.551956	0.43634	0.563892	1.070385	0.09813
0.9273	41402.09	34410.7	39977.59	32644.48	26242.29	17950.87	1.051098	0.073467	0.171071	1.506949	0.59163
0.9191	102271.8	86826.88	87992.13	96371.28	68791.42	84020.94	0.62189	0.38818	0.524675	1.111994	0.15315
0.9152	4563.13	6037.37	6058.29	11557.78	13531.91	13015.55	1.33106	0.000868	0.01464	0.437178	-1.19371
0.91	33628.91	38992.46	23417.87	68622.28	81658.5	36513.27	1.015554	0.142156	0.266749	0.514145	-0.95975
0.8886	12215.61	19848.03	10257.23	2388.72	1724.56	1045.95	1.31756	0.049289	0.130493	8.202943	3.036142

0.8838	830353.4	948555.7	987441.4	6219953	5922111	5971982	1.37054	1.91E-05	0.003728	0.152719	-2.71105
0.8762	182.19	42.79	24.19	42.97	96.87	49.07	0.029668	0.733494	0.812322	1.318988	0.399431
0.8701	434775.5	538137.6	486032	487839.6	462298.3	610415.1	0.398599	0.573659	0.683218	0.93489	-0.09713
0.8638	1781.78	2671.86	735.93	1153.05	1264.7	845.21	0.547339	0.369961	0.507798	1.590449	0.669434
0.8583	18999.74	17358.63	14283.44	22837.17	27069.54	24859.64	1.231551	0.012482	0.054164	0.677334	-0.56206
0.8345	15287.63	19634.21	8728.71	14055.82	131.03	12090.97	0.625729	0.347858	0.488603	1.661118	0.732154
0.8161	3130.48	4819.81	1769.94	8699.16	11739.59	1971.08	0.652327	0.277632	0.42144	0.433748	-1.20507
0.7923	19487.2	25049.2	11702.45	10694.96	14490.84	9319.72	0.908771	0.193601	0.329781	1.629851	0.70474
0.7914	119019	70535.29	85959.25	43259.26	39769.52	38264.1	1.277725	0.067941	0.162094	2.271473	1.183628
0.7864	43078.36	30441.17	29631.75	22341.85	9849.91	13215.63	1.166777	0.029409	0.092558	2.271685	1.183763
0.7856	30404.6	25281.65	25189.69	33620.64	20582.9	34720.78	0.268862	0.625097	0.725259	0.909492	-0.13687
0.7811	1724.03	2000.71	1416.32	2107.49	1102.94	2690.99	0.197253	0.649121	0.742851	0.871156	-0.199
0.7791	17.88	782.12	565.84	2718.28	57.34	75.14	0.049901	0.636339	0.73269	0.479114	-1.06156
0.7704	6256.1	5602.47	1256.87	15334.78	15477.9	17554.14	1.133625	0.008027	0.041903	0.271166	-1.88275
0.7697	115753	179203	96158.42	157587.6	185730.3	160250	0.819572	0.270418	0.415793	0.776687	-0.3646
0.7582	8027.69	9253.04	83.51	14752.75	16238.02	4292.09	0.644892	0.279774	0.423744	0.492144	-1.02285
0.7502	27654.94	22769.49	25019.06	45967.38	42790.96	44866.56	1.344044	0.000708	0.013717	0.564592	-0.82472
0.7485	93780.74	103285.6	77526.02	349.39	114.44	298.58	1.363209	0.006717	0.038427	360.1637	8.492509
0.7449	11360.67	1513.07	10252.94	42586.36	44222.89	42970.11	1.155324	0.00651	0.037834	0.1782	-2.48843
0.7444	4241.46	5426.39	5445.4	3444.65	5513.45	4645.19	0.470224	0.528389	0.644085	1.111	0.151858
0.7403	11957.48	14360.52	11169.78	5369.02	7460.48	3194.7	1.191964	0.011639	0.052144	2.339448	1.226168
0.7399	255.54	102.55	137.33	252.36	411.56	458.66	1.095498	0.059568	0.148227	0.441323	-1.18009
0.7392	115464.4	111304.9	121864.1	35410.04	23063.74	21234.2	1.342856	0.000172	0.007792	4.373884	2.128915
0.7321	2107.2	393.55	1501.1	739.16	555.36	1108.43	0.400283	0.402534	0.536649	1.66539	0.73586
0.7316	49191.4	47197.11	44525.34	108770.2	122811.4	108621.3	1.364345	0.002876	0.025269	0.414205	-1.27158
0.7315	6110.58	4449.81	13410.89	8896.98	5122.97	11904.15	0.217575	0.857605	0.899928	0.924672	-0.11299

0.731	802.42	1304.86	520.88	2241.85	462.8	1099.94	0.311497	0.544152	0.65906	0.690787	-0.53369
0.7278	350.27	1688.45	816.44	7188.28	5213.51	6285.56	1.251825	0.002555	0.023542	0.152786	-2.71042
0.7262	4221.05	3825.18	4280.41	4069.31	2014.34	1606.2	0.961803	0.176011	0.309993	1.602975	0.680752
0.7256	1677	1792.99	1268.29	276.82	1269.4	1078.41	0.842168	0.131814	0.253676	1.805314	0.852249
0.7256	6646.36	2295.33	3720	2179.74	2533.06	3057.37	0.728041	0.330187	0.4717	1.629525	0.704452
0.7256	67704.95	75570.98	57883.54	92290.55	88510.15	81541.9	1.160439	0.036457	0.107665	0.766782	-0.38311
0.7238	35.49	993.73	471.16	1089.75	4987.48	1200.46	0.91022	0.269358	0.415328	0.206162	-2.27815
0.7212	8848.22	8537.43	7086.51	1832.91	1769.62	1987.25	1.365337	0.00674	0.038427	4.378018	2.130278
0.7197	14871.48	13569.89	15608.89	36218.64	34692.31	37277.14	1.365737	3.55E-05	0.00416	0.407164	-1.29632
0.717	2004.11	2383.3	5035.21	1713.4	5303.9	1548.05	0.249276	0.863439	0.903657	1.100086	0.137616
0.7147	555.23	2763.78	673.96	243.84	258.36	517.02	0.983891	0.300392	0.443116	3.917672	1.969997
0.714	8527.3	7699.3	8733.87	3194.9	334.78	753.71	1.17595	0.009667	0.046822	5.82727	2.54282
0.7098	113.88	161.22	302.45	135.57	43.27	165.06	0.685739	0.322534	0.464615	1.679413	0.747957
0.7081	3721.9	2830.38	2537.93	3416.82	2576.02	1451.58	0.558892	0.4679	0.592039	1.221077	0.288154
0.7068	911.73	479.4	231.33	3526.55	7134.62	4326.18	1.281885	0.051242	0.13351	0.108255	-3.20749
0.7024	15045.63	16036.69	16075.83	17166.22	19314.53	19130.42	1.212453	0.036593	0.107996	0.847998	-0.23787
0.6995	6372.61	6337.57	4913.95	1117.9	1221.57	616.04	1.330688	0.004419	0.031524	5.963143	2.576073
0.698	4123.59	2474.02	2905.75	4094.95	2929.13	3986.37	0.543018	0.465282	0.590613	0.863122	-0.21236
0.6974	1088.72	1169.84	884.72	1463.65	573.36	1141.41	0.126822	0.969143	0.977156	0.988944	-0.01604
0.6968	179.76	124.66	126.31	22.05	435.72	256.01	0.033229	0.514419	0.631292	0.603449	-0.7287
0.6968	2799.14	3612.35	2191.83	636.71	177.03	1120.78	1.148028	0.014875	0.059927	4.447263	2.152918
0.6912	19744.44	33060.57	19488.21	50172.04	60390.79	58671.22	1.267419	0.005767	0.035765	0.427179	-1.22709
0.6912	2038.76	88.21	1791.9	1387.61	626.36	2140.84	0.356194	0.922359	0.944903	0.943213	-0.08434
0.6896	47658.9	28942.49	38003.48	76047.51	73123.32	83023.51	1.270156	0.007174	0.039537	0.493573	-1.01866
0.6887	693.71	1474.88	802.14	736.24	989.22	370.91	0.613363	0.395309	0.531181	1.417083	0.502924
0.675	1099.04	500.52	844.1	652.48	592.19	864.03	0.286288	0.603535	0.708349	1.158847	0.21269
0.675	66689	60467.14	57536.62	42083.25	48763.41	49052.65	1.238664	0.014194	0.058571	1.320183	0.400738

0.6701	29755.63	29508.49	18645.41	7300.33	10512.36	7438.2	1.296144	0.032814	0.099869	3.085417	1.625466
0.67	1194.15	1616.6	1515.02	260.1	1019.61	183.2	1.083367	0.051551	0.133994	2.956962	1.564116
0.6698	1582.61	913.76	2106.76	42780.01	41712.45	46867.06	1.358557	0.000878	0.01464	0.035042	-4.83476
0.6698	447454.2	332083	383795	96042.03	145766.4	94089.64	1.326888	0.005373	0.034495	3.463349	1.792168
0.6652	2792.75	6909.07	349	1120.96	230.12	1592.39	0.549429	0.340878	0.481754	3.414616	1.771723
0.6645	112.29	202.25	36.78	54.74	81.85	21.87	0.673042	0.310738	0.453793	2.217089	1.148667
0.6645	22.19	182.48	146.2	43.09	101.49	90.48	0.128023	0.518595	0.635166	1.492683	0.577908
0.6569	4275.8	3186.43	2773.97	7216.75	3700.41	6760.7	0.994976	0.141001	0.265554	0.579041	-0.78826
0.6558	5213.33	3507.01	3981.17	2477.05	4150.33	3090.18	0.807104	0.231392	0.372741	1.307068	0.386334
0.6553	2229.88	35.43	153.63	77.38	1284.05	197.3	0.075813	0.746079	0.821007	1.551866	0.634004
0.6503	3443.71	3640.62	1168.11	408.07	184.18	537.92	1.227999	0.093491	0.202749	7.301946	2.868281
0.6493	73.2	2535.18	257.11	1302.58	932.07	2571.26	0.744347	0.533641	0.649035	0.596243	-0.74603
0.6443	10137.78	9544.66	11564.83	9232.5	10029.44	14787.86	0.290167	0.652582	0.746028	0.917693	-0.12392
0.6409	22895.6	19349.13	17981.93	5654.26	1277.73	4013.79	1.23479	0.001161	0.015971	5.502272	2.460027
0.6398	2847.21	605.94	996.99	1998.78	4633.54	832.74	0.478296	0.496758	0.615511	0.596129	-0.7463
0.6371	12619.76	13563.38	15781.75	14564.57	14046.39	17588.55	0.611694	0.386371	0.522878	0.908341	-0.13869
0.6298	2669.76	250.46	79.52	287.18	2526.1	653.23	0.396816	0.893273	0.924668	0.865349	-0.20865
0.6295	10363.8	7264.1	5914.68	9765.28	7669.02	7528.14	0.287704	0.772765	0.841069	0.94312	-0.08449
0.6254	466.81	1053.15	12062.26	9306.93	8581.64	7393.11	0.845044	0.410035	0.541948	0.537236	-0.89637
0.6251	8161.9	11908.58	12775.84	90179.34	85061.33	91996.07	1.360685	1.97E-05	0.003728	0.122911	-3.02431
0.6214	21122.88	33420.06	24747.12	29643.81	44071.09	30310.29	0.811538	0.242292	0.385753	0.76222	-0.39172
0.621	6695.23	5419.65	17251.92	4819.93	6178.89	6944.72	0.611197	0.417211	0.548303	1.636622	0.710722
0.6148	446.95	2682.53	960.18	1403.7	2263.37	1114.99	0.435515	0.781085	0.845901	0.855209	-0.22565
0.6096	420848.4	394428	387095.5	8220.78	7913.86	8518.06	1.372721	0.000679	0.013514	48.77242	5.607994
0.6022	771.73	1331.17	654.17	3541.04	2643.08	4994.91	1.275106	0.044813	0.122632	0.246629	-2.01959
0.598	414.16	243.69	339.52	385.5	195.13	283.79	0.413022	0.581313	0.689321	1.153803	0.206396

0.5879	284956.5	263463.4	272018.6	214765.1	216081.7	284947.4	0.826108	0.268269	0.414675	1.146193	0.19685
0.5835	77.52	134.53	133.43	78.78	247	55.37	0.120179	0.86564	0.905091	0.906415	-0.14176
0.5787	31147.4	31628.41	37984.26	27939.73	25123.59	25305.67	1.187201	0.061044	0.150588	1.285714	0.362569
0.5731	1055.18	1308.86	803.62	1385.23	831.79	103.17	0.576019	0.536917	0.651944	1.365259	0.449175
0.5705	3284.96	5741.68	3311.95	3485.44	18551.93	5205.78	0.636928	0.406696	0.539991	0.452906	-1.14272
0.5603	91.52	139.29	595.81	21	92.82	339.45	0.524304	0.550502	0.665457	1.823681	0.866854
0.5571	13634.01	24097.23	16706.97	311.11	39.24	1048.37	1.251833	0.028517	0.090801	38.92002	5.282441
0.5406	8765.69	7868.84	11162.47	11535.11	9235.81	7857.14	0.123514	0.858351	0.900266	0.97097	-0.0425
0.5383	294.64	105.33	2748.72	739.92	2266.05	1984.3	0.701755	0.571013	0.680997	0.630966	-0.66437
0.5371	2604.64	5163.93	951.59	7518.57	5514.78	4231.63	0.884535	0.145048	0.27043	0.505078	-0.98542
0.537	4766.26	3189.23	3037.43	1651.61	264.33	738.12	1.144282	0.018312	0.068093	4.141926	2.050302
0.5186	3096.85	2737.84	4571.54	1529.02	2272.6	1715.96	1.165874	0.085621	0.190226	1.886013	0.91534
0.5131	4196.86	5842.4	3677.08	6020.59	7615.37	7207.74	1.117245	0.047361	0.12728	0.658057	-0.60372
0.5114	30560.41	27944.25	20972.9	10660.34	19594.62	12411.42	1.14065	0.036292	0.107252	1.862768	0.897448
0.5081	541.94	60.81	97.11	1055.4	944.66	86.34	0.634267	0.271838	0.416939	0.335439	-1.57588
0.5028	1998.78	1798.49	1256.64	561.22	1678.42	644.42	0.917206	0.176083	0.309995	1.75236	0.809299
0.9159	46593.76	87843.54	86287.35	65359.8	58042.59	69530.83	0.304028	0.567275	0.678223	1.144047	0.194146
0.987	472059.1	444360.9	462926.3	536905.6	494475.7	657778.3	1.028118	0.166014	0.296601	0.816587	-0.29232
0.9639	83283.22	65756.32	80418.61	32978.6	30852.22	31533.12	1.352557	0.01352	0.05683	2.406131	1.266715
0.9145	9405.67	971.55	8352.01	14567.26	14431.05	17847.13	0.904652	0.055273	0.140488	0.399809	-1.32262
0.7402	2159.08	475.05	2554.17	323.29	545.55	502.78	0.975388	0.182789	0.318075	3.782607	1.919381
0.7297	171400.8	144881.6	187097.5	75368.22	101063.8	104165.6	1.259593	0.010263	0.048573	1.793956	0.843145
0.7237	4431.14	5866.2	2983.47	1162.01	426.7	3836.26	0.934555	0.123362	0.24288	2.448089	1.291656
0.72	35619.76	33526.87	33891.02	10103.73	7676.97	12738.02	1.335418	0.000991	0.014993	3.376211	1.755405
0.6918	26470.67	41839.08	41553.6	40777.5	42566.26	49626.34	0.740954	0.271498	0.416565	0.826226	-0.27539
0.6543	11589.41	9814.4	12885.6	11283.07	5578.24	4970.23	0.951833	0.163503	0.294022	1.570636	0.651349

0.6507	1242.34	155.88	154.82	153.62	153.62	153.62	0.620745	0.420816	0.550853	3.369874	1.752695
0.5418	13851.15	15154.26	17564.17	14070.11	12249.37	14127.59	0.876163	0.196364	0.333315	1.151371	0.203353
0.5078	3438.5	7033.1	5531.05	5354.72	4939.95	5451.72	0.080014	0.942507	0.959007	1.016274	0.02329
0.954	1698894	1430201	1561327	1123958	35636.23	854187.1	0.793615	0.105248	0.218886	2.329162	1.219811
0.9224	273172.5	202897.8	259977.2	167055.4	1924.66	177426.9	0.718973	0.137535	0.261569	2.124806	1.087331
0.9076	3786.88	5908.03	4429.91	351.11	4454.32	447.77	0.973811	0.147192	0.27291	2.688803	1.426964
0.9055	9552.62	2883.86	3895.28	5912.86	12043.45	8624.11	0.789808	0.280481	0.424373	0.614428	-0.70268
0.8918	6480.72	32533.89	33918.28	8010.18	11510.54	4981.83	0.836767	0.208366	0.347249	2.976543	1.573638
0.8778	401804	322592.3	370392.4	2237794	2544851	1902139	1.363598	0.008964	0.04476	0.163773	-2.61023
0.8726	76151.33	58623.54	64413.99	62299.45	47648.05	50117.97	0.96016	0.131369	0.253182	1.244421	0.315475
0.8636	64855.18	68881.79	74976.23	87777.97	96060.99	82877.79	1.232086	0.018485	0.068551	0.782528	-0.35379
0.8444	185211.7	104377.8	293595.9	235644.6	242685.4	236162.9	0.596015	0.508412	0.62639	0.816223	-0.29297
0.8364	108.8	51.96	105.6	126.22	144	121.11	0.906937	0.140421	0.264746	0.680653	-0.55501
0.832	9141.88	14624.83	3659.25	4874.18	4556.61	11070.47	0.307223	0.581749	0.689651	1.337769	0.41983
0.8208	1996.79	340.08	1318.75	192064.1	236447.6	243083.7	1.346773	0.005102	0.033561	0.005443	-7.52133
0.8155	22375.81	19462.2	22759.51	6738	20606.91	13373.41	0.887104	0.179	0.313106	1.586449	0.665801
0.8093	2414.61	54.39	2984.56	760.11	71.76	45.35	0.652128	0.226614	0.366943	6.216867	2.636188
0.8064	1544.61	806.58	1417.06	600.91	2497.07	303.18	0.448714	0.878794	0.914458	1.107931	0.147868
0.8053	48935.84	38926.62	58475.11	80875.87	63628.29	49116.41	0.811131	0.231188	0.372688	0.755796	-0.40393
0.7981	348672.3	416234.5	241797.1	92746.69	228857.5	306757.3	0.795381	0.195475	0.332	1.602109	0.679973
0.7885	62739.86	107582.6	93219.44	52322.6	73047.49	52945.88	0.956555	0.152344	0.279607	1.477949	0.563597
0.7799	62764.75	83062.43	53942.86	55119.43	46602.26	37783.82	0.999336	0.131272	0.253182	1.431987	0.518018
0.7793	22946.72	20432.04	20662.11	9205.22	6172.05	4817.73	1.30669	0.001535	0.017791	3.171125	1.664995
0.7789	132047.2	136907.4	103398.3	220706.7	190451.9	217642.6	1.284853	0.003926	0.0292	0.592163	-0.75593
0.7774	158086.6	159757.4	146475	20716.91	20795.9	22293.57	1.37196	0.000838	0.0145	7.276999	2.863344
0.7766	9043.84	7350.28	16765.8	30308.02	29331.12	26195.69	1.22898	0.014892	0.05994	0.386322	-1.37212
0.7765	15156.33	10147.12	12527.87	30086.22	27814.44	26075.77	1.313541	0.001404	0.017053	0.450499	-1.1504

0.776	9053.15	8025.39	6669.45	5363.19	616.01	239.97	1.039626	0.054956	0.140007	3.818514	1.933011
0.7751	44442.15	68908.1	56270.03	41477.92	43384.17	23892.21	0.974739	0.098248	0.209215	1.559665	0.641236
0.7737	5319.88	4204.51	10207.06	5180.35	5417.05	5786.36	0.286607	0.607017	0.710714	1.20433	0.26823
0.7716	18570.73	19315.54	20868.23	4404.74	2249.1	6037.82	1.290071	0.000763	0.014145	4.629379	2.210819
0.7713	168761.1	162717.3	151736.9	189788	183207.1	190746.4	1.25969	0.01851	0.068551	0.857158	-0.22237
0.771	352.09	85.4	149.63	196.92	53.28	39.23	0.723285	0.364783	0.50367	2.028539	1.020441
0.77	125603.7	116684.4	124561.3	386046.4	6848.78	349761.7	0.1172	0.408696	0.540669	0.493969	-1.01751
0.7686	65342.89	91145.96	59547.81	21965.19	17625.37	16037.46	1.336214	0.028139	0.089858	3.883594	1.957392
0.7664	123453.6	132523.3	122525.7	174606.5	151986.1	210173.7	1.193196	0.084672	0.188695	0.705154	-0.50399
0.7635	52955.58	46586.84	41268.16	57911.75	61134.34	49826.86	0.956358	0.121246	0.239797	0.833826	-0.26218
0.7633	24689.15	32298.72	26395.66	49344.88	50718.49	38278.3	1.238632	0.024179	0.081131	0.602736	-0.7304
0.7627	4158.61	2488.95	4292.32	14462.14	31092.34	20994.21	1.309025	0.059919	0.14857	0.164389	-2.60481
0.7601	182.61	47.08	289.12	82.58	183.92	17.01	0.53845	0.415391	0.54677	1.829953	0.871807
0.76	80135.19	87725.21	75656.65	135350.9	128832.7	121719.6	1.338219	0.000898	0.014717	0.631032	-0.66422
0.7593	21544.06	18234.52	41633.73	4152.19	7113.34	5552.77	1.281015	0.096593	0.207005	4.840698	2.275215
0.7589	1082500	1096028	1302868	994260.7	944850.1	947519.5	1.13927	0.102205	0.214395	1.206041	0.270279
0.7572	52.75	1450.92	59.86	711.42	2648.69	1823.91	0.94981	0.175552	0.309651	0.301606	-1.72926
0.7556	4486.36	4923.57	3750.02	372.61	168.47	34.12	1.274318	0.003986	0.029413	22.87891	4.515947
0.7547	2748.39	8967.05	1816.71	1251.45	697.5	462.38	1.118477	0.239512	0.382011	5.611903	2.48849
0.7546	6218.73	3867.95	4608.44	3214.06	15157.02	30811.94	0.738991	0.286447	0.429679	0.298784	-1.74282
0.7512	235735.7	534797.4	279951.1	18230.63	20700.49	17892.97	1.351767	0.070846	0.166478	18.4866	4.208408
0.7475	48371.71	51362.68	45604.02	61750.95	58256.73	65089.25	1.280294	0.007342	0.039748	0.785202	-0.34886
0.7467	544765.9	878283.4	355908.7	394112.9	615522.3	451610.8	0.333576	0.574031	0.683289	1.217425	0.283833
0.7443	4882.11	2397.45	1366.24	12517.46	414.45	9686.12	0.223653	0.330078	0.4717	0.382253	-1.3874
0.744	304784.8	284410.6	313962	618097.5	598717.8	610353.1	1.367679	3.04E-05	0.003892	0.494293	-1.01656
0.7407	66049.55	68200.23	71337.01	32692.63	37370.91	46805.78	1.286429	0.011326	0.051196	1.759117	0.814851

0.7393	8952.17	1824.11	8452.32	10707.54	20891.96	16972.98	0.966827	0.063344	0.154777	0.395874	-1.33689
0.7385	10406.9	6696.52	200.3	328.19	255.22	9330.49	0.370614	0.592405	0.698484	1.7454	0.803558
0.7377	8644.36	10220.96	15987.01	2568.86	2461.79	3785.2	1.301366	0.055642	0.141056	3.953371	1.983084
0.7368	620250.7	1015646	288827	400449.6	2473718	100183.7	0.138033	0.69056	0.778219	0.647107	-0.62792
0.7348	54992.98	38172.08	46063.21	18447.56	18691.03	28176.64	1.248913	0.018041	0.067314	2.131636	1.091961
0.7343	149475.5	172010.9	189483.6	63778.93	20615.34	53448.75	1.225473	0.002141	0.021159	3.706898	1.890212
0.7311	57192.75	58929.85	38383.84	91704.67	141195.3	88787.57	1.198917	0.066866	0.160323	0.4803	-1.05799
0.731	921.8	1171.3	1180.92	2067.45	933.4	97.2	0.4518	0.927977	0.948429	1.0568	0.079703
0.7259	147127.3	111820.3	137878.6	190608.5	197530.8	185354.5	1.250701	0.021821	0.076035	0.691945	-0.53127
0.7222	1332.06	7185.82	5761.15	7449.1	4995.92	4323.51	0.420041	0.705777	0.790057	0.851537	-0.23186
0.7215	22185.45	33201.35	34911.78	44788.5	51654.61	49725.23	1.17705	0.025605	0.083793	0.617771	-0.69486
0.7189	44366.68	36748.1	43438.89	70319.38	74368.47	68735.9	1.336072	0.000929	0.014938	0.583598	-0.77695
0.7165	272.02	246.18	48.18	51.76	384.63	46.49	0.312345	0.845454	0.890607	1.172921	0.230106
0.7151	11715.11	12017.04	11637.41	10354.22	9175.35	7799.02	1.164793	0.064919	0.157511	1.294233	0.372097
0.7146	53002.49	50967.48	51234.72	117761.3	97547.86	118172.1	1.353585	0.012302	0.053896	0.465408	-1.10343
0.7141	2714916	2578147	2207019	2327586	2107898	2523577	0.566838	0.406659	0.539991	1.077743	0.108014
0.7124	9327.09	39751.42	8154.17	8806.07	12183.18	11799.82	0.369965	0.51383	0.630748	1.74548	0.803624
0.7119	1294.79	1063.19	1040.22	5509.02	9380.78	2877.89	1.251699	0.126341	0.246607	0.191257	-2.38641
0.7113	31.73	145.1	73.61	268.47	291.87	163.14	1.078726	0.03939	0.112856	0.34616	-1.53049
0.7103	2028.97	6979.98	4946.7	5413.2	5002.07	8396.39	0.60189	0.421165	0.551143	0.741862	-0.43078
0.7092	176237.7	150140.6	157559.8	138164.4	133281.7	137197.8	1.185336	0.078894	0.17977	1.184254	0.243978
0.7089	108003.5	114725.2	99397.1	68304.87	58080.86	86642.54	1.187241	0.030258	0.094144	1.512127	0.596579
0.7073	7843.46	6053.26	8755.33	12907.73	15101.77	8757.85	1.055849	0.111999	0.228523	0.616091	-0.69878
0.7016	101752.5	127560.5	35248.08	223944.8	206025.1	210192.8	1.083148	0.040575	0.115222	0.413272	-1.27484
0.9986	34227.96	27420.12	29715.16	7777.8	6744.05	7915.6	1.363525	0.006087	0.036575	4.071908	2.025705
0.9832	12174.37	3021.27	9741.78	1493.7	2085.83	3954.93	1.008735	0.160953	0.290515	3.309782	1.726736

0.9774	2678.86	2738.02	4041.54	94943.54	70629.8	91353.31	1.366205	0.008163	0.042258	0.036814	-4.76361
0.9639	341163.6	276313	316609.9	1763024	1739300	1539389	1.36748	0.001521	0.01773	0.185272	-2.43229
0.9515	13657.21	17407.06	22750.53	14968.3	15559.4	15247.26	0.58992	0.416685	0.548078	1.175638	0.233444
0.9513	18425.53	12516.56	86541.41	4830.13	17717.21	10728.67	0.81107	0.358532	0.498992	3.530577	1.819904
0.9438	37649.77	51617.59	36500.3	23070.44	30135.56	32770.02	1.066554	0.093765	0.203141	1.462823	0.548755
0.9412	670.34	1373.45	1180.85	1827.31	772.55	690.6	0.067809	0.961614	0.972095	0.979997	-0.02915
0.9391	14071.02	15884.85	10173.55	35161.21	35419.49	35025.39	1.325753	0.005725	0.035647	0.379992	-1.39596
0.9344	274928.5	259036.9	294478.5	478501.5	204492.3	207111	0.0291	0.842635	0.888712	0.930726	-0.10357
0.9291	5572.36	5743.4	4038.06	698.01	7040.7	606.53	0.845685	0.388071	0.524675	1.83983	0.879572
0.9282	19941.79	17344.39	17191.09	14653.63	11446.88	12278.37	1.22965	0.015114	0.060611	1.41946	0.505342
0.9243	15497.76	22345.88	14099.99	10537.86	4786.25	20633.47	0.687568	0.385329	0.521933	1.444581	0.530651
0.9197	35711.37	39275.45	39410.39	154324.9	141500.4	158196	1.369726	0.001207	0.016008	0.251964	-1.98871
0.9187	8424.45	7382.43	7704.07	3026.85	2855.83	280.85	1.026205	0.01481	0.05985	3.814527	1.931504
0.9083	2926.71	2744.18	2097.73	2901.17	3052.3	1650.18	0.145946	0.920704	0.944718	1.021696	0.030966
0.9055	873045.1	680734.3	832765.1	61.69	971447.1	283.76	0.947577	0.280949	0.424786	2.455817	1.296203
0.8967	2636.01	2328.86	2713.6	668.87	296.39	1617.22	1.099473	0.040311	0.114643	2.973293	1.572062
0.8859	5805.75	7325.66	9499.91	6347.36	5991.55	6388.53	0.699091	0.348419	0.489133	1.208458	0.273167
0.8729	633963.9	600463.5	632275.8	632332.6	564585.5	622962.4	0.432914	0.559419	0.673063	1.025728	0.036649
0.8572	16248.84	11593.06	11335.52	8218.91	8331.58	1646.3	0.913344	0.068317	0.162639	2.152985	1.106338
0.8571	164400.1	149887	141172	76149.29	88881.12	73272.63	1.338559	0.001508	0.017674	1.911261	0.934524
0.856	1069.17	1589.23	575.96	26306.89	30133.43	455.92	0.78726	0.194855	0.331397	0.056847	-4.13678
0.851	75841.23	73662.43	84247.56	190325.6	163722.9	185482	1.357956	0.002585	0.023719	0.433249	-1.20673
0.8252	99675.38	144130.6	108360.4	67186.07	58494.83	68643.61	1.266369	0.054255	0.138789	1.812259	0.857789
0.8204	411718.7	338101.6	398700.8	237732.5	223048.5	221476.7	1.331668	0.016721	0.064216	1.683412	0.751389
0.817	2435.96	7164.89	376.27	47327.21	1428.02	7989.53	0.664989	0.390413	0.527039	0.175825	-2.50779
0.8169	6152.59	9847.41	3541.63	21396.67	23471.28	18898.23	1.238967	0.003913	0.02918	0.306458	-1.70624

0.8149	5132264	3783576	4563556	8963108	6934927	6682949	1.22895	0.032862	0.099869	0.596936	-0.74435
0.811	71256.97	67165.16	67668.89	111665.4	98058.15	102225.9	1.344585	0.007854	0.041298	0.660655	-0.59803
0.8101	2185.05	2191.64	3457.67	5047.74	4881.15	9970.01	1.167398	0.130199	0.252238	0.393708	-1.3448
0.807	6201.94	2957.84	5208.75	8924.64	10298.54	12948.08	1.182365	0.018956	0.069671	0.446626	-1.16286
0.8069	96026.82	98997.18	87581.93	10558.14	12755.14	13043.16	1.369009	0.001084	0.015701	7.773201	2.958509
0.8069	185.63	39870.21	46640.62	429.23	60766.12	443.67	0.309548	0.75467	0.828199	1.406519	0.492129
0.8063	11200.15	4215.41	13775	11795.39	6514.01	6540.87	0.142506	0.692555	0.779661	1.174658	0.23224
0.8021	250.64	49663.8	15375.13	708.91	119.11	41816.58	0.372507	0.726436	0.805939	1.531016	0.614489
0.8006	37520.21	72770.83	27298.69	36187	38859.18	6792.78	0.648992	0.344862	0.485547	1.681225	0.749513
0.7994	890519.1	6466.69	887758.9	417.93	739344.7	1245.21	0.665875	0.417236	0.548303	2.408537	1.268157
0.7914	5395.83	6189.33	20690.05	156.24	4282.26	5827.28	0.785938	0.275179	0.419815	3.143961	1.652583
0.7902	69700.04	55133.87	46230.6	69589.54	64004.73	66926.31	0.796588	0.285571	0.428651	0.853102	-0.22921
0.7901	6034.21	1887.65	7476.53	85062.58	60503.41	87951.15	1.311006	0.01174	0.05238	0.065941	-3.92268
0.7876	15617.9	16320.12	16921.8	8789.12	7482.77	8844.55	1.351874	0.000201	0.008588	1.945332	0.960017
0.7855	112794.2	100330	112168.9	56684.79	56487.76	62619.14	1.354409	0.001788	0.019432	1.850446	0.887873
0.7846	1745.68	752.54	2649.69	2366.48	2794.41	2166.79	0.726582	0.315402	0.457821	0.702529	-0.50937
0.7836	34740.7	64093.3	33158.07	35356.92	32607.67	38134.98	0.507233	0.48226	0.604593	1.24404	0.315032
0.7821	2510.12	5622.22	6746.84	196.45	63.55	24887.68	0.627219	0.719994	0.801853	0.591672	-0.75713
0.7807	5715.12	5074.05	5981.86	2098.07	2645.76	2555.15	1.343714	0.001311	0.016474	2.297722	1.200205
0.7783	51063.51	43940.1	48284.83	9306.92	3758.69	5610.6	1.331328	0.000136	0.006718	7.672244	2.939649
0.7777	10724.75	5746.36	11843.89	518.48	287.81	810.02	1.329179	0.040811	0.115573	17.5183	4.130791
0.7775	4989.79	4939.57	4457.62	1947.18	228.87	1327.14	1.094412	0.011843	0.052522	4.106823	2.038023
0.7762	2598.35	4693.16	2609.4	1737.63	5182.97	2044.84	0.309567	0.824607	0.878412	1.104342	0.143187
0.7743	319.81	146.64	1082.97	2965.08	186.17	2596.73	0.631415	0.244914	0.388211	0.269559	-1.89133
0.7712	15832.31	17002.53	13695.42	19763.62	29889.68	16175.34	0.876021	0.254666	0.400465	0.706839	-0.50055
0.7709	81613.69	73421.83	85140.17	167181	172127.4	182590.6	1.361225	0.000126	0.006553	0.460196	-1.11968

0.7704	21995.31	15902.22	17366.92	6284.59	8576.66	5921.49	1.312639	0.013033	0.055805	2.659151	1.410966
0.7675	6895.27	7899.04	7523.56	23206	29477.01	32054.92	1.356326	0.014686	0.059716	0.263375	-1.92481
0.7672	148756.4	75191.34	123823.2	240470.4	186829	230470.6	1.150569	0.021545	0.075737	0.528712	-0.91945
0.7666	7168.31	4450.05	7283.63	4331.76	4788.48	1883.16	0.908616	0.111395	0.227749	1.717832	0.780589
0.7635	1502.03	144.09	8385.07	11937.74	10991.13	12119.88	0.949373	0.079574	0.18094	0.286207	-1.80487
0.762	8491.85	6740.08	4351.41	2958.51	171.93	2832.49	0.914627	0.043065	0.119642	3.284181	1.715534
0.761	23111.72	9589.62	22422.95	24660.44	27350.31	27536.43	0.828147	0.201327	0.339098	0.692976	-0.52912
0.7604	84769.27	194692.3	187520.8	109443.6	88684.64	74471.7	0.87628	0.203553	0.341922	1.713069	0.776583
0.76	19104.55	69647.77	42121.37	76216.04	67431.76	80657.47	0.90482	0.15961	0.288808	0.583462	-0.77729
0.7597	5348.18	4457.55	4157.5	5856.33	4866.39	4381.99	0.44207	0.53693	0.651944	0.924429	-0.11337
0.7595	115419.7	174381.3	103571.8	109975.7	119635.8	9095.35	0.66585	0.295032	0.43802	1.647933	0.720657
0.7579	5029.55	6046.56	4189.12	3082.78	3152.49	657.63	0.941901	0.055417	0.140606	2.214631	1.147066
0.7578	11845.31	25405.51	22940.14	13937.1	645.63	819.14	1.026452	0.069714	0.164884	3.908029	1.966441
0.7558	1813.2	4793.53	5308.6	1311.07	2954.28	3843.72	0.510083	0.397011	0.531969	1.469383	0.555211
0.7543	319169.7	340918	303934.6	61557.37	76339.71	73073.3	1.366415	0.000443	0.011141	4.569468	2.192026
0.7541	2344.86	1679.38	2772.33	211.61	498.11	1869.84	0.983517	0.092846	0.201854	2.634779	1.397682
0.7535	3598188	3032662	3390983	3581.89	4181378	11778.78	0.937384	0.296922	0.439503	2.388005	1.255806
0.7515	21984.88	23114.54	36597.42	24862.7	25774.79	74451.43	0.507824	0.474303	0.597539	0.65311	-0.6146
0.7506	213497	169858.6	169968.1	200013.9	250493.5	322565.7	0.981226	0.164637	0.295451	0.715746	-0.48248
0.7473	526.36	323.36	425.61	373.66	347.01	168.56	0.778437	0.214021	0.353377	1.434196	0.520242
0.7461	9668.71	9878.07	12009.82	15789.65	4901.58	16701.59	0.066715	0.661377	0.752923	0.843921	-0.24482
0.7459	38749.69	32688.22	34866.13	27574.68	27125.74	28586.82	1.261047	0.042792	0.119112	1.276354	0.352029
0.7443	1502.68	469.48	1559.76	9529.33	7147.48	7221.51	1.275195	0.005458	0.034679	0.147789	-2.75838
0.7427	153783.8	184348.6	148816.1	137411.8	163177.4	142760.8	0.655652	0.351721	0.492862	1.098339	0.135323
0.7427	280000.8	294501.7	303434.2	34239.41	32972.66	35465.89	1.372379	0.000618	0.012889	8.550392	3.095991
0.7415	1086.05	626.06	68.49	234.85	271.99	738.01	0.004978	0.630508	0.729015	1.430373	0.516392

0.7415	22580.22	37094.57	14820.43	16955.49	31377.73	20091.19	0.104577	0.811268	0.867574	1.088723	0.122637
0.7411	337.41	224.7	218.75	214.34	217.41	220.44	0.677312	0.382176	0.519136	1.197289	0.259772
0.7403	30979.06	54828.25	37102.04	139755.9	164146.3	90976.98	1.266558	0.040831	0.115573	0.311258	-1.68382
0.7396	1190692	1148614	1171437	2082767	884875.4	905968.2	0.032223	0.788846	0.851344	0.906323	-0.1419
0.7383	4561.2	5341.15	4711.87	11745.87	3553.1	113.28	0.490871	0.945634	0.961291	0.948221	-0.0767
0.7379	12393.11	11615.43	14068.56	544.91	9196.72	930.83	1.041624	0.07547	0.174412	3.56779	1.835031
0.7379	12698.16	13371.86	14559.04	694.97	708.51	237.69	1.341459	0.000863	0.01464	24.75616	4.629715
0.7377	280.49	229.39	278.08	79.17	180.91	633.69	0.249828	0.855565	0.898436	0.881614	-0.18178
0.7372	44692.81	41737.3	43098.72	78689.58	32680.95	33007.03	0.041251	0.776924	0.843452	0.897153	-0.15657
0.7362	8685.21	9800.65	14129.53	2291.24	6435.54	8843.61	0.891571	0.120272	0.238737	1.85627	0.892407
0.7353	418979.8	521448.9	451787.2	113296.5	117116.1	125211.1	1.365595	0.006956	0.039121	3.914858	1.96896
0.7349	323262.6	386066.8	433350.2	224528.5	264268.7	291477.6	1.176722	0.041838	0.117582	1.464458	0.550367
0.7349	24889.54	22743.73	27118.89	2071.65	3284.82	4469.67	1.340386	0.000532	0.012243	7.60748	2.927419
0.7347	19029.7	57549.76	26618.98	35801.43	32598.51	39989	0.314716	0.89762	0.927626	0.952112	-0.0708
0.7346	221.94	256.19	598.93	308.85	104.67	545.9	0.2578	0.834019	0.883268	1.122616	0.166864
0.7344	145596.5	127179.3	135708.2	223347.5	216261.5	214956.7	1.352961	0.000958	0.014975	0.624053	-0.68026
0.7338	25410.17	7212.4	6750.5	37802.99	20061.98	13143.35	0.754084	0.334357	0.475983	0.554485	-0.85078
0.7323	1686.73	1038.65	603.11	3130.29	753.23	2810.93	0.671832	0.268985	0.415046	0.497201	-1.0081
0.7321	11708.83	16368.26	20791.34	22988.04	40985.41	11699.32	0.536435	0.4075	0.540357	0.645786	-0.63087
0.7318	1100229	826567.6	1166301	758230.9	858905.9	811637.1	0.986717	0.159361	0.288808	1.273522	0.348824
0.7316	25653.63	28750.36	21948.37	38293.91	37721.09	34775.18	1.255158	0.013161	0.056121	0.689162	-0.53709
0.7304	3557.85	2168.61	2490.04	3347.89	4415.03	7326.09	0.998479	0.185278	0.320411	0.544535	-0.8769
0.7286	457.75	685.97	778.54	3492.99	4992.09	3900.65	1.344947	0.013071	0.055915	0.1552	-2.6878
0.7279	21415.75	12418.05	21158.41	7225.1	8637.36	9382.3	1.218736	0.072664	0.169653	2.178361	1.123243
0.7279	29570.88	41851.69	30715.32	7101.83	7612.16	7384.57	1.35825	0.020755	0.074036	4.621925	2.208494
0.7268	9450.23	12589.81	8599.49	18307.48	134053.8	24349.85	1.004647	0.324787	0.466817	0.173388	-2.52793
0.7264	72521.43	84420.45	88471.21	53264.39	90859.22	55123.2	0.745034	0.337577	0.478069	1.231704	0.300656

0.7245	9209.44	12723.17	11792.64	43010.01	1386.64	2202.44	0.469775	0.784657	0.848715	0.723732	-0.46647
0.7238	64814.02	76453.21	61172.84	51705.72	50825.44	44701.31	1.221522	0.039453	0.112856	1.374969	0.459399
0.7235	77975.64	40907.28	55834.81	193897.9	201840.2	279803.3	1.308041	0.015781	0.062196	0.258634	-1.95102
0.7234	9630.47	21170.88	6767.97	1011	7359.91	1029.73	1.043144	0.154187	0.282158	3.996464	1.998724
0.7231	1889.43	1189.82	361.72	121.6	700.78	972.64	0.580866	0.355261	0.4965	1.916954	0.938815
0.7229	671.92	208.25	606.73	831.49	390.31	185.81	0.137213	0.917759	0.942571	1.05633	0.07906
0.7221	2930.4	17001.01	16890.42	2540.17	6528.85	3712.07	0.768476	0.22401	0.364435	2.880962	1.52655
0.7217	34468.48	30947.72	33667.14	43719.76	2980.16	41126.91	0.46307	0.802809	0.862023	1.128167	0.173981
0.7216	7524.41	231.74	6185.62	14029.05	15480.06	14555.32	0.881938	0.042217	0.117812	0.316395	-1.6602
0.7212	997.55	2903.38	164.18	148.52	130.1	4236.89	0.277184	0.930219	0.94983	0.900255	-0.15159
0.7211	48542.29	38994.36	45642.82	9510.17	9815.75	11474.73	1.36317	0.005082	0.033561	4.323918	2.112339
0.7202	1680.73	1495.84	865.63	481.28	791.73	1172.75	0.857325	0.172175	0.305334	1.652738	0.724858
0.7198	261584.1	318996.4	231113.4	219962.2	211843.6	209296.4	1.052702	0.156069	0.284408	1.266091	0.340381
0.7197	5610.08	7905.8	8351	7230.86	6180.48	7456.8	0.175905	0.74697	0.821404	1.04786	0.067445
0.7191	2135.59	3904.55	4547.6	4761.08	551.73	705.16	0.788443	0.399261	0.533872	1.759354	0.815046
0.7177	132616.4	134630.9	143855.5	3541.37	201657.7	17650.99	0.825879	0.429182	0.556621	1.844751	0.883426
0.7175	14039.95	9433.61	28011.47	22301.36	19111	18665.29	0.501718	0.661845	0.753259	0.856975	-0.22268
0.7163	544990	514732.3	454621	107275.6	128213.2	109317.4	1.365246	0.002985	0.025612	4.391869	2.134835
0.7153	1720.37	2062.41	16.77	31.17	10.92	653.17	0.579145	0.238566	0.380979	5.464934	2.450204
0.7147	1334.06	2098.54	3062	4850.76	2752.21	4970	1.017718	0.090007	0.197554	0.516553	-0.95301
0.7144	23963.55	20479.96	18765.8	24106.26	25150.21	28036.73	1.059129	0.075877	0.174916	0.817786	-0.2902
0.7139	19217.98	15467.48	18021.3	20307.35	16740.96	16778.22	0.156393	0.829052	0.880434	0.979197	-0.03033
0.7139	19167.9	12061.35	19120.52	12491.66	11945.77	12961.4	0.909649	0.207471	0.346555	1.346293	0.428992
0.712	117192.6	72283.35	88940.94	153108.4	142291.2	178582.5	1.194743	0.019712	0.071248	0.5874	-0.76759
0.7116	1934.7	2179.99	2765.88	3018.53	925.26	681.41	0.748667	0.421617	0.551394	1.487626	0.573012
0.711	70692.8	56516.5	63151.88	55270.63	59272.85	57925.78	0.786729	0.279972	0.42386	1.10374	0.1424

0.7107	1462.57	50630.62	57061.2	7533.64	10320.31	8577.49	0.340802	0.25676	0.402242	4.129718	2.046043
0.7103	4053	337.29	3207.04	12657.85	2260.22	5491.59	0.737432	0.297927	0.440673	0.372242	-1.42569
0.7101	35096.62	34636.75	43385.08	15647.88	15277.92	15531.45	1.354721	0.015863	0.06235	2.434893	1.283859
0.7098	145001	265982.5	62014.55	370962.9	551881.2	450697.2	1.115667	0.019671	0.071157	0.344364	-1.53799
0.709	2426.83	1549.11	2368.36	6174	5446.45	6512.56	1.318375	0.000788	0.014349	0.349876	-1.51509
0.7085	166079.8	144248.3	139634.5	173825.1	150613.3	172182.9	0.79161	0.233078	0.374626	0.906048	-0.14234
0.7081	1269.98	3508.77	1424.83	1102.41	1836.75	2367.94	0.161826	0.736842	0.814788	1.168921	0.225177
0.7081	9291.8	7385.18	11009.71	5460.42	4393.71	3665.17	1.250083	0.028472	0.090722	2.047938	1.034172
0.708	13151.49	8296.85	9730.28	13084.24	9531.6	17177.74	0.651557	0.346509	0.48755	0.783509	-0.35198
0.7075	100206.7	92313.26	99986.92	90335.61	88304.06	89480.24	1.154878	0.08195	0.18423	1.090956	0.125592
0.7074	54486.59	66880.62	52977.2	20766.52	18582.71	22425.49	1.351891	0.009936	0.047594	2.822261	1.496852
0.707	418.11	237.74	599.01	967.1	542.4	1159.54	1.013856	0.105413	0.219049	0.470154	-1.08879
0.7069	12.37	31.4	268.3	193.46	280.34	102.13	0.779509	0.425221	0.553083	0.541854	-0.88402
0.7067	124173.5	56612.21	38722.42	20720.06	110797.9	125514.7	0.021277	0.780548	0.845901	0.854009	-0.22768
0.7065	1192.01	76.22	257.15	3750.32	2404.62	6937.71	1.152465	0.094901	0.204789	0.116507	-3.10152
0.7063	829.86	10085.62	851.79	179.1	242.25	242.14	1.088737	0.352663	0.493344	17.73541	4.148561
0.7062	8197.7	9865.53	7153.28	7216.62	10544.49	14864.26	0.604559	0.384193	0.521226	0.772911	-0.37163
0.7056	26833.65	22202.54	24465.48	90057.25	74189.45	90742.03	1.360092	0.00552	0.034788	0.288255	-1.79458
0.7054	72991.1	82803.35	79816.14	13622.83	11947.01	12106.9	1.37048	0.001437	0.017207	6.253476	2.644658
0.7052	12216.16	4802.03	8239.74	739.43	571.72	791.46	1.335652	0.068998	0.163725	12.01266	3.586483
0.705	26333.23	18124.64	28245.55	4820.73	11497.59	5631.35	1.237818	0.014374	0.059052	3.312278	1.727824
0.7034	5728.3	1321.63	6093.96	2916.97	1001.34	2425.47	0.635541	0.274966	0.419815	2.071933	1.050978
0.7028	1596.3	1944.99	1250.47	870.61	1669.89	1733.24	0.368241	0.643045	0.738729	1.12121	0.165057
0.7025	18613.57	17880.03	18549.7	6479.56	11161.28	13395.02	1.119796	0.057371	0.144555	1.773539	0.826631
0.7024	15879.64	16977.56	20070.52	18262.78	15567.44	19707.11	0.07975	0.912925	0.938326	0.988613	-0.01652
0.7024	811.18	411.04	73.1	2205.24	33.9	1849.79	0.253832	0.298672	0.441326	0.316787	-1.65841

0.7024	380.75	212.09	683.31	321.48	811.87	1215.8	0.672987	0.30808	0.451403	0.543239	-0.88034
0.7009	71367.91	43565.77	63911.04	15064.46	19264.75	16209.41	1.326472	0.033259	0.100678	3.538773	1.823249
0.7008	16188.3	16296.42	21672.8	13155.18	19171.12	18985.34	0.254197	0.741297	0.81764	1.055463	0.077876
0.7006	19670.3	3337.88	34429.33	24619.11	16243.08	13594.27	0.270516	0.924981	0.946699	1.054742	0.07689
0.6995	3727.63	3388.04	5686.09	3889.15	2512.79	2871.31	0.835087	0.24454	0.388042	1.380504	0.465195
0.6994	14901.71	11422.83	11211.22	15987.7	12768.46	11322.6	0.312328	0.667	0.757741	0.93655	-0.09457
0.6993	75790.68	71615.81	67879.28	222.86	219.32	651.9	1.360725	0.000983	0.014975	196.7733	7.620391
0.6986	12482.98	9962.92	13267.22	494.17	118.14	48	1.312134	0.006385	0.037563	54.08538	5.757167
0.698	1014.97	866.12	958.48	1146.09	1988.93	1080.93	0.916211	0.255724	0.401549	0.67353	-0.57019
0.6977	24162.76	18283.89	26298.07	4505.55	4545.12	5689.41	1.352533	0.015342	0.061075	4.663796	2.221505
0.6977	213620.1	253739.6	213748.9	150153	150715.7	110908.8	1.248751	0.008741	0.04428	1.654069	0.72602
0.6975	18889.66	33993.34	14962.09	1471.39	2699.24	1528.29	1.330118	0.069582	0.164661	11.9049	3.573484
0.6974	5822.76	5854.26	4883.15	17470.53	6842.7	10765.45	1.064906	0.183753	0.319115	0.472086	-1.08288
0.6973	82037.16	57327.9	71741.2	63378.01	50497.95	56515.41	0.884477	0.191222	0.327006	1.238949	0.309117
0.697	114027.5	90111.66	102367.6	95280.25	84012.44	94950.55	0.771893	0.261743	0.407485	1.117646	0.160463
0.6966	419780.6	384582	410776.8	309510.5	292737.5	303679.6	1.343322	0.003814	0.02898	1.341321	0.423654
0.6958	448573.6	392188.9	461131.2	312418.3	218118.5	186271.9	1.218425	0.01864	0.068858	1.816236	0.860952
0.6954	11082.39	12614.93	14532.86	13698.44	15751.67	12153.01	0.500263	0.479528	0.602555	0.918926	-0.12198
0.6951	7652.57	7923.24	4157.98	9272.1	10696.26	7877.04	0.877478	0.147929	0.273897	0.708691	-0.49677
0.695	20473.7	132706.3	67442.19	90223.53	149947.9	91675.91	0.668403	0.395683	0.531326	0.66483	-0.58894
0.6943	28234.72	23672.5	28895.48	13164.31	13433.4	15495.81	1.33201	0.007222	0.039577	1.9196	0.940805
0.6934	59229	54345.07	53243.94	70663.17	94033.96	57813.14	0.934376	0.219489	0.359682	0.749709	-0.4156
0.6926	32611.62	33186.25	28740.48	22096.29	6556.63	6689.9	1.125852	0.05354	0.137774	2.674895	1.419483
0.6923	474.82	3258.99	4772.1	1893.11	2790.67	2751.82	0.209977	0.806112	0.863988	1.143944	0.194016
0.6922	550992.6	478625.2	616305.6	398905.2	253598.4	412140.8	1.092105	0.042738	0.119112	1.545984	0.628526
0.6912	4745.62	5616.73	6094.39	4883.34	7090.24	6342.79	0.486039	0.468756	0.592263	0.898472	-0.15446
0.6912	595	481.77	147.9	306.86	258.44	118.26	0.594066	0.312666	0.455367	1.791606	0.841253

0.691	58488.58	57157.74	52824.35	22823.5	18096.89	34018.2	1.254977	0.013844	0.057749	2.248116	1.168717
0.691	40278.17	36409.58	38914.79	61919.19	57330.58	57552.47	1.350505	0.000585	0.012688	0.653852	-0.61296
0.6908	15186.34	13799.04	14039.51	16701.2	14917.37	10020.36	0.2357	0.840491	0.887873	1.033285	0.047239
0.6906	2991.85	1417.25	3306.28	2177.61	2227.7	1739.17	0.399599	0.467891	0.592039	1.25566	0.328446
0.6905	21872.98	22849.79	22958.83	29265.78	26946.57	23781.14	1.091909	0.117009	0.234503	0.846089	-0.24112
0.6889	10150.3	4850.53	12192.11	5719.64	5876.99	3473.33	0.876429	0.198278	0.335518	1.804447	0.851557
0.6886	2481.89	1434.83	461.17	170.53	2343.22	2051.11	0.158245	0.947994	0.963017	0.959041	-0.06033
0.6877	12820.3	28737.81	38449.92	23876.23	19489.92	9945.96	0.556736	0.370937	0.508144	1.500748	0.585682
0.6869	1302.8	295.02	1599.33	562.14	905.71	478.02	0.361743	0.404942	0.538646	1.643044	0.716371
0.6858	33786.72	20922.21	30389.73	12318.79	12514.65	17053.98	1.220961	0.049335	0.130493	2.031604	1.022619
0.6858	558.84	886.21	722.93	2780.36	1796.67	3435.97	1.291224	0.050098	0.131328	0.270558	-1.88599
0.6848	175779.7	293992.1	208610.8	296840.2	285831.9	375498.9	0.989825	0.111061	0.227284	0.707997	-0.49818
0.6847	6591.88	7905.38	9974.11	3079.5	4772.65	6018.97	1.082462	0.054492	0.139314	1.764196	0.819011
0.683	35123.09	8881.36	3448.02	33298.73	36191.2	26782.87	0.880484	0.233345	0.374842	0.492896	-1.02065
0.6829	9502.64	7938.5	9118.29	8090.62	4936.71	8873.85	0.69047	0.327477	0.469255	1.212694	0.278216
0.6828	789.34	316.03	504.91	3206.92	2047.98	316.1	0.720095	0.254845	0.400602	0.289047	-1.79062
0.6816	49606.16	20576.88	43764.35	64184.18	50605.72	101211.3	0.957647	0.141061	0.265554	0.527531	-0.92267
0.6813	31497.79	27403.75	44997.76	4206.04	4876.59	3162.9	1.350813	0.028063	0.089748	8.484672	3.084859
0.6806	3942.74	11199.46	2214.97	2258.57	2409.53	1375.32	0.886434	0.303584	0.446125	2.872077	1.522095
0.6804	14232.9	15686.39	17739.64	28532.4	28791.72	27366.09	1.338557	0.002397	0.022717	0.562744	-0.82945
0.6802	34078.98	33660.42	24377.06	253969.4	224215.7	235965.1	1.364507	0.000561	0.012597	0.128988	-2.9547
0.6795	2262.81	4320.62	1134.68	3402.17	857.05	2204.87	0.219495	0.743525	0.819269	1.193998	0.2558
0.6791	476660.1	538519.6	501667.7	557274.3	551027.2	544598.6	1.057865	0.122329	0.24161	0.917689	-0.12392
0.6788	5117.67	5054.52	21845.06	23439.72	6593.71	15957.64	0.508273	0.564468	0.675747	0.696162	-0.5225
0.6785	7337.08	4497.57	4769.7	23886.96	26560.67	30894.94	1.343114	0.003321	0.027107	0.204129	-2.29245
0.6785	94852.07	61039.85	78468.88	105319.2	119193	106202.4	1.101077	0.063204	0.154777	0.70865	-0.49686

0.6774	803.53	578.54	131.71	1419.61	1732.13	931.56	0.986504	0.05006	0.131328	0.370725	-1.43158
0.6772	36885.94	42866.51	35080.1	13626.56	16878.56	11802.03	1.335323	0.002007	0.020634	2.714259	1.440558
0.6764	4238.15	2669.82	5417.65	4936.53	5172.68	5854.78	0.783595	0.263535	0.408662	0.772089	-0.37316
0.6763	43328.51	38305.44	39378.16	30879.25	13039.42	26629.15	1.024829	0.079225	0.180242	1.71532	0.778478
0.6762	1582.19	1613.74	3438.78	569.6	2320.53	2659.72	0.363474	0.706044	0.790153	1.195476	0.257585
0.6762	130877.9	129877.5	96684.16	257103.9	246019.4	230336	1.319686	0.001338	0.016618	0.487334	-1.03702
0.6749	1425.78	1048.21	2702.89	16551.65	2693.96	1406.82	0.686956	0.398831	0.533775	0.250667	-1.99616
0.6748	4778.59	7978.2	6534.17	6879.47	1623.54	4666.7	0.681733	0.328537	0.470192	1.464798	0.550701
0.6747	16063.87	15409.03	16947.73	3242.46	2108.89	1153.01	1.31975	8.92E-05	0.005804	7.444334	2.896143
0.6739	5336.95	3245.51	3996.06	101754.9	95383.22	103817.5	1.367272	0.000375	0.010037	0.041795	-4.58052
0.6718	15325.88	35479.77	46644.42	12107.15	19372.01	22364.78	0.777415	0.249263	0.393816	1.809861	0.855879
0.6718	16756.96	19200.45	21043.77	20271.05	21768.54	22163.8	0.89237	0.183415	0.318909	0.887822	-0.17166
0.6711	17964.36	11227.95	29375.1	25256.62	3359.92	25126.27	0.314108	0.867651	0.906542	1.089772	0.124026
0.6709	1387.9	370.49	715.27	11085.16	13202.18	9382.31	1.319707	0.007625	0.04042	0.073469	-3.76673
0.6705	13740.46	5738.54	7120.25	1543.62	505.19	1535.4	1.254837	0.087131	0.192794	7.421231	2.891658
0.6704	30113.81	26852.8	25921.35	20066.35	18685.67	21242.79	1.290163	0.011687	0.052198	1.381586	0.466325
0.6697	5321.48	3698.06	7266.12	1725.61	3862.51	1143.32	1.054956	0.076639	0.176216	2.419343	1.274615
0.6697	10890.71	8152.05	1499.81	1832.81	9555.66	7826.13	0.010284	0.909241	0.935864	1.069113	0.096414
0.6694	125029.3	103956.9	128921.8	85986.24	67676.44	78176.34	1.254622	0.01459	0.059559	1.543778	0.626465
0.6693	510.24	991.09	919.04	2380.88	545.36	4646.92	0.740459	0.283749	0.426798	0.319598	-1.64567
0.6689	34640.97	30577.75	23109.12	11935.31	8021.98	6894.52	1.301227	0.01414	0.058571	3.289456	1.717849
0.6688	64393.41	54312.42	75985.22	23644.86	23302.41	24001.01	1.34786	0.022182	0.076801	2.744126	1.456347
0.6686	12456.95	8643.91	7555.16	171.78	7318.59	449.35	1.001513	0.078547	0.179449	3.609198	1.851678
0.6682	8090.31	4813.93	3552.91	5565.93	5937.64	3271.03	0.182473	0.745251	0.820548	1.113881	0.155595
0.6677	206373.2	372749.6	201382.9	2342.94	1745.79	1689.7	1.36708	0.0444	0.12203	135.0723	7.077588
0.6674	1632.07	890.07	1325.14	437.25	1201.65	1930.49	0.301182	0.860071	0.901416	1.077854	0.108161
0.6655	34833.95	30516.83	34354.3	30535.32	30216.36	33400.85	0.649361	0.342358	0.483283	1.058974	0.082667

0.6653	28243.04	22431.97	39208.93	64695.5	47887.48	55780.68	1.192733	0.019356	0.070605	0.533868	-0.90545
0.6648	15762.11	14422.85	30497.57	20752.31	503.74	30660.1	0.4939	0.793194	0.854767	1.168857	0.225098
0.6643	63528.11	69052.11	31129.22	90987.37	66666.08	70939.47	0.7943	0.209985	0.34896	0.716161	-0.48164
0.6636	2645.32	4618.39	4689.01	150.26	2735.65	1157.43	0.921413	0.059764	0.148355	2.95615	1.56372
0.6634	767.33	319.44	2010.13	281.47	746.69	1767.76	0.085682	0.888293	0.921557	1.10765	0.147502
0.6627	135005.1	129536.8	118510.9	28543.19	37110.25	30372.22	1.361864	0.000366	0.010037	3.989068	1.996052
0.6621	25343.34	32077.19	38054.58	2351.78	6768.18	6955.67	1.274251	0.009985	0.04767	5.939121	2.570249
0.6619	13665.71	9760.55	13481.02	30347.41	75720.68	28214.46	1.196713	0.170324	0.302477	0.274848	-1.86329
0.6612	661.05	4750.8	3025.68	7120.03	1962.3	1770.36	0.285389	0.725397	0.805196	0.77746	-0.36316
0.6608	11101.83	4581.68	14007.99	5638.45	3051.12	10466.53	0.579581	0.379705	0.516757	1.549976	0.632246
0.6604	232.26	722.05	264.57	174.75	205.95	865.81	0.128545	0.97509	0.981305	0.977834	-0.03234
0.6597	239366.9	215127.1	258425.9	158022.6	138180.6	154454.2	1.317261	0.009046	0.044899	1.581956	0.661709
0.6594	4440.45	20285.53	11143.23	9378.87	8326.48	10083.21	0.124954	0.617638	0.71973	1.290791	0.368255
0.6594	1105.06	1133.1	4602.86	2818.55	562.97	3168.01	0.0441	0.94907	0.96329	1.044505	0.06282
0.6594	4709.24	4289.59	2040.21	1844.43	5707.39	8489.17	0.327582	0.490204	0.609818	0.688177	-0.53915
0.6586	27.83	345.02	34.11	227.41	268.56	7.89	0.062197	0.81988	0.874231	0.807685	-0.30814
0.6582	44634.12	36793.42	47732	32588.85	31388.63	34203.92	1.172843	0.078791	0.179768	1.315519	0.395633
0.6562	61656.41	43106.93	50102.99	110702	112125.9	80530.68	1.253664	0.023429	0.079698	0.510506	-0.97
0.6555	676.76	2514.5	430.18	6471.74	7690.55	7153.56	1.220583	0.003916	0.02918	0.169894	-2.55729
0.6552	4362.38	4296.56	6252.57	408.54	47.06	173.75	1.289265	0.015653	0.061804	23.69351	4.56642
0.6543	1024.06	1142.55	1464.24	89.95	462.65	106.73	1.210793	0.005314	0.034337	5.506878	2.461235
0.6527	7230.85	6275.54	4226.53	12134.35	9424.9	7375.31	1.034339	0.096358	0.20673	0.612863	-0.70636
0.6525	4134.11	2302.64	1658.36	2059.3	227.14	1713.46	0.754525	0.221263	0.361637	2.023828	1.017087
0.6525	1296.76	172.45	444.99	4436.98	8202.23	106.76	0.524411	0.261286	0.407399	0.150181	-2.73523
0.6519	203374.2	194382	247559.6	225035	199281.7	204822	0.175546	0.787218	0.85043	1.025713	0.036627
0.6519	16822.49	10937.56	14025.82	5911.9	12230.6	9496.81	0.913945	0.132385	0.254438	1.511828	0.596294

0.6517	359973.5	272740.8	81891.25	10273.62	660892.4	575490.9	0.112537	0.486649	0.608061	0.573217	-0.80285
0.6516	28875.94	33006.34	42161.16	2591.95	3564.49	4303.43	1.355615	0.014343	0.058984	9.946915	3.314249
0.6507	16569.2	19048.12	13457.68	24198.88	28236.24	23770.21	1.221883	0.014184	0.058571	0.643984	-0.6349
0.6502	154691.5	126093.4	162563.5	86072.6	80967.81	60589.78	1.276652	0.008159	0.042258	1.947669	0.961749
0.6496	22486.46	15513.65	13555.65	1991.12	2024.96	2321.67	1.356843	0.030682	0.095123	8.13471	3.024091
0.6491	44405.84	41355.72	49234.31	3773.91	9655.08	7425.82	1.317063	0.000293	0.009591	6.473129	2.694463
0.649	132624.8	113014.9	127516.6	116539.1	108874.7	126225.8	0.57112	0.406998	0.540071	1.06119	0.085683
0.649	7138.23	9314.85	6712.48	1547.54	3931.46	3451.71	1.173774	0.012251	0.053778	2.593921	1.375135
0.6486	16690.67	9573.95	17618.4	8636.29	14642.98	10852.75	0.597311	0.358713	0.499084	1.285685	0.362537
0.6486	46108.16	39371.26	38817.4	49215.12	44908.88	49153.02	1.035197	0.096311	0.20673	0.867528	-0.20502
0.6476	5014	4820.26	5406.43	7583.57	7592.67	8706.56	1.330766	0.007282	0.039627	0.638145	-0.64804
0.6475	78485.1	63511.72	83041.64	27181.27	26336.84	28795.83	1.353278	0.013989	0.058128	2.733905	1.450963
0.6473	46900.69	163840.6	427807.7	59953.95	42941.47	52272.87	0.873699	0.288809	0.431386	4.115203	2.040963
0.6469	51071.28	35824.99	43638.17	2743.72	1181.51	1809.03	1.354447	0.010395	0.048879	22.76396	4.508679
0.6467	4731.41	1486.39	2173.37	104.26	2655.6	2848.67	0.543266	0.523058	0.638648	1.496144	0.581249
0.6462	3000.22	1603.67	1519.77	15166.66	15629.77	15451.25	1.343687	0.000616	0.012889	0.13241	-2.91691
0.6461	5835.56	1659.74	3650.25	2409.22	967.81	2192.68	0.779463	0.259598	0.405151	2.001101	1.000794
0.6451	31972.53	13312	37290.88	33355.89	25773.72	26955.58	0.2566	0.890195	0.922578	0.959229	-0.06005
0.6447	12156.28	9105.97	11826.59	5648.15	9799.41	4126.29	1.008234	0.099303	0.210434	1.690462	0.757417
0.6443	1780.02	2159.95	8081.57	4200.54	1801.33	1806.6	0.33207	0.573523	0.683218	1.539551	0.62251
0.6434	2072.54	602.46	3391.69	12895.89	12107.49	6690.42	1.174441	0.033872	0.102007	0.191416	-2.38522
0.6423	1527.62	1036.54	310.07	12957.45	8518.54	9885.83	1.281367	0.013732	0.057444	0.091647	-3.44776
0.6404	615.87	270.14	2066.08	622.02	1400.76	353.86	0.035969	0.780778	0.845901	1.242128	0.312813
0.64	130186.2	148801.8	130716.7	130574.2	145702.6	135608.1	0.07906	0.928504	0.948745	0.994707	-0.00766
0.64	1131.24	1334.86	2004.66	7338.78	5834.42	9061.62	1.327683	0.017833	0.066896	0.20107	-2.31423
0.6398	497517.9	426951.6	155294.3	420883.3	338386.5	485981.6	0.425782	0.662163	0.753424	0.867105	-0.20572

0.6393	55122.46	38312.43	54295.68	26762.97	37781.92	22920.25	1.118833	0.048756	0.129749	1.689022	0.756188
0.6368	664.7	1619.32	2286.77	138.73	1095.17	1453.17	0.599687	0.365162	0.503999	1.701031	0.76641
0.6367	72797.3	60060.16	75696.97	11833.1	8549.56	11328.66	1.361271	0.004949	0.033105	6.576656	2.717354
0.6367	1348.11	224.22	1558.07	3866.47	6273.5	870.51	0.813108	0.230398	0.37169	0.284311	-1.81446
0.6364	497.14	794.17	523.82	590.25	1233.79	768.55	0.735852	0.314658	0.457061	0.700122	-0.51432
0.6363	3644.18	2612.95	2572.22	1800.74	3351.64	2715.48	0.406813	0.60601	0.710061	1.122205	0.166336
0.6357	16378.9	15412.31	15344.71	2501.67	2325.38	628.95	1.280813	0.000195	0.00848	8.639282	3.110911
0.6353	191.83	318.6	216.61	58.21	271.41	282.39	0.434304	0.673792	0.763071	1.187954	0.24848
0.6343	3309.05	2314.58	3587.49	4843.9	831.91	353.35	0.733538	0.538379	0.653339	1.527762	0.61142
0.6341	7921.97	2257.42	7113.48	357.41	446.96	7809.67	0.801211	0.399982	0.534331	2.007521	1.005415
0.6337	3942.22	1771.01	609.93	2413.17	3658.17	2841.87	0.647968	0.477995	0.601149	0.709414	-0.4953
0.6327	545089.7	434986.5	424436	505102.4	454503.2	493737.2	0.30994	0.724709	0.804936	0.966401	-0.04931
0.6326	499.71	179.46	785.14	1551.55	1632.66	1656.24	1.155199	0.020523	0.073492	0.302515	-1.72492
0.6313	2367.43	1058.2	1786.38	283.28	753.35	112.47	1.132314	0.050595	0.13238	4.535732	2.181335
0.6312	1348.96	1421.34	139.66	1046.27	507.45	1115.6	0.223912	0.872785	0.909942	1.09015	0.124527
0.6311	4414.5	4676.74	5617.11	3133.22	4014.11	2548.76	1.125256	0.04201	0.117782	1.516936	0.60116
0.6305	55453.65	66102.85	99084.87	412.07	24631.88	52520.92	0.799412	0.076587	0.176216	2.844604	1.508228
0.6301	8584.19	14624.85	8202.47	57.08	190.19	245.04	1.338634	0.038317	0.111647	63.80433	5.995582
0.6298	5641.13	11369.52	926.17	6521.94	7741.63	5149.37	0.42597	0.887428	0.921024	0.923962	-0.11409
0.6296	1870.41	3402.41	3416.26	12078.25	13342.74	11524.42	1.323059	0.000227	0.009144	0.235187	-2.08812
0.6294	58412.88	57840.19	65918.54	10567.11	6943.13	6480.44	1.357084	0.000421	0.010815	7.593433	2.924752
0.6287	40129.8	19962.82	40423.91	95974.71	102877.3	95807.89	1.266589	0.00597	0.036092	0.341127	-1.55162
0.6279	15331.9	10716.22	15993.66	17648.17	13662.88	21831.46	0.726306	0.276007	0.420228	0.791114	-0.33804
0.6278	303436.4	237394.7	241137.6	378604.8	367670.9	355189.6	1.244259	0.029014	0.091844	0.709935	-0.49424
0.6271	15765.37	15971.72	13788.09	33405.96	30377.28	30202.56	1.356257	0.000452	0.011141	0.484384	-1.04578
0.6268	1161.15	2664.45	3390.66	1214.03	670.89	4456.78	0.36248	0.842585	0.888712	1.137906	0.186382

0.6256	309299.1	291639.3	337621.3	119325.4	126211.3	129371.9	1.365261	0.003616	0.028133	2.503436	1.323909
0.6254	292.77	450.17	580.19	1892.85	222.16	2281.68	0.65647	0.245016	0.388231	0.300938	-1.73246
0.6251	2741.92	2736.31	4501.57	107394.9	34351.55	45560.21	1.325581	0.121243	0.239797	0.053281	-4.23025
0.6238	496400	460326.4	562155.6	125476.7	126982.8	115271.7	1.367196	0.00541	0.034575	4.130413	2.046286
0.6228	308734.3	275937.7	318293.3	22576.81	3663.7	29108.17	1.265642	0.000191	0.008427	16.31413	4.02805
0.6212	128.53	406.13	804.16	896.6	91.14	335.32	0.115979	0.987267	0.990904	1.011912	0.017084
0.6211	2808.9	1086.78	1161.18	909.04	500.03	126.86	0.991178	0.160877	0.290498	3.292377	1.719129
0.621	4752.69	25980.59	13865.14	181.43	1254.78	800.45	1.226507	0.148146	0.273897	19.93974	4.317575
0.6203	89607.44	91030.11	93341.24	125990.5	166620.1	114765.4	1.184127	0.10522	0.218886	0.672545	-0.5723
0.6197	63.88	192.93	356.77	1081.46	634.14	854.75	1.152515	0.018276	0.068089	0.238715	-2.06664
0.6194	2800.5	3449.9	5022.26	2245.02	4140.12	950.33	0.722244	0.319003	0.4615	1.536733	0.619867
0.6193	1062.1	130.6	696.55	194.52	601.02	101.07	0.536494	0.362292	0.501185	2.107103	1.075261
0.6193	45569.11	45272.03	49804.35	38963.09	34495.38	40640.1	1.203072	0.021356	0.075509	1.232667	0.301783
0.6191	12574.34	10845.82	9118.78	13162.08	13544.37	14194.89	1.070155	0.097288	0.20776	0.795547	-0.32998
0.6189	9782.63	80.52	12098.13	116.61	89.65	65.54	0.954538	0.188488	0.323655	80.79941	6.336273
0.6181	383.73	526.15	2166.05	62066.91	48280.71	73750.31	1.331718	0.014133	0.058571	0.016708	-5.90331
0.618	18329.71	12193.45	16366.83	14592.24	15526.84	13692.9	0.290524	0.633731	0.731042	1.070255	0.097955
0.6178	8037.45	1678.69	23082.29	2399.31	1511.76	1878.47	0.873988	0.29169	0.434396	5.665118	2.502106
0.6178	39352.24	39057.4	44210.72	108327.7	108140.2	105674.6	1.367916	5.21E-05	0.004503	0.38064	-1.3935
0.6165	28950.15	30442.3	35273.56	33531	30515.98	26116.07	0.357846	0.629792	0.728766	1.049942	0.07031
0.6164	17566.68	12052.63	4472.36	682.27	316.06	394.43	1.314363	0.102765	0.215257	24.47778	4.613401
0.6162	736.59	627.13	179.22	80.17	79.93	44.55	1.219597	0.119598	0.237615	7.539409	2.914451
0.6158	9314.31	7472.39	4956.92	28564.13	36366.86	52474.76	1.314508	0.041688	0.117292	0.185201	-2.43284
0.615	10541.86	8515.59	19915.14	2574.48	9037.69	3792.81	1.021388	0.141927	0.266664	2.52987	1.339063
0.6148	74298.19	50455.55	53269.24	12112.47	19132.66	19489.65	1.310084	0.021761	0.076035	3.508894	1.811016
0.6147	3231.62	3627.28	3903.87	15082.23	16578.95	13725.26	1.364913	0.003468	0.027553	0.237136	-2.07621

0.6127	125847.4	103742.9	113805.1	81484.08	76613.23	75090.89	1.30875	0.021498	0.075726	1.47261	0.558376
0.6124	199286.3	253955	197502	237439.6	326767.4	254906	0.916109	0.174158	0.30773	0.794449	-0.33197
0.611	31828.92	12295.9	17321.88	51016.08	42952	38983.35	1.133228	0.034569	0.10321	0.462174	-1.11349
0.6102	164104.1	100860.1	99661.12	273922.3	118817.4	263187	0.897808	0.182028	0.31713	0.555893	-0.84712
0.6099	65475.55	21474.03	11037.4	103791.9	238023.6	69968.19	1.070223	0.169669	0.301626	0.237957	-2.07122
0.6098	30787.54	25027.02	33621.81	73021.8	59913.39	75105.54	1.326512	0.004952	0.033105	0.429898	-1.21793
0.6094	5464.57	1429.14	3269.25	4661.44	5561.27	1985.39	0.309999	0.689511	0.77764	0.832477	-0.26452
0.609	6641.95	5297.92	8593.77	8366.43	9312.72	7699.64	0.829973	0.229586	0.370693	0.809087	-0.30563
0.6075	171064.5	180463.2	159375.7	25652.89	21173.1	26496.41	1.36844	0.000917	0.014857	6.967903	2.800724
0.6069	2211.99	2815.29	1451.6	88.07	276.04	6461.6	0.632177	0.96137	0.972095	0.949188	-0.07523
0.6068	221.16	83.41	75.32	62.04	115.02	148.63	0.140936	0.758001	0.830391	1.166416	0.222082
0.6065	243731	255119.9	301270	381048.7	394315.1	385428.4	1.296776	0.016999	0.065056	0.689289	-0.53682
0.6062	38259.35	22839.1	39770.2	52401.44	51776.57	53915.81	1.099218	0.069946	0.165097	0.63803	-0.6483
0.6057	3054192	4832228	4200171	3092223	3374510	3223024	0.815948	0.262954	0.408452	1.247357	0.318875
0.6054	28204.96	20613.45	40366.71	57765.37	53159.97	25107.12	0.708071	0.270648	0.415793	0.655616	-0.60908
0.605	25659.21	21932.03	43643.67	10631.66	7661.53	14650.88	1.200658	0.090696	0.198664	2.769388	1.469567
0.6046	333.22	162.63	1691.54	2256.56	5171.98	193.72	0.555193	0.336926	0.477732	0.286974	-1.80101
0.6017	7930.73	4322.04	13285.92	4275.4	3471.85	4386.22	0.958657	0.227218	0.367784	2.104813	1.073692
0.6016	2290.4	12672.21	11044.02	10396.38	21604.1	15535.3	0.768769	0.191422	0.32722	0.547096	-0.87013
0.6012	330845.5	288005.9	1435505	282951.1	547422.4	523768.1	0.213164	0.600938	0.705682	1.517092	0.601308
0.5996	11429.79	15426.87	24446.95	4203.47	1758.89	4269.16	1.25351	0.064903	0.157511	5.014271	2.32604
0.5974	4104.43	3124.23	947.7	3286.18	3047.65	865.83	0.12273	0.801586	0.861532	1.135659	0.18353
0.5974	9303.96	1904.02	3557.11	1868.02	3274.2	5504.71	0.251718	0.620346	0.721277	1.386793	0.471753
0.5969	360.66	182.32	31.89	78.51	344.4	173.92	0.2194	0.955428	0.967853	0.963206	-0.05408
0.5965	1095.44	408.14	79.03	847.46	588.12	923.06	0.66216	0.485091	0.607444	0.670984	-0.57565
0.5955	2319.23	1763.26	255.98	1037.97	1178.37	608.78	0.10623	0.503286	0.621304	1.535676	0.618874

0.5954	75765.39	49809.48	66450.19	43807.47	44550.86	46650.61	1.102331	0.127595	0.248408	1.422314	0.50824
0.5952	36477.36	15941.71	44017.53	38452.53	42735.15	31765.19	0.472091	0.589647	0.695608	0.853777	-0.22807
0.5947	65558.97	50218.28	48415.12	32844.73	38799.39	46661.66	1.046006	0.092096	0.200825	1.387864	0.472867
0.5946	7789.97	25454.82	1730.19	12914.72	12156.28	9876.52	0.421067	0.999095	0.999324	1.000786	0.001133
0.5939	7770.12	7102.21	6658.31	29082.85	27397.22	29225.22	1.369553	5.11E-05	0.004503	0.251217	-1.99299
0.5934	2942.97	4889.2	3929.68	1452.03	2015.5	1287.24	1.256605	0.039214	0.112605	2.473695	1.306668
0.5916	17291.1	54471.95	28253.6	66.07	386.68	93.34	1.336563	0.095125	0.205075	183.1505	7.516886
0.5915	287.26	150.18	3919.45	318.04	319.44	295.56	0.382384	0.45274	0.580001	4.669564	2.223288
0.5891	90430.64	71017.82	62077.22	64268.9	80458.86	580.91	0.620224	0.400577	0.534797	1.538282	0.62132
0.589	379.86	381.32	674.34	1032.08	1239.73	332.36	0.651497	0.289892	0.432754	0.551239	-0.85925
0.5887	22127.03	11692.93	28163.62	24686.03	20835.02	287978.9	0.676113	0.413676	0.545932	0.185858	-2.42773
0.5867	420.51	379.32	799.47	1479.13	303.9	2090.17	0.634603	0.282724	0.426423	0.412914	-1.27609
0.5863	65478.28	67431.84	57940.72	64234.7	96494.63	75798.02	0.85742	0.243497	0.387091	0.806887	-0.30956
0.5854	1041.6	5253.08	6357.57	7126.8	1022.48	7032.92	0.101193	0.761647	0.83223	0.833361	-0.26299
0.5841	8645.29	5131.03	3944.31	301.76	2454.36	1532.47	1.06868	0.06927	0.164027	4.132041	2.046855
0.5838	395.86	384.83	193.6	5408.43	4197.67	4745.46	1.351621	0.004863	0.032817	0.067887	-3.88071
0.5803	2123.03	3098.74	1767.42	5395.39	5247.2	4663.2	1.264702	0.007791	0.041018	0.456637	-1.13088
0.5801	641.77	14663.85	12103.21	3195.61	1386.6	2308.53	0.495447	0.252473	0.397702	3.977632	1.99191
0.5801	12787.03	438.48	2688.57	1716.96	8698.57	6443.86	0.373951	0.946314	0.961661	0.94393	-0.08325
0.58	4291.87	4204.84	4675.42	1544.64	465.17	215.35	1.205349	0.006787	0.038625	5.919633	2.565508
0.5777	30594.21	28674.94	19834.05	8748.43	5787.81	7115.39	1.322388	0.022868	0.078366	3.653452	1.86926
0.5772	2302.25	1192.39	1121.73	835.12	206.45	697.23	1.011919	0.112309	0.228705	2.654917	1.408667
0.5763	38975.83	19075.67	20730.96	32355.51	34842.64	44906.24	0.861601	0.224749	0.364986	0.70276	-0.5089
0.5757	10337.57	10063.5	11722.59	5170.21	4768.69	643.57	0.999139	0.027445	0.088333	3.035554	1.60196
0.5736	327.78	96.36	632.63	4162.16	14752.98	701.24	1.06418	0.280776	0.424672	0.053872	-4.21433
0.5719	17827.1	10174.76	14829.78	25759.95	31935.18	30310.6	1.239164	0.007115	0.039357	0.486691	-1.03892

0.5718	7068.33	4181.33	5216.81	135.1	319.69	317.46	1.343763	0.024576	0.081963	21.32272	4.41432
0.5713	10747.11	13681.63	12372.26	30352.52	8386.42	10284.33	0.201878	0.621576	0.722135	0.750684	-0.41372
0.5705	75770.09	63717.12	144492.6	40570.34	277.02	105052.3	0.682369	0.311351	0.45421	1.946405	0.960812
0.5691	42247.81	35334.79	40439.33	3297.17	4997.77	3499.1	1.362884	0.002058	0.020822	10.00691	3.322925
0.5678	28901.78	23712.39	26452.52	20216.7	19218.42	14436.57	1.171999	0.023818	0.080475	1.467685	0.553543
0.5671	15122.11	20967.91	20435.44	8057.11	15299.14	7056.78	1.082919	0.058757	0.147014	1.858594	0.894211
0.5668	69278.74	66553.22	64010.87	6520.88	9285.95	3203.69	1.329867	1.57E-05	0.003622	10.51222	3.393996
0.5663	32118.91	36429.98	43794.29	14946.42	16008.43	12627.18	1.334717	0.0154	0.061135	2.577741	1.366107
0.566	2046.48	19549.9	13078.75	25892	211.18	552.95	0.642962	0.803683	0.862445	1.300831	0.379434
0.5649	2834.22	12005.41	4937.62	8930.25	8487.11	4022.77	0.270263	0.872436	0.909942	0.922441	-0.11647
0.5626	9047.69	9079.24	8611.24	7406.02	5274.14	7664.98	1.050787	0.101284	0.212668	1.314229	0.394217
0.5624	2401.75	3667.19	4014.58	1658.32	761.58	495.65	1.190606	0.020114	0.07222	3.458531	1.790159
0.5613	13921.62	12177.56	17255.99	9693.8	11551.69	14947.15	0.684882	0.327374	0.469255	1.1979	0.260508
0.5612	85378.98	492235.1	113261.1	695933	711980.5	589160.2	1.073993	0.069951	0.165097	0.345944	-1.53139
0.5611	185722.7	232166.3	177110.2	227647.9	299806.8	230225.6	0.961474	0.14354	0.268234	0.785291	-0.3487
0.5593	6370.61	4769.38	7068.13	14224.66	51356.8	13525.52	1.120327	0.245512	0.388734	0.230171	-2.11922
0.5573	100387.5	263.81	102514	131.78	42969.61	121.89	0.610868	0.251317	0.396199	4.700368	2.232774
0.5568	3437.38	2522.15	7507.11	4583.11	4015.02	4381.47	0.147604	0.925518	0.947026	1.037523	0.053144
0.5562	3196.99	6481.99	7568.19	4660.1	4269.17	12904.97	0.250072	0.658263	0.74977	0.789914	-0.34023
0.5559	3728.83	2891.33	3620.93	6829.41	6146.05	3619.19	1.000997	0.154915	0.283016	0.617132	-0.69635
0.5549	280.46	44.03	178.78	52.99	20.44	55.15	0.936528	0.2072	0.346446	3.914061	1.968666
0.5489	164.28	694.55	642.11	4786.77	19178.94	1555.3	1.132442	0.27739	0.42144	0.058812	-4.08775
0.5488	10780.59	10462.04	5419.41	15282.6	11163.81	7039.5	0.458534	0.48684	0.608061	0.796217	-0.32877
0.5461	259.98	15727.98	15202.43	1150.23	957.11	11978.3	0.221384	0.417647	0.548303	2.21434	1.146876
0.5458	94261.61	24185.66	28020.31	38235.09	44686.3	39759.26	0.033692	0.761077	0.83188	1.193893	0.255674
0.5434	6338.22	5090	7896.51	2220.64	774.44	977.69	1.270585	0.01052	0.049344	4.864296	2.282231
0.5423	1184.73	5879.17	6260.85	3016.35	6127.78	11497.02	0.504316	0.46382	0.589615	0.645543	-0.63141

0.5414	206758.2	488226.2	299820.6	309448	412490.7	337734.8	0.300231	0.824984	0.878598	0.938785	-0.09113
0.5414	274465.7	491216.1	450779	746782.6	81947.5	428261.6	0.275101	0.951982	0.965493	0.967756	-0.04729
0.5398	20731.34	7460.13	10281.51	19471.89	15118.83	13698.64	0.600035	0.515643	0.632083	0.796718	-0.32786
0.5386	467.22	587.96	747.41	729.01	462.61	95.45	0.605002	0.460119	0.586621	1.400538	0.485981
0.5373	141.19	133.15	105.6	217.66	190.58	198.91	1.271481	0.006158	0.036768	0.625776	-0.67628
0.5373	10089.78	5705.35	8299.47	6222.24	4831.41	4417.93	1.010924	0.139165	0.26352	1.557346	0.639089
0.5341	1241.52	1720.55	840.39	1697.69	1625.55	2105.55	0.908996	0.156797	0.285378	0.700425	-0.5137
0.5316	28475.07	19451.92	25914.78	54198.97	53981.41	85530.72	1.274685	0.054656	0.139652	0.381195	-1.3914
0.5308	1426.18	1044.86	1530.3	2558.64	2710.42	1602.38	1.086547	0.094066	0.203593	0.582315	-0.78013
0.5294	5345.05	3144.97	5110.49	7202.16	6817.78	4175.5	0.703519	0.269762	0.415657	0.747468	-0.41992
0.5293	9183.23	5693.96	7764.46	27546.48	37459.57	39509.29	1.337169	0.012973	0.055606	0.216635	-2.20666
0.5279	420.73	7886.18	692.55	1681.4	983.68	1424.15	0.006046	0.572463	0.68254	2.200771	1.138009
0.5244	11532.03	10314.56	9732.43	16063.06	15361	12336.98	1.180879	0.052821	0.136564	0.721624	-0.47068
0.5213	1457.95	2275.68	2420.85	2765.31	1026.23	684.51	0.646719	0.491727	0.610666	1.37498	0.459411
0.5189	2354.06	1190.24	2555.78	370.28	874.57	1847.16	0.850358	0.174161	0.30773	1.972853	0.980283
0.5179	108.78	364.69	166.5	889.71	56.73	530.94	0.338324	0.368412	0.506354	0.433179	-1.20696
0.5176	1221.6	733.97	1296.62	1084.23	1790.65	1217.6	0.615688	0.375003	0.512425	0.794675	-0.33156
0.5159	2272.71	1094.17	4671.82	2658.19	3018.27	1170.6	0.095876	0.761008	0.83188	1.174037	0.231478
0.5143	2463.99	4024.86	5458.19	4165.26	4228.24	6268.75	0.556316	0.461486	0.587622	0.814816	-0.29545
0.5134	24629.56	14762.24	26115.07	11555.35	4244.9	11796.81	1.06142	0.050103	0.131328	2.37369	1.247132
0.5058	2281.07	4674.04	6074.52	2404.58	2771.2	5100.54	0.380838	0.548243	0.663278	1.267928	0.342472
0.5049	354.19	942.97	405.15	232.69	1144.84	162.58	0.372302	0.891877	0.924047	1.105317	0.14446
0.5033	8660.25	5043.57	2052.57	47319.97	38873.35	43607.55	1.286458	0.000352	0.010018	0.121389	-3.04229
0.5031	116676.1	99830.44	113864.2	119124.4	21237.15	96236.12	0.632451	0.401575	0.535802	1.39634	0.48165
0.5022	2786.31	2545.85	5421.36	1332.35	5948.09	1417.32	0.48482	0.724004	0.80447	1.236355	0.306093
0.502	444.71	789.94	563.01	673.8	1192.36	28.78	0.402264	0.933743	0.95209	0.948663	-0.07603

0.5018	26186.61	25099.2	31180.71	29686.24	31869.43	27361.36	0.601431	0.405105	0.538698	0.927455	-0.10865
0.5018	882.08	1323.46	1405.08	248.36	236.65	787.19	1.121993	0.033503	0.10119	2.838091	1.504921
0.8692	1518.67	4179.41	1101.26	6984.77	1869.12	4882.21	0.752178	0.272107	0.417205	0.494998	-1.01451
0.8494	15255.48	52120.29	14481.09	3414.58	13752.44	5673.3	1.004761	0.250593	0.395201	3.583875	1.84152
0.8202	438.52	2076.18	3246.79	1430.62	469.83	1042.82	0.45165	0.370306	0.507798	1.957513	0.969022
0.804	64372.43	185653.1	58228.88	40946.56	22491.15	42964.72	1.025394	0.244523	0.388042	2.897062	1.534591
0.7898	15351.93	25691.76	24223.44	30544.34	24628.67	28720.92	0.841805	0.187149	0.322239	0.777972	-0.36221
0.7779	20870.86	2600.08	9783.16	9264.14	6301.94	26677.29	0.321606	0.736435	0.814545	0.787203	-0.34519
0.7722	50110.44	42748.89	40564.99	73499.28	63377.76	6794.46	0.293047	0.884999	0.919378	0.928676	-0.10675
0.7617	133.26	735.82	123.95	787.23	2937.56	3785.16	1.129705	0.12909	0.250647	0.132229	-2.91889
0.7607	28309.22	32417.01	22036.41	22706.7	33927.51	35771.66	0.374855	0.563815	0.675184	0.895643	-0.159
0.7589	326577.5	324419.7	245576.4	114585.5	128721.1	133048	1.333918	0.019239	0.070294	2.382258	1.25233
0.7494	28139.65	66406.79	33748.73	14915.31	26345.62	18014.36	1.045302	0.186879	0.322106	2.164395	1.113964
0.7389	23.7	173.14	10.08	415.89	212.03	55.37	0.838955	0.26849	0.414723	0.302829	-1.72342
0.7242	10072.71	6633.59	8984.13	1336.92	2715.37	3233.6	1.249801	0.011853	0.052522	3.526052	1.818054
0.7147	358477.6	358931.3	346009.7	110559.8	105849.6	97373.63	1.370176	1.81E-06	0.001767	3.389025	1.76087
0.9994	167713.6	169530	154388.4	48184.68	599.72	48712.2	0.927495	0.009581	0.046561	5.042555	2.334155
0.9948	59501.31	45964.36	48678.27	6011.39	8584.73	15652.5	1.299046	0.001936	0.020396	5.0959	2.349337
0.9729	133345.4	121348.7	120421.1	126571.7	130003.3	113899.3	0.169682	0.821912	0.875755	1.012527	0.017961
0.9588	1046442	843033.7	937412.5	92644.18	92583.1	86908.08	1.37075	0.004694	0.032349	10.3878	3.376818
0.9581	315.1	470.91	597.52	11069.82	147.83	137.09	0.142816	0.457843	0.584403	0.121846	-3.03687
0.9576	1564.1	4949.41	469.21	2004.08	3245.99	933.75	0.152421	0.871081	0.909132	1.129192	0.175291
0.9544	3338.1	1262.83	1457.38	4337.71	5096.9	5253.77	1.162258	0.033874	0.102007	0.412456	-1.27769
0.9412	81389.64	57389.23	70396.26	73956.2	68912.91	76948.29	0.365862	0.667953	0.757836	0.951586	-0.07159
0.933	154326.6	151831.7	144442.4	137495.6	142005.8	159039.8	0.388517	0.61835	0.720171	1.027499	0.039137
0.9115	717.04	47.69	287.78	24786.02	20348.08	23999.74	1.299272	0.003091	0.0261	0.015224	-6.03749

0.8993	18532.36	14574.67	17546.87	12328.39	12195.62	15749.98	0.991177	0.106107	0.219934	1.257732	0.330825
0.8972	1527.51	3441.95	5427.24	1773.77	1090.77	360.34	0.98696	0.157183	0.285959	3.223903	1.688808
0.8645	6919.62	6257.16	7986.77	7732.69	3906.46	13050.14	0.083948	0.703155	0.787526	0.857196	-0.2223
0.8509	4790.44	8153.82	5159.82	19546.35	20145.4	17780.14	1.319172	0.001005	0.015104	0.315008	-1.66654
0.8327	2239.03	3076.17	101.76	194.53	2458.74	2179.53	0.068458	0.872671	0.909942	1.120874	0.164624
0.8287	76516.6	80176.3	97932.93	98050.28	84860.92	99568.03	0.693817	0.321992	0.464214	0.901397	-0.14977
0.8161	59471.05	42481.73	57773.98	4230.32	4411.93	5524.17	1.364301	0.01182	0.052522	11.27503	3.495059
0.8104	43820.85	33820.74	37667.23	176322.6	153848.7	166070.8	1.363826	0.000606	0.012759	0.232364	-2.10554
0.7944	44768.34	7812.93	5280.91	1786.15	47924.89	226.08	0.561889	0.902462	0.931084	1.158701	0.212508
0.7826	63171.98	52203.11	47935.43	46663.9	44508.79	56033.28	0.586758	0.40657	0.539991	1.109401	0.149782
0.7825	25228.09	15297.75	23806.83	7982.2	4639.04	2557	1.239081	0.0185	0.068551	4.23848	2.083547
0.7819	608953.5	455129.8	661018.5	177470.1	170219.3	187799.6	1.345961	0.022816	0.078306	3.221545	1.687753
0.7813	4293.66	31389.6	8236.91	5182.85	7128.61	4980.69	0.616337	0.40385	0.537686	2.539891	1.344766
0.7729	8199.63	2410.39	1191.05	8779.14	7326.38	5535.16	0.846317	0.26664	0.412497	0.545319	-0.87483
0.7708	6723.82	4550.68	7649.3	3910.18	169.88	3276.37	0.829516	0.062349	0.152944	2.572416	1.363124
0.7705	510.47	725.25	441.97	648.08	468.83	2270.53	0.591671	0.425178	0.553083	0.495268	-1.01372
0.7652	101694.4	125856.7	133766.9	145279.3	179274.3	175288.3	1.150062	0.033416	0.101036	0.722865	-0.4682
0.7614	11745.67	7551.18	14519.41	17405.81	13395.5	9839.84	0.485995	0.487947	0.608746	0.832069	-0.26522
0.7538	41400.82	50520.38	48649.8	607.41	1117.13	355.38	1.356636	0.003435	0.027425	67.58481	6.078627
0.7518	7290.39	3156.46	7060.88	8185.9	12464.42	14470	1.03249	0.068181	0.162406	0.498507	-1.00431
0.7492	25076571	33711599	25876894	10726181	9784851	10939903	1.345368	0.021798	0.076035	2.691973	1.428664
0.7488	10229.75	7739.87	7576.06	38161.59	33749.63	36659.87	1.359456	0.000155	0.007197	0.23529	-2.08749
0.7471	4467.59	3010.91	2873.44	1670.32	807.81	1962.92	1.131456	0.039547	0.112965	2.330967	1.220928
0.7468	177131.6	147401.3	164543.1	184503.3	162815.9	173645.4	0.615674	0.379714	0.516757	0.938789	-0.09113
0.7439	16743239	22603242	17877490	8582697	8643455	9477111	1.330047	0.027179	0.087875	2.142958	1.099604
0.7424	11559.83	8618.13	3648.8	1350.54	2264.25	644.74	1.188261	0.10034	0.211903	5.593753	2.483817
0.7394	17105.36	12833.97	13657.89	7159.95	9019.79	7303.74	1.286849	0.021681	0.075985	1.856506	0.89259

0.7383	179801.5	95462.34	83502.06	91139.71	98682.26	179550.1	0.087186	0.936162	0.953887	0.971286	-0.04203
0.7379	118191.2	103809.5	138104.3	15364.05	28226.41	15362.29	1.335904	0.003909	0.02918	6.108366	2.610786
0.7377	3473.23	3192.37	3214	3083.87	3181.82	3354.69	0.465692	0.512085	0.62967	1.026945	0.038359
0.7368	13278.74	13866.74	15567.87	121406.3	114478.2	119813.7	1.371291	0.000118	0.00641	0.120083	-3.05789
0.7358	7666.39	7692.87	1764.06	1385.49	2410.65	5762.82	0.548841	0.355973	0.497078	1.791337	0.841037
0.7357	18846.34	20188.15	20589.1	4732.47	8343.38	5217.55	1.317794	0.002071	0.020844	3.259295	1.70456
0.732	794.46	1168.42	867.32	3150.71	1784.5	4512.46	1.222302	0.104389	0.217719	0.299566	-1.73905
0.7316	2377.65	1273.94	3324.97	1571.57	1952.98	2890.36	0.087076	0.807055	0.86415	1.087554	0.121087
0.7314	1899.14	1255.46	2787.92	854.93	1166.7	1390.68	0.953294	0.189881	0.325095	1.741495	0.800326
0.7313	11557.54	10418.63	11795.18	4729.75	4430.17	2136.08	1.253805	0.003892	0.029148	2.989673	1.579988
0.7306	12555.24	18341.26	17999.27	18007.94	15315.36	17685.12	0.272003	0.756318	0.828965	0.958582	-0.06103
0.7299	757.69	2706.12	540.01	2973.51	211.34	309.4	0.397696	0.888968	0.921557	1.145831	0.196394
0.7284	53420.98	50335.02	46139.02	39062.65	45123.7	39496.76	1.148529	0.038727	0.112091	1.211928	0.277304
0.7284	26960.03	4677.38	69447.41	61522.57	98639.25	55484.67	0.823849	0.184261	0.319362	0.468752	-1.0931
0.7276	85220.91	75582.54	46321.17	91269.64	69630.78	102343.4	0.702393	0.286452	0.429679	0.786817	-0.3459
0.724	8838.59	4966.83	2515.98	9433.32	6877.14	8215.55	0.788841	0.273513	0.418331	0.665473	-0.58755
0.7234	4457.48	5968.42	205.27	1709.39	2731.03	2602.93	0.168606	0.561889	0.673622	1.509391	0.593967
0.7231	1316.34	2287.86	1187.98	915.69	660.49	949.59	1.088983	0.154411	0.28245	1.897314	0.923959
0.7224	2320.55	1897.88	3500.75	2474.23	3015.58	3535.46	0.543047	0.493914	0.612859	0.855285	-0.22552
0.7213	2966.17	1545.09	1353.06	1042.29	2912.4	2285.19	0.066962	0.875391	0.912224	0.939813	-0.08955
0.7212	27524.41	41117.99	31557	53177.58	60682.7	39910.13	1.072179	0.080029	0.181144	0.651617	-0.6179
0.7197	42816.06	64799.5	40180.04	41288.4	47563.01	25312.72	0.665854	0.336457	0.47757	1.294589	0.372494
0.7194	18899.82	18254.06	19808.49	12672.99	17797.11	11757.98	1.06057	0.11342	0.229846	1.348922	0.431807
0.7179	35156.44	5332.08	22483.7	154327.9	185323.8	103921.2	1.206265	0.022732	0.078146	0.141966	-2.81638
0.7139	12594.85	15724.66	14518.47	20031.56	22219.38	21893.08	1.293146	0.004324	0.031002	0.667841	-0.58242
0.7136	17926.89	8185.86	14393.43	14602.28	26428.72	16647.54	0.731449	0.28754	0.430717	0.702275	-0.50989

0.7126	2104.36	445.18	250.23	3003.1	99.2	3159.01	0.239467	0.386639	0.523079	0.447154	-1.16116
0.7123	27095.11	28664.07	27034.08	11206.5	841.71	681.58	1.14259	0.019548	0.070831	6.503898	2.701305
0.7117	47253.54	99011.92	44753.01	34153.14	48056.66	64833.31	0.460524	0.513356	0.630521	1.299064	0.377473
0.7116	23451.27	22397.63	28952.35	13982.82	13223.21	14110.39	1.323267	0.029663	0.09309	1.810448	0.856347
0.7115	3189.3	21102.16	20615.23	1492.94	7717.7	7749.97	0.661166	0.25034	0.395088	2.647705	1.404742
0.7112	596227	434939.3	503552.3	368760.7	291974.4	313781.8	1.224475	0.039025	0.112432	1.574851	0.655215
0.7111	150815.4	130442.4	78229.69	94874.79	181644.4	129196.7	0.303258	0.667429	0.757834	0.886057	-0.17453
0.7101	69575.66	53526.97	91576.68	66586.91	67688.21	66245.06	0.195224	0.710458	0.793667	1.070612	0.098436
0.7095	1549.55	11989.92	5541.2	13923.95	2884.98	3032.28	0.047261	0.96012	0.971767	0.961669	-0.05639
0.7088	1499.06	3847.16	707.06	1033.05	35622.15	10884.42	0.761311	0.310753	0.453793	0.127331	-2.97334
0.7082	7560.12	8108.88	183.55	8459.34	9984.08	6480.57	0.629763	0.362221	0.501185	0.636036	-0.65282
0.7081	3198.61	2200.51	700.53	8772.48	5099.26	8371.41	1.137594	0.023999	0.08081	0.274226	-1.86656
0.7081	299.31	273.37	511.2	643.02	1505.09	898.35	1.165081	0.115058	0.232365	0.355783	-1.49093
0.7061	1573.46	1467.69	1862.5	1680.05	682.04	1090.74	0.834269	0.231549	0.372856	1.420183	0.506077
0.7045	4214.3	2173.75	4203.06	633.61	85.17	556.21	1.190987	0.03773	0.110452	8.306818	3.054296
0.7041	58167.4	49988.17	55779.69	239687.5	250419.9	269266.2	1.369297	0.000962	0.014975	0.215882	-2.21168
0.704	1015667	1398587	1338140	1737975	1310199	1547302	0.853209	0.176879	0.310643	0.816541	-0.2924
0.7001	293.97	576.34	27.68	342.82	111.06	194.78	0.108055	0.665803	0.756973	1.384377	0.469237
0.6995	20528.61	16310.52	19619.32	37297.53	35383.21	34767.66	1.338881	0.000981	0.014975	0.525447	-0.92838
0.6969	968.45	86.84	947.14	5076.26	4428.05	4618.72	1.140302	0.000673	0.013514	0.141785	-2.81823
0.6966	1481399	2299492	2058402	1009873	1853703	1891535	0.578869	0.392878	0.528919	1.228003	0.296315
0.6941	11280.44	7208.63	8503.24	17745.03	12705.97	17915.31	1.184021	0.031979	0.097822	0.558081	-0.84145
0.693	438.35	2671.49	3479.9	2889.98	5244.87	2667.37	0.668158	0.317093	0.459635	0.610036	-0.71303
0.6917	34468.97	20749.1	21196.76	44922.95	44343.11	38413.39	1.155933	0.045192	0.123038	0.59849	-0.7406
0.6912	38250.37	33410.88	36280.05	70644.05	64276.56	69287.94	1.357172	0.000309	0.009688	0.528584	-0.9198
0.6908	107.87	521.5	864.66	770.49	43.96	134.61	0.487591	0.596676	0.701621	1.574221	0.654638

0.69	4815.47	7468.36	8312.86	48560.69	47944.37	46435.1	1.353114	2.98E-05	0.003892	0.144093	-2.79493
0.6891	4537.52	3979.31	7019.14	7256.73	7632.31	1935.85	0.080831	0.848505	0.892527	0.923392	-0.11498
0.6887	953.5	524.63	622.32	507.2	10940.31	1170.36	0.655208	0.407756	0.54041	0.166466	-2.5867
0.6887	425.96	56.27	2971.99	712.67	2031.5	1620.66	0.603531	0.782488	0.846789	0.791376	-0.33757
0.6886	108832.2	90141.36	100094	119076.3	136453.3	124608	1.197913	0.022265	0.076931	0.786735	-0.34605
0.6876	55385.43	71581.26	59124.19	67346.98	65726.93	65368.03	0.56613	0.489525	0.609495	0.93776	-0.09271
0.687	485.6	583	536.41	203.21	31.51	691.71	0.752096	0.371061	0.508155	1.732468	0.792828
0.6858	97.14	213.48	832.12	148.91	336.65	693.44	0.205531	0.967686	0.976648	0.969245	-0.04507
0.6834	20536.02	19592.62	18672.8	14632.71	20235.6	20060.2	0.458404	0.560933	0.673398	1.070509	0.098296
0.6832	21624.35	22410.71	30010.47	21719.23	18044.75	28014.6	0.376934	0.625312	0.725276	1.092462	0.127583
0.6832	4035.94	920.34	4608.8	11926.32	1923.53	947.9	0.05845	0.675729	0.764867	0.646387	-0.62953
0.6804	423.19	1024.05	937.68	1137.01	1127.59	672.45	0.485655	0.491283	0.610666	0.812012	-0.30043
0.6804	339.15	59.22	43.59	522.19	325.19	502.22	1.041485	0.067339	0.161192	0.327475	-1.61054
0.68	138459.5	163535	124167.2	273159.1	326220.2	277209.1	1.325352	0.003066	0.026002	0.486159	-1.0405
0.6796	55045.58	51102.61	50541.16	25724.34	28681.69	30134.09	1.352241	0.000245	0.009295	1.853432	0.890199
0.6785	1750.13	1885.64	635.43	762.96	899.79	1717.03	0.297771	0.583434	0.691084	1.263751	0.337712
0.6777	6391.54	4171.65	861.57	5855.26	146.81	150.08	0.733272	0.520249	0.636476	1.857035	0.893001
0.6771	47087.1	34179.25	48617.84	77173.42	58664.74	49355.3	0.977862	0.139627	0.263735	0.701343	-0.51181
0.6768	86477.65	68680.94	84705.49	522659.7	471894	444438.5	1.366377	0.002059	0.020822	0.166689	-2.58477
0.6767	868317.3	903426.1	902045.1	909410	848191.9	789000.6	0.694003	0.347871	0.488603	1.049943	0.070312
0.6751	61077.79	66047.65	55638.26	22823.97	17262	5890.45	1.205611	0.003107	0.026142	3.975162	1.991014
0.6748	137048.1	167790.8	141636.6	36847.48	38951.87	37247.84	1.36679	0.007135	0.03937	3.949461	1.981656
0.6739	7518.7	5556.77	5873.02	541.1	727.41	595.94	1.364573	0.010725	0.049629	10.16305	3.345261
0.6732	1465.77	1438.85	1596.16	982.08	1045.39	858.01	1.316291	0.001951	0.020396	1.559803	0.641364
0.6723	2602.99	1839.02	1273.42	493.33	568.86	102.74	1.147814	0.045219	0.123038	4.906243	2.294619
0.6719	811.24	238.42	340.54	275.08	164.72	187.58	0.89748	0.284545	0.427471	2.215882	1.147881

0.6705	816.16	1853.44	1475.85	2104.67	1698.88	1628.43	0.723704	0.296003	0.43887	0.763156	-0.38995
0.6697	1791.55	1051.25	1301.23	191.09	568.4	480.16	1.186615	0.028684	0.091097	3.342903	1.741102
0.6696	721125.3	471889.1	376868.4	210079.2	805144.5	95198.8	0.643359	0.57538	0.684147	1.413771	0.499548
0.6682	18599.15	25894.76	23180.23	21713.28	22134	17527.05	0.491772	0.467854	0.592039	1.102646	0.140969
0.6682	32756.85	31312.44	55158.57	22431.23	79456.32	23333.11	0.154632	0.928842	0.948868	0.952142	-0.07075
0.6678	50859.53	46048.71	45775.78	20493.73	14406.6	28670.9	1.239235	0.013876	0.057827	2.244475	1.166378
0.666	13287.03	9702.39	17146.93	32774.95	33035.79	17074.05	1.0852	0.098958	0.210095	0.484243	-1.0462
0.6653	13577.23	9487.97	11144.21	29848.53	28186.48	31832.71	1.339798	0.000332	0.010018	0.380664	-1.39341
0.6651	43597.05	70991.59	41729.89	377.81	2561.92	3290.14	1.274935	0.032989	0.09996	25.09178	4.649143
0.6645	969.58	503.92	1606.74	2102.17	933.72	2818.52	0.779859	0.235792	0.377874	0.52614	-0.92648
0.6618	45148.57	37994.56	49709.45	26708.91	25522.83	51090.12	0.729917	0.363483	0.502353	1.285813	0.362681
0.6613	62.29	965.24	1993.65	821.24	5268.34	3137.54	0.75676	0.244655	0.388082	0.327424	-1.61077
0.6591	128135	70759.85	105479.4	50870.8	52709.26	64708.45	1.141015	0.104632	0.218122	1.808645	0.85491
0.6588	5476.51	11504.03	14461.09	7311.34	2077.55	7777.78	0.752536	0.221365	0.361669	1.831551	0.873066
0.6583	1862.18	1672.72	1545.21	4591.75	5675.39	4754.14	1.356551	0.006884	0.038918	0.338194	-1.56408
0.6578	17195.9	13926.51	12943.12	33552.45	32181.13	33126.63	1.34362	0.002553	0.023542	0.445736	-1.16574
0.6571	3952.28	6706.3	4683.93	1574.98	561.58	460.36	1.260908	0.022158	0.076778	5.907964	2.562661
0.6567	116534.2	52439.37	51387.35	56102.47	51509.96	56588.01	0.509673	0.476658	0.59964	1.342024	0.424411
0.6535	90275.79	70094.04	95431.2	41240.36	47329.39	43208.65	1.309717	0.028038	0.089735	1.941145	0.956908
0.6529	212546.7	217741.6	289348.8	372669.9	361493	325782.3	1.206514	0.02557	0.083741	0.678938	-0.55865
0.6516	27535.26	31474.27	155200.7	5688	14200	18889.84	1.026904	0.29729	0.43988	5.524038	2.465723
0.6507	53.98	1349.02	204.66	3019.75	2289.89	2708.83	1.078113	0.018864	0.069391	0.200495	-2.31836
0.6499	6617.34	20195.07	4589.92	5763.69	10203.66	8895.06	0.075067	0.704537	0.788872	1.263044	0.336905
0.6493	810.41	429.91	739.6	40.16	119.75	434.68	1.02344	0.05133	0.133581	3.329891	1.735475
0.649	30622.97	284112.7	271142.9	54386.91	34210.83	32274.07	0.866819	0.19998	0.337351	4.847107	2.277124
0.6488	1395.25	1408.7	1285.01	142.88	868.06	1756.79	0.656813	0.445107	0.572505	1.47737	0.563031

0.6461	819.68	247.07	67.22	8695.73	16371.18	275.77	0.923806	0.224449	0.364792	0.044745	-4.48211
0.6443	10990.98	5378.76	9146.72	8721.01	7953.82	7707.37	0.012063	0.841603	0.888484	1.04652	0.0656
0.6443	1904.26	1555.67	1574.79	770.03	1267.83	4934.33	0.014448	0.67196	0.761589	0.722115	-0.4697
0.6442	218944.7	186705.8	177747.5	120952.4	116510.2	149953.3	1.232592	0.017082	0.065143	1.50587	0.590597
0.6431	3934.39	2147.42	4296.41	985.29	4402.69	525.2	0.781024	0.361334	0.500654	1.7551	0.811553
0.6426	5829.71	904.06	5592.17	5270.54	4301.81	5446.37	0.493946	0.635247	0.73197	0.820705	-0.28506
0.6424	53861.87	53717.02	50213	57072.49	58594.45	65312.15	1.128439	0.074287	0.172335	0.871879	-0.1978
0.6411	273.82	1245.21	492.56	413.59	1088.75	119.37	0.326459	0.76765	0.836755	1.240413	0.31082
0.6404	67.41	9351.55	12097.23	5710.18	6222.61	6360.3	0.434189	0.795822	0.857176	1.176192	0.234124
0.6394	1189.99	675.21	346.46	414.66	1874.06	160.88	0.225539	0.901703	0.930522	0.902866	-0.14742
0.6378	66586.6	7211.38	74297.37	47779.5	37176.54	36575.46	0.177339	0.718473	0.800363	1.218576	0.285196
0.6364	20880.38	30477.77	27614.15	31806.51	32763.51	33457.54	0.970184	0.151756	0.278762	0.805613	-0.31184
0.6358	1659.91	7810.98	787.05	999.87	434.52	525.88	0.905488	0.33715	0.477732	5.232922	2.387617
0.6358	75453.11	70777.49	68875.87	5489.39	3581.65	3415.41	1.365289	0.000263	0.009471	17.22719	4.106616
0.6357	5394.76	5187.95	7740.3	1223.79	1393.26	3473.75	1.183813	0.020799	0.074132	3.008309	1.588953
0.6353	31946.66	27743.93	41942.2	5416.79	2633.57	6056.18	1.320667	0.015705	0.061951	7.204658	2.84893
0.6352	39604.69	29870.61	8468.93	55076.01	25606.71	8679.91	0.064115	0.829135	0.880434	0.872224	-0.19723
0.6343	6288.58	2240.73	7034.53	3679.85	4615.31	3300.24	0.335734	0.471213	0.594851	1.342243	0.424645
0.6339	6181.81	3516.77	7715.55	2164.72	4991.19	8827.54	0.268465	0.846769	0.891131	1.08951	0.12368
0.6319	1198.41	1762.97	2611.09	770.07	1697.48	1445.51	0.653998	0.334591	0.476161	1.42407	0.51002
0.6316	43292.58	47260.71	53477.53	35797.18	42380.31	47786.68	0.756873	0.258765	0.404341	1.143427	0.193364
0.6313	348972.5	348007.3	401757.9	172236.2	199975.4	178964.4	1.349138	0.003257	0.026835	1.993442	0.995262
0.6308	42164.46	29723.55	41553	4949.51	6950.11	4118	1.34781	0.012426	0.054133	7.082264	2.824211
0.6301	3755.31	364.39	264.37	28.87	203.48	158.75	0.94829	0.365758	0.504219	11.20959	3.486661
0.6292	862.38	373.42	1379.68	1353.61	1681.38	1608.95	0.921526	0.134315	0.256677	0.563203	-0.82827
0.6288	10126.57	4435.05	14632.55	7740.29	380.85	678.62	0.960737	0.151556	0.278512	3.31761	1.730144

0.6272	6069.73	4542.18	7099.66	1025.2	1727.4	3069.52	1.190441	0.015589	0.061718	3.042117	1.605076
0.6257	5298.45	5509.46	11008.42	12206.76	10345.36	5991.41	0.556896	0.440963	0.568038	0.764318	-0.38776
0.6253	60543.2	53976.73	58830.09	60977.34	59497.19	57853.23	0.496389	0.502474	0.621005	0.972087	-0.04084
0.6252	4501.22	3327.66	6530.78	8090.38	1093.68	1706.79	0.604193	0.669631	0.759543	1.318507	0.398905
0.6251	7865.42	8005.16	8355.63	7554.43	8418.77	7704.62	0.410359	0.588673	0.695326	1.02316	0.033032
0.625	192834.8	177063.4	200088.9	96099.15	91978.98	97035.88	1.36404	0.003574	0.028016	1.999155	0.99939
0.624	44978.21	36829.91	58607.18	32861.21	32310.89	42701.8	0.846978	0.227371	0.367895	1.301661	0.380354
0.6238	25241.18	7268.74	25425.14	42234.1	74840.8	58735.58	1.109168	0.031918	0.0978	0.329531	-1.60151
0.6234	13376.37	12927.92	22581.99	33736.42	21758.42	53546.75	1.060006	0.152923	0.280552	0.448327	-1.15738
0.62	9359.94	11804.49	9296.14	3357.87	3113.36	2035.13	1.325668	0.004598	0.032268	3.580917	1.840329
0.6198	288.68	246.37	86.25	665.12	185.7	216.76	0.557784	0.446542	0.573843	0.58197	-0.78098
0.6174	13001.24	10760.39	38990.69	1107.31	6582.2	14954.32	0.824955	0.277452	0.42144	2.771277	1.470551
0.6172	2322.13	1991.29	3345.89	10692.55	12574.81	6603.03	1.288734	0.04607	0.124725	0.256418	-1.96343
0.616	44853.55	11162.42	15585.1	31426.72	35635.96	32994.77	0.720925	0.464661	0.590169	0.7156	-0.48278
0.614	44060.54	30335.48	41753.23	27534.27	24571.75	27476.9	1.141695	0.096031	0.206308	1.459475	0.545449
0.6139	16585.57	11657.27	15825.11	82743.24	82876.1	64642.01	1.354224	0.006602	0.038167	0.191382	-2.38547
0.6118	111233.3	63465.96	112632.5	68433.98	58882.63	45441.12	1.013844	0.128178	0.249209	1.663207	0.733968
0.6118	1084.43	2552.11	2300	3170.94	2836.32	1874.82	0.630061	0.339629	0.480507	0.753169	-0.40895
0.6114	7023.46	11055.23	8879.4	31321.13	24022.47	29982.91	1.326608	0.004528	0.032107	0.31594	-1.66228
0.6113	219.69	156.19	27.72	1052.87	979.14	878.77	1.197418	0.00041	0.010631	0.138657	-2.85041
0.6113	6461.66	6251.93	5208.98	44620.03	35492.2	35771.56	1.365033	0.007534	0.040336	0.15466	-2.69283
0.6103	1901.79	8641.86	6840.83	1429.9	465.95	359.52	1.169069	0.125613	0.245648	7.708039	2.946364
0.6099	29866.7	32346.06	24651.58	3931.38	1307.59	2269.39	1.329543	0.003763	0.028827	11.56902	3.532194
0.6086	28895.3	25110.68	29956.7	27516.4	24395.93	22473.19	0.834363	0.199454	0.336984	1.12875	0.174726
0.6078	90998.16	84146.25	98277.08	2846.7	2085.1	6229.66	1.346504	0.000976	0.014975	24.49693	4.614529
0.6056	77363.95	68588.05	106652.8	28017.47	28976.21	27942.24	1.33329	0.039777	0.113325	2.974063	1.572435

0.6052	200792.9	105744	124172.8	84622.01	185394.7	189784.3	0.086364	0.840256	0.887873	0.936731	-0.09429
0.6048	349380.7	355341.1	419767.2	248990.7	291739.4	312534.7	1.154216	0.038353	0.111676	1.317866	0.398204
0.6028	52292.9	30550.7	42750.33	30418.34	36571.43	33654.73	0.710236	0.316071	0.458335	1.247897	0.319498
0.6017	62027.03	55890.86	63107.39	119344.7	112788.6	392521.8	1.088629	0.249788	0.394503	0.2898	-1.78687
0.6012	4144.19	5051.74	4488.54	62732.98	46175.59	48035.25	1.367027	0.011654	0.052158	0.087193	-3.51964
0.6011	66597.46	64215.77	80628.32	55874.79	35880.66	39093.11	1.168233	0.030373	0.094435	1.615926	0.692361
0.5981	7344.73	3491.87	5448.6	10748.49	1337.15	4006.21	0.309866	0.984459	0.988541	1.012015	0.017231
0.5976	356.07	134.45	154.26	711.87	281.87	173.23	0.615607	0.409381	0.541353	0.552525	-0.85589
0.5916	60467.31	63015.38	78369.85	56540.47	56153.66	50783.07	1.045766	0.138904	0.26352	1.234744	0.304212
0.5894	1285.12	971.9	1516.07	1342	60.99	391.69	0.823745	0.221795	0.362099	2.102375	1.07202
0.5869	2328.55	1863.27	2472	583.13	940.86	750.85	1.315019	0.005227	0.033956	2.929358	1.550584
0.5869	56133.01	123488.4	39983.07	19135.6	27424.74	20601.35	1.150468	0.184135	0.31927	3.269788	1.709197
0.5868	36785.17	33265.7	35057.43	10981.61	9035.95	10880.97	1.364157	0.00012	0.006443	3.401725	1.766266
0.5868	169388.9	229766	202154.2	105058.9	106899.5	166027	1.111478	0.049712	0.131093	1.590827	0.669777
0.581	34211.4	24444.27	33267.93	61679.08	61124.5	64809.78	1.314448	0.00464	0.032268	0.489963	-1.02926
0.5793	2669.36	4791.66	4192.64	92.16	4987.39	2753.66	0.610235	0.475988	0.599183	1.487725	0.573108
0.5785	7574.67	6689.2	9066	24115.57	24184.06	22210.31	1.354106	8.01E-05	0.00568	0.330873	-1.59565
0.5769	12179.04	10158.88	11562.58	22723.72	27080.58	2949.77	0.075375	0.48668	0.608061	0.642614	-0.63798
0.5762	116779.2	36865.57	33422.97	63797.16	78798.11	26289.66	0.036572	0.858547	0.900266	1.107664	0.14752
0.5743	7802.06	3304.71	7735.83	10966.56	5480.5	6430.99	0.4076	0.583261	0.691066	0.82361	-0.27997
0.5711	105073.9	91905.72	112362.5	333790.3	299422.8	290977	1.360935	0.00107	0.015658	0.334717	-1.57899
0.57	650.91	2075.45	471.94	340.91	698.82	524.28	0.658881	0.395782	0.531326	2.044936	1.032056
0.5669	107.32	6.57	138.75	10.25	46.08	14.9	0.518303	0.265376	0.411095	3.54682	1.826526
0.5649	26029.63	31952.56	33474.05	22825.45	25798.13	24032.45	1.09684	0.095817	0.20595	1.258756	0.331999
0.5647	25474.36	21614.05	28839.5	21848.9	29499.38	77590.83	0.603503	0.417935	0.548303	0.588866	-0.76399
0.5625	40757.21	39667.74	38384.67	29607.77	23636.92	22926.62	1.289696	0.014515	0.059459	1.559769	0.641332

0.5611	6478.82	12605.17	8408.73	4328.45	3547.35	2882.24	1.230654	0.084124	0.187762	2.555551	1.353634
0.5582	4323.59	3196.79	5590.48	363.45	358.54	1858.94	1.180651	0.017758	0.066787	5.079898	2.344799
0.5528	1842.38	1216.74	1112.01	3224.26	3446.38	133.26	0.110251	0.500247	0.619114	0.61305	-0.70592
0.5521	1998.45	210.97	197.9	86.8	1614.8	1002.93	0.102503	0.901116	0.930136	0.890107	-0.16795
0.5504	21057.89	28395.41	27479.08	304.42	607.8	720.79	1.358923	0.008208	0.042393	47.11078	5.557985
0.5504	1681.22	2868.96	2044.44	5778.61	4213.16	1623.32	0.670819	0.299525	0.442436	0.567763	-0.81664
0.5496	1659.32	1859.15	1992.61	948.71	25195.62	1484.1	0.375754	0.45379	0.58059	0.199471	-2.32575
0.5486	18949.75	15313.45	16065.98	14869.24	13549.24	13676.48	1.066779	0.118334	0.235802	1.195611	0.257748
0.5427	247533.3	198396.4	245449.5	158404.5	169532	167527.4	1.247831	0.049892	0.131232	1.395418	0.480697
0.5421	38.72	46.22	35.21	14.98	142.03	30.69	0.013085	0.630497	0.729015	0.640117	-0.64359
0.5399	22068.46	29453.7	22380.07	3679.59	5272.43	1775.76	1.303509	0.005948	0.036092	6.888865	2.784266
0.5399	3301.92	3425.02	2236.47	4573.75	4735.24	5463.63	1.193187	0.017174	0.065383	0.606758	-0.72081
0.5391	1843.87	2667.83	1900.43	1042.29	1124.03	1127.13	1.286872	0.058094	0.145702	1.946934	0.961204
0.5383	20221.6	23476.78	18692.78	8858.67	9545.33	11316.09	1.326592	0.006333	0.037461	2.099292	1.069903
0.532	91472.48	95106.93	85301.47	192539.8	198733.1	188490.1	1.367108	1.58E-05	0.003622	0.468952	-1.09249
0.5299	226.4	164.91	129.71	338.1	410.32	256.06	1.158516	0.047178	0.127089	0.518696	-0.94704
0.5232	3369.31	19620.98	13560.79	2111.63	5545.48	4281.64	0.85071	0.222273	0.362744	3.06155	1.614262
0.521	264	693.7	795.07	8744.06	7853.14	9354.18	1.331382	0.001034	0.015441	0.067541	-3.8881
0.5176	4870.84	3560	724.37	1539.42	1951.37	263.67	0.645364	0.277622	0.42144	2.438489	1.285988
0.5158	674.6	1758.64	655.28	763.64	2478.06	2035.83	0.659982	0.317738	0.46014	0.585221	-0.77295
0.511	401.18	1215.88	604.92	1402.23	920.54	560.05	0.460457	0.558518	0.672701	0.770766	-0.37563
0.5078	390.52	141.71	236.07	291.44	550.96	76.48	0.021992	0.767322	0.836608	0.836127	-0.25821
0.5078	2294.38	3053.85	3260.16	1544.34	788.71	1124.41	1.241582	0.011334	0.051196	2.489802	1.316031
0.5046	8929.61	9738.49	4286.91	7331.14	4408.38	2638.56	0.727636	0.263044	0.408452	1.596528	0.674938
0.9326	79546.9	84649.89	87664.75	106419.5	93024.72	73363.11	0.40186	0.546279	0.661269	0.923221	-0.11525
0.778	10173.52	6676.67	18466.76	25240.17	9932.15	10759.01	0.397621	0.595006	0.700414	0.768908	-0.37912

0.726	1787.21	3702.17	7116.66	25174.96	15549.07	5120.48	0.975212	0.189711	0.325059	0.274974	-1.86263
0.7172	6448.02	4442.52	9309.37	4169.53	3930.65	1287.44	0.954653	0.110564	0.226798	2.151761	1.105518
0.7127	50.04	383.85	245.8	272.3	216.31	258.17	0.409371	0.840087	0.887873	0.910161	-0.13581
0.7086	502928.7	615485.3	506153.5	466473.8	429241.7	509943.3	0.895596	0.183623	0.319115	1.155734	0.208809
1	16007.46	6809.93	12389.12	214980.3	243839.7	314470.4	1.352159	0.013562	0.056899	0.045528	-4.4571
0.9818	55079.65	43460.29	57457.94	30353.16	32791.13	30628.62	1.29216	0.037263	0.10938	1.663571	0.734283
0.9816	1916768	3286393	2424960	590696.4	513282.8	599865.5	1.340496	0.038062	0.111235	4.477004	2.162534
0.9756	9488.34	9968.33	9310.54	2265.52	1366.04	2675.71	1.328369	0.000452	0.011141	4.560961	2.189338
0.9524	74080.5	64970.39	90356.02	66405.88	90513.02	64195.71	0.18709	0.817911	0.872772	1.037502	0.053114
0.9524	30619	27035.25	27492.29	83896.05	74854.15	121963.5	1.328927	0.04493	0.122758	0.303322	-1.72108
0.947	14412.67	6958.97	9091.3	5126.13	2743.68	2055.38	1.179115	0.074334	0.172351	3.069255	1.617889
0.9451	586901.3	7788.2	542843.3	2227.7	106743	3433.25	0.795169	0.204624	0.343192	10.12004	3.339143
0.9275	406050.9	416904.6	420713.3	271276.5	274304.2	275989.4	1.370776	0.000371	0.010037	1.513771	0.598147
0.8691	56163.54	69772.8	58820.52	65979.92	53703.14	78394.62	0.324976	0.625611	0.725276	0.93275	-0.10044
0.8436	230890.1	242759.4	278217.3	160327.5	157742	160929.5	1.332244	0.02313	0.078955	1.569663	0.650454
0.8352	91.7	482.24	945.54	8785.13	8866.29	9069.64	1.261887	0.000269	0.009571	0.056865	-4.13633
0.8346	2936210	4030207	2281090	2766433	2958147	2747969	0.246126	0.66402	0.755143	1.091467	0.126268
0.8111	959662.6	1089571	352091.7	2070023	2269926	1850741	1.132373	0.015604	0.06172	0.387893	-1.36627
0.791	227533	205353.5	182784.2	171493.6	178606.4	184564.1	0.983912	0.163826	0.29436	1.15151	0.203526
0.7871	137269.8	95179.87	85821.77	82479.32	92513.81	82291.79	0.750663	0.326692	0.468901	1.237039	0.306891
0.7806	204048.8	265649.3	249641.3	118848.2	92031.78	133891.2	1.28878	0.00735	0.039748	2.086426	1.061034
0.7705	10944.71	4854.92	7103.35	1942.65	2266.85	2307.07	1.27434	0.091005	0.199142	3.514576	1.813351
0.75	9723.31	13776.15	4635.08	4464.92	4404.36	2443.04	1.027433	0.1616	0.291322	2.487071	1.314448
0.75	6542.15	2380.06	6680.68	3958.01	2230.17	3592.35	0.62973	0.302111	0.4449	1.595301	0.673829
0.7377	5287.34	3339.77	3147.16	2146.53	471.3	3606.8	0.800502	0.183735	0.319115	1.891561	0.919578
0.7377	37.84	205.08	13053.91	518.54	1239.05	49.15	0.135439	0.468252	0.592108	7.35957	2.879622
0.7353	24151.47	16148.7	10351	12113	12155.96	10815.4	0.727194	0.323712	0.46585	1.443697	0.529768

0.7316	825.38	5927.08	5738.47	1759.36	2399.3	2376.02	0.346514	0.356303	0.497078	1.911483	0.934692
0.7279	26071.73	11983.66	24291.26	11201.27	25714.26	12993.55	0.430587	0.550258	0.665347	1.249205	0.32101
0.7185	2442.4	4591.13	2237.91	5675.16	3459.84	2906.86	0.566018	0.46106	0.587306	0.769934	-0.37719
0.7168	23672.9	29748.06	42667.79	17640.08	8961.27	11951.82	1.196191	0.057872	0.145481	2.49237	1.317518
0.7143	1006.4	4691.82	6395.29	16151.23	22581.74	16833.73	1.147036	0.005904	0.036092	0.21764	-2.19999
0.7081	96.85	2693.42	57.35	343.12	2200.47	1861.16	0.674908	0.648609	0.74246	0.646488	-0.6293
0.7024	210.62	343	396.36	397.68	538.28	220.68	0.340905	0.562615	0.674302	0.821327	-0.28397
0.6968	722.36	371.31	615.07	495.41	443.67	105.5	0.714848	0.24144	0.384803	1.635815	0.71001
0.6865	539.39	951.06	308.61	791.96	1196.62	1060.67	0.912191	0.147158	0.27291	0.590001	-0.76121
0.6858	91970.64	127871.7	79216.54	97438.3	74664.16	60912.53	0.733029	0.295128	0.43802	1.283432	0.360007
0.6858	23.82	604.47	106	59.04	563.1	735.8	0.483537	0.488193	0.608879	0.540738	-0.887
0.6783	47735.08	37105.09	35085.23	39274.71	36625.27	61228.61	0.409444	0.558809	0.672701	0.874547	-0.19339
0.675	1823.42	1910.01	1421.93	2852.87	2769.28	4058.55	1.232597	0.055095	0.140178	0.53254	-0.90904
0.6658	33270.43	17704.18	12806.06	65683.96	9699.97	5509.54	0.216674	0.801646	0.861532	0.788453	-0.3429
0.6647	8763.97	2703.01	8445.41	12772.18	13866.52	6586.48	0.747442	0.214832	0.354448	0.599316	-0.73861
0.6594	44.53	61.54	169.5	121.82	100.98	35.99	0.021807	0.911647	0.937454	1.06484	0.090637
0.6592	50254.09	46836.63	51467.48	75533.29	42698.29	47936.84	0.307033	0.623743	0.724073	0.894022	-0.16162
0.6549	30491.42	43409.6	56363.88	20581.76	12116.36	15175.71	1.241885	0.054826	0.139921	2.721004	1.444139
0.641	77338.66	9995.21	94402.45	134956.9	158448.4	134357.7	0.889311	0.07523	0.173967	0.424853	-1.23497
0.6409	29889.5	24511.12	26776.11	1170.5	3164.91	9793.26	1.183005	0.00385	0.029026	5.745532	2.522441
0.6365	7914.19	5056.55	11760.13	1707.83	660.72	960.09	1.290681	0.063357	0.154777	7.429722	2.893308
0.6346	2058.65	1951.37	2195.87	576.07	1439.51	1630.83	0.943448	0.113249	0.229605	1.701918	0.767161
0.6298	1673.68	327.75	300.32	887.28	1366.07	703.21	0.578047	0.691461	0.779033	0.778523	-0.36119
0.6126	11975.8	11629.32	13266.71	13551.74	9751.67	14713.88	0.087874	0.827805	0.880098	0.96987	-0.04414
0.6092	486.62	1748.19	2641.86	819.77	708.85	501.54	0.743822	0.267042	0.412926	2.402111	1.264303
0.6024	1510.21	1870.41	1550.38	219.71	1126.67	447.96	1.081926	0.045825	0.1243	2.748086	1.458427
0.5852	2988.37	1529.57	17935.29	2898.64	1203.23	2283.35	0.613384	0.4145	0.546566	3.516438	1.814115

0.5742	6213.07	5178.33	6506.35	6158.7	2686.76	4950.27	0.7127	0.311787	0.454542	1.29734	0.375556
0.5619	862.74	1466.4	1052.8	4844.81	11198.55	10595	1.31234	0.060891	0.150298	0.126958	-2.97758
0.5597	9144.65	7095.51	5448.85	2508.09	4079.69	3118.1	1.221194	0.048336	0.129104	2.234626	1.160033
0.5596	13055.36	13924.89	31604.58	7026.4	10041.94	11527.49	0.979307	0.23667	0.379002	2.048719	1.034722
0.5533	2112.58	8370.55	20212.25	10904.39	11584.9	9212.8	0.390267	0.95555	0.967853	0.968245	-0.04656
0.5421	1297.14	839.86	2241.85	2175.74	2528.04	1035.53	0.459624	0.499449	0.618318	0.762958	-0.39033
0.5389	2676.03	3508.18	3103.41	4541.48	1283.06	5618.34	0.056689	0.638999	0.734933	0.811651	-0.30107
0.5314	4069.29	4180.02	4743.14	1614.18	3707.93	3322.5	0.90653	0.14307	0.267818	1.502954	0.587801
0.5279	1079.89	3900.99	1242.85	2545.11	2623.31	2916.78	0.648353	0.568265	0.67902	0.769768	-0.3775
0.521	11666.9	2940.76	1860.72	28655.45	28219.24	6698.95	0.976166	0.150201	0.276803	0.259044	-1.94873
0.9342	143346.3	109445.9	105247.5	224739.7	208379.7	173577.2	1.244695	0.014098	0.058527	0.590146	-0.76086
0.7392	225.17	1136.81	19.33	561.73	361.86	410.71	0.505636	0.96802	0.976696	1.035232	0.049954
0.7133	37782.07	39598.02	44051.71	46632.35	64909.46	28361.79	0.242375	0.620506	0.721277	0.867968	-0.20429
0.7013	25839.05	24904.85	25862.03	36133.61	36370.46	36050	1.36929	0.000346	0.010018	0.705694	-0.50289
0.6987	16721.19	19901.4	18330.71	7123.09	8326.27	6973.98	1.354089	0.002248	0.021923	2.450719	1.293205
0.6527	3879.27	9786.07	8780.54	7524.38	6592.16	8396.49	0.167342	0.991524	0.993446	0.997017	-0.00431
0.6493	26664.78	21762.37	24301.2	131230.9	126267.2	105600.9	1.364533	0.005295	0.034293	0.200299	-2.31977
0.5121	7964.34	8566.01	3561.35	24141.94	22031.76	39695.85	1.252332	0.049863	0.131232	0.233979	-2.09555
0.9555	349980.1	323722.8	335936.1	2510817	2121500	2274491	1.37075	0.003188	0.02649	0.14618	-2.77418
0.934	24672.59	21612.27	22614.92	61660.37	36154.33	36933.03	1.198167	0.118335	0.235802	0.511324	-0.96769
0.9328	223377.3	274416.8	336312.9	477360	316839.9	383288.8	0.983839	0.122606	0.242047	0.708378	-0.49741
0.929	14249.01	23073.63	12995.78	4913.17	5863.22	5967.27	1.294727	0.070187	0.165377	3.005222	1.587472
0.9222	27602.26	32448.43	53634.33	31084.59	52278.79	44003.61	0.34911	0.676285	0.765278	0.892578	-0.16395
0.9185	16951.12	19881.07	15214.95	18746.7	18323.5	18289.74	0.546859	0.503025	0.621304	0.940159	-0.08902
0.9107	93023.08	94739.3	111722.8	215744	152720.3	129850.4	1.146895	0.116379	0.233919	0.600996	-0.73457
0.9068	97773.99	121756.8	84019.34	41368.86	277517.3	43821.71	0.243382	0.825573	0.879011	0.8369	-0.25687

0.8996	18564.91	19315.74	17294.03	16390.05	17020.39	19832.64	0.375241	0.630503	0.729015	1.036279	0.051412
0.8983	44141.18	45612.19	41877.14	49021.72	45657.35	44352.28	0.7885	0.238649	0.380979	0.946769	-0.07892
0.8929	44315.94	53001.96	54993.08	207891.9	82200.56	20694.71	0.330636	0.438855	0.566135	0.490081	-1.02891
0.892	14680.72	9599.4	24077.92	140486	138734.2	126137.7	1.333495	4.47E-05	0.004503	0.119297	-3.06737
0.8857	37868.7	129718	52782.89	39459.69	71150.82	54218.43	0.29341	0.589252	0.695519	1.336959	0.418956
0.8824	17183.81	28073.82	32639.79	27539.75	24397.81	22847.01	0.030671	0.845758	0.890712	1.041624	0.058835
0.8638	169195	147703.5	169814.3	200122.3	179978.1	189029.8	1.12394	0.044495	0.122213	0.855187	-0.22569
0.8612	19767.24	15246.87	16559.89	5163.63	4521.64	5569.58	1.355371	0.009295	0.045573	3.380826	1.757376
0.8546	234450.5	193697.4	207923.6	975176.9	824880.8	851707.1	1.365253	0.002989	0.025612	0.239867	-2.05969
0.8515	101296.2	80648.75	90709.55	274107	296589.5	299173.9	1.362831	6.5E-05	0.005153	0.313443	-1.67373
0.8474	30909.98	30908.73	36297.76	22568.96	24347.67	587.34	0.735671	0.151847	0.278813	2.065437	1.046447
0.8469	295.64	489.24	281.66	583.26	135.55	80.42	0.615495	0.645387	0.740433	1.334459	0.416255
0.8417	48790.23	38244.03	43485.87	228312.9	192429.6	172949.8	1.35927	0.008987	0.04482	0.219845	-2.18544
0.8377	17736.26	9240.65	14023.52	29999.73	24509.74	30752.89	1.21083	0.010539	0.049344	0.480874	-1.05627
0.8376	23517402	17984792	20038013	14130257	12381021	13226989	1.274243	0.036268	0.107252	1.548638	0.631
0.8356	42864.56	35177.85	27631.62	47344.69	65895.97	56313.46	1.145708	0.039084	0.112529	0.623247	-0.68212
0.8242	570.13	3206.9	918	1332.89	1455.28	1120.97	0.113129	0.782166	0.84665	1.201039	0.264283
0.8219	44870.15	52434.54	41318.25	8464.67	16945.17	3065.08	1.199468	0.002481	0.023205	4.868247	2.283402
0.8219	3367.84	1835.96	1217.72	3392.81	3466.62	3088.16	0.908858	0.204236	0.342806	0.645535	-0.63143
0.8211	11768.82	45557.19	4225.31	1235.4	2900.54	6316.06	0.928444	0.311575	0.454384	5.888951	2.558011
0.8154	8472.6	6653.69	8135.31	231.66	693.39	3273.27	1.132501	0.008272	0.042671	5.540692	2.470066
0.8127	22958.16	24905.41	22298.89	59253.05	46051.97	43515.42	1.325135	0.030253	0.094144	0.471457	-1.0848
0.8111	10304.11	5764.97	3982.19	22410	11798.01	19376.15	1.14662	0.050001	0.131297	0.374201	-1.41811
0.8082	15350.35	22398.5	22811.67	25060.45	21925.37	19249.74	0.43145	0.559821	0.673086	0.91432	-0.12923
0.8036	9982.42	7314.74	8802.34	18537.06	22387.65	21759.62	1.333876	0.001965	0.020396	0.416364	-1.26408
0.8011	7846.78	6928	5846.13	575.92	4830.4	4197.12	0.853709	0.092796	0.201846	2.147242	1.102485
0.801	12267.04	11988.34	17936.03	5075.93	3063.09	3604.56	1.312489	0.026077	0.084829	3.592721	1.845077

0.8008	291.81	264.59	463.87	724.55	924.43	611.24	1.218066	0.025342	0.083369	0.451403	-1.14751
0.7968	228993.1	205630.6	225173.8	120703.7	111236.6	105932.8	1.357646	0.000679	0.013514	1.952797	0.965542
0.7958	214030.6	171143.2	203578.9	101546	138315.7	91895.52	1.237401	0.011254	0.051174	1.77465	0.827534
0.7748	161987.8	144956.9	138220.5	184820	185087.1	155333.7	1.015411	0.099912	0.211304	0.847545	-0.23864
0.7742	40633.32	42553.77	47648.61	231488.4	210210	200175	1.368384	0.001953	0.020396	0.203834	-2.29453
0.7719	904405.9	732726.9	774138.5	167297.4	433055.7	266826.1	1.219758	0.007777	0.040992	2.780592	1.475392
0.7691	112423	127517.5	89993.08	141133	136011.4	131014.3	1.003021	0.131157	0.253182	0.808346	-0.30695
0.7628	43289.99	48342.73	48241.04	17261.3	13912.87	3244.41	1.152249	0.007237	0.039577	4.063903	2.022866
0.761	4338.26	3932.87	3961.41	3318.94	3658.69	3274.85	1.211343	0.020986	0.074519	1.19313	0.254751
0.7598	17045.59	27265.3	13420.38	5968.16	5910.64	5494.74	1.290447	0.083066	0.186449	3.322942	1.732461
0.7597	194255.8	134549.7	175403.8	550819.4	140632.2	250120	0.692444	0.356258	0.497078	0.535497	-0.90105
0.7526	14219.83	20826.18	23821.41	237.19	21241.15	64.79	0.942581	0.211714	0.35082	2.732538	1.450242
0.7524	15107.22	13643.4	11363.82	226.84	614	4217.96	1.164149	0.002411	0.0228	7.929635	2.987255
0.7524	56378.23	41560.89	50977.88	154215.2	104897.1	103524.6	1.291364	0.04317	0.119782	0.41065	-1.28402
0.7514	1240836	1286505	1115133	1726092	1696888	1749102	1.332714	0.006282	0.037309	0.704257	-0.50583
0.7481	431295.5	314314.8	395069.1	680438.6	825624.2	613044.1	1.2685	0.018317	0.068093	0.538283	-0.89356
0.7459	41677.43	48839.91	51094.14	14991.77	14089.8	14446.44	1.364986	0.007071	0.039217	3.253341	1.701922
0.7444	54643.84	43224.56	53812.65	40652.32	47640.73	40402.76	0.900949	0.166808	0.297632	1.178601	0.237076
0.7435	25223.61	16930	29883.75	43601.03	1402.6	2456.12	0.778514	0.620202	0.721277	1.517862	0.602041
0.7415	42120.37	40749.17	41345.16	64212.18	64644.74	61126.67	1.366811	0.000943	0.014975	0.653818	-0.61304
0.7408	64044.95	64481.66	61015.57	79996.4	71507.07	75438.87	1.270197	0.02256	0.077737	0.8352	-0.25981
0.7402	29679.19	21852.31	23257.37	33991.61	33910.91	31943.77	1.15542	0.064888	0.157511	0.74904	-0.41689
0.7321	9211.42	7658.01	10893.25	3419.7	1727.83	4374.94	1.216566	0.008117	0.042173	2.915491	1.543739
0.728	11368.3	31050.36	39558.58	20904.42	9853.59	3360.02	0.836529	0.193061	0.329246	2.402754	1.264689
0.725	106696.7	86533.02	86084.68	31508.13	20518.78	19199.43	1.333347	0.002385	0.022717	3.921504	1.971407
0.7236	110346.6	81548.54	108840.7	148937.8	141848.8	82184.43	0.556965	0.380803	0.517754	0.806325	-0.31057

0.723	12386.2	37281.88	18518.25	26431.68	2354.48	704.27	0.85183	0.313872	0.45636	2.312151	1.209236
0.7159	170974.9	116465.4	163266.8	46529.16	59322.69	58823.81	1.318428	0.024968	0.082765	2.736938	1.452563
0.7106	41193.23	34384.58	43688.15	2554.84	1044.87	912.37	1.347651	0.004111	0.03002	26.43259	4.724246
0.7081	32086.89	36630.4	30816.81	29954.04	30230.53	25376.51	0.962367	0.121124	0.239774	1.16331	0.218236
0.7039	30394.86	25571.49	19027.73	63363.7	53551.77	57938.82	1.301611	0.001706	0.019081	0.428895	-1.2213
0.7037	163683.3	225943.8	145161.3	133162.7	152178.1	121554.1	0.907362	0.21652	0.356356	1.314316	0.394312
0.9853	393463.8	396874	337866.2	1580699	1425554	1386914	1.366617	0.001338	0.016618	0.256809	-1.96123
0.9778	721265.6	1400506	617847.3	861087.7	962557.8	654286.1	0.115827	0.764496	0.834361	1.105607	0.144839
0.9777	255873.3	317938.7	233346.7	415710.3	406268.9	319257	1.116008	0.050869	0.132936	0.707267	-0.49967
0.9768	208233.4	162535.3	204629.1	146159	103148.4	80536.24	1.148615	0.030784	0.095236	1.744456	0.802778
0.9724	2006203	2987943	1859925	2387001	1884149	2427530	0.028226	0.904165	0.93218	1.023197	0.033084
0.9711	850773.5	737032.4	639805.4	20736.79	5229.93	14624.41	1.345495	0.006684	0.038427	54.87926	5.778189
0.9703	39492.27	33536.58	43595.22	51038.29	56575.48	35793.27	0.716441	0.288877	0.431386	0.813238	-0.29825
0.9692	16640.09	39194.8	19156.98	23733.82	15481.89	18076.66	0.464577	0.502243	0.620987	1.308933	0.388391
0.969	4623892	4150211	4366553	507116.6	451347.7	418164.1	1.37078	0.00086	0.01464	9.545536	3.254826
0.9685	1573	1624.53	8277.79	1381.4	4688.27	2363.76	0.113839	0.707219	0.791062	1.360694	0.444343
0.9684	1320.85	918.54	133.1	14117.28	13162.88	15219.6	1.258214	0.000186	0.008277	0.055824	-4.16298
0.9684	402364.6	325968.9	377007.4	31004.33	27172.39	26035.97	1.370244	0.004189	0.030434	13.12559	3.71431
0.9604	3171196	2619869	3074204	4649220	3898055	3915176	1.234102	0.020552	0.073492	0.711358	-0.49135
0.9596	418660.2	355203.8	397260.5	155914.9	138903.8	133198.5	1.360834	0.002459	0.023047	2.736162	1.452154
0.9594	83230.41	94196.07	83160.44	43213.58	41774.82	38960.56	1.360019	0.003203	0.026541	2.102373	1.072019
0.9545	15487.16	12343.27	13389.07	2580.27	5097.85	2603.79	1.304781	0.001215	0.016008	4.008934	2.003219
0.954	1067056	861391.3	1034507	1443141	1255451	1287679	1.212461	0.017023	0.065069	0.74329	-0.428
0.9517	697239.8	675165.3	631051.7	204990.5	192629.6	178815.8	1.368684	0.000468	0.011338	3.475593	1.797259
0.9513	707.57	614.79	291.49	169.25	231.41	744.83	0.575802	0.524728	0.63998	1.408873	0.494542
0.9513	84579.72	109263.7	78592.33	135834.8	136227.6	110503.5	1.122329	0.044741	0.122599	0.712128	-0.48979

0.9491	40409.97	40680.11	40249.75	311164.2	202603.1	266808.7	1.360304	0.019951	0.071932	0.155449	-2.68549
0.9438	89816.32	83584.56	91900.16	123087.7	102628.7	104715.4	1.180638	0.064318	0.156539	0.802892	-0.31672
0.9412	605.71	234.27	866.73	427.33	107.44	171.06	0.868147	0.205423	0.344003	2.418019	1.273825
0.9408	58218.33	95771.93	6309.9	10013.76	56643.43	41557.36	0.090285	0.595256	0.700518	1.481318	0.566881
0.9346	51193.46	38665	48235.88	111560.9	117136.1	106838.4	1.34627	0.00023	0.009171	0.411564	-1.28081
0.9337	106634.7	159005.3	99272.11	118625.8	108666	100219.6	0.376914	0.580823	0.689302	1.114197	0.156004
0.9285	19703.25	16592.77	16337.94	58951.09	41730.83	39521.46	1.31944	0.038022	0.111235	0.375411	-1.41346
0.9282	315938.8	332875.3	541.13	32026.61	23862.46	24342.78	0.117941	0.221204	0.361637	8.093483	3.016761
0.928	2340275	4129085	791324.5	5703430	1301496	4985358	0.497809	0.4041	0.537854	0.605547	-0.72369
0.9275	158722.1	292120.7	349662.8	237796.5	330711.7	300936.7	0.324605	0.739706	0.816568	0.920709	-0.11918
0.9271	130517.3	99778.36	111417.6	51093.97	59352.71	52899.25	1.333091	0.016285	0.063099	2.091961	1.064856
0.9262	46592.33	118465.7	62994.14	4216.46	2622.91	2965.51	1.346334	0.078846	0.179768	23.25905	4.53972
0.9211	2484.02	227.16	143.84	611.26	842.44	650.29	0.354061	0.775447	0.842726	1.356955	0.440373
0.9196	89250.39	157530.6	107541.1	90830.67	146275.8	93134.14	0.198978	0.783429	0.847597	1.072921	0.101544
0.9179	15111.19	11910.57	16643.73	6798.89	6426	5801.01	1.326909	0.023685	0.080408	2.295055	1.198529
0.9163	4823156	5165253	3456886	7652098	7390873	7410548	1.242064	0.026384	0.085697	0.598806	-0.73984
0.9159	20257.97	41839.21	78529.59	8019.57	5828.7	5917.48	1.255972	0.141158	0.265554	7.114669	2.830797
0.9124	5103471	11861197	4316467	6566429	5773845	4373950	0.302862	0.594736	0.700285	1.273235	0.348499
0.9087	59764.48	63363.86	59353.51	33224.99	25888.06	27061.07	1.343694	0.00092	0.014857	2.117595	1.082426
0.906	233199.6	149017.9	215837.4	155698.3	146413.8	154032	0.922348	0.205338	0.343992	1.311109	0.390788
0.9055	17213.96	15980.78	15059.69	7214.33	6858.02	5254.58	1.338408	0.000375	0.010037	2.496746	1.320049
0.8963	5208.05	3274.99	3919.55	2735.63	2387.02	3554.2	0.964045	0.150248	0.276803	1.429389	0.515398
0.8944	288140.9	308411.7	291963.8	319883.5	344741.9	455550	0.974071	0.203091	0.34152	0.793194	-0.33425
0.893	394.43	564.37	6017.74	4453.65	3336.94	562.88	0.37418	0.845316	0.890607	0.835167	-0.25986
0.8909	313835.3	283107.8	369759.1	275582.2	230531	251452.9	1.085529	0.092494	0.201389	1.276063	0.3517
0.8901	4389.85	55.81	107.58	1062.31	771.68	759.68	0.496025	0.694121	0.780416	1.75552	0.811899
0.8894	19279.3	3118.08	17241.2	16644.45	15763.7	1444.93	0.228198	0.79875	0.859267	1.1709	0.227618

0.8862	37967.05	20988.54	69668.39	47393.77	77324.98	99844.66	0.835671	0.199952	0.337351	0.572774	-0.80396
0.883	20205.28	13983.99	18827.54	1026.95	1202.29	842.34	1.365138	0.012402	0.054133	17.26044	4.109397
0.8795	124.8	2085.56	220.21	688.96	318.57	271.31	0.009164	0.611364	0.714459	1.900605	0.926459
0.8789	46968.06	40551.33	43140.24	69968.16	54946.11	57709.22	1.220526	0.048951	0.129949	0.715459	-0.48306
0.8781	8053.3	6059.23	8358.46	3042.46	3465.48	3255.59	1.32793	0.025055	0.082801	2.301523	1.202589
0.8765	132034.9	105555.9	118868.2	92241.68	90174.07	56163.18	1.059219	0.05726	0.144358	1.494093	0.57927
0.8688	60340.48	59628.89	59123.81	105521.8	91064.44	86784.29	1.328594	0.025204	0.083162	0.632011	-0.66198
0.8537	39282.63	105592.2	49510.26	31318.99	32083.16	36707.1	0.9432	0.266384	0.412357	1.94173	0.957342
0.8519	1835512	3519536	1799268	769947.6	1772117	1200347	0.97036	0.173009	0.30644	1.911686	0.934846
0.841	296193	221784.4	278268.7	162382.7	181423.9	163064.9	1.262586	0.041675	0.117292	1.570904	0.651595
0.8358	1361942	1814650	1249983	544673.3	550852.5	482237.4	1.336119	0.029811	0.09327	2.805601	1.48831
0.8358	124587.7	109790.8	127168.7	140191.4	109914.8	119371.8	0.14834	0.815342	0.87067	0.978535	-0.0313
0.8293	3447.55	7857.45	6386.62	19304.16	5023.05	3901.36	0.312629	0.556361	0.670736	0.626727	-0.67409
0.8242	11288.05	13932.78	9052.82	9537.73	11217.64	8556.5	0.613085	0.377475	0.514675	1.169275	0.225615
0.8214	38412.48	24692.61	22288.69	22085.88	19353.87	3980.31	0.809959	0.153229	0.280995	1.88009	0.910801
0.8204	1977001	3462194	1711914	1259350	1276274	1402159	1.076071	0.187348	0.322405	1.816024	0.860783
0.8195	17484.39	3360.25	8330.31	12448.17	7117.51	5689.91	0.01259	0.796109	0.857273	1.155188	0.208127
0.8173	462.01	820.03	1049.8	3647.24	3344.36	3182.7	1.306074	0.000369	0.010037	0.229189	-2.12539
0.8171	23468.18	16579.14	21914.66	12259.13	11252.74	9621.82	1.269617	0.031949	0.0978	1.87006	0.903084
0.8169	274361.3	229339.1	233609.1	55937.55	73812.95	46051.69	1.345022	0.001145	0.015971	4.193972	2.068317
0.8127	482319.7	379104	444668.2	668840.8	690580	606437.1	1.278224	0.005488	0.034788	0.664388	-0.5899
0.8089	633.96	650.47	389.98	356.73	95522.06	322.23	0.480148	0.425312	0.553083	0.017405	-5.84433
0.8061	20733.12	19635.1	37121.73	1410.38	1823.64	16302.52	1.06218	0.062399	0.15298	3.966411	1.987834
0.8018	190732.5	172230	180134.7	217657.1	202713.3	166176.4	0.525979	0.449429	0.576873	0.925923	-0.11104
0.8009	538996.5	465923.2	477606.9	2342596	2293606	2182015	1.370077	8.12E-05	0.00568	0.217436	-2.20134
0.7977	8271.51	20071.03	6559.37	6911.35	4474.68	5075.4	0.910291	0.283355	0.426648	2.120223	1.084216

0.7965	497040.9	621166	502939.9	111995	71582.49	103637.4	1.35157	0.004838	0.032744	5.644369	2.496812
0.796	270.39	345.32	176.66	4346.15	6787.25	6739.45	1.356711	0.019143	0.070181	0.044334	-4.49545
0.7948	10505.33	13958.74	9807.8	49769.87	46560.12	43373.75	1.355156	0.000206	0.008701	0.245318	-2.02727
0.7911	120816.2	75189.79	116110.6	55674.76	151299	47373.49	0.522349	0.636376	0.73269	1.227128	0.295286
0.7901	6371.24	6708.41	1551.74	6149.78	1675.51	4491.64	0.131578	0.734922	0.813284	1.187909	0.248424
0.7849	55370.34	45185.9	58036.11	46625.21	43755.99	42282	0.998871	0.148164	0.273897	1.195451	0.257555
0.7817	4878.57	18978.23	9433.3	83163.15	74637.48	66403.78	1.279945	0.000634	0.013098	0.148481	-2.75165
0.7814	971233.4	968811.6	961521.3	751062.2	680837.1	682029.1	1.345204	0.007017	0.039121	1.372594	0.456905
0.7778	56767.01	48843.75	63519.34	30737	25301.07	20363.67	1.295734	0.005556	0.034847	2.213694	1.146456
0.7778	5256.34	5590.92	6551.93	232.28	2699.02	738.6	1.120117	0.012459	0.05415	4.741053	2.245207
0.7745	221946.8	147350.1	142753.3	116286.2	111004.5	96151.99	1.127534	0.127371	0.248082	1.583125	0.662775
0.7724	27085.09	11711.71	18863.32	6194.33	6027.77	6307.43	1.251384	0.098976	0.210095	3.111796	1.637748
0.7712	2189.78	2552.98	267.42	3461.15	4139.93	3756.47	0.870859	0.087099	0.192794	0.441132	-1.18072
0.7697	10439.16	9567.38	8618.84	6635.67	5770.53	5939.62	1.309377	0.010538	0.049344	1.560322	0.641843
0.7678	10047.42	8907.66	11502.02	31460.46	21470.55	33002.52	1.314104	0.031692	0.097286	0.354426	-1.49644
0.7671	29894.46	73669.39	62392.05	14983.26	7960.12	9591.3	1.266727	0.073562	0.171109	5.100892	2.35075
0.766	54541.32	47039.75	45345.92	41129.33	38250.7	34655.09	1.17978	0.039011	0.112432	1.288436	0.365621
0.7655	171152.3	430322.7	229207.8	256199.6	269295.1	218627.8	0.09245	0.750675	0.824853	1.116325	0.158757
0.7642	11366.67	14611.97	6690.75	17156.32	14412.06	9270.87	0.52129	0.450493	0.577733	0.799951	-0.32202
0.7631	5850.36	4073.58	2031.06	1277.03	966.33	1277.06	1.193222	0.124416	0.243894	3.395902	1.763795
0.7628	112036.7	129541.3	105672.5	40167.87	37573.62	37686.97	1.363587	0.007747	0.040883	3.008361	1.588978
0.7615	13049.56	23001.3	14516	13734.56	14950.02	12414.31	0.605059	0.41736	0.548303	1.23037	0.299093
0.7602	557399.5	451781.3	515483	702641.8	636038	614215.4	1.18338	0.025406	0.083469	0.78072	-0.35712
0.7592	260.38	409.72	3863.83	827.61	6865.91	1239.2	0.560259	0.561531	0.673575	0.507564	-0.97834
0.7586	1016348	1463840	1423312	505025.7	1621672	1298819	0.399587	0.691865	0.779085	1.139536	0.188447
0.7574	3545.91	5314.61	3375.95	246.05	182.55	61.12	1.326749	0.023439	0.079698	24.98667	4.643087

0.7573	3577970	6066321	3667567	2227656	2483111	1627200	1.183569	0.093061	0.202219	2.100336	1.07062
0.7555	68377.8	67792.78	186470.7	486011.2	441599	107578.6	0.930968	0.176532	0.310408	0.311674	-1.68189
0.7538	304484.2	298269.2	243557.4	348459.3	340713.1	381643.5	1.145308	0.039412	0.112856	0.790342	-0.33945
0.7517	34921.59	17440.83	28975.48	84155.09	119702.9	69533.97	1.263084	0.03855	0.112011	0.297514	-1.74897
0.7504	19833.46	18251.92	16298.56	17132.63	5882.77	8281.48	0.979917	0.144454	0.269628	1.737679	0.797162
0.7494	69067.49	55203.54	60806.32	53598.41	48968.14	55505.26	0.986734	0.141214	0.265554	1.170843	0.227548
0.7455	525.77	429.47	4497.51	5742.1	7554.85	7219.02	1.07559	0.048594	0.129474	0.265781	-1.91169
0.7453	217251.8	1516987	696820.2	536968.9	358957.2	229281	0.560303	0.370335	0.507798	2.160544	1.111394
0.7414	3029.76	3112.03	8968.88	4513.64	5099.17	7440.18	0.397491	0.785268	0.848954	0.886101	-0.17446
0.741	5198.63	4700.96	7641.54	337.11	11604.39	2393.73	0.595678	0.79051	0.852296	1.223638	0.291177
0.7405	5804.95	4155.73	7159.56	9455.78	8415.08	7717.48	1.071665	0.062179	0.152646	0.669064	-0.57978
0.7404	11910.28	12936.75	14277.87	5215.99	6630.42	4553.32	1.323343	0.001248	0.016093	2.385704	1.254415
0.7389	337564.2	1130793	943710.9	146206.6	108127	91190.24	1.259243	0.101866	0.213787	6.980901	2.803413
0.7378	3365.54	25432.44	5770.27	598.23	1145.58	6508.57	0.882155	0.335786	0.477238	4.188882	2.066565
0.7373	37365.8	26697.48	29301.99	17299.16	11454.26	11546.37	1.275775	0.014694	0.059716	2.316768	1.212114
0.7336	366.26	795.59	1209.78	1266.24	1583.12	1016.92	0.846489	0.174899	0.308695	0.613414	-0.70507
0.7327	102776.1	102205.5	94054.17	246068.7	216013.1	212995.3	1.360869	0.004568	0.032241	0.442965	-1.17473
0.7324	400.29	246.79	2939.36	2631.69	3515.75	995.53	0.769787	0.359859	0.500359	0.502094	-0.99397
0.7312	3330.8	218.98	745.47	630.91	215.14	332.61	0.603709	0.393019	0.528919	3.644181	1.865594
0.7307	34047.35	34756.82	49983.9	54683.11	44156.87	8210.01	0.392774	0.813898	0.869555	1.10965	0.150105
0.7298	160982.1	355928.1	163327	109981.6	115903.1	66172.03	1.076979	0.177406	0.311318	2.329127	1.219789
0.7249	97014.79	92944.16	96650.91	10591.09	41562.66	42018.21	1.117152	0.023834	0.080475	3.043473	1.605719
0.7248	55514.83	49845.64	52655.87	17346.87	15037.71	15942.63	1.367771	0.000522	0.012159	3.269718	1.709166
0.7235	12257.08	2861.24	1386.34	20675.9	20055.14	14482.07	1.067289	0.042197	0.117812	0.298926	-1.74214
0.7228	1359899	1072108	1206454	884130.9	720191.4	743756.8	1.262308	0.017449	0.066068	1.549548	0.631847
0.7226	17605.69	19744.55	30510.59	51726.02	42754.33	31526.32	1.121363	0.059577	0.148227	0.53855	-0.89285

0.7224	233203	83478.3	118240.9	167795.5	145862	135999.2	0.269345	0.924362	0.946288	0.967232	-0.04807
0.7207	127663.2	48867.84	120807.2	186182.7	172983.7	184649.2	1.016932	0.078852	0.179768	0.546763	-0.87101
0.7178	2747.06	2920.47	4125.97	5149.79	1910.43	6292.62	0.354456	0.466916	0.591482	0.733439	-0.44725
0.7146	127523.9	133472.6	119017.7	201987.6	189026.2	188701.7	1.348892	0.000394	0.010355	0.655518	-0.60929
0.7142	3482.53	1565.63	3526	941.91	581.16	5255.21	0.552027	0.740851	0.817562	1.264946	0.339076
0.7126	1795734	2524829	1845894	2180113	1769087	1699009	0.400791	0.57477	0.683981	1.091754	0.126648
0.7108	415.97	4407.43	224.12	265.82	769.61	502.35	0.320663	0.481729	0.604275	3.282342	1.714726
0.7103	3944.61	8954.28	3682.94	616.89	1194.58	2091.53	1.169378	0.125342	0.245226	4.248483	2.086948
0.7096	2277.32	720.21	6526.3	553.84	945.53	1166.59	0.793813	0.317975	0.46033	3.572383	1.836887
0.7093	8348.93	7657.37	11598.74	8229.89	6709.46	6506.69	0.875098	0.228001	0.368505	1.287186	0.36422
0.7068	17372.5	18439.19	17417.76	17360.4	13229.61	16307.76	0.849425	0.225745	0.366216	1.13501	0.182705
0.7048	2753.88	25812.44	12206.3	380.7	8439.2	5585.64	0.662061	0.319481	0.461897	2.830343	1.500977
0.7044	26473.2	20230.53	23503.93	19280.9	16436.99	18876.23	1.100364	0.08345	0.187137	1.285993	0.362883
0.7036	41086.92	28237.65	36239.05	9270.7	11311.48	7101.13	1.329097	0.013441	0.056593	3.813259	1.931024
0.7031	28379.99	21857.86	23286.66	8130.33	7633.97	2577.64	1.224535	0.00239	0.022717	4.008546	2.003079
0.7023	18130.01	21887.66	10740.44	64034.44	61825.59	62659.65	1.309982	0.003875	0.029122	0.269246	-1.893
0.7014	3903.78	5463.19	1530.45	7519.55	2486.58	7093.43	0.560096	0.360385	0.5006	0.637292	-0.64997
0.6994	8032.58	5014.24	8665.47	16964.08	13476.8	8116.74	0.972608	0.148095	0.273897	0.563113	-0.8285
0.6947	3096.68	1926.75	3156.58	2494.52	75103.3	15025.16	0.882219	0.335909	0.477258	0.088315	-3.5012
0.6946	38571.09	49170.29	43248.65	132458.1	104053.1	119110.3	1.346547	0.0061	0.036575	0.368341	-1.44089
0.6933	4525979	2217331	3646015	2944064	4853989	2752903	0.060185	0.957493	0.969503	0.984681	-0.02227
0.6933	22165.73	21468.36	30486.16	46091.67	36639.79	37354.49	1.203729	0.021862	0.076054	0.617227	-0.69613
0.6919	7032.4	10008.28	8268.93	6319.48	3852.04	3714.27	1.155757	0.034532	0.103171	1.822699	0.866076
0.6914	83754.03	128367.7	66878.73	90599.82	73780.29	83461.71	0.283101	0.633073	0.730623	1.12572	0.170848
0.6912	1693.14	1070.9	813.08	34880.7	31743.09	30546.25	1.361657	0.001187	0.015971	0.036813	-4.76364
0.6904	2824.48	3062.47	1899.42	26265.28	26387.96	6799.84	1.226139	0.117644	0.235234	0.130967	-2.93273
0.6899	48847.5	41815.42	47965.74	79989.39	82081.69	64486.82	1.28632	0.022093	0.076615	0.611891	-0.70865

0.6884	14809.61	16865.92	20814.34	22904.3	19352.29	21000.91	0.904909	0.170356	0.302477	0.829781	-0.2692
0.688	809305.4	780304.7	734341.7	91700.29	51996.63	52898.77	1.357827	5.27E-05	0.004503	11.82097	3.563277
0.6875	35270.55	31225.53	30774.44	25406.2	38329.04	21956.35	0.55846	0.524497	0.639877	1.135123	0.182849
0.686	10759.48	8310.78	8763.85	21609.44	21996.73	23084.76	1.350383	0.000438	0.011111	0.41736	-1.26064
0.6857	66675.47	51227.66	63693.68	51480.15	53547.22	43640.32	0.952104	0.13463	0.256991	1.221495	0.288648
0.685	8067.53	11694.98	12692.51	5778.05	4003.75	1635.19	1.128525	0.020055	0.07222	2.842695	1.507259
0.6813	31844.09	14204.49	22201.63	3872.62	9699.01	2734.98	1.186836	0.060299	0.149259	4.185432	2.065377
0.6812	112.95	776.97	302.4	12272.73	12892.75	11281.46	1.314411	0.000362	0.010037	0.032714	-4.93395
0.6805	7245.04	6915.68	2977.81	111614.1	103272.7	99321.94	1.347792	0.000374	0.010037	0.054545	-4.19641
0.6799	3502.4	6323.45	1467.97	6591.89	4270.58	2582.1	0.353829	0.715248	0.797587	0.840028	-0.25149
0.6768	8193.06	9504.44	11092.68	44955.1	43896.74	40647.17	1.362499	8.6E-05	0.005682	0.22232	-2.16929
0.6747	709.72	18.46	1312.32	21.42	1019.6	636.09	0.026531	0.811448	0.867574	1.216676	0.282945
0.6742	9191.6	12222.32	9363.8	15754.51	12952.01	12412.69	1.069547	0.073397	0.171071	0.7485	-0.41793
0.6726	844.43	2851.79	4572.7	5888.64	4721.41	3854.57	0.809738	0.188286	0.323507	0.571665	-0.80676
0.6725	9354.94	23365.32	16564.84	1991.23	10281.68	2751.47	1.06179	0.087886	0.194268	3.280342	1.713846
0.6721	6898.42	9088.06	8030.19	5098	3806.66	3424.22	1.264428	0.00964	0.046791	1.948001	0.961994
0.672	4356.6	4689.77	5361.8	3404.56	2597.68	2728.35	1.27171	0.008693	0.044113	1.650309	0.722736
0.6711	6389.87	7838.71	1168.88	480.34	687.29	270.82	1.170465	0.147955	0.273897	10.7042	3.420105
0.6701	1172.03	440.3	478.14	606.88	41.3	379.83	0.716972	0.295482	0.438246	2.033511	1.023973
0.6696	237105	224915.6	202678.2	316379.2	325155.8	491419.7	1.187198	0.107087	0.221526	0.586695	-0.76932
0.6683	1173.42	3320.48	534.07	147.17	770.6	230.2	0.973618	0.261841	0.407485	4.379879	2.130891
0.6646	6235.5	5013.13	5578.84	3270.6	1028.98	752.33	1.157187	0.024483	0.081843	3.330912	1.735917
0.6642	564591.2	990848.6	744354.2	164008.5	159860.3	127332.2	1.339586	0.036913	0.108666	5.097051	2.349663
0.6628	24935.34	10715.77	21575.39	7424.72	1367.3	1579.22	1.171236	0.049933	0.131232	5.517807	2.464095
0.6627	155.24	3.59	64.7	65.84	23.41	3.59	0.295376	0.435997	0.563704	2.407691	1.26765
0.6626	1389.53	3032.68	938.26	2021.57	3083.99	2246.01	0.684038	0.421736	0.551394	0.72916	-0.45569
0.6613	31754.64	29774.54	29130.56	184118.1	146554	161585.7	1.368073	0.006365	0.037563	0.184171	-2.44088

0.6611	4394.94	1616.15	4697.51	12833.65	25804.18	23883.24	1.252326	0.043968	0.121282	0.17128	-2.54557
0.6605	4734.5	32909.18	22041.47	105746.6	57808.51	103977.2	1.115414	0.029366	0.092491	0.223095	-2.16427
0.6588	5203.82	425.12	83.56	5686.79	491.92	5601.98	0.624719	0.443985	0.571569	0.484904	-1.04423
0.6577	27182.76	22954.32	30696.17	98295.17	104432.6	4798.77	0.208391	0.320259	0.462561	0.389508	-1.36028
0.6567	16086.54	27043.2	12038.92	13236.87	275.92	313.3	1.011527	0.091186	0.199378	3.990185	1.996456
0.6533	68880.48	64114.57	78553.54	57815.93	50862.11	52166.14	1.217083	0.038995	0.112432	1.315239	0.395325
0.6522	156.46	17613.53	224.06	145.28	7959.9	21667.9	0.379134	0.670468	0.760096	0.604373	-0.72649
0.6514	4234.87	3414.9	3323.84	11342.46	6573.94	9362.61	1.275871	0.053671	0.137774	0.402273	-1.31375
0.6496	1069.46	1973.35	617.34	2328.49	2141.64	2413	1.015697	0.109227	0.224903	0.531757	-0.91116
0.649	752.04	2695.63	363.04	14580.16	13421.54	12648.97	1.259751	0.000253	0.009348	0.093743	-3.41515
0.6476	33094.31	34399.82	33628.87	1200.15	3051.6	1117.56	1.342896	1.24E-05	0.003599	18.83352	4.235231
0.6443	177358.5	163053.4	127315.4	194549.1	25537.01	23780.73	0.873541	0.317178	0.459635	1.917962	0.939574
0.6409	14749.45	6617.52	6652.99	1071.86	2403.55	1234.83	1.269251	0.0995	0.210748	5.948733	2.572582
0.6377	12628.21	5177.6	11009.93	37649.85	32344.77	36090.73	1.270331	0.001237	0.016047	0.271628	-1.8803
0.6328	33492.32	28297.04	25809.24	11376.92	17956.73	1319.71	0.934993	0.041556	0.117093	2.857716	1.514863
0.6325	1043.45	350.53	388.14	13601.69	10394.93	10699.57	1.337692	0.006483	0.037805	0.051364	-4.28311
0.6307	2946.98	1754.43	383.32	288.7	113.61	1664.44	0.705585	0.329542	0.471284	2.460254	1.298807
0.6272	680.38	650.54	1710.24	1485.57	2259.18	2225.06	1.019589	0.092267	0.200995	0.509423	-0.97306
0.6265	8163.07	12703.24	24918.42	10797.84	7499.12	2251.99	0.838905	0.231315	0.372741	2.228081	1.155802
0.6263	2687.33	4015.12	1567.83	1941.38	1745.75	2148.4	0.616548	0.369863	0.507798	1.417229	0.503072
0.626	91276.26	122569.3	68904.8	310404.4	281136.1	259914.2	1.312009	0.000907	0.0148	0.332079	-1.5904
0.6223	2715.92	1864.4	1740.79	2576.26	2238.74	331.68	0.484943	0.646359	0.740857	1.228192	0.296536
0.6214	342239.3	317539.6	315253.2	1202.89	3514.39	1800.36	1.362572	0.000667	0.0135	149.5989	7.224956
0.6196	1968.68	1495.53	503.43	534.07	1370.08	366.32	0.61119	0.352939	0.493571	1.747497	0.80529
0.6138	22378.16	18712.31	19415.39	117909.7	118315.3	109937.4	1.369801	0.000159	0.007278	0.17479	-2.5163
0.6095	16076.05	10902.2	9993.44	36076.48	26516.47	31233.18	1.291269	0.006863	0.038918	0.394045	-1.34357
0.6039	168878.7	149132.5	155657.2	44074.54	43578.79	37278.02	1.366468	0.000835	0.0145	3.79143	1.922742

0.6036	44724.41	51795.39	41392.23	3136.89	4396.53	3577.95	1.366847	0.00473	0.032421	12.41179	3.63364
0.6029	421333.6	387478.4	319391	2238629	2752601	2015961	1.360795	0.011028	0.05044	0.161006	-2.63481
0.5953	16609.99	17043.5	13039.76	11540.86	19658.59	18105.73	0.132549	0.77579	0.842888	0.947025	-0.07853
0.5867	5431.69	12171.54	2060.29	10247.98	2787.33	4417.84	0.027111	0.853947	0.896953	1.126646	0.172034
0.5839	0.16	0.16	0.16	0.16	0.16	2.73	0.614848	0.42265	0.551654	0.157377	-2.6677
0.5777	768758.4	769255.2	693258.1	8456.53	10805.72	9735.01	1.371976	0.001173	0.015971	76.94767	6.265806
0.5756	126.25	131.55	103.98	220.37	329.03	333.28	1.296453	0.036782	0.108406	0.409865	-1.28678
0.5746	6093.87	5003.33	7769.45	12102.63	14777.42	11929.62	1.276487	0.005864	0.036053	0.486133	-1.04058
0.5736	19854.86	24307.47	16339.66	32967.14	34652.39	30508.5	1.234946	0.016802	0.064416	0.616562	-0.69768
0.5696	15013.61	17242.36	13746.89	5971.39	4711.4	4077.64	1.339379	0.002613	0.023767	3.116634	1.639989
0.5643	16159.17	12305.71	14396.26	29392.74	24396.95	18389.58	1.167025	0.078465	0.179354	0.593815	-0.75191
0.5585	3210.97	2705.4	2552.26	20863.14	21151.5	19858.61	1.36951	3.63E-05	0.00416	0.136871	-2.86912
0.5581	7290.99	8754.21	6061.4	662.62	174.2	844.23	1.290325	0.009277	0.045573	13.15047	3.717043
0.5512	898.13	398.67	488.26	725.05	479.86	1252.04	0.523397	0.466794	0.591482	0.726535	-0.4609
0.5481	24221.96	28225.97	22425.84	51606.61	28606.74	32117.72	0.958434	0.218764	0.358899	0.666546	-0.58522
0.5415	1891.09	1508.84	438.73	1714.65	2151.7	205.88	0.130041	0.920878	0.944718	0.942643	-0.08522
0.5415	162716.1	173686.6	144991.7	307074.6	331774.7	288429.9	1.345766	0.001153	0.015971	0.519147	-0.94578
0.5401	11523.37	2407.8	1614.93	2175.47	1844.1	7600.72	0.115654	0.744503	0.820138	1.337841	0.419907
0.5386	1472.29	788.25	3067.45	657.06	1809.11	2154.06	0.138877	0.788034	0.85093	1.153187	0.205627
0.5289	4634.6	2717.09	3932.44	14752.94	14036.08	13792	1.336378	0.000488	0.01162	0.265004	-1.91592
0.5091	472.04	329.58	1539.9	1282.63	658.08	158.8	0.172538	0.880202	0.915267	1.11527	0.157393
0.5061	16746.39	22136.23	22632.17	22506.42	18709.42	19189.27	0.077926	0.87774	0.913986	1.018371	0.026263
0.9603	23787.82	21617.5	20792.25	49025.67	56221.91	47583.16	1.356744	0.004578	0.032241	0.433143	-1.20708
0.9587	35137.39	30603.06	36609.95	16035.62	14300.62	15494.2	1.355946	0.005839	0.035947	2.233241	1.159139
0.9329	242324.3	297126.6	335643.9	310498.6	306872.6	522714.7	0.709554	0.343468	0.484649	0.767569	-0.38163
0.9163	25560.62	17601.35	19079.48	18594.36	15794.67	13597.47	0.906923	0.185916	0.320954	1.297062	0.375247

0.908	211455.1	278949	138049.2	180965.3	178647.1	186030.4	0.32732	0.567618	0.678433	1.151767	0.203849
0.8968	79996.51	83861.4	76188.94	163870.1	156005.4	157584.2	1.367256	1.82E-05	0.003728	0.502758	-0.99206
0.8962	9130.23	17192.26	12007.45	12847.13	2291.6	12826.8	0.558083	0.466346	0.591103	1.370614	0.454822
0.8932	46752.31	36496.14	50157.25	46045.33	37753.13	42241.87	0.305341	0.638921	0.734933	1.058437	0.081935
0.8913	56240.87	59536.88	68566.32	56933.1	57614.41	54246.82	0.779879	0.292664	0.4353	1.092122	0.127135
0.8781	187944.9	154640.6	108216.3	35956.85	73182.97	78551.87	1.147256	0.042079	0.117806	2.401821	1.264129
0.8585	34102.23	20818.19	27919.68	30299.37	30859.82	21448.81	0.014178	0.988198	0.991154	1.00281	0.004048
0.8473	152555.8	134494.6	142372	149186.7	128409.7	135285.9	0.453296	0.531507	0.647523	1.04006	0.056667
0.8367	151847.5	142577.2	124362.8	186518.1	158798.5	137821.4	0.75449	0.273711	0.418486	0.866807	-0.20622
0.8302	3403.34	1383.25	151.45	2962.52	475.93	324.76	0.087488	0.774492	0.842107	1.312188	0.391975
0.8035	38706.25	37354.67	37397	84547.15	69831.64	32216.82	0.794109	0.258065	0.403626	0.608042	-0.71776
0.7956	62452.36	53994.61	58186.75	17946.84	13006.58	13350.37	1.355554	0.000301	0.009591	3.941733	1.97883
0.7938	79640.98	64719.94	52529.26	15170.83	29657.44	67806.67	0.862666	0.20964	0.348521	1.748038	0.805737
0.7893	1165.61	658.37	400.21	1980.62	4224.57	1431.82	1.110114	0.162171	0.292189	0.291238	-1.77973
0.7771	10347.5	10283.4	7140.35	13996.96	16453.78	13724.08	1.193517	0.017448	0.066068	0.628667	-0.66963
0.7726	17970.01	19923.59	20143.07	21919.03	22307.38	20732.22	1.09765	0.058535	0.14664	0.89344	-0.16256
0.771	1728.51	1144.36	46105.46	185.64	542.47	2272.25	0.806484	0.411575	0.543818	16.32415	4.028936
0.7512	112078.2	157143	106570	111326.4	95517.08	97158.66	0.829685	0.270682	0.415793	1.236146	0.30585
0.749	107353.6	38046.28	94003.02	130202.1	116883.6	119876.9	0.852937	0.179344	0.313354	0.65239	-0.61619
0.7391	30690.99	14089.13	13791.09	1052369	884922	839646.2	1.3601	0.004804	0.032564	0.021092	-5.56716
0.7379	547241.7	179705.5	131190.2	1300069	1108882	1020843	1.200481	0.008565	0.043718	0.250201	-1.99884
0.7162	64364.41	60230.97	118775	154668.5	145421.3	152109.7	1.150831	0.063358	0.154777	0.538193	-0.89381
0.9931	17513.61	6632.8	13244.94	12273.99	8090.29	8819.43	0.392261	0.489032	0.609137	1.28124	0.357541
0.9877	7483.44	6300.53	4261.31	1111.56	1593.79	9772.84	0.696037	0.584738	0.692252	1.446146	0.532213
0.9842	50772.96	39079.4	38881.92	34385.02	32637.17	31320.5	1.122486	0.116987	0.234503	1.309038	0.388507
0.9824	49.54	5874.58	152.73	8507.39	7853.7	6980.55	1.002855	0.089226	0.196432	0.260344	-1.94151

0.9804	11157.11	6862.7	12420.69	18839.63	8875.21	19158.72	0.745723	0.244261	0.387881	0.649417	-0.62278
0.9766	1591.48	310.76	1477.71	2805.8	2023.86	1161.5	0.722737	0.238778	0.380979	0.564156	-0.82583
0.9664	20780.36	13265.18	18999.87	35307.45	29464.08	28267.91	1.211431	0.013277	0.056224	0.570139	-0.81061
0.9597	686134.4	435991.5	632658.2	123897.6	118513.7	110976.6	1.351105	0.025259	0.083162	4.965603	2.311969
0.9494	404540.7	398396.5	393345	147787.5	115674.7	124138.2	1.361723	0.000448	0.011141	3.08638	1.625916
0.943	15176246	14121816	13403277	4061046	3779591	486546.1	1.124143	0.003795	0.028964	5.127945	2.358381
0.9427	1259.48	178.17	392.86	320.44	1377.67	973.36	0.474835	0.568702	0.679171	0.685207	-0.54539
0.9356	1174571	932396.1	2061255	3418302	3088617	656710.6	0.417692	0.374792	0.512297	0.581859	-0.78126
0.9325	10181.42	7202.67	7610.95	18469.34	14705.33	13655.49	1.254043	0.019227	0.070294	0.533738	-0.9058
0.9316	34473.14	31552.05	32958.91	13851.57	14523.63	11788.69	1.357715	7.67E-05	0.00568	2.464505	1.301298
0.9287	85995.72	57858.72	69474.64	65232.82	52878.87	56297.35	0.814853	0.250043	0.394762	1.223154	0.290606
0.928	120628.3	134719.7	69756.71	11828.19	75279.98	155766.6	0.552468	0.59583	0.701005	1.338569	0.420692
0.927	4074.25	12004.37	2094.28	2660.82	805.93	1711.44	0.918014	0.287417	0.430681	3.509508	1.811269
0.9207	470252.3	775925.1	427373.5	588013.8	652863.2	497980.5	0.228893	0.867455	0.906542	0.962443	-0.05523
0.9201	5072069	4941443	4039476	10478862	6292981	10485156	1.189471	0.080699	0.182357	0.515574	-0.95575
0.9177	1928.76	4729.09	1218.7	4111.63	148.38	772.28	0.621791	0.592905	0.698885	1.565202	0.646349
0.9134	3094.33	8199.87	6609.1	24516.26	20959.55	17942.23	1.24547	0.003934	0.0292	0.282306	-1.82467
0.9093	50227.79	55083.43	136000.5	197822.7	186905.4	42367.97	0.506916	0.356059	0.497078	0.565006	-0.82366
0.9067	22232.93	20519.12	21146.72	3357.13	2514.83	5779.35	1.322583	0.000567	0.012605	5.484256	2.455296
0.9063	1554.09	1752.87	465.13	367.51	2190.95	1308.01	0.060361	0.964494	0.973652	0.97559	-0.03565
0.9028	550110.3	729017.7	507746.4	452327.9	502518.3	385086.7	0.993245	0.145971	0.271569	1.333555	0.415277
0.9026	156.57	1960.85	1769.9	157.8	1869.75	1364.71	0.060431	0.83993	0.887873	1.145938	0.196529
0.9015	1285.44	25630.13	48998.16	6787.44	8939.72	78843.57	0.180161	0.834029	0.883268	0.802719	-0.31703
0.9013	88333.25	69936.87	68966.65	114429.7	62234.65	60319.66	0.0076	0.875899	0.912534	0.95887	-0.06059
0.8997	274514.8	49764.77	260488	175348.2	177111.4	150057.4	0.1089	0.743135	0.819046	1.163677	0.218691
0.8994	8347166	9313904	6873852	16330002	19868438	15393168	1.309508	0.009769	0.047054	0.47556	-1.0723

0.8982	179176.9	171431.2	159048.5	327356.9	280840.3	272863	1.33394	0.011396	0.051426	0.578458	-0.78972
0.8981	172359.5	204577.2	158916.5	374724.4	360955.6	356076.6	1.343207	0.001887	0.020155	0.490817	-1.02674
0.8981	1353493	1995460	1294155	1414792	1488236	1200389	0.459727	0.516846	0.63338	1.131522	0.178265
0.8933	41023.71	38180.13	32054.86	8258.4	6366.31	10345.42	1.343474	0.003178	0.02649	4.455672	2.155643
0.8928	15931.73	16686.02	24053.82	105160.3	34153.75	18775.78	0.824312	0.331378	0.472823	0.358477	-1.48005
0.8919	14375.98	34143.86	18014.84	11133.12	63942.78	59996.4	0.530336	0.310333	0.453635	0.492586	-1.02155
0.8917	39542.41	35847.03	30329.08	6563.72	7165.86	16755.96	1.245202	0.004705	0.032349	3.467825	1.794031
0.8899	24795.65	35694.98	21446.06	72647.51	44793.68	58356.71	1.209448	0.040318	0.114643	0.466085	-1.10134
0.8898	55284.84	78789.85	94268.09	36850.78	32487.15	51880.8	1.14238	0.067939	0.162094	1.883725	0.913589
0.8891	11544.32	10403.92	12857.58	7257.57	5388.29	6787.69	1.287492	0.005572	0.034899	1.791017	0.840779
0.8864	100989.8	105988.9	131059.1	197095.6	79872.49	99676.52	0.088639	0.760188	0.831742	0.897498	-0.15602
0.8858	559544.4	385782.2	591860.7	449543.4	415751.7	413571.7	0.71685	0.309613	0.453191	1.201992	0.265427
0.8853	2101.75	1546.1	630.32	8380.38	2494.93	3705.56	1.052255	0.190607	0.32621	0.29341	-1.76901
0.8817	16012.47	13244.25	13913.66	10273.1	10657.69	7712.03	1.197475	0.018052	0.067314	1.507197	0.591868
0.8801	1834.97	3148.44	3271.58	30191.76	25770.1	24405.54	1.352952	0.003311	0.027078	0.102716	-3.28327
0.8799	307978.6	373055.4	269180.8	302406.1	251636.4	224960.5	0.837612	0.211747	0.35082	1.219783	0.286625
0.8795	24189.18	21039.96	881.49	211.74	2981.3	3532.71	0.725973	0.212126	0.351225	6.855835	2.777332
0.8753	4213746	3363603	3459168	579740.4	542300.1	467322.4	1.366333	0.006564	0.038048	6.943988	2.795764
0.8751	1581658	1558777	1593087	2423769	2042079	2002254	1.276186	0.049257	0.130493	0.731825	-0.45043
0.8745	6370.57	7491.41	13026.66	22126.3	28727.47	25909.97	1.265298	0.004151	0.030206	0.350278	-1.51343
0.8724	374883.3	376957.6	196356.6	2827807	537735.4	2531273	1.123277	0.147835	0.273897	0.160798	-2.63668
0.87	75625.42	67717.31	87401.41	103427.5	98824.26	83679.28	1.015316	0.090125	0.197713	0.806992	-0.30937
0.8685	254993.5	674532	276674.4	2331.48	8672.65	6757.08	1.334946	0.100867	0.2122	67.91203	6.085595
0.8673	566307.4	514760.9	1289949	909827.3	416052.9	515812	0.387227	0.585079	0.692468	1.287412	0.364474
0.8671	32000.17	35950.12	24801.48	183664	145581	135908.9	1.352078	0.010589	0.049419	0.1994	-2.32626
0.866	43603.5	63054.06	38899.32	32251.44	65.93	87.65	1.013431	0.051172	0.133408	4.4918	2.167294

0.8653	100484.5	90468.16	88275.44	73731.22	63804.71	63711.29	1.282727	0.006874	0.038918	1.387488	0.472475
0.8632	111686.4	125882.3	111407.4	122538.7	111357.3	102116.9	0.38804	0.601112	0.705695	1.03858	0.054612
0.8611	13810.21	14898.65	13535.11	44694.59	38565.16	40852.31	1.366543	0.002906	0.025269	0.34037	-1.55483
0.8585	43361.67	19327.35	37260.8	4148.58	9733.42	6116.62	1.259406	0.059684	0.148242	4.997836	2.321304
0.8585	44256.55	47372.53	128645.4	63651.04	64485.9	74654.44	0.082179	0.853085	0.896263	1.086212	0.119306
0.8534	5738.92	6627.42	8195.71	60231.68	49491.2	51685.98	1.363636	0.003475	0.027553	0.127391	-2.97266
0.853	22076421	17718417	18730411	33415249	29510014	27217837	1.269704	0.011481	0.051648	0.649248	-0.62316
0.8528	18208007	17275676	15272610	18559633	19399446	17290990	0.786294	0.23824	0.380957	0.918665	-0.12239
0.8515	182999.8	168414.2	147369.4	499213	454342.9	433862.7	1.358426	0.000787	0.014349	0.359505	-1.47592
0.851	642295.2	581592.1	601675.6	657375.2	637152.9	535149.1	0.009504	0.976005	0.981408	0.997751	-0.00325
0.851	581683.9	533097	513767.8	636317.1	573051.1	549348.5	0.762681	0.261494	0.407399	0.925987	-0.11094
0.8492	89765.99	56107.47	67124.14	33912.59	26671.49	24125.41	1.286622	0.040533	0.115178	2.514448	1.330242
0.8491	421899.5	407624.4	214211.3	1054135	1059612	890286.1	1.268763	0.001921	0.020396	0.347445	-1.52514
0.8476	296866.7	180287.4	376234.9	56378.29	264712.4	145755.8	0.81764	0.195514	0.332	1.827986	0.870255
0.8462	8418.22	2272.98	3008	19353.11	17127.79	16058.58	1.212226	0.009899	0.047469	0.260741	-1.93931
0.8458	3253.04	2654.41	1172.87	1838.98	1045.9	2695.39	0.354731	0.55872	0.672701	1.268813	0.34348
0.8456	169242.7	93982.07	155222.1	243386.3	226794.8	317122.6	1.155073	0.028581	0.090939	0.531494	-0.91188
0.8454	2237.55	2899.42	550.35	110.31	373.05	626.33	1.025796	0.155752	0.28395	5.125143	2.357592
0.8449	93279.52	66543.6	69903.61	32588.37	38023.15	30106.56	1.312103	0.02886	0.091558	2.280889	1.189596
0.8448	613725.8	564645.2	707979.5	580008	939093.6	507222.4	0.136019	0.765853	0.835633	0.930922	-0.10327
0.8445	189592.4	78216.79	119476.4	67226.3	28624.82	29582.66	1.14577	0.10073	0.212117	3.08757	1.626472
0.8442	5062.81	5121.36	3383.18	7833.91	6674.41	5077.35	0.967115	0.117853	0.235467	0.692718	-0.52966
0.8441	25844.16	31990.04	29657.39	40230.66	29973.58	33850.19	0.858555	0.203871	0.342325	0.840825	-0.25012
0.844	761631	1140612	921483.6	495517.7	1352626	1498901	0.14018	0.642163	0.737988	0.843648	-0.24529
0.8433	318.79	824.61	1084.49	975.66	705.72	679.2	0.283475	0.868915	0.907209	0.943789	-0.08346
0.8422	1347072	1147621	1122185	77407.95	1522037	1387440	0.510663	0.694735	0.780905	1.21092	0.276103

0.842	13.4	60.6	49.01	673.28	4240.52	235.97	1.179069	0.317336	0.45971	0.023887	-5.38766
0.8402	293657.8	269815.9	254093.3	610593.1	515729.5	496350.5	1.339916	0.010797	0.049697	0.50384	-0.98896
0.84	78297.34	66335.99	53830.9	33396.74	43191.84	30117.02	1.231744	0.030055	0.09378	1.859923	0.895243
0.8394	1388384	1242657	1492036	677541.5	747942.2	642175	1.34604	0.00465	0.032268	1.99408	0.995724
0.8388	186597	177395.9	186627.8	56539.36	47775.66	78139.46	1.329195	0.00257	0.023635	3.017853	1.593522
0.8368	98615.8	53565	76248.93	29581.75	34858.47	45155.15	1.177058	0.079787	0.181047	2.084301	1.059564
0.8367	785113.6	883790.1	688063.2	377878.1	391383.6	356697.4	1.343384	0.016053	0.062701	2.093297	1.065777
0.8353	8481.33	40346.08	40264.52	27822.45	4464.03	2258.31	0.797498	0.250455	0.395126	2.579027	1.366827
0.835	4582561	4451909	4780072	8646842	8079595	7655633	1.358683	0.003403	0.027425	0.566586	-0.81963
0.8349	40552.61	35562.25	28157.16	13960.07	11190.99	9396.41	1.321067	0.014425	0.059207	3.018224	1.5937
0.8348	182785.2	121870.2	120672.7	12752.73	10886.16	7020.81	1.353471	0.022824	0.078306	13.87254	3.79416
0.8344	8408.54	10014.2	7429.24	7554.93	5014.88	23153.3	0.165856	0.621489	0.722135	0.723677	-0.46658
0.8343	3301605	3509804	2901886	4901174	4296292	4195995	1.247475	0.013561	0.056899	0.725227	-0.4635
0.8341	3422639	2285953	3289123	52768.72	240451.5	556074.7	1.221052	0.00882	0.044309	10.59433	3.405221
0.8335	33137.09	32365.9	32721.61	89327.52	68003.28	155966.5	1.254644	0.113717	0.230341	0.313519	-1.67338
0.8327	260082.4	251624.1	308016.7	822446.2	775275.4	781875.6	1.362607	2.84E-05	0.003892	0.34448	-1.53751
0.8325	72271.2	55969.33	64317.05	82659.92	64520.08	68690.26	0.65572	0.344126	0.485035	0.892006	-0.16487
0.8319	8448.51	3872.65	34024.29	14721.78	1669.39	14540.66	0.266017	0.655154	0.7474	1.498309	0.583336
0.8314	552.2	887.77	478.29	18553.77	17038.97	16615.55	1.364194	0.000792	0.014349	0.036742	-4.76641
0.8307	15921.82	12454.96	14241.29	9173.15	12448.31	11052.29	1.046794	0.074269	0.172335	1.304352	0.383333
0.8294	14863.73	33636.19	23725.36	27681.28	11360.56	32780.71	0.079734	0.988085	0.991154	1.005607	0.008067
0.8293	43012.96	38228.51	41269.9	61321.91	47637.55	41213.67	0.849326	0.257224	0.402745	0.815801	-0.29371
0.8284	3485.46	4473.13	3752.96	2837.57	716.95	2988.48	0.856275	0.12991	0.252015	1.789936	0.839908
0.8279	211.9	745.93	594.04	5018.75	4254.14	2533.89	1.267991	0.038077	0.111235	0.131439	-2.92754
0.8272	6172.91	3558.36	6131.57	686.86	745.71	19579.97	0.494805	0.811346	0.867574	0.754923	-0.4056
0.8264	13943.2	9300.59	11061.55	27055.8	19516.69	24754.21	1.268485	0.014441	0.059218	0.480961	-1.05601

0.8244	53421.5	80418.22	62161.92	15575.92	10378.72	14564.89	1.341077	0.019451	0.070696	4.837214	2.274176
0.8236	236489.5	215467.6	226538.5	203237.8	191737.6	194702.2	1.248215	0.021524	0.075726	1.150621	0.202413
0.8232	606666.2	510331.2	604265.3	1106384	937909.3	862705.9	1.293326	0.018725	0.069055	0.59211	-0.75606
0.8201	13313.92	35407.58	36586.17	70694.51	51726.93	60066.36	1.065663	0.02978	0.093255	0.467471	-1.09705
0.82	7457.51	6587.05	1522	2915.92	133.3	658.76	0.930461	0.153802	0.281635	4.198124	2.069745
0.8197	491471.1	529387.5	444139.2	2604444	2210810	2433624	1.367786	0.002444	0.022956	0.2021	-2.30686
0.8176	711980.9	668091	666871.1	417689	314537.7	361804.2	1.326442	0.002679	0.024125	1.87101	0.903817
0.8162	53650.84	45411.63	58385.29	2432.26	439.47	45038.21	0.962607	0.120807	0.239254	3.286328	1.716476
0.8151	44698.64	40771.95	41084.14	57725.73	87583.25	53734.7	1.113267	0.150246	0.276803	0.635814	-0.65332
0.815	49859.16	54071.99	45157.94	17039.06	12247.72	10181.12	1.336387	0.000489	0.01162	3.777477	1.917423
0.8137	483331.7	437556.1	447345.8	519436.9	476159.7	456183.7	0.708755	0.302694	0.445458	0.942452	-0.08551
0.8137	493004.1	142328.3	219421.8	266648.3	123428.9	114879	0.630295	0.397479	0.532292	1.69273	0.759352
0.8126	45036.77	16227.4	19199.19	1957.18	1208.81	34850.81	0.844331	0.382649	0.519616	2.116521	1.081695
0.8125	33852.96	37101.17	36735.72	184642.5	168330.4	141445.8	1.364578	0.009057	0.044899	0.217811	-2.19885
0.8111	19252.15	16339.59	21597.49	18300.07	14196.5	15654.66	0.845572	0.198758	0.33607	1.1877	0.248171
0.8092	1438.47	2073.28	560.37	1025.74	14014.74	9517.84	0.867382	0.213544	0.352857	0.165814	-2.59236
0.8084	2785.04	2832.57	6635.26	686.88	2768.51	421.48	1.01678	0.148681	0.274619	3.160506	1.660155
0.807	11256.87	7725.12	8884.59	8543.12	2834.3	2726.87	0.995255	0.124222	0.243802	1.975752	0.982402
0.8069	22607.59	6729.24	22916.75	1233.86	1732.02	1024.2	1.294562	0.094701	0.20456	13.09587	3.71104
0.8067	13023.07	2505.15	6519.76	8368.59	9081.35	1993.99	0.113044	0.831616	0.881999	1.133926	0.181327
0.8067	4749.67	2404.67	2593.29	21914.77	16435.64	32319.28	1.318067	0.045625	0.123913	0.137932	-2.85797
0.8066	21491.66	17914.34	16548.79	43413.03	40673.85	35080.62	1.327806	0.003801	0.028964	0.469547	-1.09066
0.8063	9953.31	12525.23	7448.67	40113.76	40701.85	41992.02	1.34374	0.00072	0.013717	0.243692	-2.03687
0.8055	6728.64	7294.78	321.81	12055.79	11676.89	12098.68	0.83159	0.084671	0.188695	0.400354	-1.32065
0.8055	57239.32	37257.54	46248.42	54201.01	47530.36	41188.89	0.113089	0.922069	0.944828	0.984782	-0.02212
0.8049	194107.7	78016.47	216360.7	188158.5	158151.6	123505.3	0.07503	0.903355	0.931785	1.039738	0.05622

0.8049	4664.25	5575.95	8042.42	4420.27	4973.3	2377.97	0.876227	0.169605	0.301626	1.55312	0.63517
0.8047	248359.6	196649.4	238158.7	39410.58	36618.61	23087.35	1.34982	0.00354	0.027803	6.892569	2.785042
0.8044	28669.5	23373.97	29971.89	36479.78	29870.33	30492.53	0.884333	0.165614	0.296229	0.846893	-0.23975
0.8042	2270.46	1141.4	3089.44	4662.93	4069	8522.93	1.102368	0.109502	0.225363	0.376781	-1.4082
0.8041	288151.1	236565	264277.3	103392.4	105222.2	98182.41	1.361628	0.007547	0.040336	2.571711	1.362728
0.8035	2285.16	57.77	7417.73	3344.5	358.15	295.31	0.141126	0.485514	0.607645	2.44141	1.287715
0.803	415988	463088.7	300062.3	265669.9	244018.7	130766.7	1.067617	0.04971	0.131093	1.841095	0.880564
0.8026	11512.41	2144.03	13737.01	2930.92	1668.14	9687.2	0.528219	0.376909	0.514481	1.917468	0.939203
0.802	85829.52	73555.97	78371.03	443846.4	377628.5	359999.2	1.366473	0.005751	0.035761	0.201237	-2.31303
0.802	1092.3	447.02	342.97	15242.26	19609.67	13892.64	1.342137	0.01088	0.049974	0.038615	-4.69468
0.7987	955.49	1036.4	1043.2	4488.03	3220.97	3588.06	1.356342	0.017722	0.066712	0.268662	-1.89614
0.7983	8431.2	7150.17	2189.85	16302.41	37582.98	24485.22	1.168401	0.071771	0.168221	0.226759	-2.14077
0.7958	83298.44	268684.1	274482.9	60125.84	68072.08	169408.5	0.795471	0.221041	0.361545	2.105013	1.073829
0.7958	751.45	293.96	796.4	690.48	647.66	12895.21	0.671758	0.417496	0.548303	0.129401	-2.95008
0.7954	861.47	24840.29	14270.58	9510.28	7728.87	6385.62	0.092492	0.515286	0.631823	1.691967	0.758702
0.7951	75153.36	86059.99	73317.34	75250.66	60027.65	66854.79	0.927722	0.143563	0.268234	1.160278	0.214471
0.7944	42003.29	48203.04	51952.85	31281.06	34299.5	47461.81	0.893676	0.183833	0.319127	1.257574	0.330644
0.7938	9589.02	17799.78	10434.36	6935.02	34193.71	37765.38	0.577321	0.292896	0.4353	0.479417	-1.06065
0.7938	12916.39	16472.26	13760.45	27599.69	21432.01	20299.03	1.223609	0.043396	0.120257	0.622366	-0.68416
0.7932	26352.23	19995.74	16226.12	28181.89	30576.78	28344.37	1.064442	0.101226	0.212668	0.718392	-0.47716
0.7921	18421.58	14259.99	15207.71	28996.33	30665.3	32254.8	1.331628	0.001051	0.015525	0.521009	-0.94062
0.7918	1502.74	5145.37	1322.51	1355.01	6290.19	4471.55	0.44878	0.509024	0.626967	0.657818	-0.60424
0.7904	37468.17	25108.46	29667.41	12208.53	15498.84	12895.15	1.300916	0.033521	0.10119	2.27188	1.183886
0.7903	66441.4	72511.56	74689.82	192767.2	252080.2	230443.8	1.357693	0.011214	0.05113	0.316371	-1.66031
0.7901	10887.03	13324.1	12036.12	20447.2	17395.9	16707.37	1.267134	0.016214	0.063093	0.664472	-0.58972
0.7898	3710.96	5530.05	11819.96	153490.5	158105.7	116528.8	1.338633	0.007614	0.04042	0.049194	-4.34539

0.7893	29507.38	42248.94	35999.39	246047.2	175601.9	151286.9	1.342311	0.030224	0.094144	0.188076	-2.41061
0.7891	735.96	573.22	310.45	1768.62	176.25	62270.98	0.597515	0.414685	0.546579	0.025222	-5.30919
0.7888	125896.1	222742.7	133748.3	161037.4	164081.1	123358.8	0.157408	0.76178	0.83223	1.075611	0.105156
0.788	740990.4	618091.2	1370229	1965235	1789552	1695919	1.149154	0.048138	0.128732	0.500726	-0.99791
0.7854	7990.57	7461.96	8046.35	7439.46	6065.56	5538.67	1.056012	0.108415	0.223548	1.233946	0.303279
0.7849	97293.05	132287.5	138377.2	173264.4	54362.89	234998.3	0.107461	0.615879	0.718388	0.795368	-0.3303
0.7832	7422.77	25994.63	6820.17	10557.31	34963.88	2147.88	0.126167	0.844145	0.889874	0.844102	-0.24451
0.7827	122.43	57.21	1606	1057.57	1060.5	79.11	0.342647	0.832714	0.882734	0.812696	-0.29921
0.7819	4440.03	12931.69	7491.56	1684.93	1467.13	1295.05	1.279787	0.111061	0.227284	5.590885	2.483077
0.7815	32811.58	29916.04	30468.04	111152	89679.82	90276.25	1.360699	0.010346	0.04881	0.320141	-1.64322
0.7811	82.67	234.8	692.17	38.2	5804.16	7425.87	0.553116	0.209231	0.348105	0.076095	-3.71606
0.7801	9031618	9467404	8204684	2103325	1839588	988912.1	1.325033	0.000139	0.006718	5.414568	2.436846
0.78	114184.5	80270.33	79860.51	151954	156586	114552.3	1.121962	0.04853	0.129383	0.648358	-0.62514
0.7799	80574.37	77809.67	74764.69	63732.14	48819.23	49431.11	1.237216	0.029143	0.092055	1.439345	0.525413
0.7788	490673.9	948267.7	451811.5	404298.4	443231.5	326320.1	0.887759	0.270297	0.415793	1.610728	0.687713
0.7777	212060.7	175462.3	171412.7	237749.4	217622.1	201579.3	0.96764	0.124004	0.243653	0.850803	-0.2331
0.7774	108.36	57.94	105.3	88.32	69.45	2341.99	0.558976	0.428656	0.556105	0.10865	-3.20223
0.7764	12093868	10799815	10674481	1265059	1385810	1147206	1.370282	0.001707	0.019081	8.838202	3.143753
0.7761	8804.74	3150.78	9265.03	44721.42	44034.45	41981.62	1.289297	0.000815	0.014373	0.162314	-2.62314
0.7759	7373.92	10684.9	8163.93	1531.47	30303.57	26504.28	0.151811	0.357158	0.498033	0.449487	-1.15365
0.7748	7613.86	8168.84	7606.83	253.13	292.06	6324.09	1.014547	0.110856	0.227077	3.404946	1.767632
0.7746	10035.82	3452.97	1219.9	5794.14	11095.14	10049.75	0.795626	0.272449	0.417289	0.545999	-0.87303
0.7742	32460.36	45849.53	32206.97	26960.05	22799.92	27691.69	1.09228	0.122852	0.242313	1.426914	0.512898
0.7731	440315.1	426216.3	428829.6	57416.3	20905.94	56131.24	1.321459	0.00027	0.009571	9.634269	3.268175
0.7731	237635.7	199524.4	224328.5	115830.3	125865.2	121759.1	1.348579	0.008858	0.044329	1.820003	0.863941
0.7731	180191.7	118961.8	158950	315036.7	168496.6	314167	1.013605	0.133637	0.25583	0.57428	-0.80017

0.7724	73964.46	99516.34	90289.84	132455.5	156043	126304.7	1.239899	0.013807	0.057649	0.635893	-0.65314
0.7719	6647.37	5923.16	5072.4	837.06	665.8	34.08	1.141124	0.001734	0.019282	11.47926	3.520957
0.7716	28905.76	26111.03	35050.87	278.64	4243.1	25437.36	0.893728	0.112425	0.228705	3.006354	1.588015
0.7715	554174.2	440217.5	465872.4	107821	101034	97455.43	1.366534	0.00759	0.040398	4.767269	2.253163
0.7714	357077.2	319962.5	328305.8	1267869	1125478	1170423	1.368956	0.001353	0.016706	0.282102	-1.82571
0.7712	56033.04	43119.07	39347.37	132817.5	129060.6	116450.2	1.340216	0.00035	0.010018	0.366083	-1.44976
0.7697	2654.79	4935.92	8312.61	2736.18	432.9	1322	1.026444	0.133403	0.255606	3.54109	1.824194
0.769	35927.36	25463.08	33121.98	109669.9	107436.1	90691.76	1.34562	0.001792	0.019432	0.30706	-1.70341
0.7679	190883	246929.8	337060.2	237308.2	200053.2	211649.4	0.55914	0.430457	0.55778	1.193929	0.255717
0.7674	20894.08	20134.41	18831.34	4891.55	3912.7	575.01	1.167223	0.001807	0.019494	6.382148	2.674042
0.7659	1024.86	650.76	315.38	446.45	503.87	242.28	0.658315	0.324812	0.466817	1.669462	0.739383
0.7654	109630.8	115160.7	101628.5	160737.5	139268.4	144640.3	1.289877	0.010712	0.049629	0.734112	-0.44593
0.7638	5137.12	6257.53	1282.45	59528.68	32582.81	47112.01	1.269957	0.028694	0.091097	0.091056	-3.45711
0.7633	205.56	294.16	658.04	1461.84	160.02	111.57	0.111886	0.713098	0.795634	0.667901	-0.58229
0.7616	4813.34	7312.57	7123.12	1465.19	3437.89	1766.4	1.215673	0.016391	0.063341	2.886137	1.52914
0.761	21698.08	14782.95	10520	32994.18	7844	28368.21	0.343641	0.449238	0.576797	0.679143	-0.55821
0.7592	2368.55	5603.48	3395.49	3273.01	25822.2	679.85	0.051027	0.523196	0.638648	0.38178	-1.38919
0.7588	80866.56	69957.86	83106.97	214826	199557.5	181918.9	1.356837	0.002154	0.021199	0.392303	-1.34996
0.7584	1665.17	4521.21	4080.04	1719.23	823.62	5187.32	0.491809	0.628965	0.728197	1.328098	0.409361
0.7583	473.08	6096.44	2687.06	3758.92	3430.7	3943.7	0.525954	0.739393	0.816568	0.83143	-0.26633
0.7581	256648.2	235439.9	194542.3	1438332	1209594	2020580	1.351336	0.030914	0.095503	0.147077	-2.76536
0.7579	67802.3	67632.38	68361.11	65195.38	65789.97	61011.24	1.074263	0.117419	0.235019	1.061455	0.086044
0.7577	1721.38	1906.37	2276.31	2305.59	4341.02	2341.5	0.889888	0.263111	0.408452	0.656874	-0.60631
0.7571	207678.7	476079.3	549867.1	160730.1	169103.5	129583.7	1.131541	0.129307	0.250958	2.685195	1.425027
0.7564	9212.19	8354.65	6476.72	7384.87	12686.56	7962.72	0.440235	0.528831	0.644443	0.857653	-0.22153
0.7545	7974.45	4928.37	8639.88	8108.06	8198.86	83581.68	0.694579	0.408061	0.540517	0.215667	-2.21312

0.7537	286087.7	419762.6	336145.1	1954.89	543.08	1711.48	1.357943	0.01246	0.05415	247.5372	7.951501
0.7535	1584.24	3590.94	1204.82	1393.63	508.19	1553.37	0.731687	0.321505	0.464052	1.846498	0.884791
0.7534	4181.67	2721.25	2381.44	12360.97	4042.98	9211.49	1.074919	0.148009	0.273897	0.362452	-1.46414
0.7529	67684.58	46931.47	29433.99	15738.76	23692.36	29356.47	1.046687	0.139973	0.264131	2.094128	1.06635
0.7509	9255.07	199115.2	228682.1	2550.89	25380.71	34500.47	0.753424	0.20912	0.348054	7.000447	2.807447
0.7502	2175631	1851850	1906388	1855720	1408861	2100641	0.548961	0.464508	0.590146	1.105988	0.145335
0.7491	433.99	8.37	398.03	438.79	449.65	162.62	0.492048	0.695777	0.781472	0.799564	-0.32271
0.7487	823.85	252.49	527.18	786.3	208.38	662.16	0.004731	0.944759	0.960626	0.967818	-0.04719
0.7483	1110.4	99.22	1077.4	1425.51	382.24	361.22	0.124741	0.939014	0.95657	1.054427	0.076459
0.7481	12435.17	11752.83	13103.8	38412.51	30948.13	30502.62	1.354341	0.013244	0.056224	0.373429	-1.4211
0.748	42657.82	18600.4	35950.69	98039.4	86662.43	71526.54	1.213953	0.007342	0.039748	0.379384	-1.39827
0.7473	11491.72	16003.93	3139.4	4113.39	9697.92	9742.73	0.154925	0.614787	0.717499	1.300628	0.379209
0.7462	84091.34	86647.25	83086.12	64356.34	66301.9	49430.07	1.210557	0.039466	0.112856	1.409446	0.495128
0.7459	28274.73	23394.86	22884.83	8594.24	5798.4	6561.73	1.343726	0.002991	0.025612	3.557941	1.831043
0.7457	208714.3	169059.8	183476.9	73451.33	63470.17	61689.89	1.356273	0.005287	0.034293	2.825876	1.498698
0.7454	23783.39	29018.6	21662.26	35094.73	25188.21	19404.25	0.16184	0.755223	0.828598	0.934457	-0.0978
0.7451	105082.1	134019.2	127605.4	60390.19	56167.37	46905.69	1.328701	0.007462	0.040107	2.243359	1.16566
0.7445	609112.4	492787.3	590524.7	872382.6	757516.9	712641.9	1.203731	0.024988	0.082768	0.722474	-0.46898
0.7441	1467.52	14.57	397.28	7708.76	4147.42	2926.03	1.018658	0.084928	0.188975	0.127137	-2.97554
0.744	174371.7	260065.9	246532.9	102110.1	73144.97	57962.39	1.283464	0.016317	0.063167	2.919894	1.545916
0.7438	16834.17	33108.51	17151.48	27789.55	18699.96	16914.42	0.071851	0.85732	0.899846	1.058202	0.081615
0.7437	47121.07	38617.31	33752.99	627.36	66	4141.63	1.196104	0.005952	0.036092	24.71388	4.62725
0.7435	337.59	2102.27	55.16	354.67	800.85	2604.09	0.520371	0.677078	0.765797	0.663638	-0.59153
0.7433	16817.34	13563.2	15648.26	853.42	1077.09	1823.38	1.350378	0.002381	0.022717	12.26163	3.616079
0.7432	342.07	775.82	192.55	39.27	270.04	1516.36	0.229219	0.753152	0.826741	0.717786	-0.47837
0.7421	10692.59	16109.56	64901.38	89832.86	79154.56	81913.64	1.05517	0.086647	0.192016	0.365497	-1.45207

0.7416	22057.38	7293.14	14307.89	3563.37	543.04	7364.04	0.974456	0.112226	0.228705	3.806164	1.928338
0.7415	3787.85	5340.71	8922.01	1445.51	1012.88	417.93	1.243572	0.074595	0.172646	6.275578	2.649748
0.7408	2304.93	2693.95	2275.97	42602.5	30125.73	5842.12	1.182825	0.158569	0.287881	0.09259	-3.433
0.7408	2538.96	3150.37	3292.61	1579.35	1481.28	1618.64	1.328614	0.022236	0.076928	1.919517	0.940744
0.7405	981430.6	833200.3	934600.6	392966.9	106693.3	52477.43	1.174506	0.010903	0.050026	4.97925	2.315929
0.7403	110426.6	148802	103940.6	1515.3	27139.67	9058.84	1.174783	0.005918	0.036092	9.629608	3.267477
0.7403	63326.96	37308.6	33919.38	65595.6	94679.16	110260.2	1.129379	0.053684	0.137774	0.497366	-1.00762
0.7403	51335.06	49730.15	41632.41	66842	70392.75	61329.73	1.247756	0.009977	0.04767	0.718646	-0.47665
0.7402	8743.26	6366.21	6836.34	10977.53	12808.08	10874.17	1.241265	0.012135	0.053419	0.633178	-0.65932
0.74	314051.4	258595.1	306675.1	363653.7	313956	312196.5	0.827512	0.203129	0.34152	0.888378	-0.17076
0.7399	14356.65	10652.46	14099.09	2164.62	2833.37	2838.21	1.354629	0.010793	0.049697	4.99071	2.319245
0.7389	13496.03	772.25	10061.54	1125.39	5967.23	10436.68	0.080845	0.654441	0.746978	1.387952	0.472957
0.7374	37679.05	37034.12	44273.42	68445.46	61477.69	56776.11	1.295863	0.007608	0.04042	0.637317	-0.64992
0.7374	65165.44	58138.55	68842.98	79881.43	77011.73	69716.34	1.084147	0.058002	0.145613	0.847921	-0.238
0.737	26278.77	22374.33	25184.53	32811.64	20073.79	32031.46	0.456952	0.468497	0.592108	0.869528	-0.2017
0.737	26426	12852.73	30854.8	556.59	1127.41	5415.52	1.207077	0.051389	0.133654	9.87863	3.304311
0.737	271366.2	263431.7	306574.3	153517.2	153748.6	163834.7	1.352128	0.008093	0.042098	1.785972	0.83671
0.7366	1197.27	552.6	1156.56	204.23	182.22	58.39	1.236444	0.053097	0.13716	6.533653	2.70789
0.7364	172396.3	179347.2	169860.2	1788953	1222789	1608919	1.365284	0.014593	0.059559	0.112885	-3.14707
0.736	3906.22	13746.42	14927.02	3705.14	11520.57	16437.91	0.048658	0.955086	0.967853	1.02893	0.041145
0.7359	26668.45	29675.17	38474.75	159835.1	123394.3	105776.3	1.337408	0.021135	0.0749	0.243745	-2.03655
0.7358	8662.27	7375.22	8338.41	28563.03	38696.63	27932.9	1.353326	0.020087	0.07222	0.256069	-1.96539
0.7357	17240.76	19442.69	15860.5	5441.6	4887.68	2111.78	1.274098	0.000803	0.014349	4.22343	2.078415
0.7352	9286.07	34418.45	28962.96	124195.5	165568	158297.9	1.260759	0.002522	0.023404	0.162182	-2.62431
0.7347	824323.3	1384454	677532	456542.2	428205.4	405822.2	1.193125	0.131567	0.253424	2.236461	1.161218
0.7344	5339.42	4460.29	2507.21	11297.69	10403.87	10761.34	1.252412	0.009888	0.047468	0.379107	-1.39932

0.7342	37358.72	26867.99	28392.24	65119.71	56590.49	53090.97	1.288177	0.004931	0.033105	0.529853	-0.91634
0.734	3641.26	4289.57	4499.01	757.28	1347.2	1690.47	1.274187	0.001574	0.018061	3.275363	1.711655
0.7334	133483.3	112594.7	63123.32	263640.1	227646	190260.9	1.181932	0.013988	0.058128	0.453676	-1.14027
0.7332	10505.6	7793.52	16674.99	34.9	49.6	133.14	1.352051	0.047772	0.127926	160.6971	7.3282
0.733	5358.09	26689.84	44775.88	675.2	690.77	29293.13	0.808447	0.360492	0.5006	2.505743	1.325238
0.7328	6357.23	14478.86	10525.55	4750.23	12383.57	2666.94	0.704599	0.367361	0.505898	1.583862	0.663447
0.7328	42775.09	90376.92	82146.61	342960.5	306764.4	320673.5	1.312792	0.000279	0.009591	0.221866	-2.17224
0.7327	50059.23	43078.66	49598.53	10575.32	2318.41	1726.94	1.261106	0.000403	0.010522	9.762646	3.287272
0.7324	151.07	541.26	23219.3	1014.55	1232.9	939.71	0.074048	0.460584	0.586871	7.502488	2.907369
0.7316	41762.73	45579.92	50463.18	43970.01	36453.87	40127.55	0.901928	0.160178	0.289716	1.143129	0.192988
0.7311	60212.69	37245.07	60754.62	159820.6	130005	126787.9	1.291671	0.003665	0.02833	0.379758	-1.39685
0.7308	17526.09	7593.88	19654.91	47007.13	31514.44	38756.7	1.164394	0.01522	0.060802	0.381783	-1.38917
0.7308	89441.38	92701.62	64753.48	185821.7	139062.4	131701	1.224178	0.035093	0.104415	0.540746	-0.88698
0.7306	244	911.12	217.19	2163.5	1403.13	2972.88	1.186051	0.043753	0.120862	0.209849	-2.25258
0.7301	14066.01	3040.37	17645.86	12270.15	2112.51	3607.48	0.573301	0.365983	0.50437	1.931738	0.9499
0.7301	1150.52	2736.53	7618.34	1509.13	435.9	6182.31	0.41233	0.690433	0.778219	1.41564	0.501455
0.7295	52380.11	42896.02	39055.85	11196.12	1030.1	963.69	1.207409	0.001647	0.018733	10.18445	3.348296
0.7294	34673.86	42002.71	21934.67	22638.62	12065.49	17571.41	1.050624	0.101236	0.212668	1.886375	0.915617
0.7294	161689.1	113102.2	146886.5	271864.5	265110.6	251366.5	1.298461	0.006372	0.037563	0.534892	-0.90268
0.7292	8963.2	4500.26	8228.98	5454.05	5230.2	7074.24	0.454124	0.452797	0.580001	1.221525	0.288683
0.729	44656.27	38474.07	46072.75	8748.89	3800.21	137.63	1.083035	0.00035	0.010018	10.18411	3.348248
0.7288	221227.2	151306.8	166104.4	46376.91	47620.66	10049.19	1.191984	0.008077	0.042062	5.176887	2.372085
0.7283	1937.17	1070.97	1856.19	3644.19	3749.08	2489.64	1.156533	0.032427	0.099123	0.492196	-1.02269
0.7281	170757.3	104316.6	158752.4	75008.35	68458.85	71725.89	1.248029	0.069263	0.164027	2.015986	1.011486
0.7278	16471.91	43016.96	52840.71	99540.61	108766	43022.19	0.919083	0.139129	0.26352	0.446943	-1.16184
0.7277	3842.36	1757.72	9631.15	3393.56	2638.4	2056.83	0.522688	0.418979	0.549438	1.883005	0.913037

0.7275	8079.52	1115.05	12100.87	4575.71	2518.19	620.29	0.595597	0.292153	0.434937	2.760554	1.464958
0.7273	45442.78	45191.63	53343.1	44405.94	44871.9	45453.93	0.688779	0.368468	0.506354	1.068623	0.095753
0.7273	86742.01	73109.63	80516.18	404878.1	379170.6	350338.9	1.368141	0.001698	0.01908	0.211892	-2.2386
0.7272	94.07	1255.37	1756.42	1883.63	222.84	1848.28	0.253297	0.720602	0.80212	0.785349	-0.34859
0.7269	2579	71.03	9869.47	9444.62	5657.73	9617.22	0.735268	0.302032	0.4449	0.506461	-0.98148
0.7267	107335.7	17224.52	93866.88	31216.44	24787.01	14909.7	0.802323	0.219391	0.359656	3.080205	1.623027
0.7263	5523.88	1272.4	4051.36	3066.18	5429.6	660.64	0.276874	0.776744	0.843452	1.184703	0.244526
0.726	3399.35	4025.68	696.7	3394.92	767.12	3037.73	0.057184	0.826749	0.879619	1.128054	0.173836
0.7256	45.41	116.33	28.01	584.96	478.92	346.52	1.269707	0.017107	0.065183	0.134536	-2.89393
0.7255	47619.7	87561.76	238929.7	661.54	137925.3	69034.56	0.59807	0.480501	0.603429	1.801891	0.849512
0.7254	7387.65	7966.71	7826.41	4700.09	3522.02	4019.38	1.32645	0.002544	0.023542	1.893623	0.921149
0.7247	107290.5	45480.79	58882.52	24974.21	39947.52	28878.75	1.094641	0.165593	0.296229	2.256425	1.174039
0.7243	29207.69	19259.96	47570.06	23780.7	28511.7	22794.87	0.441387	0.490396	0.609882	1.279015	0.355033
0.7241	132212.3	58622.51	24710.15	53736.86	82297.13	48190.08	0.049419	0.779635	0.84538	1.170015	0.226527
0.7239	15735.28	8487.53	9906.48	22579.05	17230.52	16094.36	1.062024	0.072586	0.169637	0.610499	-0.71194
0.7238	13564.87	12254.59	8157.33	11532.41	4500.6	4511.02	0.881669	0.200073	0.337377	1.653852	0.72583
0.7238	2075.68	256.57	6621.19	8682.3	955.01	1758.47	0.273784	0.806496	0.86415	0.78568	-0.34799
0.7238	656.52	313.84	926.58	13.62	288.18	1093.83	0.586117	0.680863	0.769081	1.3592	0.442758
0.7232	42869.01	38321.62	34357.31	25420.4	22450.19	14147.27	1.18566	0.015622	0.061737	1.86314	0.897736
0.7226	2543.43	4602.49	2744.78	1156.24	710.99	449.24	1.255257	0.050503	0.132217	4.269729	2.094145
0.7224	30861.9	48370.45	29552.58	36995.11	36469.77	6224.51	0.565716	0.468351	0.592108	1.365112	0.449019
0.7222	4841.76	1938.85	1363.61	4894.55	1442.62	820.34	0.274423	0.85307	0.896263	1.137857	0.186319
0.7219	12002.89	22373.57	23946.17	87687.62	86092.33	68951.22	1.307841	0.002054	0.020822	0.240277	-2.05723
0.7216	3517.87	1271.34	2245.85	44896.08	38525.87	34379.17	1.343571	0.005128	0.033664	0.05972	-4.06565
0.7216	4647	3147.69	7774.55	588.98	492.84	112.33	1.261108	0.070651	0.166267	13.03793	3.704643
0.7214	16988.7	13749.71	13106.29	9350.17	9641.68	699.21	0.818137	0.095642	0.205776	2.22663	1.154862

0.7208	32079.25	25673.97	26908.94	65608.03	40923.44	51504.54	1.228404	0.066838	0.160323	0.535714	-0.90046
0.7198	52168.91	42705.99	47772.56	79654.15	56515.28	66140.3	1.140272	0.081718	0.183898	0.705094	-0.50411
0.7198	4600.81	3263.08	4032.16	580.39	681.43	1351.82	1.294435	0.004578	0.032241	4.551526	2.18635
0.7196	18043.07	18880.92	14419.08	137671.1	112173.4	125575.9	1.365835	0.00362	0.028133	0.136762	-2.87027
0.7193	10885.82	18792.82	11102.62	22729.79	19826.02	19982.99	1.070755	0.094616	0.204478	0.652095	-0.61685
0.7191	29747.35	9557.5	9386.67	3909.34	4299.14	3496.14	1.172915	0.209561	0.348521	4.160026	2.056592
0.719	117831.1	32130.2	71626.86	371986.5	410838.4	420832.1	1.267214	0.000967	0.014975	0.184096	-2.44147
0.719	104934.8	81628.21	105704	19655.97	12965.83	17210.81	1.354107	0.006727	0.038427	5.864974	2.552125
0.7189	3151.15	2265.86	1098.67	2325	1101.29	2598.58	0.090222	0.839005	0.887675	1.081464	0.112986
0.7189	32905.66	41529.61	12338.76	143336.6	193025.7	131794.3	1.259703	0.010423	0.048959	0.185353	-2.43166
0.7185	23187.18	24813.04	18238.31	401676.3	339433.5	322713.2	1.36834	0.004942	0.033105	0.062265	-4.00544
0.7184	15075.57	15099.87	13090.07	86313.38	89274.67	75574.24	1.368716	0.002957	0.025562	0.172261	-2.53733
0.7184	412030.1	439297.4	458182.7	141503.4	99667.66	91314.26	1.347169	0.000106	0.006088	3.938551	1.977665
0.7182	227812.2	61127	9863.86	1360.33	1540.92	2139.64	1.209957	0.275068	0.419815	59.27584	5.889372
0.7179	11988.17	17259.25	8926.48	7434.83	10354.59	6510.89	0.930911	0.189074	0.324095	1.570922	0.651612
0.7173	17054.27	6751.84	13648.22	3627.18	4644.36	1459.14	1.152237	0.08177	0.183919	3.849097	1.94452
0.7171	34965.15	38830.98	22115.37	76445.85	64546.4	86489.75	1.25942	0.006499	0.037825	0.421622	-1.24598
0.7167	4523.22	19548.6	5060.75	42608.05	40467.78	39429.45	1.196731	0.020993	0.074519	0.237807	-2.07214
0.7167	2368.2	2330.32	2696.94	1494.48	2521.47	2326.4	0.634423	0.385001	0.521933	1.166044	0.221622
0.7162	15191.74	10016.82	10763.81	3500.72	288.14	3661.97	1.053156	0.011283	0.051196	4.827968	2.271416
0.7155	731.82	175.14	736.59	219.36	172.54	55.79	0.968336	0.158915	0.28839	3.671179	1.876243
0.7149	469894.8	167683.2	395834.8	910308.9	830049.3	831282	1.156376	0.022832	0.078306	0.40185	-1.31527
0.7145	46737.76	89216.12	48496.76	44323.82	39898.07	28902.38	0.936404	0.222888	0.363203	1.630513	0.705326
0.7145	37614.01	32538.11	46428.91	27051.98	24414.46	20178.94	1.201017	0.047232	0.127089	1.627195	0.702387
0.7145	108752	83878.41	87854.95	427747.4	401706.3	424746.1	1.364872	9E-06	0.003019	0.223637	-2.16077
0.7137	61206.26	47065.49	38804.93	76090.41	69019.12	76595.46	1.157926	0.048889	0.129866	0.663389	-0.59207
0.7133	105027.3	80776.11	91864.72	708.9	2048.94	838.31	1.357304	0.005692	0.035547	77.2126	6.270764

0.7129	76002.23	43101.16	43439.79	91872.7	66275.06	75644.68	0.935747	0.156271	0.284657	0.695246	-0.52441
0.7128	54460.76	17281.55	22500.21	26951.5	17357.79	31324.64	0.212691	0.655919	0.747686	1.246035	0.317345
0.7127	7859.07	7793.79	5070.96	29508.59	16953.9	28868.3	1.291034	0.041099	0.116181	0.275104	-1.86195
0.7127	2116.76	969.34	2910.21	3481.52	2784.65	797.95	0.10365	0.737286	0.815072	0.84884	-0.23643
0.7126	9583.57	8898.95	11106.97	11914.83	3389.92	6923.07	0.662614	0.42781	0.555173	1.331192	0.412718
0.7125	2456.31	2248.01	2388.5	1975.52	4097.28	1537.2	0.045044	0.847828	0.89203	0.932039	-0.10154
0.7125	59854.95	45528.66	46235.61	105654.7	90720.79	87959.15	1.302971	0.003891	0.029148	0.533242	-0.90714
0.712	176.41	47712.94	21379.21	19974.71	12028.39	160.55	0.152282	0.474072	0.597421	2.153629	1.10677
0.7115	128337.6	114603	109370.9	208728.4	185635.9	179367.6	1.32527	0.00403	0.029577	0.61407	-0.70353
0.7115	15614.04	10155.05	8612.84	128682.7	48636.05	22741.13	1.131198	0.225008	0.365157	0.171858	-2.54071
0.7109	87437.14	79146.38	92521.92	97582.01	98477.46	71882.44	0.151451	0.779515	0.84538	0.967021	-0.04838
0.7106	44741.19	38874.91	40630.11	102614.1	67154.51	71719.49	1.262687	0.069193	0.164027	0.514502	-0.95875
0.7103	476404.9	412191.3	403933.1	657649.1	206941.1	300861.6	0.419174	0.788266	0.85093	1.109037	0.149308
0.7094	1168242	1177922	1018006	3445601	1705615	2879898	1.217628	0.092062	0.200825	0.418892	-1.25535
0.709	25453.17	22009.46	26961.02	8692.21	15059.51	14124.24	1.215245	0.009678	0.046822	1.964931	0.974479
0.708	21854.12	14219.84	20491.1	17284.16	15153.43	20593.25	0.232912	0.701792	0.786504	1.066645	0.09308
0.7078	208.82	1040.93	373.08	464.53	241.63	633.18	0.055232	0.758562	0.830588	1.211664	0.27699
0.7077	3143.99	4734.28	8720.9	107172.7	107256.9	78410.75	1.342386	0.009108	0.045011	0.056683	-4.14093
0.7065	74182.65	57236.68	77901.1	81841.93	60008.11	68750.86	0.034784	0.964381	0.973652	0.99392	-0.0088
0.7065	6350.86	11751.08	10805.48	3966.89	3088.72	4735.44	1.217349	0.066002	0.159276	2.451641	1.293748
0.7061	309.4	1115.51	365.51	7181.26	6486.44	7050.6	1.312101	6.24E-05	0.005041	0.086417	-3.53254
0.7061	92033.97	101753.7	82794.7	30899.13	41858.93	32421.82	1.337999	0.001928	0.020396	2.629613	1.39485
0.706	8464.22	843.55	7443.44	83099.15	63666.72	79442.25	1.226331	0.002961	0.025562	0.074052	-3.75531
0.7058	16188.75	9791.54	15525.11	10876.75	11679.62	11013.37	0.687961	0.322062	0.464214	1.236393	0.306138
0.7051	3404.66	2050.72	3326.53	293.89	404.96	210.19	1.340778	0.025257	0.083162	9.660642	3.272119
0.7047	35363.64	38947.29	25215.85	22320.18	24578.98	20947.74	1.093258	0.116158	0.233919	1.466932	0.552802

0.7046	87323.51	73153.84	84973.98	40309.36	34377.38	67056.06	1.119074	0.058025	0.145613	1.731667	0.792162
0.7045	1455.8	902.41	790.63	1080.13	469.92	874.37	0.568922	0.426924	0.554518	1.298801	0.377181
0.7043	42257.8	46445.21	120793.9	67562.23	95497.22	101213.5	0.584281	0.560562	0.673229	0.792729	-0.3351
0.7041	65284.49	62857.45	115251.4	51907.57	66910.82	52553.35	0.821753	0.292892	0.4353	1.420265	0.50616
0.7037	4221.05	8069.61	6778.12	6015.95	7155.75	7323.52	0.339256	0.723724	0.804363	0.930401	-0.10407
0.7036	157977	113675	139064.4	25450.07	25221.86	27439	1.36302	0.012937	0.055505	5.258117	2.394546
0.7032	567.58	336.96	925.12	371	126.16	739.81	0.573647	0.468926	0.592306	1.479147	0.564765
0.7032	19948.85	13427.04	18739.41	9071.34	11538.84	8244.84	1.208121	0.042032	0.117782	1.806109	0.852885
0.703	3231.08	6426.91	2100.05	43282.4	37545.81	41771.89	1.326294	0.000111	0.006218	0.095906	-3.38224
0.7026	828.25	1811.69	1904.52	4464.48	2570.63	2695.52	1.064689	0.086498	0.191784	0.467026	-1.09842
0.7023	281.71	713.97	436.78	11338.45	12271.72	8145.66	1.349172	0.014212	0.058571	0.045109	-4.47045
0.7021	272629.4	249237.8	407221.4	279250.1	255149.8	420822.4	0.084587	0.908756	0.935585	0.972641	-0.04002
0.7021	9721.55	4733.73	7610.22	4834.6	2745.72	3819.71	1.056435	0.118618	0.236098	1.935565	0.952755
0.7013	39829.22	35077.88	41654.17	19028.52	7453	10612.9	1.249466	0.005789	0.035809	3.142286	1.651814
0.7011	2609004	1610350	2161780	2288244	2101783	1766060	0.096018	0.832997	0.882819	1.036557	0.051799
0.7007	359.66	304.94	15690.52	4745.85	5939.62	5605.82	0.691595	0.997065	0.997935	1.003918	0.005641
0.6996	800278.7	736494.9	645536.6	1724706	1787946	1564029	1.352481	0.000569	0.012605	0.42987	-1.21803
0.6995	2945.96	708.34	2835.21	1430.36	1995.65	2058.38	0.003172	0.695542	0.781409	1.183269	0.242778
0.6995	11264.27	10491.43	8725.1	5478.16	9399.16	8181.73	0.877983	0.159528	0.288808	1.321858	0.402568
0.6992	416.15	162.53	679.61	965.09	140.75	318.07	0.02167	0.861026	0.901781	0.883686	-0.17839
0.6988	123533.2	113451.9	93184.94	392555.6	326873.8	338458.7	1.353128	0.002385	0.022717	0.312103	-1.67991
0.6987	51130.79	31008.53	41460.08	56663.84	44748.29	42082.12	0.585788	0.420328	0.550583	0.861354	-0.21532
0.6985	127769.4	95085.78	106933.5	837.14	861.3	1071.88	1.371263	0.00759	0.040398	119.0435	6.895345
0.6984	6133.9	10220.07	8963.92	5101.31	2802.74	13016.6	0.498635	0.693087	0.779706	1.210187	0.275229
0.6982	86469.57	71399.14	37141.95	19724.86	16650.71	11482.76	1.256113	0.07458	0.172646	4.074749	2.026711
0.6973	2532.21	1453.57	1388.33	1944.87	1813.89	1722.56	0.200887	0.932587	0.951564	0.980441	-0.0285

0.6962	8233.32	6974.56	2709.14	3449.21	2201.03	6937.25	0.480963	0.464192	0.589916	1.423399	0.50934
0.6954	69587.71	48126.41	59521.73	556.96	20811.55	16063.72	0.966348	0.005883	0.036092	4.734846	2.243318
0.6951	93659.6	102050.2	81450.82	76244.49	104272.7	99988.55	0.042213	0.921695	0.944828	0.988075	-0.01731
0.6951	335943.2	289199.1	276136.7	181593.7	175680	173402.6	1.337509	0.019407	0.070615	1.698359	0.764142
0.6948	13092.94	10081.95	13185.01	124379.3	129774.3	120845.4	1.368811	0.000103	0.006088	0.09696	-3.36647
0.6947	18793.24	34362.31	20967.15	752.24	2578.94	898.35	1.319873	0.039291	0.112751	17.52504	4.131346
0.6947	10313.34	64574.84	34789.05	26198.58	23235.7	17257.16	0.319476	0.458417	0.584793	1.644547	0.717691
0.6947	2678029	2242267	2654472	2547556	2258000	2188895	0.644685	0.344418	0.485079	1.082968	0.114991
0.6942	112524.5	107650.8	94000.4	100797.4	99665.89	93857.84	0.649305	0.358298	0.498984	1.067459	0.094181
0.6942	16533.11	13877.8	19861.83	1364.61	3366.75	1646.27	1.323552	0.007637	0.04042	7.882668	2.978684
0.6936	1600.47	5773.43	4155.86	46747.43	24550.65	37527.09	1.288764	0.033424	0.101036	0.105948	-3.23858
0.6936	2180.81	3070.05	1785.73	1657.28	2327.62	2277.4	0.352408	0.594048	0.699663	1.123643	0.168184
0.6931	2914.97	3217.97	1498.82	490.25	1010.61	503.8	1.226571	0.059815	0.148398	3.80701	1.928658
0.6928	36121.09	24376.35	28705.92	323626	306688.3	291324.2	1.366088	0.000322	0.009957	0.096788	-3.36903
0.6922	41581.5	1079.58	1080.28	848.57	1318.73	2683.94	0.47186	0.438303	0.565657	9.016532	3.172573
0.692	74721.66	21236.25	30633.22	6567.54	7456.87	4694.58	1.25347	0.160644	0.290317	6.762712	2.757602
v	12468.26	11104.39	25039.22	12664.44	6747.89	15378.72	0.569501	0.430519	0.55778	1.397252	0.482592
0.6915	19550.29	37480.42	44053.04	20469.57	26241.92	34050.75	0.428633	0.473638	0.597047	1.251621	0.323798
0.691	10965.31	3624.78	6274.35	88349.96	79130.3	74113.67	1.330713	0.000573	0.012605	0.086362	-3.53347
0.6906	20775.04	33642.33	18424.45	21289.37	21609.81	25885.41	0.090933	0.806786	0.86415	1.058985	0.082682
0.6901	116700.6	102144.8	188847.8	6874.81	12172.54	8038.34	1.349676	0.041423	0.116944	15.05198	3.911881
0.69	18718.78	299607.1	15310.13	310112.1	17610.82	137678	0.371916	0.746699	0.821312	0.716879	-0.4802
0.6898	42890.39	64769.84	49847.86	62377.93	64665.86	68928.92	0.938989	0.177851	0.311532	0.803725	-0.31523
0.6892	25213.47	33684.86	23450.68	121325.7	104006.4	103674.9	1.352337	0.00099	0.014993	0.250296	-1.9983
0.689	58910.56	62062.23	42705.51	107146.9	124928	97335.16	1.279857	0.006744	0.038427	0.496883	-1.00902
0.689	55387.74	97863.94	112182.1	47215.84	43561.11	96327.38	0.673598	0.339365	0.48029	1.41864	0.504509

0.6887	23544.09	18694.72	22206.93	19838.88	27519.86	25498.51	0.603512	0.370211	0.507798	0.884548	-0.17699
0.6885	75355.47	69701.55	154981.3	197337.6	179132.8	102161.9	0.829097	0.212153	0.351225	0.626866	-0.67377
0.6882	322590.9	315927.4	307662.6	21440.82	32359.69	32040.71	1.364128	1.27E-06	0.001767	11.02245	3.462374
0.6878	265.76	1412.87	2659	2515.41	1745.61	365.88	0.11776	0.922812	0.945098	0.937481	-0.09314
0.6876	19276.52	16595.77	21404.43	17397.78	11492.33	7241.52	0.963073	0.12382	0.243562	1.585224	0.664687
0.6875	13686.19	8272.02	15854.15	106.73	129.82	92.35	1.367054	0.031051	0.095859	114.9661	6.845065
0.6873	94034.91	59730.89	122492.3	352421.2	275788.3	306637.6	1.294461	0.001845	0.019813	0.295512	-1.75871
0.6872	3507.63	2200.2	614.89	129.36	603.05	1303.17	0.821185	0.223554	0.364017	3.106102	1.635105
0.6871	239591.1	204424.4	197262.8	574167.5	527336.2	402939	1.318739	0.024268	0.081247	0.426256	-1.23021
0.6868	65263.92	78973.07	46532.77	2395.69	1381.64	2166.99	1.361076	0.022409	0.077339	32.09278	5.004177
0.6866	27125.18	23736.01	24383.49	62509.11	50691.34	53654.61	1.349204	0.008824	0.044309	0.450958	-1.14893
0.686	36792.98	47817.62	34912.29	8441.09	9045.12	3865.4	1.305788	0.00742	0.039933	5.59784	2.48487
0.6858	1659.35	1417.05	1462.54	583.35	566.54	1061.08	1.201389	0.025696	0.083901	2.052918	1.037676
0.6857	1814.49	2998.31	1101.8	1678.32	11075.09	615.47	0.16431	0.534096	0.649408	0.442416	-1.17653
0.6856	3267907	2831105	2934496	607006.3	518101.1	456685.2	1.36532	0.001213	0.016008	5.710931	2.513726
0.6853	683681.1	590197.5	842727.8	586644.2	735191.4	690027.5	0.243854	0.709392	0.793085	1.052063	0.073221
0.6846	11731.09	10851.24	9013.13	28830.2	30473.9	12060.3	1.052436	0.150666	0.277228	0.442734	-1.17549
0.6843	55305.48	55924.17	45274.91	63353.64	54296.09	60958.89	0.866431	0.172427	0.305533	0.876243	-0.1906
0.6842	23887.15	15154.05	16958.54	35240.51	51841.85	33059.61	1.221094	0.051607	0.134062	0.466113	-1.10125
0.684	440260.9	347390	274153.5	176155.5	174939.2	190118.3	1.262991	0.067452	0.161284	1.961897	0.972249
0.684	7317.17	4504.75	5971.85	40286.56	33909.35	42849.15	1.35457	0.003613	0.028133	0.152025	-2.71762
0.684	920.58	850.49	3313	22799.62	6625.03	15779.94	1.22531	0.099789	0.211156	0.112468	-3.15241
0.6839	21091.99	44058.08	22111.01	10342.32	962.66	9053.15	1.025381	0.081633	0.1838	4.286301	2.099733
0.6839	26550.37	17204.8	15516.65	4479.09	3462.48	1835.88	1.30024	0.035274	0.104742	6.062094	2.599816
0.6837	28278.73	10223.63	13750.36	45692.09	28748.89	34804.55	1.065904	0.063394	0.154777	0.478305	-1.064
0.6834	7238.32	1656.53	5615.4	18789.68	4578.45	3186.33	0.430228	0.511865	0.629577	0.546434	-0.87188

0.6831	46852.65	39744.79	41284.4	50207.29	49941.24	48630.24	1.146553	0.077551	0.177918	0.859544	-0.21836
0.683	2726.1	8388.4	10985.24	4306.83	1761.66	703.31	0.947476	0.159382	0.288808	3.263496	1.706418
0.6826	53106.22	34732.03	49279.58	1048.58	3091.19	2086.16	1.341813	0.015247	0.060807	22.02367	4.460983
0.6824	13493.03	13186.88	13687.62	22240.74	17591.41	12702.26	0.808623	0.278535	0.422308	0.768402	-0.38007
0.6824	20942.87	2350.13	11047.06	13394.45	7562.49	7073.55	0.092407	0.74228	0.81831	1.225097	0.292896
0.6823	36035.66	26407.49	28551.62	2567.09	4446.19	1544.82	1.328458	0.007207	0.039577	10.63259	3.410421
0.6813	253444.7	248578.3	143594.4	300512.2	294482.9	257041.2	0.857303	0.185875	0.320954	0.757735	-0.40024
0.6811	3711.63	10031.78	3484.8	4285.61	2082.06	1736.22	0.861687	0.290609	0.433378	2.125919	1.088086
0.6804	200413.7	265968.9	275066.5	173531.5	200568.6	246124.9	0.729998	0.27146	0.416565	1.195452	0.257556
0.6803	71978.66	75746.72	55187.3	69494.55	67064.98	127088.3	0.607351	0.413983	0.546172	0.769635	-0.37775
0.6796	6532.98	4801.65	14959.62	53801.16	59063.74	57844.72	1.298592	0.000898	0.014717	0.154029	-2.69873
0.6788	16585.28	25657.4	25147.22	20709.23	13186.48	16358.63	0.828369	0.199804	0.337351	1.340977	0.423284
0.6786	14180.42	4999.31	10428.56	13403.8	11843.67	3246.09	0.150292	0.932791	0.951564	1.039122	0.055365
0.6783	7613.89	7481.02	9977.99	12628.91	8974.71	16807.65	0.964891	0.179357	0.313354	0.652749	-0.6154
0.6783	4360.13	6551.97	1707.88	1269.75	20711.11	2585.61	0.082971	0.593644	0.699496	0.513708	-0.96098
0.6778	39741.5	47611.29	39499.14	68652.23	70155.62	53407.85	1.218515	0.03692	0.108666	0.659946	-0.59958
0.6775	186077.4	173596.1	178434.3	314600.1	275574.6	301472.9	1.35246	0.005455	0.034679	0.603498	-0.72858
0.6773	19306.51	11680.89	15974.53	443.31	2077.83	102.09	1.227336	0.016071	0.062714	17.90233	4.162075
0.6771	7590.9	179893.5	59233.5	386805.3	750928.3	443111	1.086158	0.042044	0.117782	0.156067	-2.67976
0.677	4878.81	3041.92	4390.98	13362.16	15700.69	5896.41	1.133134	0.120717	0.239185	0.352173	-1.50564
0.677	59317.55	41275.17	56280.26	25667.08	6799.34	13896.91	1.180711	0.0093	0.045573	3.383557	1.758541
0.6764	25161.99	44261.29	25785.46	27151.58	29701.01	27388.22	0.313275	0.619605	0.721277	1.130197	0.176575
0.6761	94697.55	54944.57	90363.91	78495.64	109768.8	96432.83	0.589743	0.396332	0.5319	0.843022	-0.24636
0.6758	33327.08	28197.52	30141.32	2874.99	1879.66	694.09	1.320714	0.000732	0.013872	16.82332	4.072391
0.6752	81856.05	62507.12	82549.27	209666	74977.75	36267.62	0.130732	0.612455	0.715352	0.707088	-0.50004
0.6749	4098.83	2944.68	5904.63	7924.14	4101.57	3080.32	0.221073	0.700077	0.785087	0.85715	-0.22238
0.6745	6444.05	10198.31	13626.89	82940.26	70217.33	64699.17	1.338058	0.003244	0.026783	0.138941	-2.84746

0.6744	20628.99	34010.8	30866.76	355.55	7527.5	7164.29	1.034299	0.012909	0.055476	5.682503	2.506526
0.6736	9851.75	8827.99	8216.71	39308.84	10904.11	14179.98	0.919981	0.298101	0.440781	0.417693	-1.25949
0.6734	41203.3	42335.22	43516.85	20110.33	24646.18	15897.59	1.300786	0.009082	0.044935	2.094753	1.06678
0.6731	395289.1	202142.8	249276	1069.49	988.86	66025.26	1.140281	0.034132	0.102536	12.43629	3.636485
0.6731	58.5	62.6	47043.63	28416.11	22515.55	34144.2	0.906632	0.506707	0.624643	0.554384	-0.85104
0.6729	28094.86	25951.41	29572.14	1522.64	2197.68	2881.43	1.358217	0.000513	0.012047	12.6661	3.6629
0.6726	454835.6	355567.9	432861.1	3941679	3484037	2602502	1.35977	0.017077	0.065143	0.123977	-3.01186
0.6725	1171.61	1327.35	5832.5	6129.51	3580.13	4844.27	0.830575	0.311975	0.454663	0.572455	-0.80477
0.6719	331283.4	392221.5	323560.6	182181.9	3832.46	1126.74	1.049416	0.029521	0.092844	5.595058	2.484153
0.6719	2036.91	17503.51	3075.84	8774.74	4082.55	2552.25	0.056039	0.687737	0.776241	1.467679	0.553537
0.6718	7668.79	3249.46	3294.73	24778.8	8617.47	26163.56	1.149563	0.107129	0.221526	0.238634	-2.06713
0.6715	11605.7	9855.4	8857.47	4561.39	5329.8	6035.92	1.294965	0.012813	0.055229	1.903583	0.928717
0.6713	42599.3	45927.02	41682.52	1840.47	4496.95	4051.15	1.341088	4.94E-05	0.004503	12.53386	3.647758
0.6711	777.02	106.89	3632.36	462.4	1732.59	2787.54	0.381321	0.90979	0.936	0.906421	-0.14175
0.6706	13464.31	14584.42	18147.69	18426.32	16982.99	13665.09	0.319476	0.655745	0.747686	0.941355	-0.08719
0.6705	43013.47	63626.98	50091.5	12576.85	8704.07	15115.59	1.325747	0.015386	0.061135	4.306236	2.106427
0.6702	9009.63	10995.44	8060.14	2289.6	2299.04	3137.22	1.342285	0.010072	0.047952	3.632632	1.861015
0.6697	457.88	1045.38	397.19	316.15	1612.57	1198.64	0.434735	0.414533	0.546566	0.607685	-0.7186
0.6697	7036.82	10143.74	7767.19	19574.06	18746.89	17059.52	1.319007	0.001327	0.016575	0.450479	-1.15047
0.6697	51709.53	38007.71	30228.38	165967.3	123172.5	101719.2	1.293544	0.030884	0.095478	0.306877	-1.70427
0.6697	9888.78	14841.94	10477.67	24508.11	1009.2	14065.11	0.3332	0.852119	0.895897	0.889496	-0.16894
0.6694	165820.2	117822.4	104451.3	214627.8	193082.1	182604.8	1.141229	0.049184	0.130488	0.657436	-0.60508
0.6693	52737.91	16699.68	47090.52	102.04	15960.31	16742.13	0.790299	0.113904	0.230506	3.552201	1.828713
0.6692	7019.28	7363.74	14033.94	16330.65	27919.74	32550.39	1.172915	0.060431	0.149499	0.370009	-1.43437
0.6691	42088.02	33670.17	44068.5	80623.23	67402.84	78348.51	1.310221	0.002906	0.025269	0.529329	-0.91776
0.669	14563.62	18718.23	10846.46	13225.87	15563.88	6771.34	0.530024	0.458365	0.584793	1.240916	0.311405

0.6685	20881.1	10973.38	13940.45	282.76	1943.98	765.12	1.276713	0.036665	0.108135	15.30651	3.936073
0.6682	1260.17	1844.1	3653.49	690.01	680.71	1011.1	1.124441	0.177662	0.311501	2.837225	1.504481
0.668	54739.68	65840.05	38118.04	2973.16	7648.59	7348.86	1.310514	0.024948	0.082765	8.830962	3.142571
0.6675	540	1106.57	694.52	1902.76	226.54	1011.84	0.016265	0.645843	0.740536	0.745299	-0.42411
0.6675	264.82	2242.72	330.8	20005.95	18378.3	13838.74	1.272183	0.006893	0.038918	0.05435	-4.20157
0.6673	3801.17	6023.16	2213.79	46307.23	44080.74	41603.5	1.336725	3.03E-05	0.003892	0.091204	-3.45476
0.6666	80682.36	113198.6	92354.1	316602.7	58103.64	296080.1	0.663213	0.261437	0.407399	0.426716	-1.22865
0.6663	7816.88	148.73	1210.23	4828.58	2470.88	1491.56	0.458547	0.964048	0.973652	1.043774	0.06181
0.6662	2729.33	3184.14	597.11	3774.19	881.12	457.89	0.331728	0.741692	0.817868	1.273289	0.34856
0.6661	42732.27	40946.45	38896.76	251020.5	226678.3	220272.6	1.370835	0.002112	0.021043	0.175617	-2.5095
0.6659	101266	110560.7	119637	24575.38	13036.91	23971.12	1.332958	0.000298	0.009591	5.382352	2.428237
0.6654	7910.32	6819.86	11022.79	22076.35	16169.32	14163.78	1.205891	0.044628	0.122426	0.49138	-1.02509
0.6649	17286.96	18062.95	13703.48	21807.22	21677.05	34277.66	1.080715	0.1391	0.26352	0.630815	-0.66471
0.6647	15474.61	17432.51	22770.48	980.46	3517.09	1308.08	1.301651	0.009712	0.046882	9.590277	3.261572
0.6646	562568	467022.1	540981.1	2462878	2230483	2213184	1.367428	0.000713	0.013717	0.227403	-2.13667
0.6641	849.5	5014.4	225.07	8937.66	4478.88	2222.29	0.835667	0.273166	0.417956	0.389349	-1.36086
0.6635	14825.03	11182.69	14025.73	15521.53	7960.68	7518.9	0.72133	0.372033	0.509325	1.291355	0.368886
0.6632	132.5	77.9	3068.17	351.76	494.57	96.01	0.118295	0.513718	0.630748	3.479179	1.798747
0.6629	48423.74	47887.75	44819.26	15777.42	13697.28	11206.51	1.355193	5.14E-05	0.004503	3.469188	1.794598
0.6627	51542.19	43908.2	45589.89	26601.22	28193.57	18370.82	1.263737	0.005065	0.033561	1.927685	0.94687
0.6622	840.43	611.07	660.93	721.54	228.74	621.46	0.647447	0.360934	0.500654	1.344007	0.426541
0.6618	269008.1	228043.5	239306.8	1883702	1796152	2081517	1.370235	0.002127	0.021071	0.12781	-2.96793
0.6617	21880.43	59311.26	64704.26	40873.57	66403.4	22814.07	0.150227	0.789701	0.852056	1.121491	0.165418
0.6609	18974.1	17216.25	16772.01	35946.9	34181.61	31696.94	1.355663	0.001191	0.015971	0.520129	-0.94306
0.6596	167287.3	115101.4	151286	108009.2	125752.3	116682.3	0.885865	0.207307	0.346492	1.237501	0.30743
0.6595	29246.52	53866.08	10601.7	8386.12	5643.18	8513.71	1.07463	0.198196	0.335509	4.157133	2.055589
0.6589	2632509	2200987	2118279	24798.36	24258.83	23385.47	1.372278	0.004798	0.032564	95.96245	6.584398

0.6588	46129.97	34317.49	4943.22	239120.6	12481.49	5016.01	0.108414	0.536249	0.65148	0.332754	-1.58747
0.6588	802.84	1030.6	802.75	1564.28	2363.7	91.72	0.150018	0.560638	0.673229	0.655818	-0.60863
0.6584	4987.41	4593.33	81.24	1617.3	206.02	315.88	0.417383	0.24806	0.392342	4.516632	2.175247
0.6583	145424.3	118283.1	108910.7	29249.76	136.8	301.88	1.12294	0.001533	0.017791	12.55095	3.649725
0.6578	3450.28	12362.32	2554.16	4171.8	8067.66	641.13	0.394794	0.658152	0.74977	1.425925	0.511898
0.6574	39811.21	22240.28	21339.51	13350.46	15175.05	6767.46	1.10604	0.100841	0.2122	2.362822	1.240511
0.657	500.03	95.53	633.49	1443.84	589.8	433.95	0.704872	0.326987	0.46917	0.498077	-1.00556
0.6566	611.58	704	298.8	759.57	4723.23	529.07	0.72111	0.394218	0.530369	0.268532	-1.89683
0.6564	14977.06	11776.78	15040.97	33770.09	32447.51	33048.14	1.349349	0.001252	0.016101	0.42104	-1.24797
0.6563	3420.64	3273.81	1744.44	6103.17	3221.07	4127.69	0.876732	0.185038	0.320198	0.627337	-0.67269
0.6561	14827.41	15474.68	11760.68	48545.62	68684.52	35043.74	1.300822	0.062193	0.152646	0.276231	-1.85605
0.6556	211889.1	173479.4	206031.7	200412.4	190911.8	184754	0.233136	0.720399	0.802099	1.026597	0.03787
0.6554	17334.59	10752.55	6463.44	12963.35	4801.26	7085.2	0.523566	0.465756	0.590698	1.390376	0.475475
0.6552	5339.4	14604.53	6354.4	7500.02	10959.81	9767.27	0.325359	0.85178	0.895756	0.93167	-0.10211
0.6552	127755.1	111633.2	124007.5	16998.49	14429.69	79620.61	1.110238	0.052331	0.13562	3.272397	1.710348
0.6551	42532.8	32637.88	34618.09	13088.65	10274.34	13253.09	1.343511	0.00954	0.046412	2.998376	1.584181
0.655	1253.56	3425.96	1477.74	16691.19	6268.91	4880.16	1.141753	0.188595	0.323655	0.221164	-2.17681
0.6537	42226.18	38477.01	34460.92	97599.3	87402.54	74166.23	1.333458	0.012629	0.054611	0.444361	-1.1702
0.6534	444390.9	270851	456318.1	25648.39	24597.18	24618.29	1.362401	0.025838	0.084177	15.64921	3.968018
0.6533	16979.07	8517.08	6963.3	3114.86	2499.65	7467.93	1.017537	0.162283	0.292189	2.481146	1.311007
0.6533	854.23	1122.17	1962.34	340.63	2016.74	800.12	0.465384	0.690326	0.778219	1.247428	0.318956
0.6521	9492.4	10952.71	13187.81	42645.66	46093.29	44985.88	1.359309	2.35E-05	0.003778	0.251508	-1.99132
0.652	299867.3	232224	264988.2	1076156	1008386	616189	1.307769	0.045165	0.123038	0.295135	-1.76055
0.6512	684127	399816.4	632702.9	1230099	1125928	1057961	1.234477	0.009514	0.046338	0.502827	-0.99187
0.6507	35420.64	112766.4	43035.94	63344.53	63528.06	68373.2	0.293118	0.961548	0.972095	0.979396	-0.03004
0.6504	363391.9	271384.1	286320.4	322235.3	358392.3	321353.8	0.586385	0.454755	0.581654	0.919275	-0.12143

0.6495	1624.23	1524.79	199.26	996.4	22.79	63.84	0.813531	0.255979	0.401805	3.091586	1.628347
0.6494	652519.5	511569.1	608305.8	343339.2	371704.1	332416.6	1.313199	0.021797	0.076035	1.692088	0.758805
0.649	13552.31	10034.92	5487.28	19683.08	20066.89	12202	0.97026	0.093119	0.202246	0.559642	-0.83742
0.649	31466.84	28556.62	30815.41	4949.63	2670.84	3296.11	1.353596	3.03E-05	0.003892	8.321184	3.056789
0.6489	12218.76	11832.92	7498.68	24277.98	23143.24	14908.09	1.151759	0.054017	0.138329	0.506188	-0.98225
0.6487	317.71	2370.04	676.15	3503.82	1445.16	2716.13	0.891825	0.175647	0.309651	0.438859	-1.18817
0.6485	7761198	6822824	7439098	19839382	15580304	16426582	1.347676	0.013687	0.057351	0.424777	-1.23522
0.6484	38707.25	16773	12731.52	20820.05	20934.07	4296.51	0.506089	0.497242	0.615936	1.481234	0.5668
0.6481	42831.08	34550.86	57795.82	47559.49	110944	44568.55	0.625112	0.408518	0.540598	0.665664	-0.58713
0.6476	53285.59	44820.99	33772.56	35704.02	44467.33	25359.17	0.660857	0.328593	0.470192	1.249678	0.321556
0.6474	11452.17	9081.13	12077.8	19939.1	13069.66	20864.96	1.120527	0.088183	0.194627	0.605325	-0.72422
0.6474	3657.73	9168.64	4405.84	1145.79	5009.04	3375.31	0.745765	0.290443	0.43328	1.80818	0.854538
0.6467	1881.67	1473.44	330.57	682.4	3120.79	3543.86	0.578975	0.311337	0.45421	0.501654	-0.99523
0.6467	216059	178998	186753.9	52853.29	12940.93	36564.43	1.252162	0.000591	0.012746	5.684042	2.506917
0.646	52101.58	27592.89	68876.31	57573.29	39333.54	34941.55	0.165788	0.712628	0.79548	1.126831	0.172271
0.6454	33780.21	38220.47	31127.21	5661.91	13937.46	13284.31	1.23156	0.002779	0.024726	3.136142	1.648991
0.645	19792.06	28094.5	15236.49	10257.98	7744.93	5362.89	1.219138	0.057811	0.145411	2.701515	1.433768
0.6449	1205.83	731.44	1748.21	5086.95	4450.23	5101.22	1.292893	0.000855	0.01464	0.251768	-1.98983
0.6448	53974.43	46043.64	46893.47	49675.48	21923.77	54763.49	0.486644	0.575066	0.684146	1.162618	0.217377
0.6447	20405.24	26139.32	21353.37	77817.35	83265.69	69353.99	1.357354	0.001786	0.019432	0.294649	-1.76293
0.6447	4719.2	4921.59	6296.89	8965.47	13830.26	8203.33	1.191697	0.094886	0.204789	0.514134	-0.95978
0.6444	12257.35	6873.43	12269.1	5313.65	6058.86	10850.05	0.721933	0.287876	0.430924	1.412973	0.498734
0.6443	47147.3	30369.93	37327.65	5952.61	361.19	1803.05	1.219835	0.011323	0.051196	14.14895	3.822623
0.6443	115915.1	104888.6	121776.6	9733.26	1338.1	1670.48	1.300587	0.000231	0.009171	26.88625	4.748796
0.6438	938810.8	1152321	783236.5	937651.2	825000.4	682659.3	0.65668	0.340043	0.480781	1.175461	0.233227
0.6437	60866.77	35985.09	39870.5	4811.21	285.3	7242.69	1.135272	0.026602	0.086277	11.08033	3.469928

0.6436	74460.71	103614.6	49061.63	179872.7	221653.7	147767.2	1.209686	0.018412	0.068362	0.413507	-1.27402
0.6434	12017.87	215.94	10100.36	100068.3	91725.17	87295	1.091663	8.22E-05	0.00568	0.080025	-3.6434
0.6432	8764151	6623167	7594579	6958285	6793856	6063500	0.848054	0.224735	0.364986	1.159786	0.213858
0.6432	12894.25	12477.34	10562.02	14491.02	13500.91	14210.68	1.075297	0.084754	0.18878	0.851455	-0.232
0.6431	1258.74	404.07	241.88	1749.45	675.14	963.78	0.75778	0.33351	0.475088	0.562126	-0.83104
0.6429	119542.8	86457.34	102447.1	35063.37	21178.26	28789.16	1.326425	0.007954	0.04162	3.627476	1.858966
0.6423	79505.63	281457.3	154543.4	55398.71	78523.06	58354.48	1.033493	0.207546	0.346555	2.681071	1.422809
0.6422	37334.86	32141.32	2006.09	28555.24	3997.79	7764.34	0.202549	0.486465	0.608061	1.772989	0.826184
0.6418	44582.62	24028.71	39449.9	10838.13	7043.37	25980.79	1.051254	0.065006	0.157511	2.463648	1.300796
0.6418	184458.1	289956.1	293244.9	56206.43	101737	133746.4	1.181263	0.026889	0.087144	2.631765	1.396031
0.6414	8631.44	2646.71	9557.47	14950.16	9201.96	7455.19	0.669658	0.315815	0.458116	0.659203	-0.60121
0.6411	44685.9	30531.26	41460.4	17611.83	11967.37	14640.33	1.302918	0.01948	0.070703	2.638598	1.399772
0.6411	11708.32	14022.44	13864.54	10094.08	11770.13	11533.13	1.023492	0.093859	0.203246	1.185582	0.245596
0.6408	40622.74	10508.67	30338.13	311.98	12634.07	9496.17	0.839733	0.1431	0.267818	3.630191	1.860045
0.6406	86595.67	81437.27	73811.28	89430.45	64859.33	57583.24	0.643813	0.414957	0.546629	1.141458	0.190878
0.6404	46454.56	24557.78	40941.41	6030.36	10935.71	8707.32	1.293243	0.043146	0.119782	4.360692	2.124557
0.6401	24956.44	11232.68	10361.44	63288.19	24022.98	33983.53	1.055956	0.157425	0.286282	0.383781	-1.38165
0.64	1305.88	3217.49	3371.87	3053.42	12619.62	14943.08	0.992484	0.169135	0.301292	0.257879	-1.95524
0.6398	139884.2	115428.7	87964.36	30832.44	33875.34	21762.43	1.323228	0.024071	0.080898	3.96989	1.989099
0.6397	177813.4	118434.6	166812.8	167020.9	19053.32	6750.43	0.914244	0.215784	0.35575	2.401461	1.263912
0.6396	17371.88	21357.62	27943.69	375155.7	348949.5	333940	1.365675	0.000719	0.013717	0.063015	-3.98815
0.6394	17374.28	16111.98	12505.42	31462.59	32652.74	32382.4	1.327032	0.005159	0.033718	0.476609	-1.06912
0.6383	343236.5	300330	293008.2	153325.1	137473.5	133182.8	1.354742	0.003681	0.028397	2.208999	1.143393
0.6383	9982413	8395771	9156980	15418978	13892673	13156942	1.309363	0.005198	0.033818	0.648365	-0.62512
0.6382	580.59	959.26	1477.39	1258.43	529.25	942.76	0.167249	0.790009	0.852178	1.105038	0.144096
0.6382	80216.6	67877.9	53509.18	86394.9	28059.46	43544.04	0.601286	0.506034	0.624166	1.275986	0.351612
0.6382	55608.54	56773.75	52812.93	39937.53	29649.83	24946.42	1.23714	0.027004	0.087386	1.747473	0.80527

0.6379	281656.8	490779.9	295806.4	78802.28	62889.75	79825.91	1.331791	0.05181	0.134347	4.822377	2.269745
0.6375	18286.83	35693.69	95591.17	53593.04	26621.73	18495.39	0.335533	0.559901	0.673086	1.515261	0.599567
0.6374	2310	2932.44	2857.13	6231.57	6871.86	6636.72	1.350999	0.00014	0.006718	0.410309	-1.28522
0.6372	18389.11	10076.4	14623.9	1754.27	1405.26	1262.48	1.35187	0.032643	0.099575	9.744304	3.284559
0.637	53619.69	67515.62	71588.44	6915.92	8716.74	6632.03	1.364282	0.008317	0.042805	8.656027	3.113705
0.637	670741.2	527106.2	517517.7	408454.7	378055.7	374724.4	1.247776	0.059156	0.147518	1.477191	0.562856
0.6368	14468.44	12279.12	12759.31	64169.42	53937.17	48557.99	1.360936	0.010259	0.048573	0.237044	-2.07677
0.6367	9696.17	9507.68	8750.01	37008.5	34725.7	31014.9	1.367016	0.004027	0.029577	0.272059	-1.87801
0.6363	211328.7	188121.7	186284.5	332783.1	396945.1	316530.6	1.323562	0.016905	0.064754	0.559837	-0.83692
0.6361	10661.76	5238.86	9904.35	1427.38	2449.71	9848.75	0.82209	0.281287	0.425149	1.880028	0.910755
0.6359	5622.47	8845.79	434.3	6865.65	4151.16	470.74	0.086795	0.731076	0.810646	1.297279	0.375489
0.6358	42400.49	53457.14	35492.26	4564.67	1769.23	2114.25	1.340262	0.013965	0.058128	15.54777	3.958636
0.6354	16065	10621.95	2241.61	7003.64	13492.97	9751.21	0.313474	0.927662	0.94833	0.956385	-0.06434
0.6346	62724.88	83267.11	52373.27	22017.02	31576.11	3965.83	1.044599	0.018613	0.068858	3.446297	1.785047
0.6346	30785.69	38508.61	23435.48	21393.11	23279.33	4746.86	0.835353	0.126008	0.246198	1.876388	0.907958
0.6345	8137.45	5107.14	6032.33	3880.48	879.49	2271.55	1.080212	0.030766	0.095236	2.741501	1.454966
0.6336	120.81	1797.38	628.61	1672.18	535.25	190.76	0.049474	0.944512	0.960599	1.061968	0.08674
0.6335	45931.18	37084.97	41622.83	22622.63	20424.13	20179.94	1.343157	0.010077	0.047952	1.971303	0.97915
0.6334	1861.03	1376.69	869.15	7823.68	8186.17	8169.26	1.334278	0.000458	0.011221	0.169852	-2.55765
0.6331	12041.44	1523.51	2370.46	144.45	439.34	1666.86	0.997709	0.308216	0.451449	7.080359	2.823823
0.6324	94779.08	131310.7	155683.8	42439.56	72496.15	54943.82	1.217045	0.039021	0.112432	2.247319	1.168205
0.6323	19536.28	10885.69	14781.16	33493.23	16220.61	12969.76	0.514168	0.465601	0.590674	0.721132	-0.47167
0.6323	213.16	633.42	591.02	151.83	48.28	131.95	1.133168	0.103024	0.21553	4.329338	2.114146
0.6318	8432.71	5038.22	624.96	2406.11	4239.11	2447.29	0.015594	0.54174	0.656687	1.550275	0.632524
0.6315	62227.98	79138.96	77583.72	57097.99	49167.69	41317.16	1.173185	0.029304	0.092362	1.483578	0.569081
0.6314	6390.07	4010.55	2851.05	18666.68	20516.1	28115.03	1.308963	0.015966	0.062461	0.196911	-2.34439

0.6312	2070.27	1182.06	2507.08	2632.77	805.61	552.79	0.643222	0.491709	0.610666	1.443038	0.529109
0.6311	1841076	1753009	1725551	1035540	1299806	830804.2	1.233718	0.027725	0.088994	1.680159	0.748598
0.6306	12328.39	8600.74	11812.09	51534.54	48801.83	42363.4	1.352868	0.001824	0.019636	0.229441	-2.1238
0.6306	53850.51	261363.4	223481.1	29125.64	45304.27	30271.06	1.116196	0.150633	0.277228	5.145081	2.363194
0.63	37411.77	7194.88	27815.99	36780.24	28275.94	24207.05	0.495511	0.606295	0.710061	0.811338	-0.30163
0.63	240.43	284.34	614.58	622.08	1790.38	68.98	0.136159	0.473258	0.597047	0.459149	-1.12297
0.6298	18289.42	19249.3	17650.65	14624.87	14178.15	15082.82	1.325356	0.004951	0.033105	1.257567	0.330635
0.6295	89165.31	65159.23	76521.97	100768.2	104017.8	99662.52	1.155454	0.066744	0.160296	0.758245	-0.39926
0.6295	38540.27	29703.48	32097.34	5838.51	13790.94	4999.64	1.260276	0.002831	0.02493	4.074088	2.026477
0.6288	10762.32	3549.5	5318.17	2089.89	3771.82	792.29	0.989203	0.17444	0.308098	2.950104	1.560766
0.6287	2040.34	1474.77	3315.37	7962.34	9826.8	10578.94	1.299698	0.002524	0.023404	0.24078	-2.05421
0.6286	162.26	763.56	615.05	305.22	1399.29	916.8	0.521965	0.392307	0.528614	0.587824	-0.76654
0.6285	22862.34	22222.44	37233.8	25428.16	25933.35	26876.3	0.089842	0.807756	0.864687	1.052159	0.073352
0.6279	930.96	2319.6	3218.13	5414.45	4146.73	3526.73	0.986322	0.065545	0.15864	0.494249	-1.01669
0.6277	2272.56	2440.59	3548.22	859.61	789.98	627.49	1.328735	0.034737	0.103608	3.628054	1.859196
0.6272	15358.98	7680.17	20517.55	98362.22	75765.27	73241.63	1.299939	0.00554	0.034797	0.17608	-2.5057
0.6263	111202	85140.03	90335.12	10957.63	17472.23	14123.12	1.35462	0.006938	0.039073	6.736947	2.752095
0.6263	5577.31	5142.08	5758.76	3628.58	4373.4	995.56	0.882737	0.131313	0.253182	1.831406	0.872952
0.6261	18266486	23419478	16478608	7247959	7197588	9433013	1.311362	0.021742	0.076035	2.435849	1.284425
0.6259	325967.5	281719.5	355633.1	188725.1	172214.6	169845	1.330676	0.01597	0.062461	1.814898	0.859889
0.6258	137568.4	129508.1	140855.8	377837.2	173432.6	111140.4	0.60816	0.40276	0.536726	0.61583	-0.6994
0.6257	112750.8	108829.2	119831.1	40107.13	27265.46	9896.83	1.223202	0.004885	0.03291	4.418451	2.143541
0.6256	775992	753311.6	720235.1	580991.9	272457.8	69868.39	0.960933	0.095156	0.205075	2.436364	1.28473
0.6256	2070.87	170.73	1452.75	949.5	1354.87	1296.06	0.332093	0.96103	0.972095	1.026086	0.037151
0.6253	77433.16	60030.25	75590.97	38018.76	39754.39	58740.38	1.124871	0.04347	0.120384	1.560683	0.642178
0.6251	351.19	2383.02	499.99	1957.02	2012.26	611.26	0.522853	0.607327	0.710806	0.706074	-0.50211

0.6247	4998.16	10738.18	16722.52	14090.11	10076.12	6893.94	0.046454	0.913339	0.93853	1.045032	0.063547
0.6244	576622.4	531037.7	540928.8	407624.5	413381.7	384655.7	1.342784	0.001765	0.019432	1.367373	0.451406
0.6242	110875.4	81035.71	37797.15	48722.62	76344.31	48313.05	0.392998	0.481896	0.604311	1.324883	0.405865
0.6241	5271.04	4712.6	3654.59	2852.55	2081.13	5043.65	0.754037	0.310502	0.45373	1.366922	0.450931
0.6235	1299.12	1389.2	370.24	643.11	3032.5	1787.48	0.55434	0.374305	0.511793	0.559859	-0.83686
0.6231	8211.25	8238.53	9947.53	597.21	1692.17	2751.62	1.22875	0.001125	0.015971	5.236523	2.388609
0.6218	53438.58	69108.37	56994.18	991.9	6028.7	10753.99	1.209857	0.001602	0.018328	10.101	3.336426
0.6217	42031.17	31394.38	37867.25	37957.34	38599.29	40607.85	0.432594	0.596126	0.701163	0.949885	-0.07418
0.6212	3730.7	7548.35	1974.89	1405.61	4687.82	7944.88	0.019811	0.921956	0.944828	0.944126	-0.08295
0.6207	26007.04	19542.13	23209.4	3386.35	8315.99	1557.74	1.214873	0.002605	0.023753	5.185381	2.37445
0.6206	66056.28	90389.18	61828.04	150533.7	133072	141735.9	1.29184	0.005608	0.03507	0.513172	-0.96248
0.6205	13067.48	31419.56	25037.32	54864.97	36288.41	24787.61	0.822011	0.220843	0.361358	0.599653	-0.7378
0.6183	1181334	863669	1140040	846807.6	1529993	610959	0.300269	0.839412	0.887873	1.06603	0.092249
0.6182	1277199	1107533	1319199	660793.2	591599.2	572881.5	1.347772	0.004638	0.032268	2.029246	1.020944
0.6181	19349.2	21601.46	14944.69	2172.83	2166.33	2788.73	1.360585	0.013398	0.056592	7.841781	2.971181
0.618	18094.79	28461.85	20351.06	22813.26	17922.58	21194.11	0.278427	0.66656	0.757439	1.080377	0.111535
0.6175	1925.83	838.3	3357.04	2298.8	780.31	2370.53	0.102672	0.816061	0.871224	1.123225	0.167647
0.6173	82131.21	58688.17	66805.99	35442.26	66322.78	68308.67	0.605624	0.387687	0.524334	1.220796	0.287823
0.6173	6556.74	4736.52	343.8	10357.63	283.15	5505.96	0.065775	0.688877	0.777126	0.720706	-0.47252
0.6166	34939.56	21807.17	33382.59	26722.06	32115.88	30617.87	0.046827	0.963343	0.973617	1.007529	0.010821
0.6166	12158.36	16192.2	13386.95	30339.49	20622.66	13511.61	0.844948	0.257329	0.402763	0.647357	-0.62737
0.6154	278664.3	426344.7	281113.8	156691.1	134457.4	127316	1.289788	0.056401	0.142688	2.356526	1.236662
0.6151	5552.58	11368.7	11618.63	20660.78	17681.18	21383.88	1.159425	0.017461	0.066068	0.477849	-1.06537
0.615	649.58	303.66	7331.49	4719.76	3453	4973.39	0.781701	0.554536	0.669219	0.630202	-0.66611
0.6146	798397.7	520860	704739.8	763326.9	1422065	789791.9	0.824331	0.276424	0.420569	0.680293	-0.55577
0.6144	85789.15	15339.4	14997.28	8996.27	10157.16	7107.79	0.954701	0.331079	0.472552	4.421951	2.144683

0.6144	34656.35	18099.15	23512.56	3682.39	4505.36	4815.48	1.335962	0.048868	0.129866	5.865317	2.552209
0.6139	2438.5	3660.08	3710.3	4483.75	6962.56	4520.83	1.056794	0.112819	0.229053	0.614317	-0.70295
0.6134	180.65	100.9	206.55	155.42	90.91	318.27	0.095634	0.756638	0.829106	0.864506	-0.21005
0.6134	450076.2	286768	449945.9	401001.7	335893.2	490852.3	0.158525	0.856331	0.899024	0.96664	-0.04895
0.6132	80141.79	71724.29	94974.11	24435.51	21855.08	21993.22	1.359988	0.011833	0.052522	3.614915	1.853962
0.6129	17426.39	21661.66	24801.52	167.85	5701.74	281.38	1.159572	0.002598	0.023737	10.38691	3.376695
0.6128	2235.12	733.37	2726.51	3930.79	6333.33	4351.95	1.063542	0.03762	0.110205	0.38964	-1.35979
0.6123	67741	53842.67	53150.02	138540.4	149410.6	109211	1.314981	0.015163	0.060748	0.439956	-1.18457
0.612	2710.23	2412.22	3237.84	3795.49	1804.32	6573.07	0.426938	0.456093	0.582852	0.686796	-0.54205
0.6117	16043.03	13648.19	7947.92	9309.79	5299.31	15518.63	0.468469	0.549571	0.6647	1.249319	0.321142
0.6109	6234.33	4617.13	2962.15	3273.89	1720.41	3247.62	0.885539	0.179961	0.314032	1.676018	0.745038
0.6108	19962.45	12140.79	3115.41	30954.77	34309.89	24250.12	0.991572	0.043983	0.121282	0.393439	-1.34579
0.6105	315819.2	274473	280630.7	86516.27	62593.99	56374.2	1.349849	0.000278	0.009591	4.238388	2.083516
0.6095	9236.24	15948.7	10725.05	44551.62	41445.3	24219	1.25053	0.048405	0.129148	0.325815	-1.61788
0.609	6389.15	5490.67	5777.89	3163.04	1750.85	544.1	1.104326	0.023042	0.078797	3.235204	1.693857
0.608	249.11	5573.02	196.14	7035.6	4736.8	4776.53	0.960046	0.177896	0.311532	0.363665	-1.45932
0.6078	24755.38	37632.22	26875.15	80088.47	64082.87	20523.24	0.654589	0.291438	0.434169	0.54199	-0.88366
0.6063	5675.49	3975.73	2724.82	5029.48	5376.6	4318.18	0.597903	0.463689	0.589615	0.84052	-0.25065
0.6061	112186.9	95612.71	105202.8	270461.4	245711.2	243795.8	1.361634	0.000485	0.01162	0.411862	-1.27977
0.6059	1064.27	25.11	1295.04	15.63	27.51	21.67	0.998498	0.186289	0.32147	36.79093	5.201278
0.6053	47440.3	12289.31	48659.69	4180.47	8819.23	7051.22	1.152918	0.130499	0.252709	5.405702	2.434482
0.6045	187439.3	135960.5	198865.8	2033.55	6584.69	2934.51	1.349456	0.012337	0.053993	45.20704	5.498476
0.6044	31988.64	55073.97	34972.59	22276.64	4672.28	7105.53	1.139624	0.035605	0.105579	3.583532	1.841382
0.6043	81.68	119.47	7.45	1176.23	1662.78	1065.68	1.221844	0.018827	0.069351	0.053423	-4.2264
0.6041	37007.4	41777.89	36163.63	56502.33	71323.86	46038.6	1.140022	0.108714	0.224058	0.66114	-0.59697
0.6039	57099.53	62216.25	76246.09	92052.93	88839.31	91575.56	1.222243	0.042789	0.119112	0.717743	-0.47846

0.6036	124620.8	8723.97	95431.2	115151.2	69631.07	49778.02	0.339012	0.964308	0.973652	0.97534	-0.03602
0.6035	10570.82	22909.68	16127.47	6096.91	5979.57	560.3	1.000897	0.054821	0.139921	3.925681	1.972943
0.6035	14166.61	15398.05	20926.57	11726.81	11189.83	12997.17	1.08786	0.136967	0.260716	1.4059	0.491494
0.6033	2670.34	972.38	662.11	182.73	1856.06	1905.36	0.247903	0.893511	0.924694	1.091447	0.126242
0.6028	5845.59	2080.81	4509.39	5253.12	6356.87	3745.2	0.505668	0.511822	0.629577	0.809875	-0.30423
0.6022	529834.7	551932.7	457105.8	536055.7	516275.7	504122	0.163032	0.860895	0.901781	0.988705	-0.01639
0.6022	6961.62	8623.06	8751.54	19007.3	15046.22	10105.89	1.124258	0.117933	0.235489	0.551099	-0.85962
0.6019	512603.5	462831.5	425836.7	612624.9	527504	537702	1.078091	0.066528	0.159926	0.835169	-0.25986
0.6012	2589.07	4451	3292.47	6182.22	3423.71	8687.39	0.881597	0.216426	0.356356	0.564826	-0.82412
0.6009	147006.1	164244.5	116424.8	1502.48	5870.54	6215.16	1.327441	0.009464	0.046198	31.47407	4.976092
0.6009	11805.38	15427.58	7905.1	4586.74	3991.02	9131.58	1.022006	0.104323	0.217684	1.984154	0.988524
0.6005	6561	1284.08	18290.94	41728.1	1981.7	204.29	0.288486	0.713932	0.796323	0.595163	-0.74864
0.6005	17524.36	16133.86	21362.48	117845.5	116949.2	18513.9	0.972068	0.182531	0.317752	0.217208	-2.20285
0.6004	493.84	533.08	1535.33	122.02	1805.18	4948.23	0.193853	0.417792	0.548303	0.372668	-1.42404
0.6003	7968.45	3589.65	4110.96	2683.53	3424.24	3696.45	0.844696	0.290736	0.43342	1.598195	0.676444
0.6002	133164.7	2979.72	125817.6	14428.67	126374.5	96455.43	0.199675	0.886289	0.920499	1.104121	0.142898
0.5998	150646.2	194721.5	124371.6	266962.5	241035.3	230089.7	1.184158	0.030105	0.093869	0.636428	-0.65193
0.5991	6014.14	4696.68	6546.65	30678.77	44922.42	45280.68	1.354177	0.017788	0.066844	0.142763	-2.80831
0.5983	9629.02	14087.17	26317.85	5309.42	3978.1	4072.85	1.231332	0.132874	0.255232	3.744959	1.90495
0.5983	1234.01	4692.66	3506.2	10597.55	4740.69	9053.9	1.011895	0.08524	0.189475	0.386718	-1.37065
0.5978	51987.48	67512.84	37789.86	223627.4	282427.4	199197.6	1.329239	0.010934	0.050063	0.223027	-2.16471
0.5972	931.07	1211.73	262.95	24297.07	21460.93	22165.35	1.326329	0.00058	0.012688	0.035419	-4.81935
0.597	1621.12	30602.29	4341.07	79934.38	1410.65	73428.16	0.49081	0.253811	0.399553	0.236246	-2.08164
0.5969	115557.9	108023.8	99444.43	60900.81	64726.88	61029.72	1.352152	0.007036	0.039121	1.730583	0.791258
0.5968	16544.09	46170.86	7341.06	48551.61	13960.65	53342.63	0.567285	0.421673	0.551394	0.604688	-0.72574
0.5958	9169.42	302030.1	10535.36	671687.4	540522.6	497966.4	1.078743	0.023822	0.080475	0.18813	-2.4102

0.5957	76238.03	45118.29	73502.92	33264.41	47462.67	35351.25	1.071621	0.102287	0.214464	1.678687	0.747334
0.5954	1070.12	1062.45	1581.05	1714.64	572.67	852.25	0.475555	0.653267	0.746224	1.182847	0.242264
0.5952	1426.12	224.42	828.14	23211.04	17126.24	17843.89	1.30464	0.008818	0.044309	0.042603	-4.55291
0.5948	159.22	498.84	493.05	176.94	52.52	505.96	0.581519	0.47602	0.599183	1.565242	0.646385
0.5936	4073.21	3786.56	5252.4	2231.39	1989.07	1300.89	1.259408	0.013437	0.056593	2.374812	1.247814
0.5936	19661.24	11385.72	14678.26	41636.55	33836.77	36380.28	1.28795	0.002709	0.024343	0.408795	-1.29055
0.5933	43710.64	52630.23	33396.1	161684.3	138207.5	135875.3	1.337068	0.000972	0.014975	0.297721	-1.74797
0.593	280301.9	213153.4	204309.1	14242.14	1531.19	273.58	1.241079	0.009121	0.045025	43.48279	5.442373
0.5927	3531.89	6410.67	2629.24	144280.4	178362.8	168892.1	1.358978	0.003666	0.02833	0.025577	-5.28903
0.5919	2077.11	4650.78	4204.68	863.73	1785.95	1346.18	1.142715	0.088172	0.194627	2.735974	1.452055
0.5918	26571.94	30121.46	30386.55	17703.31	16375.08	177.68	0.768507	0.082712	0.185847	2.54203	1.345981
0.5916	58850.11	38709.06	123.06	18205.45	244.84	87.83	0.537136	0.261816	0.407485	5.269263	2.397601
0.5913	2249.33	4075.8	6700.49	1638.35	1421.87	113.5	0.963995	0.113025	0.229364	4.104212	2.037105
0.5912	15613.18	20312.18	60112.7	37501.54	58813.9	35203.64	0.640016	0.512375	0.629849	0.730222	-0.45359
0.5905	99349.23	68038.85	114668.3	31924.55	24898.64	21083.88	1.312476	0.032818	0.099869	3.620421	1.856158
0.5903	1398.44	1177.58	1485.92	1367.54	3376.32	4587.35	0.958049	0.201307	0.339098	0.435307	-1.1999
0.59	13546.91	10119.16	12243.6	21608.49	22526.18	38024.06	1.227338	0.096761	0.207163	0.437077	-1.19404
0.5898	535.64	7586.45	11085.96	743	14131.33	8473.76	0.117703	0.795586	0.857133	0.822682	-0.28159
0.5896	24028.53	53280.91	28287.85	41157.53	41208.63	24515.46	0.105105	0.970334	0.977962	0.987984	-0.01744
0.5885	22232.69	13207.3	22037.07	14063.78	8211.43	14347.75	0.921198	0.13467	0.256991	1.569427	0.650238
0.5882	191.45	525.87	370.37	44.94	221.38	240.08	0.81447	0.179111	0.313175	2.147887	1.102918
0.5881	22427.8	190994.1	98012.53	178327.2	74150.25	74673.96	0.273847	0.93483	0.952976	0.951958	-0.07103
0.5881	694068.7	576971.9	444593.9	4065.98	209122.2	2272.97	1.09886	0.007409	0.039933	7.962617	2.993243
0.5871	14228.21	10580.29	13709.05	95.95	3863.45	1757.12	1.050812	0.002288	0.022161	6.737937	2.752307
0.5858	48558.96	29906.33	57306.36	20344.66	8072.82	13642.7	1.203664	0.044205	0.12157	3.228033	1.690655
0.5854	438332	490721.1	540603.9	2459391	2042167	1892021	1.360417	0.008932	0.044648	0.229865	-2.12114

0.5851	243209.8	152406.2	230965.4	838.05	2463.2	1915.19	1.358164	0.018324	0.068093	120.1167	6.908292
0.585	161798.8	122138.9	158578.7	32808.47	22208.97	18148.05	1.342534	0.005792	0.035809	6.048158	2.596496
0.5839	636.03	1652.53	86167.98	3332.95	600.04	28637.26	0.057018	0.585331	0.692511	2.715869	1.441414
0.5832	24776.76	24822.8	23680.17	54331.52	57576.72	54877.44	1.370766	0.00028	0.009591	0.439365	-1.18651
0.583	34941.29	29544.59	30939.3	387045.7	322559.5	434328.5	1.368541	0.008356	0.042902	0.083418	-3.58349
0.583	33677.26	6446.65	216.6	59834.87	61843.52	4313.65	0.673639	0.273159	0.417956	0.320183	-1.64303
0.5826	731668.4	542428.9	597814.5	47975.43	39558.15	38997.7	1.368337	0.009052	0.044899	14.79406	3.886947
0.5825	99591.54	242390	68708.5	20952.08	14443.97	5390.39	1.238995	0.146704	0.272446	10.06928	3.331889
0.5821	6955.14	10029.37	14464.69	16278.1	12382.82	12528.8	0.759657	0.283049	0.426423	0.763521	-0.38926
0.5821	500136.5	127679.1	134788.4	15037.45	22596.03	188432.5	0.92047	0.28373	0.426798	3.373369	1.75419
0.5807	16329.82	8225.35	27245.5	27600.76	27373.68	41330.39	0.94983	0.110124	0.22611	0.537882	-0.89464
0.5805	82030.38	67302.12	59421.57	147004.2	136100.1	131852.6	1.320394	0.001748	0.019334	0.503074	-0.99116
0.5795	9561.08	10214.14	11433.27	68702.19	63860.95	61337.7	1.369664	0.000887	0.01464	0.160951	-2.63531
0.5785	2642.93	10682.52	9969.11	3504.83	5680.99	26485.05	0.18733	0.638843	0.734933	0.653042	-0.61475
0.5761	12196.26	12318.02	13106.64	868.71	2312.23	617.11	1.306589	0.000283	0.009591	9.905325	3.308204
0.5718	11924.12	2620.51	17646.73	12168.15	8159.95	10571.59	0.243632	0.931994	0.95142	1.041802	0.059081
0.5713	11542.15	13777.53	26654	19788.79	22547.7	20615.71	0.614021	0.519925	0.636373	0.825605	-0.27648
0.5708	2219812	1856401	2093187	642589.5	589481.6	559298.1	1.365153	0.003728	0.028674	3.443958	1.784067
0.5684	106589.9	91972.42	98099.05	46208.07	40187.45	36167.45	1.349778	0.000663	0.0135	2.420481	1.275294
0.5673	5724.58	4096.43	4700.2	1710.34	1335.93	1729.46	1.33741	0.015557	0.061649	3.040626	1.604368
0.5665	15290.56	25992.36	23555.76	978.95	503.37	2508.46	1.303164	0.021518	0.075726	16.24712	4.022112
0.5663	25341.92	21647.74	12876.5	9406.43	13810.04	11402.62	1.02246	0.139642	0.263735	1.729282	0.790173
0.5661	2654.56	550.91	115.43	2033.66	1183.85	548.78	0.453743	0.878362	0.914226	0.881743	-0.18157
0.5652	10645.11	11349.87	8358.85	59558.61	48339.82	40148.91	1.349707	0.017899	0.067027	0.205028	-2.28611
0.5651	473886.9	487184.5	448141.1	254649.7	271056.7	222505	1.346589	0.000352	0.010018	1.883442	0.913371
0.5645	4991.77	5812.52	840.97	7401.56	8632.41	5825.36	0.803101	0.144275	0.269449	0.532736	-0.90851

0.563	427849.4	294713.7	360307.2	606424.6	523546.1	415240.5	1.03644	0.092244	0.200995	0.700791	-0.51294
0.5606	85026.12	72031.53	81164.75	259361.7	221186.3	216940.5	1.359565	0.004776	0.032479	0.341543	-1.54986
0.5606	20788.47	17360.79	20447.12	43181.79	65.89	24988.3	0.479683	0.821458	0.875486	0.858731	-0.21972
0.5605	30547.13	5592.01	40039.37	154630.5	152350.2	110149.2	1.160036	0.004304	0.030911	0.182625	-2.45304
0.5604	395248.6	373339.2	397487.5	314046.8	299936.1	289227.9	1.33429	0.001166	0.015971	1.291033	0.368526
0.56	2815.72	5579.53	2389.73	5260.24	11913.91	6033.71	1.000275	0.177573	0.311484	0.464712	-1.10559
0.5585	2900940	3215820	2833263	15895146	14436071	14083254	1.36984	0.001498	0.017674	0.201511	-2.31107
0.558	523910.2	517206	434548.7	1212606	645140.4	963816.3	1.16307	0.107823	0.222749	0.522995	-0.93513
0.5577	4362.12	40856.12	18721.58	11554.11	2425.7	8970.57	0.642866	0.326011	0.468077	2.786003	1.478197
0.5575	56718.8	38833.56	43631.5	628.56	11147.78	8825.5	1.093074	0.006099	0.036575	6.755895	2.756147
0.5573	16523.87	10365.78	18341.87	11819.73	15112.86	14015.9	0.268302	0.626155	0.725714	1.104596	0.143518
0.5566	30666.45	26454.37	27040.34	81084.21	31462.89	40526.92	0.918413	0.270611	0.415793	0.549807	-0.863
0.5555	172010.9	141352.6	138796.2	107425.2	124808.1	123903.8	1.116598	0.075881	0.174916	1.269623	0.3444
0.5549	1410.77	4583.71	2969.53	6701.56	14458.28	9806.53	1.161734	0.066986	0.160434	0.289476	-1.78849
0.5536	22469.68	40495.31	28741.24	98.46	7707.99	1832.68	1.077144	0.021821	0.076035	9.513953	3.250045
0.5526	2602.2	2411.38	3100.04	374.64	563.05	980.87	1.282561	0.001747	0.019334	4.229016	2.080322
0.552	28954.24	24572	25599.91	2789.2	12691.87	8217.73	1.146609	0.011795	0.052516	3.338825	1.739341
0.5484	46103.2	68620.11	25403.03	425898.9	429421.4	377872.9	1.329346	0.000105	0.006088	0.113629	-3.1376
0.5461	27895.73	38784.82	34087.86	2656.19	10805.07	2617.25	1.244997	0.00267	0.024096	6.267273	2.647838
0.5456	629.31	1749.77	372.13	250.01	353.21	304.51	0.957964	0.283108	0.426423	3.030868	1.599731
0.5442	718288.4	288821	175272.4	150044.2	97506.74	126578.3	1.035178	0.244138	0.387827	3.160357	1.660087
0.544	79186.84	82004.4	73653.94	101603.2	95928.85	90016.86	1.244289	0.015977	0.062461	0.816714	-0.2921
0.5413	13601.82	16162.34	13877.93	5288.14	3525.67	6338.53	1.301648	0.00119	0.015971	2.880221	1.52618
0.5398	10248.33	1968.98	5710.28	40433.14	32595.61	6654.24	0.954243	0.175829	0.309797	0.224986	-2.15209
0.5394	33124.67	30620.47	31788.98	17530.39	11327.87	11060.27	1.300996	0.007371	0.039816	2.393227	1.258957
0.5389	16578.94	22379.75	15316.43	35001.58	34982.06	24245.87	1.171701	0.043993	0.121282	0.575989	-0.79589

0.5384	14551.69	9641.06	12811	11915.91	8888.14	7406.49	0.824119	0.208873	0.347909	1.311699	0.391437
0.5375	53233.95	3243.38	9510.9	3983.42	2402.67	42953.05	0.257022	0.801015	0.861279	1.337442	0.419476
0.5366	1361.2	2179.76	2911.48	1891.6	2871.49	1395.76	0.099091	0.882849	0.917582	1.04767	0.067184
0.5355	41527.68	48226.07	42312.61	54283.37	51355.37	44956.28	0.913488	0.154638	0.282628	0.876964	-0.18941
0.5338	6568.08	5654.7	3558.4	5592.92	2817.75	2729.21	0.730077	0.29846	0.441162	1.416638	0.502471
0.5336	2621.9	598.03	613.12	7262.73	8004.78	1789.46	1.03041	0.142738	0.267436	0.22472	-2.1538
0.5335	1602.44	1575.1	1318.12	648.21	1412.19	1548.81	0.614406	0.405462	0.539008	1.245608	0.31685
0.5333	44074.02	39045.6	36797.18	4787.84	2423.31	3360.36	1.354659	0.00165	0.018733	11.34339	3.50378
0.5326	1849139	1650092	1517074	3317011	3325738	2977049	1.344303	0.000596	0.012748	0.521456	-0.93938
0.5314	42062.38	41460.67	29440.99	220147.7	87539.67	42523.62	0.940943	0.275859	0.420225	0.32256	-1.63236
0.5306	7795.39	9229.66	12100.39	2659.43	4227.01	3309.38	1.294989	0.026984	0.087386	2.856606	1.514302
0.5305	260011.7	242912.1	250578.5	104351.6	96853.3	93618.86	1.368627	4.81E-05	0.004503	2.555772	1.353759
0.5303	583.71	47.25	1002.74	452.18	243.96	314.19	0.053124	0.5335	0.649035	1.616996	0.693316
0.528	41777.89	23581.47	27426.53	7053.16	9965.97	1716.31	1.163947	0.031541	0.096959	4.952427	2.308136
0.5268	1883.38	6599.92	2097.67	4389.44	4626.77	3472.28	0.513857	0.7226	0.803604	0.847258	-0.23913
0.5267	7254.53	8766.7	6793.7	34243.86	31615.9	30964.67	1.364186	0.000134	0.00671	0.235632	-2.08539
0.5266	7062.37	9358.16	5772.09	10633.92	8956.99	8340.06	0.841691	0.21264	0.351897	0.794552	-0.33179
0.5237	5861.95	10748.83	5862.45	1564.06	2690.06	1289.96	1.263599	0.065775	0.158932	4.053554	2.019188
0.5237	87348.98	90644.71	106793.9	18969.63	12175.98	15896.23	1.356434	0.002916	0.025269	6.053921	2.59787
0.5228	26219.88	19211.25	8939.77	43767.78	21148.32	22279.87	0.740456	0.294826	0.438019	0.623548	-0.68143
0.5202	26651.83	21188.83	21631.55	41873.6	36639.61	41427.09	1.312074	0.00228	0.022139	0.579223	-0.78781
0.5177	30071.45	29149.69	36205.24	542.31	373.51	3575.27	1.276999	0.001468	0.01744	21.24793	4.409251
0.5174	19889.37	19211.44	19604.7	912.88	3172.38	806.09	1.305559	0.00105	0.015525	12.0019	3.585191
0.5136	8070.08	7662.84	8262.17	279.83	260.75	282.2	1.372685	0.000514	0.012047	29.16343	4.866089
0.5118	9657.26	8218.21	12264.94	284.28	1065.18	1798.34	1.251793	0.009706	0.046882	9.575071	3.259283
0.504	11308.39	14137.12	13159.06	9959.25	3751.66	5843.25	1.089873	0.055659	0.141056	1.974238	0.981296
0.504	195.98	101.58	190.77	334.08	162.02	647.13	0.867989	0.261505	0.407399	0.427149	-1.22719

0.5023	56101.24	51603.85	48353.64	38433.84	31300.52	40479.45	1.223409	0.014172	0.058571	1.415963	0.501784
0.7643	882.87	3450.97	109.51	3721.73	3298.54	1441.78	0.73452	0.34388	0.484948	0.525091	-0.92936
0.7097	9151.47	8853.53	1288.57	6893.83	13934.73	21415.47	0.776029	0.209083	0.348054	0.456717	-1.13063
1	1878004	1662528	1781023	1620052	1444096	1330761	1.128512	0.046472	0.125587	1.210845	0.276014
0.9594	16287.85	16974.15	13504.32	4024.17	1002.16	2615.2	1.263266	0.000818	0.014373	6.12002	2.613536
0.9374	22738.06	22979.54	22547.19	7502.55	1476.5	2576.35	1.240562	0.009226	0.045392	5.907609	2.562574
0.9186	50205.92	45994.65	48554.41	72606.92	40183	71656.52	0.6406	0.339839	0.480649	0.784808	-0.34959
0.89	37173.95	37213.11	13417.49	16392.5	24231.35	14933.84	0.62509	0.307312	0.45066	1.580421	0.660309
0.8795	14335.59	16562.9	17866.41	5944.88	7216.82	6230.03	1.34919	0.005523	0.034788	2.514727	1.330402
0.8556	7540.15	925.37	7448.47	797.26	6786.5	931.32	0.506059	0.450425	0.577733	1.868918	0.902204
0.854	6389.57	189.39	977.38	1065.94	445.31	338.08	0.406405	0.432189	0.559357	4.085988	2.030685
0.82	24891.75	11855.92	25544.69	2434.29	5804.42	6051.01	1.217077	0.061378	0.151042	4.359243	2.124078
0.7824	396403.2	363425.4	379806.9	552848.7	498006.1	486475.3	1.310176	0.011623	0.052124	0.741308	-0.43185
0.7823	24581.84	4123.82	33955.81	20638.22	19566.63	17260.21	0.241027	0.862721	0.903122	1.090427	0.124894
0.7703	51222.07	50759.39	49430.82	47252.47	28421.42	40037.64	0.955279	0.161071	0.290609	1.308532	0.38795
0.75	28957.82	25243.25	28776.71	61835.92	51035.5	58652.66	1.346013	0.005895	0.036092	0.483768	-1.04761
0.7356	4920.94	32062.66	5226.67	3657.8	233.53	13891.01	0.636406	0.474552	0.597681	2.373719	1.247149
0.7289	1363.28	2312.24	3208.03	2361.19	889.36	821.06	0.788927	0.269891	0.415709	1.690621	0.757553
0.7218	124696.1	111776.7	120237.2	112827.4	91849.72	94986.61	1.07025	0.08088	0.182671	1.190367	0.251407
0.7152	35076.69	21224.96	32012.33	12751.48	15428.2	1984.75	0.980746	0.029988	0.093637	2.927752	1.549794
0.6802	381648.7	314510.7	318322.6	27894.06	33260.11	23003.7	1.366241	0.0043	0.030911	12.05451	3.591501
0.6593	7731.62	3651.03	1939.29	58430.94	49083.26	39468.95	1.305487	0.009492	0.046283	0.090636	-3.46377
0.6543	16320.77	14600.64	8249.28	22107.49	18229.16	19273.34	0.993185	0.091387	0.199677	0.657116	-0.60578
0.6523	426168.1	404990.1	389739.2	520856.7	461590.2	469131.2	1.211848	0.033885	0.102007	0.841083	-0.24968
0.643	3032.05	7299.22	4949.48	2611.76	8953.24	2072.81	0.347025	0.841969	0.888655	1.120469	0.164103
0.6417	153923.5	141584.3	170050.6	108695.6	103685.3	94813.72	1.307984	0.01131	0.051196	1.515516	0.599809
0.6204	507.97	488.14	766.68	1132.4	579.9	3092.59	0.843092	0.31437	0.456932	0.366874	-1.44664

0.5772	554.42	5724.61	2272.71	10865.11	10497.23	9385.47	1.063044	0.031458	0.096773	0.278125	-1.84619
0.5021	164.38	275.04	192.52	1564.83	1878.82	973.09	1.329567	0.039715	0.113237	0.143078	-2.80512
0.8044	366804.2	378506.8	343449.3	1124.48	25251.26	8333.29	1.252688	2.62E-05	0.003892	31.36821	4.971231
0.7316	13063.59	11264.59	11129	9739.14	10528.78	12200.97	0.646614	0.357858	0.498531	1.092035	0.12702
0.6662	2183.79	480.57	1013.28	52.3	574.33	198.68	0.995191	0.192184	0.328007	4.456071	2.155772
0.9349	961305.7	779620.3	932363.8	751318.2	670769.4	637842.1	1.162947	0.047005	0.126792	1.297758	0.376021
0.8958	134826.4	108429.2	126266.2	85659.43	81356.47	74274.33	1.290893	0.019073	0.070042	1.531441	0.61489
0.8949	67664.6	130525.3	122708.6	46633.52	9417.78	46274.75	1.040115	0.044975	0.122759	3.136039	1.648943
0.8934	5008.05	6752.4	6448.95	5275.35	1536.87	487.5	0.953575	0.116364	0.233919	2.494534	1.31877
0.8619	15590.48	16356.7	20496.77	1553.27	5601.07	8983.27	1.093397	0.012813	0.055229	3.249797	1.700349
0.8574	123316.3	68769.05	92604.48	50778.76	30482.72	125917.4	0.631561	0.489798	0.60966	1.374126	0.458514
0.8327	2741.18	3713.04	3459.44	31168.74	27262.68	30556.29	1.367122	0.001297	0.016383	0.111405	-3.16612
0.8043	16954.09	15462.84	18404.93	390.95	588.34	933.08	1.356894	0.002041	0.020785	26.57533	4.732016
0.7754	390477.3	622977.8	498599	915325.5	899907.3	781877.6	1.219186	0.015364	0.061104	0.582206	-0.7804
0.7742	31388.11	31959.05	29621.19	46842.15	42802.69	41702.55	1.333821	0.006439	0.037623	0.707805	-0.49858
0.7689	69587.25	56974.26	67540.08	40959.01	35263.31	25362.73	1.244691	0.007237	0.039577	1.91073	0.934124
0.7675	12274.39	8018.66	12428.14	3838.83	860.52	1264.95	1.229826	0.009983	0.04767	5.486174	2.4558
0.765	109770.8	88727.04	145964.3	74079.13	86485.42	74131.64	1.052931	0.153528	0.281307	1.467694	0.553551
0.7602	9481.23	3872.94	4889.34	120693.5	53028.69	48295.13	1.308029	0.100105	0.211515	0.082172	-3.60522
0.7573	89238.27	65220.8	45930.92	2364.57	4104.39	240.37	1.248312	0.03475	0.103608	29.86736	4.900498
0.7289	62891.42	64477.71	49173.65	155890.2	154161.2	130036.3	1.338789	0.002114	0.021043	0.401154	-1.31777
0.7277	29296.3	16527.5	15403.47	10073.96	122.36	10421.34	0.796414	0.077324	0.17749	2.969652	1.570294
0.7159	149831.6	107979.9	149135.8	610779.4	641555	551960.8	1.356402	0.000534	0.012243	0.225544	-2.14852
0.7075	9582.23	2587.13	11271.62	1846.91	2740.63	3940.25	0.891959	0.197826	0.335143	2.748775	1.458789
0.9593	1436.33	2257.07	716.51	4056.58	3253.02	3199.37	1.120862	0.024884	0.082674	0.419633	-1.2528
0.9524	340864.5	280373	297644.2	971431	897808.4	838238.1	1.361002	0.001054	0.015525	0.339387	-1.559

0.9184	286711.5	1150230	571651.4	560578.4	600417.9	493551.4	0.071995	0.688841	0.777126	1.213983	0.279748
0.9152	3571241	3564749	3283113	5565306	4408814	4325287	1.201878	0.076688	0.176216	0.728639	-0.45672
0.9067	69477.48	39386.95	51931.8	40812.98	41618.61	43101.04	0.764748	0.310055	0.453533	1.280912	0.357171
0.9025	22543.34	10781	14883.29	15383.77	16360.78	7968.97	0.410318	0.552603	0.667208	1.213885	0.279631
0.9018	548.47	2437.87	11340.74	645.9	862.74	364.09	0.852269	0.338394	0.479071	7.650371	2.93553
0.8926	147084.6	237072.3	221250.8	129772.3	142600.7	144895.6	1.037408	0.148669	0.274619	1.450882	0.536931
0.8926	327785.2	264536.6	287366.4	251690.4	151507.9	132005.9	1.087194	0.071158	0.167122	1.64365	0.716903
0.8913	18407.95	25556.48	25808.63	59375.69	50457.73	48182.38	1.310891	0.003157	0.026416	0.441557	-1.17933
0.891	1854421	2336627	1842939	2534029	2275773	2079866	0.787505	0.247345	0.391353	0.875802	-0.19132
0.8901	13917.69	14754.17	9695.86	2644.88	2379.42	6047.32	1.232576	0.011563	0.051928	3.465412	1.793027
0.8847	17958.63	17796.03	15838.33	42589	41910.17	35294.74	1.352982	0.006589	0.038142	0.430681	-1.21531
0.8778	125317.8	128927.9	115667.6	142406.9	154118.6	128391.2	1.017271	0.116313	0.233919	0.870555	-0.19999
0.8739	91033.45	97950.5	68670.49	225468.8	356909.2	188846.1	1.271479	0.074467	0.172568	0.334085	-1.58171
0.873	17664.73	15538.81	20579.44	25849.15	24961.68	26233.78	1.250987	0.027466	0.088333	0.698076	-0.51854
0.8652	215954	240288.2	256753.1	88357.76	64892.5	67288.24	1.350188	0.000757	0.014145	3.232974	1.692862
0.8646	660845	751328.5	803404.5	187333.4	180854.3	179604.5	1.368243	0.005442	0.034679	4.044559	2.015983
0.8569	55091.56	43179.3	58744.15	40151.33	39302.12	40609.3	1.111707	0.118985	0.236614	1.307775	0.387114
0.8394	159955.1	215406.4	172621	71194.68	76010.3	91646.03	1.321016	0.016269	0.063099	2.294244	1.198019
0.8387	791153.7	1147848	645823.9	398659.3	462009.1	304008.5	1.211911	0.075642	0.174549	2.21935	1.150137
0.837	494.2	174.31	508.37	12260.37	9463.94	4161.79	1.311369	0.073722	0.171388	0.045464	-4.45914
0.8355	75464.11	222774.9	162189.7	191297.3	103720.5	111682.7	0.127568	0.746233	0.821007	1.132107	0.179011
0.8297	274453.1	257002.1	230772.8	137446.6	87556.13	44504.72	1.186765	0.013312	0.056282	2.828226	1.499897
0.8259	3641.36	3224.64	3847.66	7968.12	6034.35	8153.76	1.312964	0.023747	0.08043	0.483551	-1.04826
0.8194	87690.86	42783.61	112102.1	361383.8	349440.8	289498	1.274738	0.001146	0.015971	0.242498	-2.04395
0.8168	137992.3	106912.7	89018.57	494.72	510.58	648.07	1.370547	0.01628	0.063099	201.9654	7.657964
0.8097	510.56	3277.82	976.67	1268.14	1443.36	4529.68	0.508935	0.578437	0.687032	0.658049	-0.60373

0.8095	885.18	671.97	917.69	2734.8	1169.31	2882.99	1.14831	0.117148	0.234672	0.364639	-1.45546
0.8046	6678.2	9198.57	12202.22	7630.08	6356.36	10955.5	0.324446	0.645909	0.740536	1.125774	0.170917
0.8	4733.18	3541.41	1505.06	27716.16	31045.85	29155.97	1.315421	4.25E-05	0.004423	0.111236	-3.1683
0.7964	555788.9	684518.1	532978.7	1248992	1034632	1026205	1.307035	0.006637	0.038268	0.535764	-0.90033
0.7958	3529.98	3528.65	4661.22	67117.67	2923.72	68559.32	0.896344	0.189844	0.325095	0.084558	-3.56391
0.7928	71250.93	64260.47	59587.75	155072.9	137204.7	122734	1.339118	0.009155	0.045143	0.470105	-1.08894
0.7903	21699.61	18020.02	22048.24	5236.63	10940.14	7159.61	1.256577	0.004586	0.032243	2.646849	1.404276
0.7887	1759.82	2655.76	374.84	1872.63	1290.92	1155.44	0.173603	0.839625	0.887873	1.109153	0.149458
0.7882	167968.1	128620.6	140833.6	304403.6	292470.4	272293.1	1.333466	0.000802	0.014349	0.503266	-0.99061
0.785	24758.49	17246.8	24606.41	15897.16	8400.32	19415.54	0.879823	0.1401	0.264256	1.523841	0.607713
0.7806	53918.99	51035.34	51128.21	11821.37	779.28	580.41	1.195688	0.003988	0.029413	11.84143	3.565771
0.7785	22139.99	23882.5	15065.36	23960.06	13584.36	17469.36	0.343344	0.64396	0.739079	1.11041	0.151092
0.7731	22949.63	7015.9	22613.74	43838.17	36390.58	34616.84	1.029984	0.038756	0.112103	0.457826	-1.12713
0.7729	324942.2	288584.3	312412.1	156379.9	150272	135103.1	1.357686	0.000646	0.013279	2.096046	1.06767
0.7719	158290.4	121226.2	100825.8	183675.2	145025.3	165801.5	0.936385	0.143217	0.267818	0.769142	-0.37868
0.7674	9192.4	6268.7	6842.62	3495.96	7123.46	3376.09	0.94816	0.14941	0.275616	1.593634	0.67232
0.766	7139.13	5047.6	5748.1	3674.17	4659.81	4695.36	1.061047	0.099002	0.210095	1.376496	0.461
0.7629	7509.69	4932.34	6916.43	1592.32	116.56	1217.96	1.11611	0.007493	0.040174	6.614116	2.725548
0.7615	2999.57	3182.63	2665.52	5036.78	6225.83	33653.48	0.98553	0.327141	0.469235	0.196983	-2.34385
0.7592	333472.4	276346.5	317933.7	131631.8	137833.9	134203.4	1.359678	0.008815	0.044309	2.2983	1.200567
0.7563	13694.01	23326.42	18183.37	5581.08	8002.02	3995.18	1.259011	0.031415	0.096707	3.140455	1.650974
0.7536	1210.15	3771.21	4806.3	1485.5	766.27	820.48	1.021008	0.166256	0.296891	3.185828	1.671668
0.7501	47451.88	74528.5	44642.68	17793.97	19159.19	19226.79	1.31174	0.060598	0.149829	2.965881	1.568461
0.7489	682520.4	425774.7	587411.1	281942.5	286785.3	287580.5	1.265996	0.06476	0.157511	1.980252	0.985684
0.7445	40560.29	36537.55	29970.61	67530.67	59789.8	44501.16	1.149037	0.06787	0.162094	0.623137	-0.68238
0.7433	1954.32	2161.78	5069.54	1343.7	2130.28	2062.4	0.738877	0.34985	0.490827	1.659142	0.730437
0.7409	49620.73	42749.17	62196.87	50225.04	109311.2	36091.62	0.263532	0.608025	0.711321	0.790106	-0.33988

0.7396	26007.48	22312.69	24646.93	683.13	1727.33	4847.8	1.25828	0.000213	0.008835	10.05297	3.32955
0.735	77.04	581.39	828.25	431.87	727.2	5241.98	0.702652	0.403182	0.537125	0.232256	-2.10621
0.7342	600025.1	436452.8	547947.3	88151.9	83709.83	71248.66	1.362849	0.010792	0.049697	6.517307	2.704276
0.7337	9729.49	15012.46	13490.85	482.45	980.9	1669.53	1.317132	0.014215	0.058571	12.20372	3.609249
0.7326	57352.92	73013.27	57405	130094.3	112063.7	89329.97	1.236583	0.039208	0.112605	0.566449	-0.81998
0.7274	99613.24	67515.46	81567.73	58903.54	79904.25	71555.09	0.676296	0.323636	0.46585	1.182226	0.241506
0.7263	27945.16	28716.58	10877.46	34519.91	29750.51	29443.34	0.741663	0.269676	0.415657	0.720697	-0.47254
0.726	136123.7	86805.76	46740.23	3920.98	2248.48	2572.83	1.343661	0.078059	0.178825	30.84657	4.947038
0.7258	1003.45	838.87	1077.31	1006.68	972.02	1216.04	0.557617	0.427211	0.554726	0.913887	-0.12991
0.7201	89714.36	76730.15	88703.88	308104.2	253884	267146	1.361293	0.00486	0.032817	0.307729	-1.70027
0.72	5328.61	3898.62	4624.29	839.09	828.59	1030.83	1.359442	0.010619	0.049505	5.133025	2.359809
0.7187	20301.19	58127.65	28695.33	18416.46	12809.19	9439.16	1.070402	0.188278	0.323507	2.634321	1.397431
0.7158	950502.5	229427	277592	252977.6	41534.01	71218.81	0.994854	0.254635	0.400465	3.985234	1.994664
0.7154	12272.97	5497.74	5144.76	73149.31	53248.14	64935.6	1.32642	0.00476	0.03246	0.119767	-3.06169
0.7149	7198.07	7059.04	7046.48	4840.45	9085.33	4382.61	0.565261	0.573543	0.683218	1.163597	0.218592
0.7137	17857.1	1321.53	10198.47	137730.5	120440.5	107214.5	1.210474	0.001374	0.016837	0.0804	-3.63666
0.7125	5292.32	13962.67	3621.22	9630.35	6997.14	4191.32	0.02628	0.86026	0.901416	1.098824	0.13596
0.7111	8321.42	5766.25	5209.43	8845.65	9878.57	5648.64	0.62406	0.352419	0.493266	0.791745	-0.33689
0.7108	64650.38	146680.5	185276.7	3940.72	8600.75	41617.03	1.154804	0.072618	0.169637	7.323089	2.872452
0.7103	207.87	75.23	320.41	1405.89	1123.13	1549.4	1.262663	0.003297	0.027017	0.147976	-2.75656
0.7081	240.46	1470.14	2239.65	1681.87	890.52	1361.12	0.296144	0.993524	0.994893	1.004256	0.006127
0.7066	18056.1	15900.62	15423.28	17572.34	16668.18	17806.11	0.635891	0.394453	0.530521	0.948765	-0.07588
0.7057	3293.15	8380.74	5136.55	29043.87	25744.09	22900.95	1.296246	0.00107	0.015658	0.216381	-2.20835
0.7023	5580.49	5750.11	4507.61	13479.81	12221.74	10929.36	1.337358	0.003477	0.027553	0.432373	-1.20965
0.7011	37193.61	84363.94	30009.14	235.1	20278.11	22683.38	0.811109	0.156499	0.284953	3.508765	1.810963
0.6971	14889.13	15920.52	19811.95	8082.93	2921.76	2839.47	1.228342	0.006248	0.037156	3.656531	1.870476

0.6937	209669.2	177285.6	193578.1	348929.7	302751.5	283491	1.304047	0.013249	0.056224	0.620777	-0.68785
0.6892	9860.86	5313.09	9742.58	564.45	8321.26	922.25	0.936105	0.177957	0.311532	2.54044	1.345078
0.6875	3861	4615	3907.73	4964.05	4563.9	3492.57	0.237908	0.700068	0.785087	0.951093	-0.07234
0.6868	1277.95	415.42	1532.39	61.13	653.37	641.45	0.747653	0.202952	0.34152	2.378967	1.250335
0.6864	231240	257106.9	213767.7	282788	205465.2	125179	0.49172	0.587624	0.694725	1.144567	0.194802
0.6861	43116.14	26333.05	15827.59	95519.82	130495.1	87126.36	1.248217	0.013203	0.056205	0.272327	-1.87659
0.6846	4192.59	6330.36	6772.89	5208.12	2711.22	5312.39	0.658439	0.309839	0.45337	1.307149	0.386423
0.6799	186610.7	160157.1	151886.9	30523.08	44150.39	9361.94	1.237474	0.000692	0.013647	5.933863	2.568972
0.677	1437.33	717.77	1703.1	1543.49	1197.91	498.42	0.332486	0.653514	0.746311	1.190869	0.252014
0.6764	4308.29	4221.97	5364.52	8564.58	5542.22	5561.1	0.976117	0.18675	0.322011	0.70647	-0.5013
0.675	1202.46	1411.19	4012.77	1408.54	5797.24	1308.57	0.165882	0.738632	0.816146	0.778265	-0.36167
0.6737	73010.06	46417.58	42936.69	86043.69	79332.48	82641.56	1.104294	0.089618	0.196799	0.654648	-0.61121
0.6679	107122.9	34084.7	84285.44	18291.03	17295.64	4036.34	1.145233	0.096935	0.207365	5.690962	2.508673
0.6665	658532.7	636926.2	554267.6	817914.3	771986.4	741540.9	1.221525	0.018026	0.067314	0.793383	-0.33391
0.6663	7428.25	1025.42	7423.82	1629.21	316.37	376.63	1.027172	0.163927	0.29442	6.837233	2.773413
0.6649	9753.94	2713.3	6859.73	121429.8	102036.4	88144.99	1.325187	0.007551	0.040336	0.062023	-4.01106
0.6648	2894.78	3425.15	5481.68	1971.05	5701.65	64156.92	0.522826	0.425094	0.553083	0.1643	-2.6056
0.6639	514.11	234.27	125.86	369.42	914.68	183.87	0.489273	0.482532	0.60476	0.595544	-0.74772
0.6637	28869.63	29828.92	31949.89	72636.44	64189.75	65847.79	1.362984	0.002114	0.021043	0.447262	-1.16081
0.6628	3284.57	10777.4	6190.33	317.43	4767.5	170.04	0.990607	0.141336	0.265668	3.853933	1.946331
0.6565	321702.8	225176.5	263017	300046.2	221448.3	218448.1	0.406961	0.579925	0.688425	1.094539	0.130324
0.6563	2448.97	1121.33	2658.73	7541.88	3988.09	1334.3	0.606222	0.343557	0.484649	0.484212	-1.04629
0.6552	15265.17	17621.94	8759.07	6104.64	5128.26	1214.83	1.06929	0.046025	0.124688	3.345685	1.742301
0.6546	3360.23	4308.16	1789.92	2355.48	9801.19	731.88	0.122441	0.726173	0.805852	0.733854	-0.44644
0.6531	78652.53	111407.2	61850.43	316992.4	336306	205362	1.285685	0.027479	0.088333	0.293376	-1.76918
0.6504	64513.28	35601.72	62201.11	84617.23	38590.89	63957.02	0.282971	0.639569	0.735201	0.867235	-0.20551

0.6486	21667.59	34043.69	35596.44	127034.8	129518.8	170770.2	1.335572	0.010258	0.048573	0.213673	-2.22652
0.6476	8129.74	6698.69	6060.71	2536.54	26051.95	31456.04	0.485385	0.279006	0.422874	0.347894	-1.52328
0.646	38857.57	32851.07	31598.06	10182.92	140.6	15357.98	0.879415	0.014621	0.059621	4.022612	2.008132
0.6458	4140.99	1857.77	3283.12	5323.41	202.63	154.36	0.803389	0.567287	0.678223	1.634019	0.708425
0.6432	590508	153724.3	126811.8	429971.1	318085.6	903572.4	0.779621	0.330374	0.4717	0.527385	-0.92307
0.6367	115146.3	132379.8	97353.98	143959	149236.1	124848.3	0.944273	0.130007	0.252092	0.824987	-0.27756
0.6344	12118.28	8736.42	11400.55	5308.01	3204.72	5729.06	1.22996	0.011412	0.051441	2.264831	1.179404
0.6327	15101.72	10165.86	15513.49	38271.48	36601.63	49437.89	1.316904	0.010686	0.049629	0.328057	-1.60798
0.6323	21980.02	20520.34	16889.79	2424.8	2942.77	4246.97	1.346464	0.004078	0.029875	6.177118	2.626934
0.6237	8949.5	5686.01	8842.9	4374.85	13726.44	5037.25	0.229515	0.974371	0.981094	1.014688	0.021037
0.6235	274253.9	243327.2	308130.6	536908.4	466815.8	349546.1	1.179394	0.07178	0.168221	0.61016	-0.71274
0.6222	5737	4900.53	4406.91	62970.31	100140.8	52647.38	1.356457	0.043369	0.120257	0.069728	-3.84212
0.6205	70827.88	62355.01	62934.79	206181.6	190937.8	181430.1	1.365812	0.00113	0.015971	0.338982	-1.56072
0.6203	3046.85	2854.86	485.17	24092.8	26086.6	28832.16	1.26042	0.000385	0.010245	0.080835	-3.62888
0.6199	4681.2	357.21	3574.38	3317.25	3210.77	2122.94	0.354513	0.993232	0.994829	0.995588	-0.00638
0.6189	31868.81	34061.1	61137.44	111285.2	133755.8	120827.5	1.27924	0.003428	0.027425	0.347303	-1.52573
0.6177	935559.9	437003.1	794701.4	260032.6	284145.1	262580.7	1.229788	0.092066	0.200825	2.686386	1.425667
0.617	2794.98	4661.41	676.67	19973.83	14578.83	9525.16	1.155545	0.044037	0.121282	0.184516	-2.43818
0.6134	290.18	1145.59	1495.73	660.91	285.58	205.79	0.781649	0.234172	0.375692	2.544087	1.347148
0.6098	13073.2	12563.58	10482.36	17601.31	20030.66	16515.2	1.258965	0.011772	0.052468	0.667055	-0.58412
0.6095	2476.83	3109.96	2587.3	5213.27	4844.82	6047.65	1.321729	0.006611	0.038167	0.507527	-0.97844
0.6085	2544.2	2808.01	43.72	1475.41	1707.06	1145.78	0.358416	0.727325	0.80672	1.246677	0.318088
0.6037	36855.17	40960.79	30726.49	313517.3	272976.3	281167.4	1.367446	0.001483	0.017568	0.125098	-2.99887
0.5986	51544.35	40193.77	44858.78	17582.42	438.99	18153.41	0.904377	0.01345	0.056593	3.776022	1.916867
0.598	172990.1	25638.71	3959.2	43757.3	35111.73	33358.11	0.222706	0.627956	0.727221	1.80516	0.852127
0.5977	177403	177363.6	171332	11741.15	6772.93	9660.88	1.36488	9.9E-07	0.001767	18.67256	4.222848
0.5936	11057.96	8392.95	12268.72	6210.08	2744.96	3475.17	1.207002	0.014684	0.059716	2.551818	1.351525

0.5932	160919.1	386668.4	436686.4	77422.22	208905.2	265006.3	0.729292	0.238521	0.380979	1.78526	0.836134
0.5923	404089.6	304673.1	355158.5	363318.7	439636.9	368966	0.60256	0.395689	0.531326	0.907843	-0.13948
0.5852	6263.17	9461.74	5600.21	14305.64	9079.97	16126.92	1.08753	0.083529	0.187137	0.539705	-0.88976
0.5806	8051.59	5642.54	5939.77	15528.88	14139.43	15965.38	1.324585	0.00116	0.015971	0.43025	-1.21675
0.5794	19038.13	21743.65	21196.63	37196.92	26026	34947.95	1.216652	0.064218	0.156384	0.631332	-0.66353
0.571	5574.49	8251.82	3054.88	7597.07	5035.91	399.32	0.4918	0.648471	0.74246	1.295335	0.373325
0.5671	1338.77	2718.43	1110.61	1017.52	1372.19	1420.76	0.515009	0.465458	0.590664	1.356213	0.439584
0.5611	5252.97	61287.74	77474.62	67237.42	68707.58	67998.87	0.603451	0.457649	0.584327	0.706152	-0.50195
0.5609	1506.01	850.15	1659.4	683.29	151.84	147.36	1.143492	0.034293	0.10288	4.087126	2.031087
0.5598	30945.41	22147.73	23775.41	2057.35	4610.04	1120.63	1.297751	0.006984	0.039121	9.870102	3.303065
0.5538	16910.13	13563.23	20607.03	15200.94	13254.13	6650.88	0.819746	0.184937	0.320151	1.455035	0.541054
0.5489	489.22	639.86	483.59	22.24	71.5	163.43	1.201322	0.002754	0.02464	6.270833	2.648657
0.5427	5494.62	4299.64	5791.27	767.35	4566.39	4973.29	0.667373	0.319101	0.4615	1.512126	0.596579
0.5385	2467.61	1066.24	2501.58	140.8	395.26	1189.23	1.028022	0.073996	0.171934	3.498212	1.806618
0.5318	273.02	1534.41	1852.47	649.32	726.17	1098.1	0.147208	0.502964	0.621304	1.47959	0.565198
0.5253	475.91	977.15	2953.17	142.38	211.95	894.31	0.88957	0.296771	0.439503	3.528823	1.819187
0.5161	1657.57	412.68	749.19	2492.15	2269.51	1321.99	0.968894	0.103043	0.21553	0.463445	-1.10953
0.5001	327.24	3570.57	3656.66	2728.2	2520.88	1470.36	0.234265	0.829086	0.880434	1.124271	0.16899
0.8115	79625.31	123407.4	110988.9	84736.6	103292.7	78939.87	0.607016	0.3679	0.506211	1.176246	0.23419
0.7917	281.54	405.07	1299.14	2510.16	2002.88	1473.5	1.09931	0.038612	0.112058	0.331702	-1.59204
0.7742	11269.26	12333.85	13702.57	21160.42	12259.38	9694.6	0.26061	0.636031	0.732679	0.865272	-0.20877
0.7147	231.84	305.44	178.45	860.99	3442.6	4545.51	1.246784	0.130884	0.252892	0.080882	-3.62804
0.9587	3290060	3756051	3234051	6805964	2078873	5652457	0.407655	0.423934	0.552941	0.707158	-0.4999
0.9563	10797.7	4345.98	8050.98	1183.9	1520.45	737.7	1.290587	0.069964	0.165097	6.738618	2.752453
0.9322	14672.73	12195.85	12429.73	1269.07	1189.96	1908.04	1.358843	0.002595	0.023737	8.998782	3.16973
0.9267	2105788	3304367	1916940	2194087	2256800	1834442	0.459657	0.513296	0.630521	1.165746	0.221253
0.9229	4850.59	441.14	621	3049.7	3407.84	2031.96	0.709408	0.617153	0.719489	0.696476	-0.52186

0.8883	93946.83	83585.1	68869.08	202211.3	205527.7	182403.4	1.340406	0.000365	0.010037	0.417528	-1.26005
0.8692	10183.95	9369.24	6936.81	11885.06	16733.25	12734.4	1.125731	0.059263	0.147701	0.640587	-0.64253
0.8451	137192.9	318817.5	565710.1	2068575	1714317	1627653	1.246637	0.001375	0.016837	0.188839	-2.40477
0.8344	29068.42	25727.2	28301.87	30706.59	28323.62	28810.86	0.733452	0.27925	0.423097	0.945998	-0.08009
0.8241	19572.85	11940.96	15572.66	22743.92	25020.13	22444.05	1.124141	0.058993	0.147387	0.67067	-0.57632
0.8228	9803.35	3904.33	6490.2	51879.85	42098.11	46662.3	1.324796	0.000759	0.014145	0.143614	-2.79973
0.8208	71015.48	121513.1	92502.8	301807.3	193268.6	256877	1.267962	0.023489	0.079805	0.379055	-1.39952
0.8201	3181.34	4080.43	345.98	2233.3	3306.79	1077.15	0.154031	0.814554	0.870042	1.149686	0.20124
0.8096	22628.86	16734.42	18449.92	9224.24	8478.26	8587.78	1.335687	0.02496	0.082765	2.199033	1.136869
0.8079	204532.6	156327.5	141632.6	102117.6	89766.79	92535.69	1.266656	0.057108	0.144226	1.766727	0.821079
0.8013	64894.98	47234.44	96211.36	19132.27	53365.11	14947.66	1.024443	0.100385	0.211903	2.382534	1.252497
0.7997	48112.53	18557.37	13599.45	43532.87	47767.97	37465.5	0.859586	0.269256	0.415317	0.623372	-0.68183
0.783	14362.33	19559.52	14388.28	4383.39	5533.73	4648.06	1.343975	0.019462	0.070696	3.316823	1.729802
0.7815	178067	169501.9	183608.3	85921.15	71362.91	78505.6	1.359201	7.45E-05	0.00568	2.252758	1.171692
0.7793	673910.5	301065.3	330805.8	1666.03	158756.7	159314.3	0.854908	0.094389	0.20419	4.083924	2.029956
0.7772	235068.7	188646.7	190918	32771.85	31990.51	424.64	1.030862	0.000977	0.014975	9.428773	3.23707
0.7763	570.95	613.15	301.32	364.87	228.31	192.07	0.998961	0.124034	0.243653	1.891652	0.919647
0.7756	18164.83	18976.27	23501.45	17741.82	15903.73	17709.93	0.931435	0.195148	0.331638	1.180839	0.239812
0.7699	1387204	831737.1	1175011	700407.6	673373.4	737338	1.138074	0.1165	0.23402	1.607656	0.684958
0.7656	31354.93	48019.23	33828.67	50870.4	46931.94	38407.89	0.731116	0.301206	0.44398	0.831089	-0.26693
0.7626	25760.42	21742.68	24403.67	11629.85	10830.41	5964.89	1.252466	0.003946	0.0292	2.529688	1.33896
0.7473	321743	393903.5	550097.6	35047.73	59173.79	47872.43	1.346437	0.029762	0.093255	8.907797	3.155069
0.7437	2562.47	3747.28	1774.98	10155.56	4334.97	2074.46	0.662573	0.361225	0.500654	0.488061	-1.03487
0.7424	77095.54	28916.84	65904.57	14842.82	12162.33	15709.66	1.239184	0.096999	0.207365	4.024762	2.008904
0.7396	65152529	53351604	57776571	54088015	49314328	44781206	1.01074	0.102589	0.214993	1.18961	0.250489
0.7395	152574.3	134770.9	112925.4	252404.4	257569.2	270200.1	1.327378	0.00276	0.02464	0.513053	-0.96282
0.7329	310909	262005.7	482841	280150	244527.5	241475.1	0.836927	0.284589	0.427471	1.377996	0.462572

0.7235	23429.52	13410.11	14992.32	4500.85	7689.14	7098.95	1.234825	0.062125	0.152646	2.687133	1.426068
0.7199	453847.1	444361.3	393402.5	260416.8	275353.4	261903.7	1.346048	0.009305	0.045573	1.619222	0.695301
0.7197	574390	850232.9	356272.4	698994.3	725565.1	650255	0.53298	0.565169	0.676248	0.85834	-0.22038
0.7151	522972.8	439463.9	459121.1	30903.88	35804.21	12974.18	1.342035	0.00178	0.019432	17.84033	4.15707
0.7135	5159.44	6229.87	5593.05	299.3	133.37	131.24	1.356032	0.002504	0.023375	30.11537	4.912428
0.7127	4739.78	5608.93	4892.14	9491.45	9309.49	7129.57	1.273382	0.031232	0.096282	0.587757	-0.76671
0.7122	117518.8	108182.2	111786.3	131062.2	114289.7	115375.2	0.746136	0.292701	0.4353	0.935575	-0.09607
0.7119	9110.49	6142.71	4352.07	7187.92	5371.73	6769.58	0.071093	0.955242	0.967853	1.014281	0.020457
0.7058	792.16	494.85	353.7	286.25	1172.94	314.31	0.120375	0.898953	0.928563	0.925125	-0.11228
0.7013	3572.99	3643.35	376.05	1647.28	661.01	1661.11	0.273168	0.380718	0.517754	1.91273	0.935633
0.6994	59166	58762.94	56530.24	26005.32	16926.29	27763.75	1.30122	0.006681	0.038427	2.46776	1.303202
0.6964	8925.64	8068.37	348.07	3425.35	353.1	156.7	0.686497	0.237943	0.380621	4.406968	2.139786
0.6902	515980.1	437425.6	551993.1	25725.96	50863.43	53935.07	1.346429	0.00342	0.027425	11.53346	3.527754
0.6881	45386.24	48967.86	41317.65	8706.48	4424.14	3793.74	1.334698	0.000252	0.009348	8.016359	3.002947
0.68	207991	305777.4	465078.2	83890.67	81747.02	68600.69	1.29692	0.079702	0.181044	4.178848	2.063105
0.6758	16901.69	16820.38	15634.37	52046.43	59364.03	49496.7	1.366013	0.005533	0.034797	0.306739	-1.70492
0.6713	11256.46	13824.34	13371.62	7787.64	8715.91	7859.93	1.306824	0.017363	0.065872	1.578281	0.658354
0.6685	2202.87	2913.93	4408.13	147.29	1105.36	329.3	1.180813	0.038523	0.112011	6.021006	2.590004
0.6654	510.02	348.27	145.85	26.94	477.28	239.92	0.481875	0.633032	0.730623	1.349397	0.432314
0.6623	20384.87	4411.53	28009.39	80940.72	68351.18	66543.22	1.129259	0.004692	0.032349	0.244658	-2.03116
0.6578	14538.7	10765.2	4890.27	920.03	1703.62	1324.89	1.28417	0.088431	0.194977	7.64692	2.934879
0.6534	30808.76	25147.34	24519.54	24789.41	18068.59	18791.13	1.017354	0.098498	0.209543	1.305382	0.384472
0.6502	2335.59	3496.91	1962.89	16324.67	13956.57	13497.92	1.344659	0.001149	0.015971	0.178062	-2.48955
0.6491	68067.7	14218.66	50833.7	54724.61	67997.22	70354.16	0.681812	0.335062	0.476586	0.68947	-0.53644
0.6488	5008.06	1647.1	3618.13	2682.28	1659.3	74.05	0.741313	0.193411	0.329585	2.326574	1.218207
0.6434	58.1	232.01	426.47	1627.66	996.88	936.7	1.143925	0.032906	0.099885	0.201216	-2.31318

0.6407	63.83	5601.78	6536.18	109.43	38.44	3994.79	0.507584	0.336315	0.477523	2.9454	1.558463
0.6396	12011117	12361712	11287741	1453206	1302112	1242830	1.371329	0.0006	0.012748	8.919273	3.156926
0.6358	14761.01	9349.53	10655.65	8390.03	5443.23	7202.93	1.10141	0.088148	0.194627	1.652685	0.724812
0.6329	64461.69	63413.41	47585.45	183462.7	153586.6	144874	1.335306	0.005138	0.03368	0.364084	-1.45766
0.6322	6291.71	782.17	7039.03	5245.22	8039.43	8195.55	0.638897	0.347111	0.488083	0.657019	-0.60599
0.6318	7113.89	2089.23	8167.39	74891.58	59930.77	66923.56	1.301123	0.001547	0.017853	0.086101	-3.53783
0.6292	199728.8	48700.99	113175.7	5192.55	261041.1	2179.43	0.744582	0.768364	0.837115	1.347198	0.429962
0.6212	4059.79	4590.57	3612.81	1422.89	3533.1	5759.19	0.438839	0.723339	0.80414	1.144467	0.194676
0.62	41945.72	42327.94	47003.66	7136.13	5076.3	46322.06	0.942995	0.211079	0.350112	2.242734	1.165259
0.6172	15292.78	11092.73	15548.68	1717.75	1203.42	3355.18	1.306279	0.006378	0.037563	6.681302	2.740129
0.6158	13310.09	13971.97	12466.39	653.7	127.9	1240.43	1.272225	3.99E-05	0.004343	19.6577	4.297022
0.6095	12930	10143.71	13215.17	978.54	33107.03	4396.7	0.516548	0.949446	0.96329	0.943003	-0.08467
0.6081	89169.15	92436.06	55676.28	7623.47	11613.12	32500.45	1.204791	0.016127	0.062877	4.586298	2.19733
0.6071	7742.89	4567.1	3816.77	5927.59	5620.05	5949.01	0.396447	0.741279	0.81764	0.921706	-0.11762
0.6008	195702	130594.4	145177.2	307041.1	308913.5	349002.8	1.295795	0.003585	0.028059	0.488595	-1.03329
0.6006	14626.22	13512.86	15158.04	19723.48	18518.81	18772.63	1.32091	0.00221	0.021644	0.7594	-0.39707
0.597	8450.92	7035.78	4308.71	1548.52	1311.32	831.06	1.304719	0.044044	0.121282	5.363302	2.423121
0.5969	8915.72	7889.93	8565.83	74959.1	25362.57	69605.91	1.278505	0.09189	0.200676	0.149308	-2.74364
0.5928	1806.98	1871.49	2958.19	5810.67	6772.85	3080.13	1.126778	0.100555	0.211952	0.423698	-1.23889
0.5852	10762.02	14066.71	6277.09	11014.63	15982.86	10076.08	0.485079	0.532937	0.648652	0.83903	-0.25321
0.5842	73917.34	71716.81	81340.35	23138.99	11272.32	6345.58	1.272768	0.001241	0.016047	5.568985	2.477414
0.5797	22887.96	15450.93	18984.96	1839.19	1084.44	3027.31	1.324855	0.011225	0.05113	9.632739	3.267946
0.5792	7845.23	6374	6180.48	8850.65	6675.62	6819.4	0.482895	0.503722	0.62149	0.912916	-0.13145
0.5715	28015.94	26804.68	30021.87	10136.55	7009.15	7886.85	1.350402	0.000113	0.006218	3.389287	1.760982
0.5652	1605.03	1616.58	968.93	5151.39	5702.7	2113.83	1.140794	0.115048	0.232365	0.323147	-1.62974
0.5616	12000.79	26257.82	41331.88	35909.43	24251.48	61354.26	0.634805	0.373271	0.5107	0.654984	-0.61047
0.5591	1564.52	2966.73	125.28	4507.45	5155.36	4559.12	0.917884	0.0527	0.136331	0.327419	-1.61079

0.5573	69006.64	26741.76	71526.28	228062.8	226033	199264.8	1.252801	0.001504	0.017674	0.256022	-1.96566
0.5555	7612.8	6563.2	5743.91	572.9	254.02	121.75	1.321885	0.005004	0.0334	20.99772	4.392161
0.5533	12761.95	10249.84	22637.91	115.01	138.65	47.8	1.355936	0.057235	0.144358	151.4287	7.242495
0.5458	434.58	1513.77	239.75	300.46	101.7	4805.26	0.011977	0.58388	0.691424	0.420189	-1.25089
0.5446	17201.83	20353.73	20061.94	584.18	24013.06	29164.41	0.477553	0.897567	0.927626	1.071721	0.09993
0.5288	404.26	339.61	296.25	25944.93	25285.01	22725.8	1.371525	0.001612	0.018397	0.014064	-6.15184
0.5244	1982.1	112.61	190.57	471.58	1059.57	1929.29	0.649432	0.629163	0.728232	0.660402	-0.59858
0.5133	2946.24	6181.48	2500.15	4966.57	871.52	7631.01	0.101745	0.804229	0.862819	0.8633	-0.21207
0.5052	36851.73	33894.32	32162	62876.67	45014.91	54299.2	1.243681	0.053642	0.137774	0.634488	-0.65634
0.8851	253410.5	187361.6	226175.6	169035.1	197080	151825.8	1.007589	0.108405	0.223548	1.287691	0.364786
0.878	57605.39	59991.42	62135.77	20134.38	20843.57	3889	1.134992	0.011438	0.051506	4.005901	2.002127
0.8331	809512	1114411	719897.5	814780.3	718943.3	677177.5	0.689369	0.351754	0.492862	1.195811	0.25799
0.8202	4436085	3785804	3589755	1104.4	5429.85	7385.2	1.353496	0.004202	0.030476	848.5712	9.728892
0.7517	3027.73	501.72	334.69	38853.08	26669.34	40057.85	1.289894	0.012822	0.055229	0.036599	-4.77205
0.7213	8716.53	2545.25	4097.65	5016.5	76.91	2088.43	0.703288	0.313162	0.455677	2.138648	1.096699
0.7206	234688	205648.1	202605	150186.2	130880.4	138321.1	1.319156	0.006983	0.039121	1.533047	0.616402
1	14323.59	16248.57	20234.17	26427.95	38038.09	22382.6	1.116433	0.111378	0.227749	0.584999	-0.7735
0.9722	470121.6	501893.3	411981.9	1570037	1104261	1036646	1.31798	0.040798	0.115573	0.37295	-1.42295
0.8357	359979.5	633815.3	204281.5	96054.82	150123.8	197309.3	1.05363	0.177725	0.311501	2.701486	1.433753
0.8252	315922.9	301699.9	303598.7	327702.8	320986.3	315159.5	1.067971	0.071339	0.167366	0.955774	-0.06526
0.8053	202136.6	213267.6	36017.39	349618.9	295148.5	229762.1	0.832683	0.117868	0.235467	0.516188	-0.95403
0.7995	9290.24	2822.4	5155.62	79683.85	77767.59	89360.72	1.332107	0.000305	0.009631	0.069965	-3.83722
0.7921	10678.76	10732.2	11249.83	23796.17	20552.32	19427.57	1.348898	0.014216	0.058571	0.512117	-0.96546
0.771	492310.4	477051.3	444477.3	478643.6	451605.3	449221.8	0.430964	0.541694	0.656687	1.024914	0.035503
0.7695	326.18	1285.68	1557.15	7802.5	11392.29	9873.59	1.265315	0.008044	0.041941	0.109019	-3.19735
0.7551	3404.31	7356.11	6004.18	9682.9	5248.38	7645.78	0.653877	0.325602	0.467643	0.74255	-0.42944

0.7316	10637.01	21256.59	14290.62	27379.3	11931.64	22402.44	0.53613	0.407592	0.540357	0.748366	-0.41818
0.6919	353894	306164.9	325699.9	451060	394727.4	400733.8	1.224295	0.020541	0.073492	0.790808	-0.3386
0.6906	72713.7	45733.38	51585.25	37992.56	22933.42	34038.96	1.120884	0.072511	0.169569	1.790475	0.840342
0.5894	13263.76	11994.86	12836.36	5391.41	4478.24	4245.7	1.356539	0.0001	0.006073	2.698834	1.432336
0.5646	1115	708.46	177	2826.59	1137.34	2144.75	0.997316	0.089051	0.196146	0.327478	-1.61053
0.5463	45916.58	6220.97	24940.85	94986.26	6604.01	57529.65	0.354887	0.407005	0.540071	0.484404	-1.04572
0.5143	3490.39	313.39	285.64	5271.15	212.07	1786.02	0.329859	0.597513	0.702227	0.562565	-0.82991
0.5046	3680.75	3979.03	2919.62	465.5	867	1901.53	1.164307	0.012145	0.053419	3.271275	1.709853
